# Machine-learning strategies for testing patterns of morphological variation in small samples: sexual dimorphism in gray wolf (*Canis lupus*) crania

**DOI:** 10.1186/s12915-020-00832-1

**Published:** 2020-09-03

**Authors:** Norman MacLeod, Liora Kolska Horwitz

**Affiliations:** 1 School of Earth Science and Engineering, Zhu Gongshan Building, Nanjing University, 163 Xianlin Avenue, Nanjing, 210023 Jiangsu China; 2 National Natural History Collections, Faculty of Life Sciences, The Hebrew University of Jerusalem, The Edmond J. Safra Campus - Givat Ram, 9190401 Jerusalem, Israel

**Keywords:** Carnivores, Morphometrics, Machine learning, Automated identification, Convolution neural networks, Ecomorphology, Shape analysis

## Abstract

**Background:**

Studies of mammalian sexual dimorphism have traditionally involved the measurement of selected dimensions of particular skeletal elements and use of single data-analysis procedures. Consequently, such studies have been limited by a variety of both practical and conceptual constraints. To compare and contrast what might be gained from a more exploratory, multifactorial approach to the quantitative assessment of form-variation, images of a small sample of modern Israeli gray wolf (*Canis lupus*) crania were analyzed via elliptical Fourier analysis of cranial outlines, a Naïve Bayes machine-learning approach to the analysis of these same outline data, and a deep-learning analysis of whole images in which all aspects of these cranial morphologies were represented. The statistical significance and stability of each discriminant result were tested using bootstrap and jackknife procedures.

**Results:**

Our results reveal no evidence for statistically significant sexual size dimorphism, but significant sex-mediated shape dimorphism. These are consistent with the findings of prior wolf sexual dimorphism studies and extend these studies by identifying new aspects of dimorphic variation. Additionally, our results suggest that shape-based sexual dimorphism in the *C. lupus* cranial complex may be more widespread morphologically than had been appreciated by previous researchers.

**Conclusion:**

Our results suggest that size and shape dimorphism can be detected in small samples and may be dissociated in mammalian morphologies. This result is particularly noteworthy in that it implies there may be a need to refine allometric hypothesis tests that seek to account for phenotypic sexual dimorphism. The methods we employed in this investigation are fully generalizable and can be applied to a wide range of biological materials and could facilitate the rapid evaluation of a diverse array of morphological/phenomic hypotheses.

## Background

Among the more prominent and long-standing questions in evolutionary biology are whether sexual dimorphism in the vertebrate skeleton is the product of selection for specific features or general body size and whether different aspects of sexual dimorphism are correlated with different aspects of a species’ ecology, behavior, phylogeny, life history, etc. An understanding of sexually dimorphic patterns in vertebrate species also has implications for understanding the evolutionary history of biodiversity. More practically, methods for analyzing patterns of morphological variation—such as sexual dimorphism—in small samples have implications for improving the value and research utility of museum collections. In the case of humans, such analyses have an additional forensic utility [1, 2].

Most biometric studies of mammalian carnivores have concentrated on interspecific differences in the form of skulls and teeth, along with the biomechanical and behavioral correlates that account for differences in hunting and killing strategies [3–8]. Relatively few investigations have focused on the biometric analysis of intraspecific differences [9–19]. Gittleman and Van Valkenburgh [13] found that, in contrast to most other mammals, the predominant pattern of sexual dimorphism in carnivore canine tooth size was correlated with breeding system while a lesser dimorphism in carnassial tooth size was associated most strongly with diet. These authors also found greater dimorphism was exhibited by large-prey pack hunters whose diets consisted of greater than 70% meat (e.g., wolves) and suggested this pattern of form differentiation was associated with breeding system, including the degree of intramale competition for access to females, possibly reinforced by adaptations that promote solitary (as opposed to pack) hunting. The lesser correlation with diet that was noted remains unexplained from mechanistic, ecological, or evolutionary standpoints.

Alternatively, dimorphic differences in the morphology of wolf post-cranial skeletons have been related to conspecific aggression and competitive behavior trends in males [18, 19]. Wild canids differ in the prominence of sexually dimorphic maxillary and dental forms [11], which may be associated with certain hunting strategies [17, 20–22]. As male wolves typically outperform females in hunting, this difference would be expected to be reflected in their dentition as well as in other post-cranial skeletal elements and soft tissue composition [18, 19, 23].

Further, the tendency toward tooth dimorphism in canids may have broader behavioral origins. For example, among wild wolves, there exists a clear division of labor, with females predominantly engaging in pup care and defense while males focus on foraging and food provision [17]. Kieser and Groeneveld [9] suggested that larger post-canine tooth size in female carnivores matches the higher masticatory demands associated with lactation and pregnancy.

In the study of carnivores, it is, perhaps, understandable that the forms of the teeth and post-cranial skeletal elements have often been the primary targets of analysis (e.g., [13, 18, 19]) and that the geometries of these structures have been summarized typically by a very small number of traditional linear measurements (e.g., mediolateral breadth, anteroposterior length), along with lengths and breadths of various long bones. More recently, investigations have employed landmark-based sampling strategies to summarize patterns of skeletal variation across larger and more complex skeletal structures [15–17].

The use of topologically homologous landmark locations in studies such as these has resulted in more geometrically faithful representations of form than those afforded by linear distance measurements because aspects of these forms remained unsampled under that approach, and so remained unanalyzed. This is especially true for the geometric character of form outlines which are used routinely by many taxonomists to distinguish mammalian species. In addition, the avoidance of representational bias, introduced as a result of the clumping of landmarks together in particular regions of the forms under consideration, can pose a considerable challenge when designing landmark-based sampling strategies. In several of the investigations cited above, bias may also have been introduced inadvertently owing to landmarks being placed on only one side of a bilaterally symmetric skeletal element such as the cranium in ventral view ([15]; see also [24, 25]).

Despite these methodological issues, Schutz et al. [15], for example, were able to demonstrate pelvic dimorphism in both of the *Urocyon* species evaluated, presumably related to offspring size at birth. However, no significantly dimorphic trends were detected in their set of cranial landmarks. Extrapolating from such results, these investigators concluded that dimorphic trends were focused in particular regions of the body in both species, as opposed to being the result of whole-body allomorphic effects associated with body-size dimorphism. A second example is provided by Milenković et al. [16], who focused on differences between mixed-sex Dinaric-Balkan and Carpathian wolf populations. These authors concluded that both cranial and mandibular size and shape differences were present and postulated that they arose due to isolation in separate glacial refugia, strengthened later by differences in environmental and social behavior factors.

The skull and pelvis have long been recognized as aspects of the mammalian skeleton in which dimorphic differences might be expected to be expressed. But even within the context of these elements, the manner in which their morphology has been sampled has, in effect, been controlled by a priori decisions reflecting either systematic tradition [9, 10, 13, 16, 18, 19] or geometric morphometric formalism [15, 16]. As a result, sexually dimorphic patterns of shape variation in the canine tooth were not assessed by Schutz et al. [15] because a decision had been made to represent the position, but not the form of this aspect of *Urocyon* morphology by a single landmark. In the Milenković et al. [16] investigation, over 50% of the cranial landmarks were confined to the facial region with no landmarks constraining variation in the braincase and only a single landmark used to quantify the position of the zygomatic arch. Similarly, these authors used over 50% of their mandibular landmarks to quantify aspects of the dental arcade with only single landmarks constraining the positions, but not the forms, of the coronoid, condyloid, and angular processes. Gittleman and Van Valkenburgh [13] restricted their quantification of dimorphic patterns to linear dimensions of the vacuity—or socket—that contained the canine tooth. Yet, despite these representational limitations, the authors cited above, along with many others, discussed their results as if they constituted generalized proxies for overall morphological trends characterizing the species in question.

Our purpose in calling attention to these issues is not to criticize the authors of these investigations. Sampling strategies identical to those they employed can be found across the biological literature. Moreover, we regard their results as perfectly correct and valid in the context of the measurements they took and for the samples from which those measurements were obtained. In all cases, these researchers’ approaches to the analysis of their data can be cited as examples of biometric best practice within the multivariate morphometric and geometric morphometric traditions they represent. Rather, we wish to address the larger issue of whether the range of approaches most researchers use currently to characterize patterns of morphological variation can, or should, be regarded as sufficient to address the generalized morphological questions we all seek to answer. Furthermore, is the representation of morphology using small sets of traditional linear distances, small sets of landmarks, or relatively small sets of boundary outline semilandmarks in two or three dimensions really “the best we can do”? We also feel it is important to explore how the conclusions reached by morphometric investigations are shaped by the data investigators choose to collect and the procedures they select to provide analytic summaries of the patterns inherent in such data. Broadly then, we seek to explore whether the morphometric data and data-analysis approaches employed in these, and many other, morphometric investigations were truly adequate to the task of finding and identifying generalized and representative patterns of morphological variation.

Resolution of these larger questions demands the employment of truly generalized, descriptive approaches to the characterization of morphological variation. What is needed is a comprehensive strategy for assessing any aspect of hard or soft tissue anatomy quantitatively for the geometric signature of similarity or difference. Simply put, such a strategy should seek to extract as much information relevant to the hypothesis test(s) under consideration, from the data available. Ideally, such a strategy should allow for the post hoc discovery of specific anatomical region(s) where differences are manifested as well as enabling both broad, and/or specific, differences to be identified and evaluated, via objective statistical hypothesis testing. A successful exploratory morphometric research strategy of this nature should also be able to accommodate minor imperfections in the specimens under consideration, as well as being flexible enough to evaluate a wide range of structural, textural, color, and size and shape differences, any or all of which may have played roles in developmental, morphological, ecological, and/or behavioral evolution [26]. Finally, this strategy should be able to obtain statistically robust results from either large, generalized, or small, localized samples as the latter are often the only data to which researchers have access.

Gray wolves (*Canis lupus*) are known to be dimorphic in a large number of body dimensions and weights (body length, contour length, tail length, body length, humerus length, ulna-radius length, femur length, tibia-fibula length, girth, neck, heart mass, liver mass, lung mass, spleen mass, kidney mass; see [23] and references therein, [27–29]). Nonetheless, it is unknown presently whether this dimorphism is simply a reflection of generalized size differences or reflects sex-specific developmental trajectories. In this investigation, we conducted a search for sexually dimorphic form differences using a small sample of modern gray wolf crania collected from northern Israel and the Golan Heights (henceforth “Israeli wolves”) for the purpose of determining whether recent developments in the field of geometric morphometrics and computer vision (e.g., [30–33]) could support more exploratory and confirmatory approaches to the analysis of sexual dimorphism in carnivore skeletons. Previous applications of this approach have proved useful in social media [34] and the scientific fields of entomology, where they have, in part, been employed to discover an unexpectedly strong set of sexually dimorphic differences in the morphology of fly wings [35], in the study of Mullerian mimicry in butterflies [36], and in archeology where they have been used to assess both temporal [32] and regional geographic differences [37] in the forms of lithic artifacts.

## Results

### Size variation

**Fig. 1:**
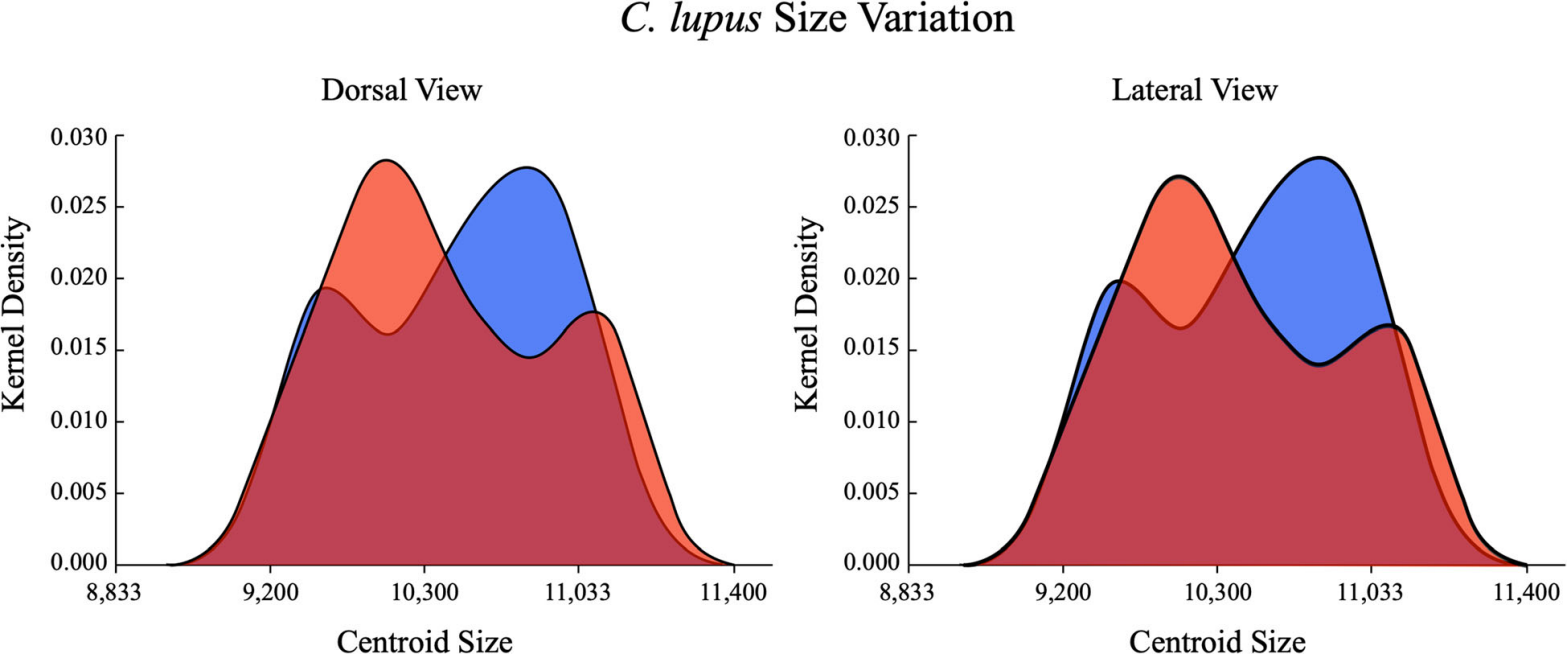
Smoothed histograms of size estimates of male (blue) and female (red) Israeli *C. lupus* crania obtained from 300 equally spaced semilandmark dorso-ventral and lateral outlines. Despite expected differences in sex average and modal values, the distribution distinctions between both these distributions are insufficient to support an interpretation of sex-based cranial size differences for this sample, based on both parametric (2-sample *t* test) and non-parametric (Mann-Whitney test) results

Sex-based, cranial centroid size data were tabulated for both dorso-ventral and lateral cranial outlines and tested statistically for sexual dimorphism. The smoothed histograms for both datasets were virtually identical (Fig. 1). This strong similarity was expected, to some extent, by the small sample size as well as by the large and constant number of semilandmark coordinate points used to quantify size variation. Nonetheless, congruence between these dorso-ventral and lateral cranial size distributions provides confidence that comparable patterns of spatial variation are being reflected in both views. This similarity also extended to the statistical test results as the null hypothesis of no sex-based outline size difference was accepted for both views (*t* test, *p* value = 0.737; M-W test, *p* value = 0.741 for both dorso-ventral and lateral views).

### Shape variation

Distinctions between sex-based patterns of shape variation were assessed using two different datasets and three different data-analysis procedures: elliptical Fourier analysis (EFA) of the cranial outlines in dorso-ventral and lateral views, Naïve Bayes (NB) machine-learning analysis of the same set of EFA harmonic coefficients, and deep-learning analysis (LeNet-5 CNN) of cranial images.

### Elliptical Fourier analyses

#### Dorso-ventral view results

A preliminary principal component analysis (PCA) transformation of the 177 elliptical Fourier variables indicated nine orthogonal axes (= eigenvectors) were needed to represent 95% of the pooled shape-covariance structure for these data. The quality of this decomposition is indicated by calculating the correlation between the original covariance matrix and the covariance matrix estimated on the basis of the nine retained eigenvectors in the manner of a cophenetic correlation coefficient. For these data, that value is 1.000.

**Fig. 2:**
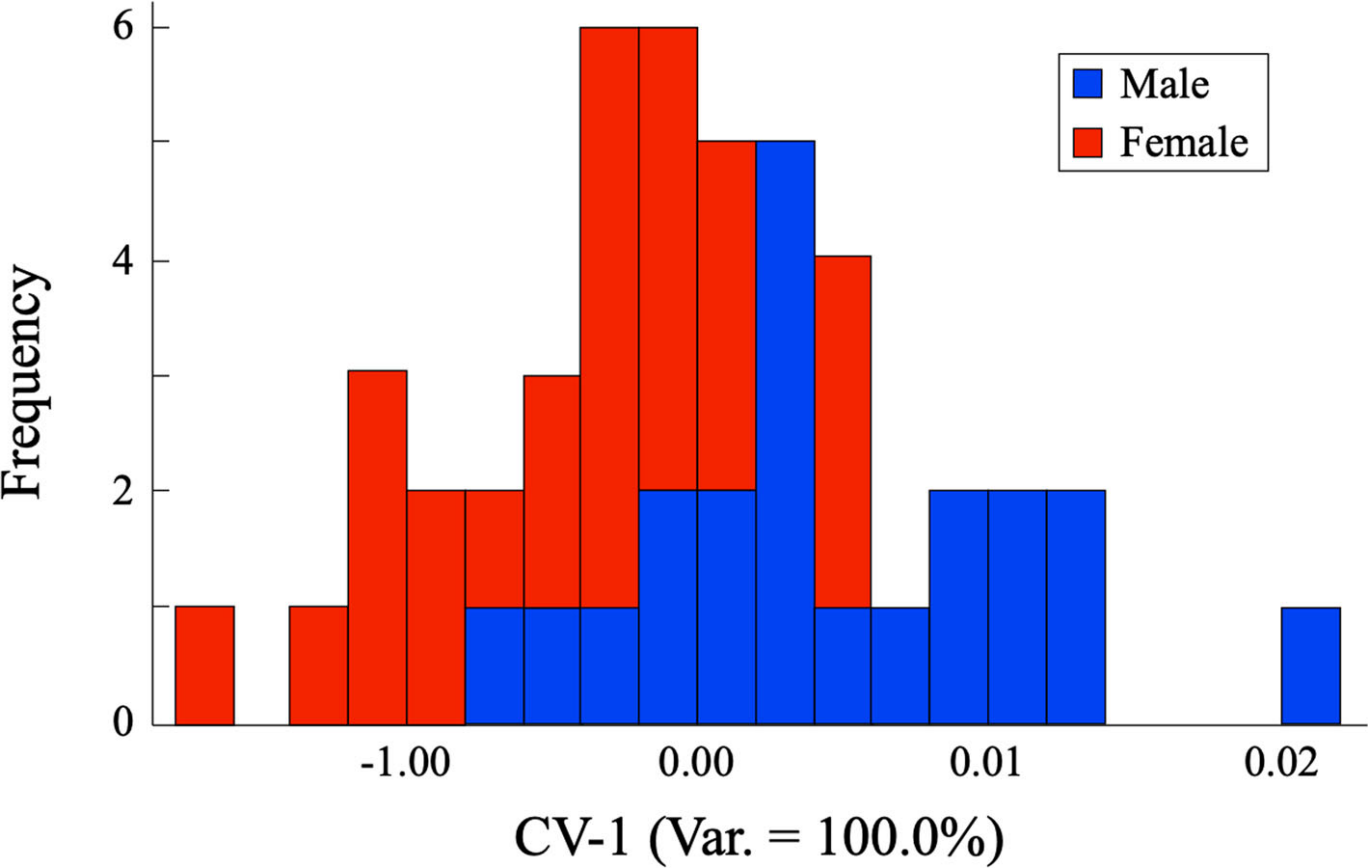
Stacked frequency histogram of shape similarity estimates of male (blue) and female (red) Israeli *C. lupus* crania obtained from elliptical Fourier harmonic data that model dorso-ventral cranial outlines. Note that almost 70% of the data fall into the region of overlap between male and female shape distributions. Despite expected differences in sex modal values, the distribution distinctions between these distributions are insufficient to support an interpretation of sex-based cranial size differences for this sample

Because only two subgroups are involved, a single canonical eigenvector (CV-1) was extracted from the projected coordinate positions of the outline shape descriptors in this nine-dimensional PC space. This discriminant axis represents the linear, major axis regression joining the two group centroids within a transformed space that represents the optimal between-group separation relative to within-group dispersion in a least-squares sense [38, 39]. The histogram of dorso-ventral view cranial shape outlines projected onto this discriminant axis is shown in Fig. 2.

While sex-based shape differences were recovered by the analysis of these EFA data, the majority occupy the region of overlap between the male and female cranial outline shape distributions. Thus, clear distinctions between characteristically male and characteristically female dorso-ventral outline shapes would only be possible for quite a small proportion of this sample. Moreover, owing to the predominance of female cranial outlines occurring within this region of overlap, there would be a tendency to associate intermediate shapes with the female category despite the fact that this would be an incorrect assignment for a substantial proportion of the sample.

The confusion matrix calculated for the canonical variate analysis (CVA) solution of these EFA dorso-ventral outline data is provided in Table 1. Given the degree of overlap between the sex classes shown in Fig. 2, the CVA confusion matrix results for these data are surprisingly good with over three quarters of the training set being assigned to their correct categories. However, a 78% identification accuracy ratio would be considered marginal by most taxonomists, especially if it could not be reproduced for genuine unknowns (= specimens that were not used to train the discriminant system).

**Table 1:** Confusion matrix for CVA of *C. lupus* elliptical Fourier harmonic data (dorso-ventral view). True identifications are shown in the table rows, post hoc assigned identifications in the table columns

Group	Female	Male	Total correct	Class totals	Percent correct
Female	15	6	15	21	71.43
Male	4	21	21	25	84.00
Total correct	15	21	36	46	78.26
Total estimated	19	27	46		
Percent correct	78.95	77.78	78.26		

The subtlety of the secondary sexual characteristics inherent in this CVA solution also deserves comment. If morphological distinctions between sexes are not pronounced, it may be questioned whether they actually exist at all or, rather, simply reflect an incidental by-product of the small sample size. Uncertainties in this context might arise from, in the case of these Fourier harmonic data, inclusion of non-representative individuals in the sample, re-description error resulting from the a priori selection of a subset of the available harmonic series, etc. This aspect of the analysis is especially problematic since only a limited aspect of the overall morphology (in this case, the dorso-ventral cranial outline) is being subjected to analysis.

**Fig. 3:**
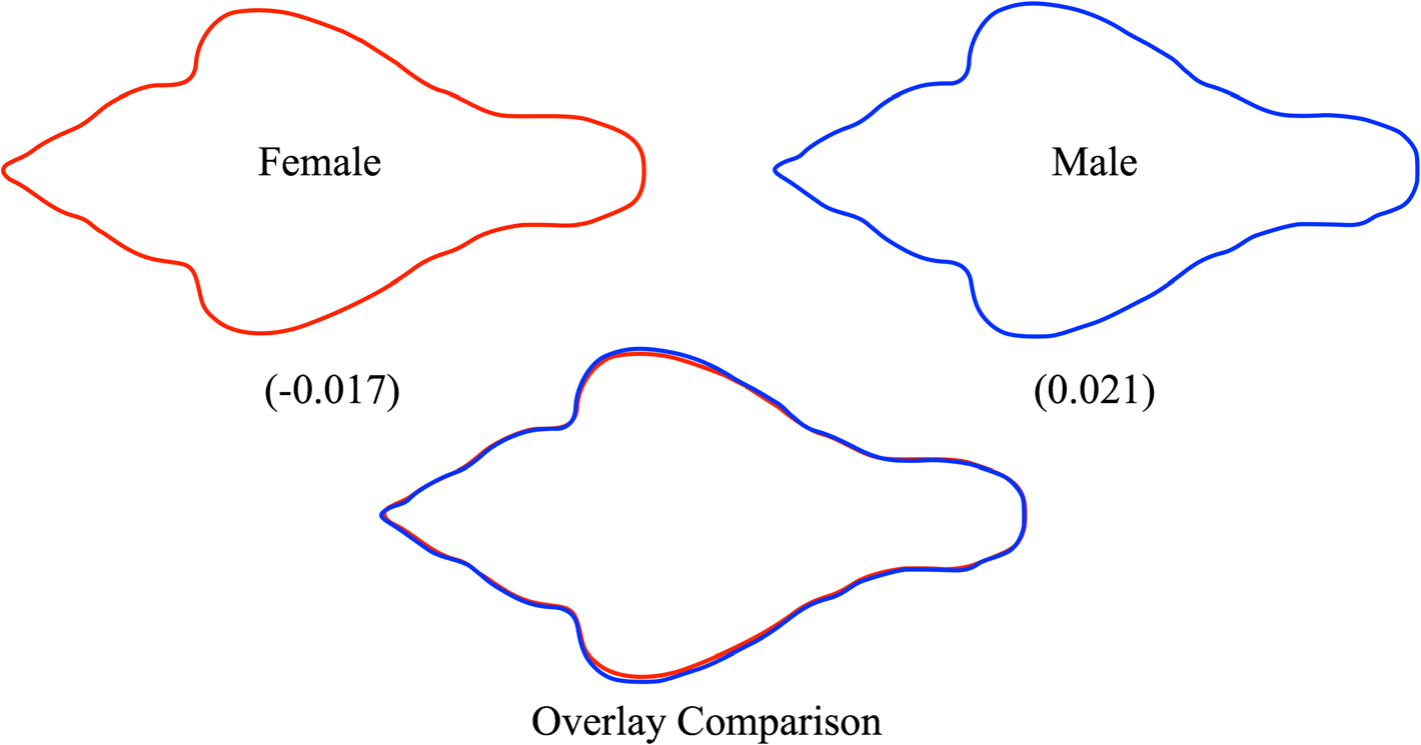
Dorso-ventral view shape models reconstructed at the extreme ends of the distribution of EFA outline data projected onto the single canonical variate eigenvector (CV-1, see Fig. 2). Note that even at these extremes, the shape distinctions between characteristic male and female morphologies are quite small for this dataset and focus primarily on the lateral extent of the zygomatic arches. It is exceedingly doubtful that sex-based discriminations of this nature could be made reliably by eye. Moreover, since these outlines were interpolated from a subset of the Fourier harmonic data available for these outlines, there is also a question as to whether this putative difference is artifactual, arising as a result of the interaction between the small sample size and the interpolations inherent in shape reconstruction from Fourier harmonic data

For landmark and semilandmark datasets, procedures exist that enable models to be created for the geometric shapes that lie at any coordinate location with CVA (or PCA) eigenvector-defined spaces. Figure 3 illustrates shape models for the EFA-described dorso-ventral view cranial data at coordinate positions corresponding to the extreme negative (female) and extreme positive (male) termini of the projected CV-1 score distributions.

As can be seen in Fig. 3, the dorso-ventral view shape distinctions inherent in this Israeli sample are quite minor, involving the lateral extent of the zygomatic arches predominantly. Given such a fine distinction, the influence of even a single aberrant specimen—such as the single male outlier in Fig. 2—can exert a substantial and possibly confounding influence on the calculated result. This is not to say this difference is necessarily erroneous, only that such group-distinction results cannot necessarily be taken at face value with confidence.

This ambiguity is further reflected in the results of standard statistical tests of the mean vector separations relative to sample variances along the CV-1 axis. Parametric versions of Hotelling’s *T*^2^ and the log likelihood ratio (*ϕ*) tests both reject the null hypothesis of no difference between mean vectors at the 95% confidence level (*T*^2^ = 24.50, *p* value = 0.043; *ϕ* = 17.49, *p* value = 0.042). However, both tests assume multivariate normality of the distribution of means, equal sample sizes, and equal covariance matrices. A more conservative, non-parametric, 1000-iteration, bootstrap evaluation of Hotelling’s *T*^2^ and the log likelihood ratio tests for these same data accepts the null hypothesis for the former, but rejects it for that latter (*T*^2^ = 24.50, *p* value = 0.039; *ϕ* = 17.49, *p* value = 0.055). On the basis of these results, the significance of the dorso-ventral cranial outline shape difference, as assessed by the EFA-PCA-CVA strategy, must be considered marginal, at best.

#### Lateral view results

Analysis of the lateral view EFA harmonic coefficients proceeded in a manner identical to that of the dorso-ventral view. Once again, 45 harmonic coefficients were extracted from the training set of 46, 300 semilandmark-constrained outlines, yielding a total of 177 EFA harmonic variables. A preliminary PCA transformation indicated that 16 eigenvectors were required to account for 95% of the pooled shape-covariance structure. The quality of this decomposition was assessed in the manner described above. For the 16 retained-eigenvector solution, the value of the cophenetic correlation coefficient was 1.000.

**Fig. 4:**
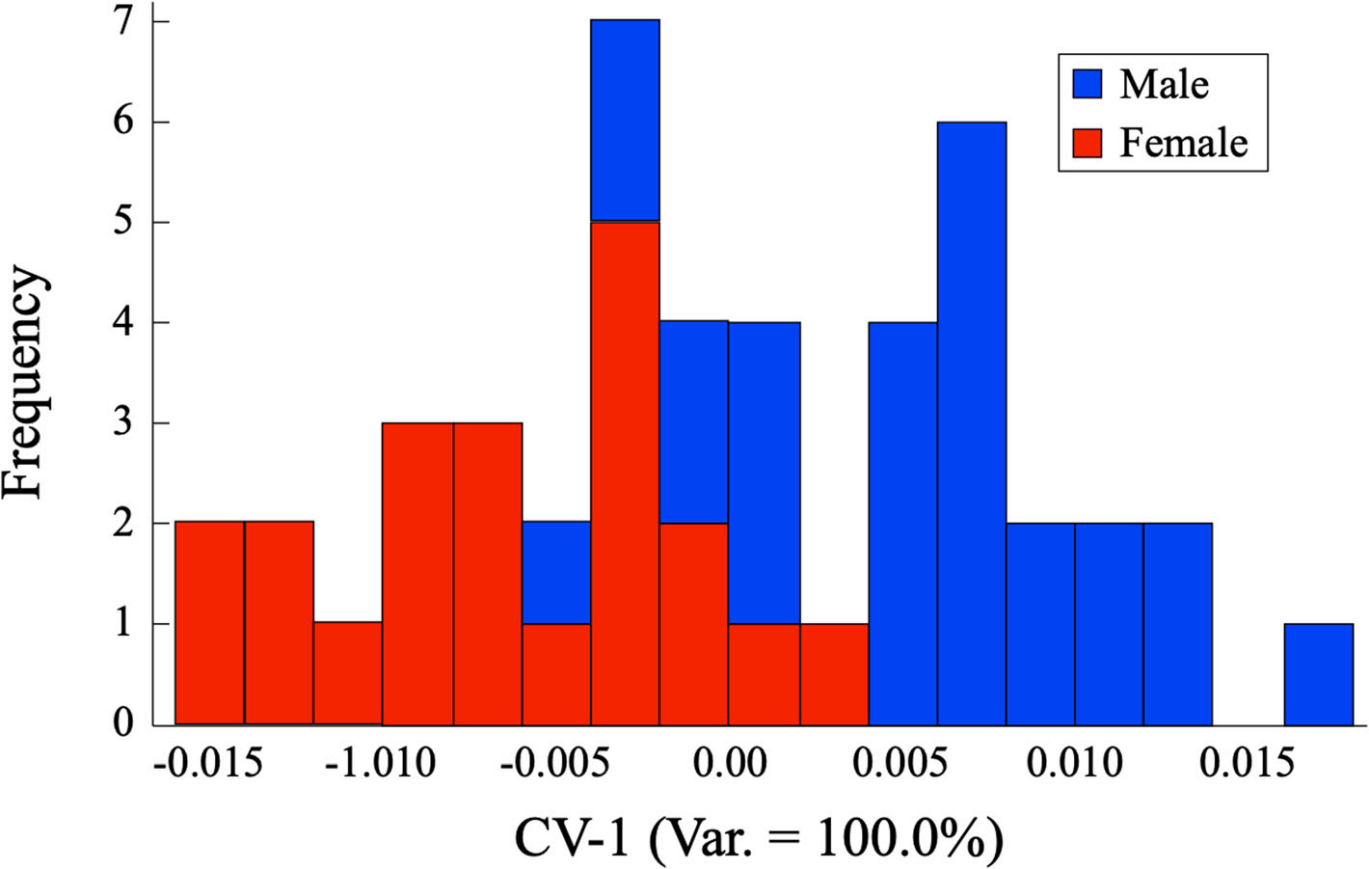
Stacked frequency histograms of shape similarity estimates of male (blue) and female (red) *C. lupus* crania obtained from elliptical Fourier harmonic data that model dorso-ventral cranial outlines. Note that almost 70% of the data fall into the region of overlap between male and female shape distributions. Despite expected differences in sex modal values, the distribution distinctions between these distributions are insufficient to support an interpretation of sex-based cranial size differences for this sample

Subsequent to the PCA transform, a CVA was applied to these data in order to achieve an optimized representation of sex-specific cranial shape differences. These differences were assessed initially via inspection of the histogram of male and female lateral view outline scores on the single discriminant axis that resulted from this analysis (Fig. 4).

As in the previous dorso-ventral-outline analysis, sex-based shape differences are clearly evident in the lateral views of these crania. Interestingly, the separation between sexes appears to be better for the lateral, than for the dorso-ventral, views, with a distinct minority of lateral outlines residing in the overlap interval. This might be regarded as a remarkably good result when it is recalled that no effort was made to improve the separation between sexes by eliminating the contribution of the dental arcade which exhibited variation in presence, preservation, damage, and tooth wear. Still, 39% of the sample occupies the interval in which the sex assignment could be regarded as ambiguous. Within this overlap interval, male and female outlines are subequally represented (7 and 9 outlines, respectively). This (sub)equality, if anything, renders qualitative interpretation of outlines exhibiting shape variation modes projected into the overlap zone even more problematic.

Table 2 summarizes the post hoc assignments of these training-set outlines to sex-based classes on the basis of their CV-1 scores. As is suggested by a close comparison of Figs. 4 and 6, the discriminant axis for the lateral outlines did a better job of allocating outlines to their correct sex classes. But given the small size of the sample, this achievement must also be regarded as disappointing as it involved only two outlines that were allocated incorrectly in the dorso-ventral view analysis, but correctly in the lateral view analysis. Moreover, an error ratio of 17% would still be regarded as of marginal quality since this ratio refers to the same set of outlines that was used to train the discriminant system.

**Table 2:** Confusion matrix for CVA of *C. lupus* elliptical Fourier harmonic data (lateral view). Row and column counts as in Table 1

Group	Female	Male	Total correct	Class totals	Percent correct
Female	18	3	18	21	85.71
Male	5	20	20	25	80.00
Total correct	18	20	38	46	82.61
Total estimated	23	23	46		
Percent correct	78.26	86.96	82.61		

**Fig. 5:**
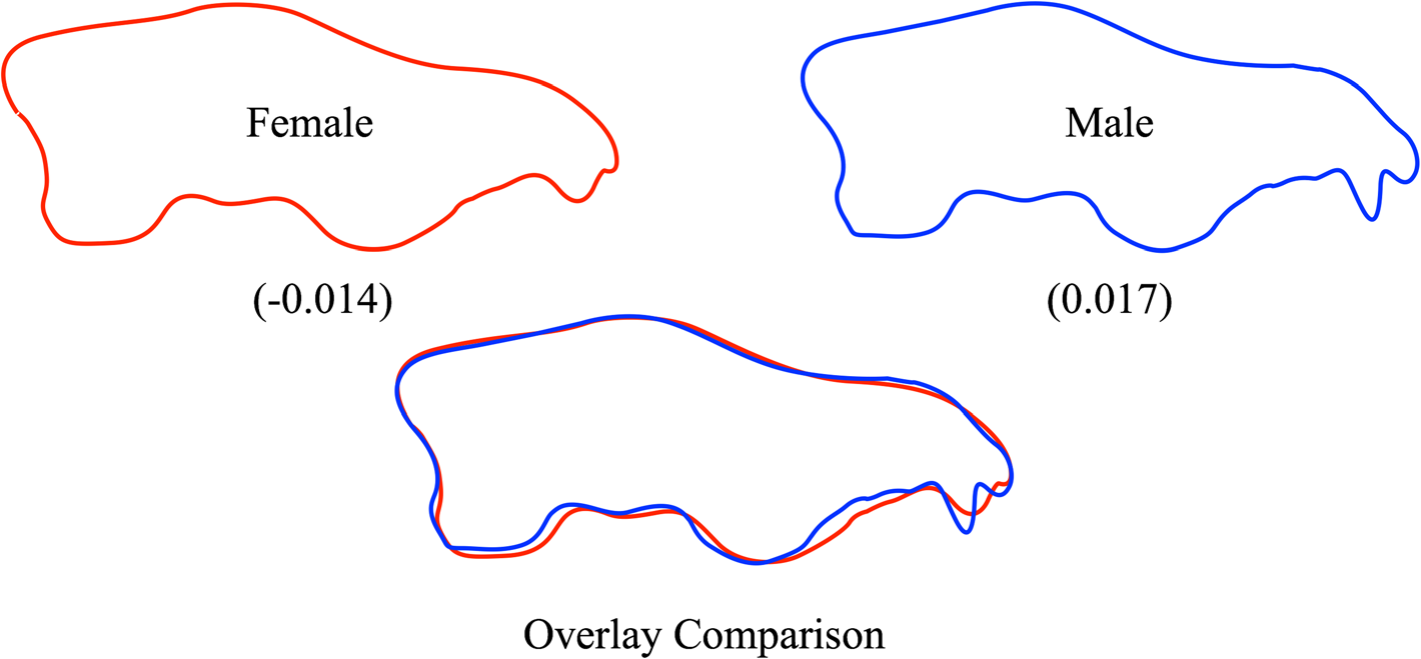
Lateral view shape models reconstructed at the extreme ends of the distribution of EFA outline data projected onto the single canonical variate eigenvector (CV-1, see Fig. 4). Note that the shape distinctions between characteristic male and female morphologies are larger than they were for the dorso-ventral view analytic result (see Fig. 3) and, to some extent, involve obviously artifactual aspects of the morphology (e.g., the excessively worn canine and incisor teeth characteristic of females). It is exceedingly doubtful that sex-based discriminations of this nature could be made reliably by eye. Nonetheless, these results also capture distinctions between male and female cranial shapes that may represent more biologically important aspects of sex-based cranial shape variation (e.g., structure of the nasal opening, aspects of the occipital condyle and pterygoid flange lateral outlines)

Extreme position shape models for the lateral view discriminant axis are illustrated and compared in Fig. 5. While this comparison identifies several regions in which lateral outline shape is markedly divergent between sexes, it also represents a complex mixture of differences, some that are likely artifactual in the sense of their not being part of any fundamental aspects of either sex’s biology, together with the set of valid biological differences.

With regard to the former, five female specimens exhibit absent canine teeth and three exhibit missing incisor teeth, as opposed to one specimen in each of these categories for males. Sex-based discrepancies in the presence and character of the incisor and molar teeth are also part of this sample’s composition. Accordingly, this aspect of the discriminant system should be regarded as an artifactual attribute of the particular specimens analyzed rather than being a generalized difference between male and female Israeli *C. lupus* phenotypes.

This issue could be addressed by selecting an outline data-analysis procedure that does not require that boundary outlines be closed (e.g., eigenshape analysis, see [40–42]) or by doubling an incomplete boundary outline back on itself in order to create an artificially closed outline and calculating the EFA harmonics associated with this “force fit” complete outline [43]. The former solution is viable and efficient; the latter has the disadvantage of somewhat artificially inflating the number of semilandmark coordinates required to represent for shape and so the number of Fourier harmonic coefficients required to characterize each boundary outline.

Leaving complications in the degree of dental arcade preservation in this sample aside, the EFA-PCA-CVA data-analysis strategy did reveal consistent, sex-based differences in well-preserved regions of the lateral cranium outline. These focus on the height of the snout (higher in extreme males), prominence of the anterior dorso-ventral part of the cranium formed by the zygomatic processes (placed higher and positioned more anteriorly in males), and relative size and position of the occipital condyle (slightly smaller and placed more posteriorly in males). Overall, these differences appear to be associated with a slightly more rugged construction of male crania relative to the condition seen in extreme females. Most importantly, these associations are consistent with the different ecologic and behavioral roles of male and female gray wolves (see the “Materials” section).

Morphological distinctions between male and female crania of this species are further reflected in the results of statistical tests for sex-based mean vector differences relative to sample variances along the CV-1 axis. For these lateral outline data, parametric versions of Hotelling’s *T*^2^ and log likelihood ratio tests reject the null hypothesis of no difference between mean vectors at the 95% confidence level in both cases (*T*^2^ = 52.25, *p* value = 0.035; *ϕ* = 28.18, *p* value = 0.030). The more conservative, non-parametric, bootstrap-based evaluations of these same statistics also rejected the null hypothesis in both instances and improved the confidence of this rejection (*T*^2^ = 52.25, *p* value = 0.028; *ϕ* = 28.18, *p* value = 0.025).

### Traditional machine-learning (Naïve Bayes) analysis

In order to explore how alternative modes of outline analyses might perform on these data, the NB algorithm was used to analyze the PCA-transformed scores for the EFA coefficients of the same Israeli *C. lupus* dataset in both dorso-ventral and lateral views. This test examined the effect of altering the procedure used to find the best between-class discriminant decision axes within complex geometric morphometric contexts.

#### Dorso-ventral view results

Table 3 summarizes results of the application of the EFA-PCA-NB machine-learning procedure to the dorso-ventral view, cranial data. Obviously, the improvement in discriminant performance is due to resolution of fully half the previous analysis’ sex-class allocation errors by this alternative data-analysis algorithm.

**Table 3:** Confusion matrices for analysis of *C. lupus* dorso-ventral view, PC-transformed elliptical Fourier harmonic coefficient data using the Naïve Bayes machine-learning procedure. Row and column counts as in Table 1

Group	Female	Male	Total correct	Class totals	Percent correct
Female	19	2	19	21	90.48
Male	3	22	22	25	88.00
Total correct	19	22	41	46	89.13
Total estimated	22	24	46		
Percent correct	86.36	91.67	89.13		

Of the ten specimens misidentified by the EFA-PCA-CVA (M07791 ♀, M07924 ♂, M07941 ♂, M08039 ♂^♰^, M08266 ♀, M09181 ♀, M11684 ♀, M12130 ♂, M12476 ♀, M12477 ♀), only two (M07791 ♀ and M07924 ♂) were also misidentified by the NB analysis. Moreover, whereas the majority of CVA-misidentified specimens were female, males formed the majority of misidentifications in the NB result. Among the specimens misidentified by the CVA algorithm, all differ from one another, but a few exhibit unusual morphologies or obvious damage. Specimen M07791 ♀ exhibits a markedly recurved aspect to its squamosal, M08039 ♂^♰^ and M11684 ♀ possess very well-developed and robust frontals, and the dorso-ventral view of M08039 ♂^♰^ seems narrower and more gracile than other male specimens. Nonetheless, it is difficult to infer exactly why these specimens were misidentified whereas others, with apparently similar or other equally idiosyncratic dorso-ventral cranial shapes, were identified correctly. In part, this probably reflects the subtlety of the sex-based morphological distinctions recovered by EFA and over which the question of statistical significance also hangs.

With regard to the specimens misidentified by the NB analysis, the interpretation based on qualitative inspection of the outlines themselves seems much more straightforward. The posterior inclination of the M07791 ♀ zygomatic arch has already been noted, and this structure identified as of potential importance in the identification of Israeli *C. lupus* sex distinctions. Specimen M08207 ♀ is a powerfully built female with wide zygomatic arches whereas M07924 ♂ is a male of decidedly more gracile aspect. By far, the most obvious problematic specimen in the entire sample, though, is M11108 ♂ which exhibits a distinctly deformed dorso-ventral morphology with very narrow and upturned zygomatic arches. This specimen also displays a distinct right-left asymmetry. Indeed, the fact that this specimen was identified correctly by the CVA seems even more counterintuitive in retrospect.

Despite the lack of sophisticated results-visualization procedures for the NB discriminant solution, the results of this analysis appear more readily understandable and interpretable than those of the CVA. In addition, the fact that such different sets of morphologies were misidentified by these two classifiers suggests they are either assessing different aspects of the dorso-ventral outline and using those to construct their discriminant axes or giving more weight to the variables that represent changes in zygomatic arch shape.

With regard to statistical significance, this also differs between the CVA and NB analysis. Statistical tests of the CVA results returned marginally significant values for the parametric versions of Hotelling’s *T*^2^ and log likelihood ratio tests and mixed results for their alternative, 1000-iteration bootstrapped versions. Hotelling’s *T*^2^ test is not available for the NB analysis, but both the parametric and bootstrapped versions of the log likelihood ratio (*ϕ*) test were markedly significant with *p* values of less than 0.001 (*ϕ* = 15.581).

#### Lateral view results

Table 4 summarizes results of the application of the EFA-PCA-NB machine-learning procedure to the cranial lateral view data. As was the case for the dorso-ventral view, changing the manner in which the EFA-PCA-described lateral outline data were analyzed for between-class differences resulted in a dramatic improvement in the results, producing allocation accuracy values approaching those that would be considered compelling by most practicing systematists (Table 4).

**Table 4:** Confusion matrices for analysis of *C. lupus* lateral view, PC-transformed elliptical Fourier harmonic coefficient data using the Naïve Bayes machine-learning procedure. Row and column counts as in Table 1

Group	Female	Male	Total correct	Class totals	Percent correct
Female	19	2	19	21	90.48
Male	1	24	24	25	96.00
Total correct	19	24	43	46	93.48
Total estimated	20	26	46		
Percent correct	95.00	92.31	93.48		

Comparison of Table 4 with Table 2 shows that, for these lateral outline data, a greater than 50% improvement in identification accuracy was achieved by relaxing the constraint of strict geometric linearity in the formulation of discriminant decision axes. This result confirms that, despite the complications introduced by the variable presence and preservation states of the teeth, more sex-specific shape information appears to be present in the lateral view of *C. lupus* crania than in the dorso-ventral view. Since wolf crania are bilaterally symmetrical, this difference may reflect the fact that a significant proportion of the information presented in the dorso-ventral view was redundant for the purpose of identifying sex differences. Departures from strict symmetry are, of course, present in each specimen. Since no aspect of the cranial morphology in lateral view is characterized by any symmetry, however, the effective information content of the lateral, as opposed to the dorso-ventral, representations of *C. lupus* cranial morphology would appear to be greater on both theoretical and (now) empirical grounds.

Once again, it is instructive to compare the misidentified training-set specimens in both analyses. Consistent with the independence of patterns of morphological misidentifications between alternative discrimination procedures seen in the dorso-ventral view analysis, there is no overlap between the four outlines misidentified by the lateral view CVA solution and the three outlines misidentified by this NB analysis. In contrast, two of the three outlines misidentified by this NB analysis represent the same specimens misidentified by the dorso-ventral view NB analysis (M07924 ♂ and M08207 ♀). This correspondence suggests the NB analysis is focusing on aspects of morphological variation that are present in both dorso-ventral and lateral views. In contrast, only a single specimen (M12476 ♀) was misidentified by both the dorso-ventral and lateral view CVA solutions, a result that suggests the CVA result was not influenced greatly by morphological similarities and differences common to both views. Similar to the dorso-ventral view results, however, the same discrepancy exists between sex classes in terms of the number of misidentified specimens. In the case of the lateral view CVA, the preponderance of misidentified specimens were male (63%) whereas misidentified female outlines (67%) dominated the NB analysis result.

Comparing the morphologies of these two misidentified specimen subsets, we were, again, confronted by confusing inconsistencies. In terms of the CVA result, specimen M08291 ♂ lacks a canine tooth and exhibits an unusually forward placement of its lateral incisor. But the crania of other specimens that also lack canine teeth and/or have unusually placed incisors were identified correctly by the CVA discriminant function. Incorrectly identified specimens M08068 ♀, M10334 ♀, and M11108 ♂ exhibit small sagittal crests. But other specimens with equally reduced sagittal crests (e.g., M07952 ♂, M07957 ♀, M07987 ♂) were identified correctly by the trained CV-1 axis. Incorrectly identified specimens M08291 ♂ and M11417 ♂ exhibit robust orbits such that the curve of the frontal bone becomes part of the lateral outline between the snout and the braincase. Nonetheless, other specimens with equally prominent frontal bones (e.g., M07941 ♂, M07987 ♂, M08026 ♀) were identified correctly in the CVA result. Incorrectly identified specimen M11108 ♂ exhibits a distinctly rounded auditory bulla that imparts a curved aspect to its basicranial outline. But again, other specimens with equally robust auditory bullae (e.g., M08288 ♂, M12418 ♀) were identified correctly. As in the dorso-ventral analysis, it appears constraints imposed by requiring specification of a strict, geometrically linear discriminant decision surface operated to exclude a number of training-set specimens from their correct placement classes.

With regard to these lateral crania NB analysis results, commonalities are far easier to identify. Both M08207 ♀ and M08286 ♀ possess abnormally short snouts; very robust, prominent canine and lateral incisor teeth; sharply curved molar dental arcades; and highly arched premolar dental arcades when compared to typical females. Similarly, M07924 ♂ exhibits reduced canine teeth, a lower snout, more extended braincase, and more gently curved molar dental arcade than typical males. As was the case in the dorso-ventral crania NB result, it is difficult to escape the impression that this algorithm found different, and arguably more generalized, characteristics on which to separate *C. lupus* crania into sex classes relative to the CVA solution. The NB analytic result was also far more statistically significant than the CVA result with both the parametric and 1000-iteration bootstrapped versions of the log likelihood ratio test returning markedly significant *p* values of less than 0.001% (*ϕ* = 8.65).

### Image-based analyses

In order to explore how strictly image-based analyses might operate in an advanced machine-learning context, the LeNet-5 CNN architecture was used to analyze medium-resolution images of the same Israeli *C. lupus* dataset from which the foregoing boundary outlines had been extracted in both dorso-ventral and lateral views. This test examined the effect of altering the type of data collected for morphological analysis as well as the performance of this alternative, non-linear approach to finding the best between sex-class decision surface.

#### Dorso-ventral view results

**Fig. 6:**
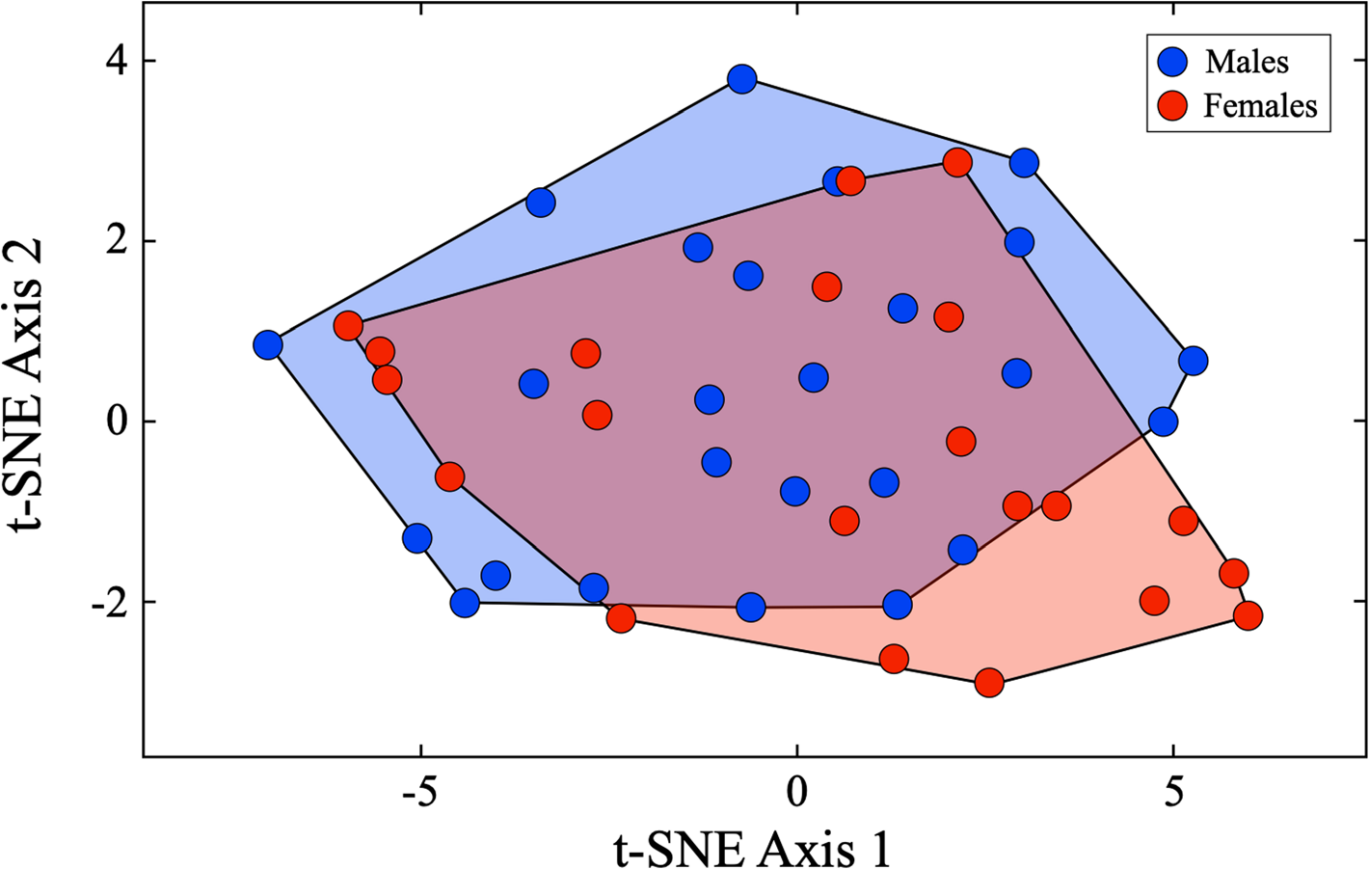
Two-dimensional feature-space plot of the Israeli *C. lupus* crania in dorso-ventral view based on the *t*-distributed stochastic neighbor embedding (*t*-SNE) algorithm. Note the lack of obvious groupings in the distribution of these coordinate-based representations of morphological variation and sample structure

Figure 6 summarizes the raw structure of the Israeli *C. lupus* images in dorso-ventral view using the *t*-distributed stochastic neighbor embedding (*t*-SNE) algorithm [44, 45] as the basis for ordination. This distance-based index was developed to facilitate reduction of high-dimensional data for the purpose of visualization in low dimensions and has been shown to produce ordinations that reveal structure at many different scales. Owing to the *t*-SNE index’s sensitivity, care must be taken when interpreting such results as it is well known that apparent clustering can result, even in cases where there is no structure (e.g., when applied to data derived artificially from a single statistical distribution, [45]). However, for these dorso-ventral view images, the 2D *t*-SNE ordination is noteworthy for its lack of obvious internal, sex-based structure. Irrespective of these broadly overlapping fields of morphological variation, there is an unanticipated amount of offset between the male and female fields with fully 39% of the total sample lying outside the region of overlap. This suggests more sex-based variation is present in the images than the outline analysis results suggested even before processing designed to maximize group differences.

**Fig. 7:**
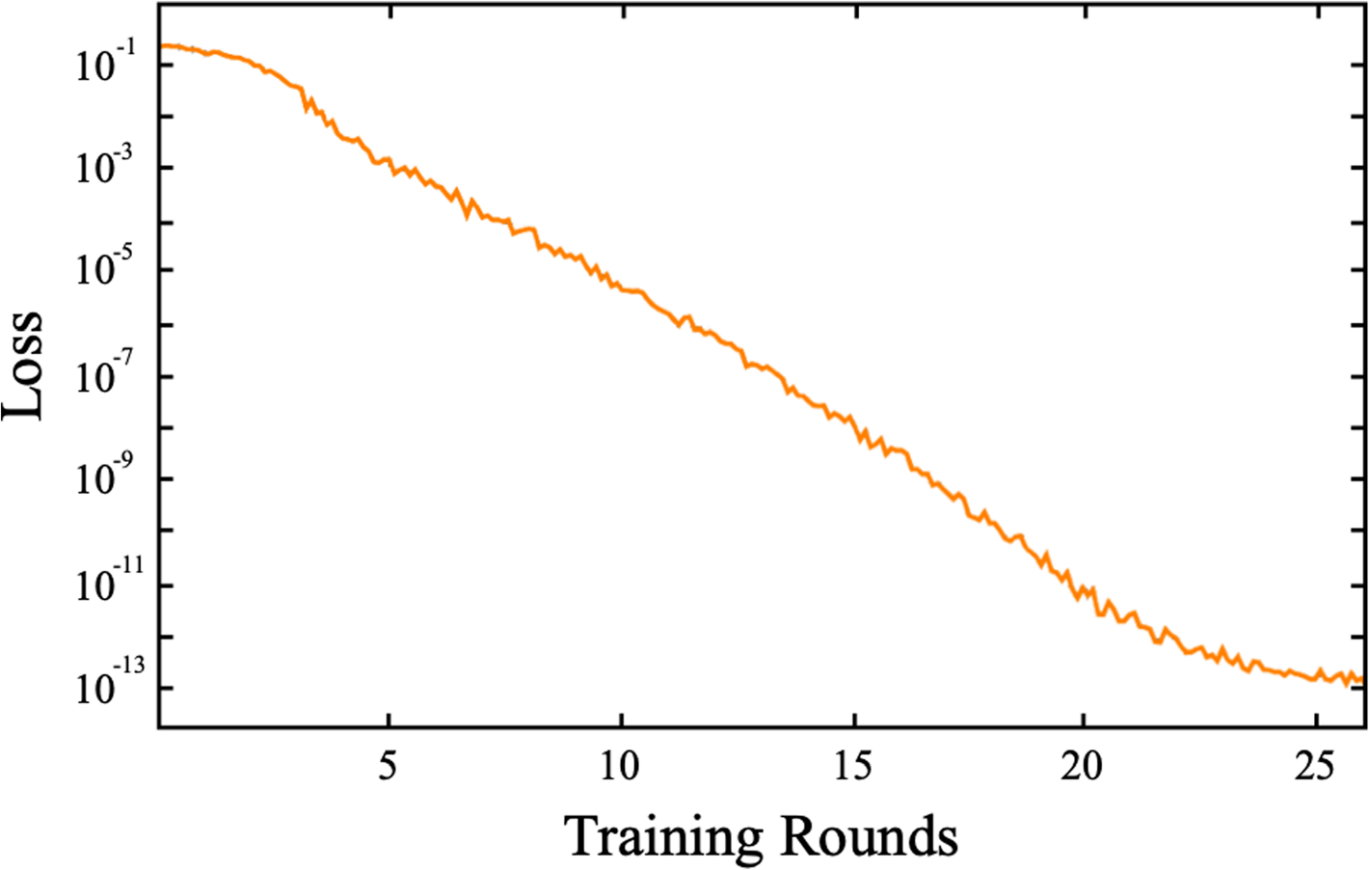
Embedded LeNet-5 training summary for the dorso-ventral crania dataset. Note the length of the training cycle, the steady improvement in the loss ratio during the whole of the training interval, and the distinct reduction in loss improvement as the point of convergence is reached. The entire training cycle took 21 s of CPU time to complete

Our embedded learning LeNet-5 CNN was initialized prior to training with random weights and then trained using 1692 pairs of images drawn randomly from the complete set of 2116 pairwise combinations of Israeli gray wolf, dorso-ventral view images. Each training round involved 702 randomly selected image pairs. Results of each training round were compared to the training-set designations and network weights adjusted via backpropagation to new values using a standard gradient-descent algorithm. Loss-rate convergence was achieved at a value of 1.72 × 10^−13^ after 26 processing rounds, by which time a total of 44,928 image pairs had been evaluated (Fig. 7).

The result of embedded LeNet-5 CNN training for these dorso-ventral view images is summarized in Table 5. Unlike the previous EFA-PCA-CVA and EFA-PCA-NB analyses of the outline morphometric data, the image-based embedded LeNet-5 system returned perfect sex-based discrimination results for the training set. The distinctiveness of the male and female morphological fields discovered by this analysis not only suggests sex-based morphological differences exist in these dorso-ventral view morphologies, but that these differences are not confined to the shape of the boundary outline. Thus, they could not be recognized by outline-based morphometric methods.

**Table 5:** Confusion matrices for analysis of *C. lupus* dorso-ventral view images using the LeNet-5 CNN architecture in an embedded learning mode. Row and column counts as in Table 1

Group	Female	Male	Total correct	Class totals	Percent correct
Female	21	–	21	21	100.00
Male	–	25	25	25	100.00
Total correct	21	25	46	46	100.00
Total estimated	21	25	46		
Percent correct	100.00	100.00	100.00		

While it might be argued that the degree of separation indicated by Table 6 is biased from an evolutionary point-of-view, owing to the fact that biologically corresponding topological (not homologous, see [42, 46]) position matchings were not employed exclusively in this comparison, the gross similarity between male and female members of this species, in terms of their cranial anatomy, along with the steps taken to standardize the sizes of images across the dataset, minimized the effect of such differences. As these same standardization procedures were applied to all images (see Additional file 3), our image data contained no artificial clues that could have been responsible for the sex discrimination. More tellingly, the fact that the embedded LeNet-5 CNN was so successful in finding very prominent sex-specific differences in a view of cranial anatomy for which no such differences had been recognized previously—either qualitatively by taxonomists or via morphometric analysis—illustrates both the power of this approach and the wealth of relevant information standard multivariate and geometric morphometric approaches to morphological analysis have been unable to access.

**Table 6:** Confusion matrices for analysis of *C. lupus* lateral view images using the LeNet-5 CNN architecture in an embedded learning mode. Row and column counts as in Table 1

Group	Female	Male	Total correct	Class totals	Percent correct
Female	21	–	21	21	100.00
Male	–	25	25	25	100.00
Total correct	21	25	46	46	100.00
Total estimated	21	25	46		
Percent correct	100.00	100.00	100.00		

Drawing on an analogy with CVA (see [47]), it also might be argued that this result is either artifactual and/or insignificant statistically insofar as interactions between the dimensionality of these image data and sample size were such that between-class differences would be found for any class-level grouping. The fact that a perfect discrimination between sexes was achieved argues against the significance of this result being tested using the standard statistical procedures employed previously. However, if this discrimination result was artifactual—the result of overfitting of the trained model to this particular dataset—the trained network’s performance should be poor when used to identify genuinely novel morphologies that had not been used to train the system.

Since the rarity of well-preserved Israeli gray wolf crania is the reason our sample size is small, splitting the dataset into training and testing sets was inadvisable. Nevertheless, a “leave-one-out” jackknife procedure was feasible. Under this protocol, the number of pseudo-analyses undertaken was equivalent to the number of specimens in the sample. This strategy has often been used to evaluate the stability of linear discriminant analysis results though, despite the fact that cross-validation is considered a viable check on post-training ML performance [48, 49], to date, the leave-one-out jackknifing has been employed much less commonly in the context of either ML analysis in general or CNN analyses in particular.

When this jackknife evaluation strategy was used to test the ability of the trained LeNet-5 CNN to identify the sexes of Israeli gray wolf crania in dorso-ventral view, a set of a posteriori identification results identical to those shown in Table 6 were obtained. Based on this performance, there can be little doubt that (1) sex-specific patterns of morphological variation are present in our sample of Israeli gray wolf crania and (2) aspects of these differences are visible in dorso-ventral view. Despite being trained on a small dataset, the embedded LeNet-5 CNN system converged on a set of interconnected-layer node weights that allowed the sex-difference signal encoded in these cranial morphologies to be identified and employed to make correct sex assignments for both the training-set morphology and novel morphologies that fall into the range of sex-based variations represented in our sample.

#### Lateral view results

**Fig. 8:**
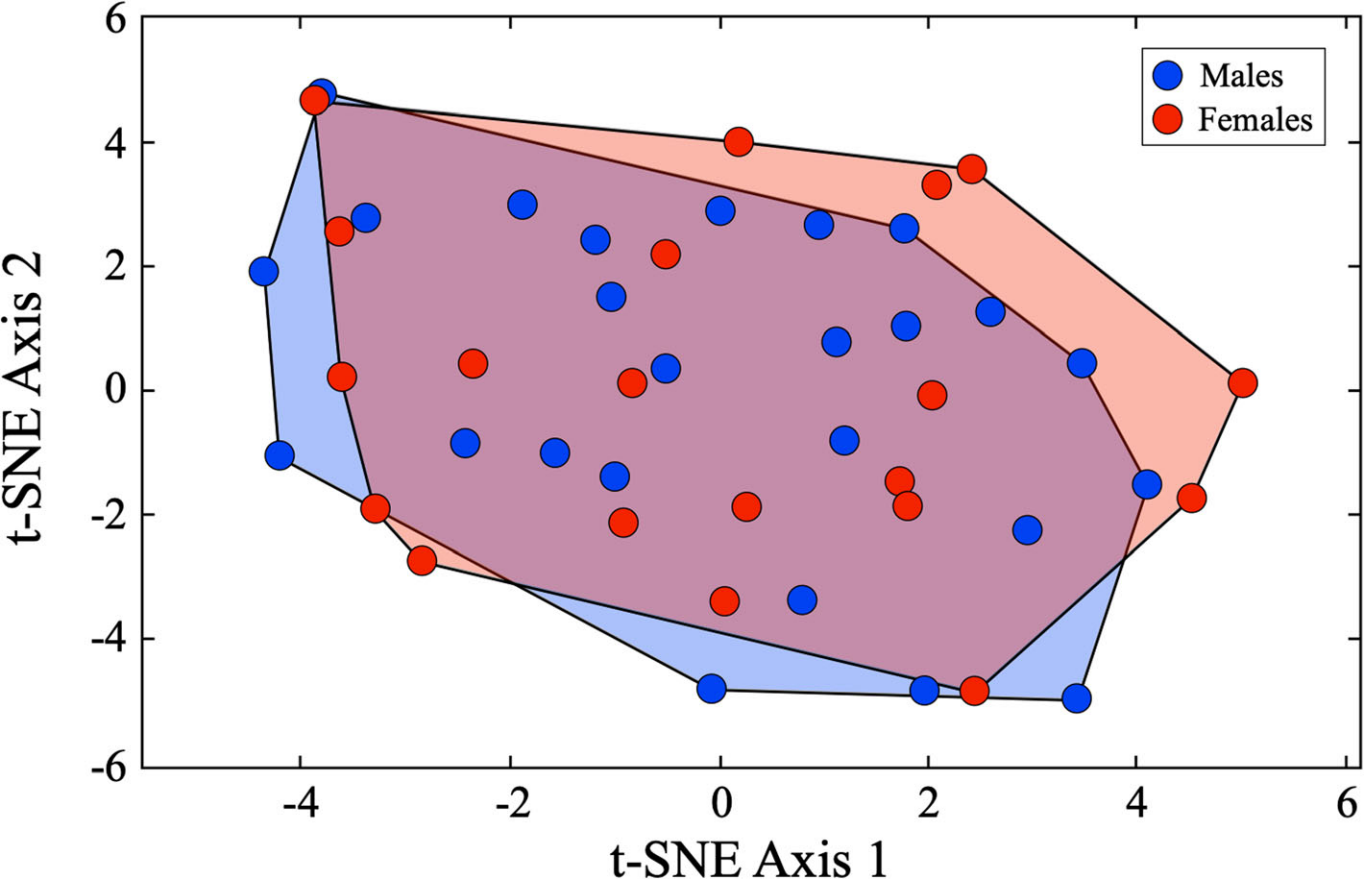
Two-dimensional feature-space plot of the Israeli *C. lupus* crania in lateral view based on the *t*-SNE algorithm. Note the lack of obvious groupings in the distribution of these coordinate-based representations of morphological variation and sample structure

Figure 8 summarizes the raw structure of the Israeli *C. lupus* images in lateral view using the *t*-SNE algorithm. As was the case for the dorso-ventral view image dataset, it is evident that no overt within-sex group clustering is present in this 2D *t*-SNE ordination space. However, unlike the previous dorso-ventral view analysis, only a small proportion of the sample (c. 15%) lies outside the common field of variation, and of these, it is the female morphologies that exhibit the strongest shape departures. Of the five female specimens that comprise this group (M07957 ♀, M08041 ♀, M08193 ♀, M09181 ♀, M12477 ♀), all exhibit prominent zygomatic processes placed on very prominent frontal brows, either high (M08041 ♀, M08193 ♀, M12477 ♀) or long (M07957 ♀, M09181 ♀) snouts, and prominent sagittal crests. Nevertheless, aspects of these characteristics are present across the female component of the sample and some crania that appear to exhibit all three (e.g., M11684 ♀) plot well within the region of sex overlap. This result suggests that, if a consistent pattern of sex-based variation exists in these data, it is probably quite subtle and possibly confined to comparatively small cranial regions.

**Fig. 9:**
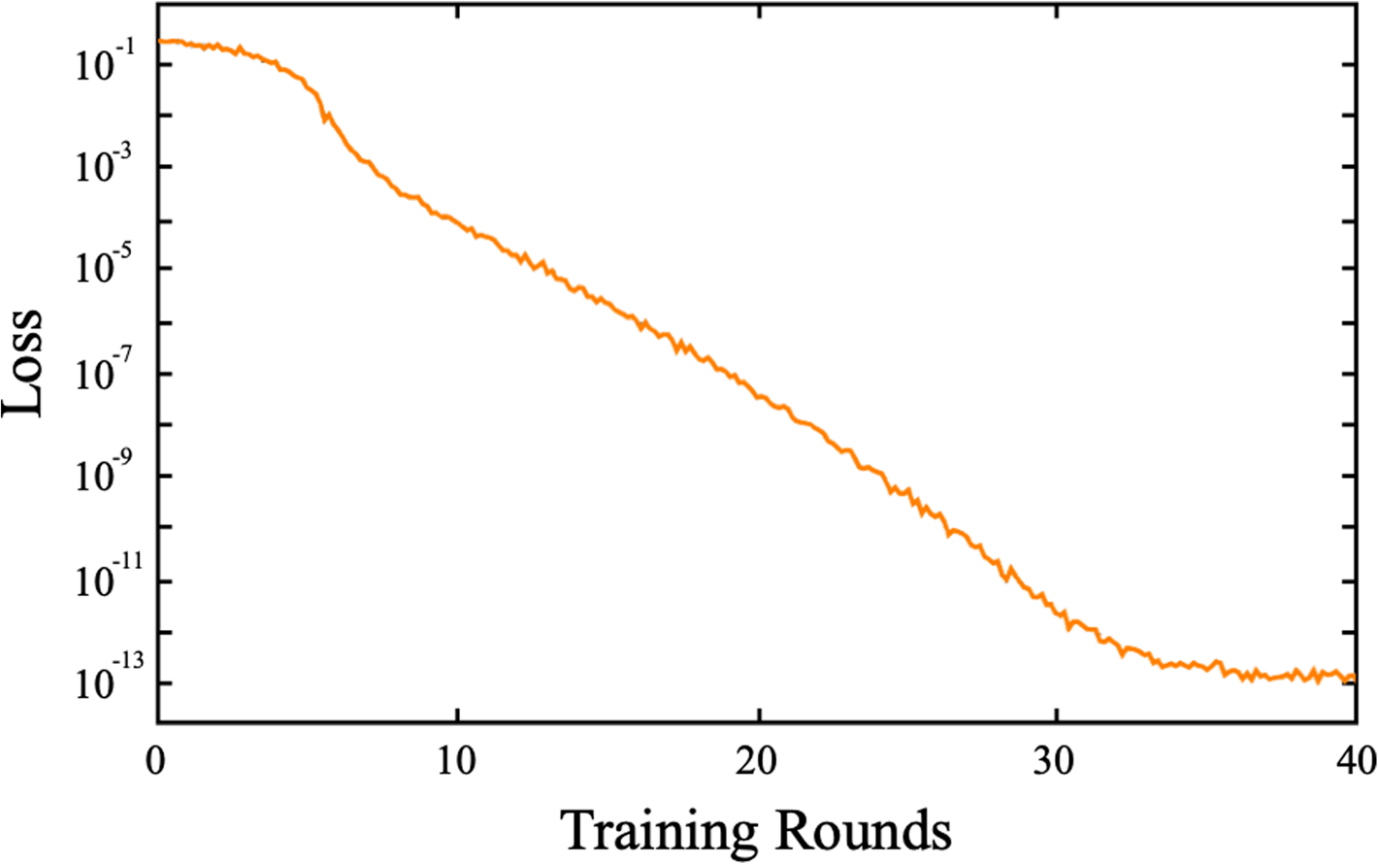
Embedded LeNet-5 training summary for the lateral crania dataset. Note the greater length of the training cycle relative to the dorso-ventral view analysis (see Fig. 7), the steady improvement in the loss ratio during the whole of the training interval, and the distinct reduction in loss improvement as the point of convergence is reached at or around 36 training rounds. The entire training cycle took 35 s of CPU time to complete

For the lateral view image set, the embedded learning LeNet-5 CNN was trained using 1692 pairs of images drawn randomly. After each training round, results were compared to the training designations and network weights adjusted via backpropagation to new values. Loss-rate convergence was achieved at a value of 1.56 × 10^−13^ after the 40 processing rounds (Fig. 9), by which time a total of 69,120 image pairs had been evaluated.

Substantial differences were evident between the CNN training histories of the dorso-ventral and lateral view training sets. In the case of the latter, training continued for more rounds, signaling the greater morphological complexity—and information richness—of the lateral view images. These differences cannot be accounted for by the different sizes of the dorso-ventral and lateral image frames because (1) the dorso-ventral image frames are actually 23% larger than the lateral image frames (see the “Materials” section) though the lateral view image takes up more of the frame than the dorso-ventral view images (see Fig. 2) and (2) all images in both analyses were interpolated down to the LeNet standard of a 28 × 28 pixel frame prior to training (see the “Methods” section). Overall, these results give the impression that the embedded LeNet-5 CNN architecture found the lateral view images more challenging to separate into sex-specific groups. This result is consistent with the preliminary indications provided by the *t*-SNE subspace plot (Fig. 8).

In some ways, this result is also expected on traditional taxonomic grounds insofar as human taxonomists would naturally focus on the morphological information recorded in the lateral view because a greater number of taxonomic characters are visible. However, this greater information content also represents a greater challenge as the complexity of the morphological patterns on display can easily overwhelm the processing capabilities of human visual systems, even those trained to an expert level. This is why taxonomists constantly search for simple morphological indicators that express, or serve as a proxy for, differences whose overall signatures may be distributed far more broadly across the morphologies in question.

Given this level of complexity and apparent morphological intergradation, what was unexpected is that the embedded LeNet-5 CNN system was also able to achieve perfect sex-separation results for these lateral view images (Table 6). Indeed, judging from the number of processing rounds necessary to achieve convergence, the dorso-ventral view dataset contained more obvious sex-specific information. This seems counterintuitive from a qualitative morphological analysis point-of-view. However, human and computer vision systems exhibit substantially different sensitivities (see [50–53]). It may well be the case that sex-specific aspects of the dorso-ventral view dataset are present and obvious, but simply difficult for human visual systems to perceive.

As with the dorso-ventral view analysis result, any suspicion that perfect sex discrimination for the lateral view training set based on the embedded LeNet-5 CNN might be a consequence of overfitting was evaluated objectively using the leave-one-out jackknife identification strategy. This analysis resulted in an error-free set of post hoc sex identifications for all specimens after their removal from the training set sequentially, prior to retraining and subsequent identification of the sequestered specimen. As a consequence of this test result, there can, once again, be little doubt that substantial, consistent, and recognizable sex-specific differences exist in the lateral views of our Israeli *C. lupus* specimens.

## Discussion

For the collection of *C. lupus* specimens analyzed here, clear and consistent evidence of sex-specific morphological shape differences has been demonstrated using two different morphological datasets, two different means of sampling morphological data, and three different data-analysis procedures. Extension of these results to an interpretation of sex-based morphological differences that may exist within larger populations of northern Israeli gray wolves—which we do not advocate at this juncture—would require acceptance of the assumption that the present sample contains a statistically adequate representation of this larger population. Such may, or may not, be the case. Only the collection of crania from additional individuals will provide an answer to this larger question. What our results do demonstrate—without question—is that compelling and statistically significant sex-specific differences exist in the morphologies exposed in the dorso-ventral and lateral views of the specimens included in our sample, which is the largest sample of Israeli gray wolf specimens assembled to date. Our results also document the superior performance of both the NB and embedded CNN machine-learning procedures over those of the linear ordination and discriminant data-analysis procedures employed routinely by geometric morphometricians, at least in terms of recovering group-level patterns of morphological distinction. There is no reason to suspect similar results would not be obtained if these procedures were applied in other situations in which group-level morphological distinctions were of interest, irrespective of taxonomic group.

In terms of size variation, our findings agree with results obtained by Mendelssohn and Yom-Tov ([54]: Table 86) using linear measurements taken on a small sample of wolf crania from the Golan Heights and northern Israel. Though the expected differences between sexes are apparent in the sex-specific measurement averages and distribution modes, there is obviously great overlap in cranial size ranges between the sexes in our sample.

Our results may seem at odds with some other findings. For example, Milenković et al. [16] reported sex-based size variation in cranial assessments of a small gray wolf sample. However, their test was applied to a pooled sample of Carpathian and Dinaric-Balkan wolves, and so cannot be used to infer the states of individual populations. In addition, these authors’ use of a pooled sample increased the sample size used for the body-size differentiation test and so would be expected to return a significant difference for a smaller size differential. In another example, Hillis and Mallory [23] reported sex-based dimorphism in the mass of various gray wolf body organs (e.g., heart, liver, kidney), some long-bone lengths (e.g., ulna radius, tibia-fibula, femur, but not humerus), and total body mass. As our investigation is focused only on the cranium, our results do not disagree with theirs [23], especially since that investigation found non-significant sex-based differences in total body length and contour length.

Sex-mediated dimorphism can occur either in the context of size or shape variation. Rensch’s rule [55–58] states that, in groups of related species, sexual size dimorphism is more pronounced in species where males are the larger size. Since the gray wolf is the largest extant member of the Canidae [27], it is reasonable to expect this species to exhibit pronounced patterns of size dimorphism. Moreover, the fact that gray wolves exhibit polygyny and have sexually differentiated behavioral roles that reward male size differentials when defending territory or competing for mates makes this species a classic mammalian example of Rensch’s rule [55, 56].

The standard allometric model [59–64] assumes that differences in shape arise as a result of selection on generalized body size. In one of the most widely cited textbook applications of this model, Gould [65] argued that the antlers of the Irish Elk (*Megaloceros giganteus*) attained their outstanding size not as a result of direct adaptive selection on antler size itself for some specific utility (e.g., sexual display), but as a result of indirect selection for body size through allometric linkage (see [66] for an alternative physiological model that still involves allometric scaling relations). Under this model, sexual dimorphism among adults might arise as a result of the differential expression of allometric scaling relations that arise from one sex attaining a greater size than the other. Countless allometric investigations have applied this model to morphometric data collected from various species. However, a lesser-discussed alternative also exists in which each sex exhibits ontogenetic differences, such that observed morphological distinctions represent the unfolding of inherently different sex-specific trajectories through the size-shape-time space (see [67, 68] for alternative discussions of this space).

These two models make different predictions with regard to the modes of shape variation that should be found in individuals of different sexes. Owing to our failure to find sex-based size differences in our Israeli *C. lupus* sample despite the existence of clear and consistent shape differences between male and female crania, our results are more consistent—at least superficially—with this latter allometric model. We acknowledge that, owing to our small sample size and lack of direct ontogenetic data, confirmation of this working hypothesis must await focused ontogenetic investigation and the collection of additional specimens. Nevertheless, the results we have documented are plainly inconsistent with the standard allometric model. If our suspicions are correct, gray wolves might supply an important cautionary tale with regard to the classic mistake of interpreting patterns of static allometry as if they were patterns of ontogenetic allometry.

In their study of sexual dimorphism in *Urocyon*, Schutz et al. [15] failed to find pronounced patterns of size or shape cranial dimorphism. Instead, these authors were able to demonstrate sexually dimorphic pelvic shape differentiation in the extant species they investigated, results that are, again, plainly at odds with the prediction of Rensch’s rule. These authors proposed that sexually mediated differences in factors such as offspring size and locomotor mode may play a greater role in developing and maintaining intraspecific patterns of sexual dimorphism than “whole-body” allometric affects associated with dimorphism in body size. Alternatively, in a comprehensive study of 5300 bird species, Dale et al. [58] found that Rensch’s rule was driven primarily by correlated evolutionary change in females to generalized directional selection on males even after normalization for a variety of potential generating factors (e.g., polygyny/polyandry, plumage dichromatism, fecundity, intersexual resource competition).

While logic would suggest statements made by Dale et al. [58] and Schutz et al. [15] regarding the factors responsible for Rensch’s rule, as well as the implications of differential shape dimorphism, cannot both be correct, this assumes that the results produced by these (and other) investigations are comparable and document both fair and representative summaries of generalized sexually dimorphic patterns, not only in terms of the specific morphological structures investigated, but across the entire phenotype. Indeed, this discrepancy raises the more fundamental issue of whether results obtained from *any* set of investigations of specific morphological structures can be taken as valid proxy representations of the morphological states of whole populations or species.

The results obtained by Dale et al. [58] were due entirely to comparisons of male and female avian wing lengths which were demonstrated to have a reasonably high coefficients of determination with body mass (*r*^*2*^ = 0.89) and, as a consequence, assumed to be valid proxies for body size. Owing to the employment of a single localized variable to represent whole-body size, these investigators were unable to address the question of the spatial variability in the expression of sex-based shape differences or to assess the ontogenetic, evolutionary, ecological, or behavioral implications thereof. In effect, their research design equated sexual dimorphism with sex-specific size differences. While it may be true that a single linear distance variable might provide an adequate proxy for generalized size variation across a taxon’s body, the empirical findings of most morphometricians over the last 50 years suggest this is rarely, if ever, the case. Consequently, it has long been considered best practice in allometric studies to employ measurements taken from different regions of the body to construct synthetic, whole-body estimates of both size and shape.

In the case of the Schutz et al. [15] investigation, body size was estimated using centroid size, which is the multidimensional estimator that, by definition, is uncorrelated with all measures of shape [69]. Here, shape variation was tied to determination of the partial Procrustes distances between paired landmark configurations in which the extraneous variables pertaining to (centroid) size, position, and rotation relative to a reference configuration (= the mean landmark configuration for the sample) had been removed from consideration [69–72]. This clear separation of size variation from shape variation allowed Schutz et al. [15] to consider both the size-based and shape-based aspects of sexual dimorphism as distinct phenomena. However, both these aspects of cranial and pelvic variation were tied ultimately to the cranial and pelvic landmark locations used to represent these structures which were targeted a priori, before any empirical results had been generated.

In both these cases, representation of the complex three-dimensional morphologies of bodies and/or body structures via sparse sets of linear distances or two-dimensional landmark locations (= in effect, two linear distances) inevitably excluded much information relevant to the documentation of sexual dimorphism—or any relevant morphological contrast. By itself, this does not represent an insurmountable problem so long as the goals of the analysis are tied to the specific structures, and positions on these structures, that have been sampled. But the stated goals of both these investigations were to discover whether sex-specific dimorphic patterns of morphological variation were present in bodies and body structure of the taxa in question as a whole.

“The objective of this study is thus to test two critical predictions of the sexual selection hypothesis using a close to complete representation of taxa (subfamilies) within an entire class (Aves). First, prediction 1: groups of related species in which sexual selection on size is stronger in males should demonstrate positive allometry, independently of confounding factors such as the overall degree and range of size dimorphism. Second, prediction 2: groups of related species in which sexual selection on size is stronger in females should demonstrate negative allometry” ([58], p. 2972)

“We examined sexual size and shape dimorphism in the cranium and os coxae (one of two paired bones comprising the pelvis) because dimorphism in these body regions is potentially affected by differences in sexual selection.” ([15], p. 340).

Clearly, the a priori selection of particular aspects of the bodies or body structures becomes problematic in such cases insofar as patterns of variation inherent in a small number of particular features may not constitute an adequate representation of the body, or the structure, as a whole. This danger is particularly obvious and pertinent if negative results are generated as they were in aspects of both these investigations.

At this point, it must again be stressed that no challenge is being offered to the results obtained by Dale et al. [58] or Schutz et al. [15], only to the extended interpretations offered by these authors which could be taken to imply their results can, or should, be considered generalized assessments of the species morphologies under investigation and/or to cover aspects of avian and mammal morphology that were, in fact, not sampled. In other words, there is an important difference between stating that a species is (or is not) sexually dimorphic because a few characteristics have been assessed and found to have (or not) sex-specific expressions at comparable life-history stages, and stating such dimorphism extends to all aspects of the morphology whose expression thereof might be either obvious or subtle owing to allometric scaling relations or sex-specific developmental pathways. At the very least, it is incumbent upon investigators to specify the level of phenotypic generality to which their interpretations apply.

The reason why generations of biologists, morphologists, and morphometricians (including the senior author of this article) have been trapped into this pattern of inappropriately generalizing from results obtained as a consequence of highly specific analyses of aspects of organismal morphology is that, for (literally) centuries, there has been no alternative. The quantitative analysis of morphology and morphological variation required the quantification of particular sets of observations, first as sets of linear distances between corresponding landmark locations and later as configurations of landmark or semilandmark locations themselves (see [31] for a review). This requirement forced investigators to assume that the small sets of quantifiable distances or coordinate points which could be located on all specimens within a sample were sufficient to represent the general geometries of the forms and/or structures in question. Moreover, the ability to quantify patterns of morphological variation, even in minor and somewhat idiosyncratic aspects of the morphology, was generally held to be superior to more holistic qualitative “analyses” that relied on the visual inspection of aspects of the morphologies in question, performed by highly trained and experienced morphologists, but which were not susceptible to probabilistic hypothesis testing. With the advent of widespread image digitization, the increase in the power of even modestly priced computer platforms, and most importantly the development of more generalized computer vision-based morphological analysis algorithms, an effective synthesis between the qualitative Gestalt and quantitative morphometric approaches to morphological analysis has now been achieved [30–37, 73]. Our results, along with others published recently (e.g., [32, 34–37]), illustrate how new and generalized approaches to morphological analyses make it possible to assess patterns of variation in any aspect of an organism’s morphology, in any sample for which images can be obtained, irrespective of complexity and without forcing the analyst to make a priori decisions regarding which aspects of the morphologies in question to measure, compare, model, or interpret. As our analysis of Israeli *C. lupus* crania has demonstrated, investigations of both size and shape variation that support quantitative comparisons, the ordination of specimens in mathematical spaces, and statistical hypothesis testing can be conducted directly from 2D digital images on a pixel-by-basis. By direct extension, they can also be performed on 3D digital scans on a voxel-by-voxel basis. These assessments can be undertaken in a manner fully compatible with most geometric morphometric data-analysis conventions [30, 32] or by using newer, more mathematically sophisticated approaches to the analysis of patterns in morphological variation [32, 34–37]. The former approach is especially convenient for conducting exploratory analyses whose primary purpose is to identify regions of particularly prominent patterns of morphological variation, either within groups or between groups, whereas the power of the latter resides in the use of non-linear methods to achieve detailed and robust evaluations of generalized group differences.

One common assumption often made about machine-learning algorithms is that they recognize the same between-group differences as linear discriminant algorithms but, in some way, do so more accurately or more efficiently. This perception is understandable because it is difficult to imagine how classes might differ in ways other than those that can be seen or otherwise assessed visually (e.g., as class-demarcated distributions of points in a variable space, as models of morphological differences based on discriminant spaces). In this sense, the operation of machine-learning algorithms are genuinely difficult to comprehend since, owing to their complex mathematical nature, it has been difficult to devise procedures that enable researchers to identify which specific aspects of morphology these algorithms are recognizing as class-defining differences in the manner (say) of Figs. 3 and 5. Nonetheless, the close comparison of our CVA, NB, and by extension CNN results suggests they are keying on very different aspects of the morphologies under consideration.

Despite the small number of specimens comprising our sample, our results also offer substantial evidence that northern Israeli *C. lupus* populations may be characterized by spatially varied amounts of sex-based shape dimorphism that are likely related to the different behavioral and social roles males and females perform in their packs. The shape differences recorded by our results are subtle, and it is not surprising that they have been missed by previous investigators. However, they are consistent, interpretable, statistically significant, and subject to further testing with larger samples.

Specifically, our results extend the findings of Gittleman and Van Valkenburgh [13] insofar as they point to additional features of the cranium which suggest that jaw-muscle size, and the consequences of jaw-muscle activity, may play a large role in determining the patterns of sex-based dimorphism in *C. lupus* populations. Our results are also consistent with their hypothesis that breeding system can serve to enhance the patterns of cranial dimorphism in this species (though this consistency needs to be explored further) as well as demonstrating that aspects of the cranium other than the length of the canine teeth may be involved in sexually dimorphic shape variation.

Additionally, our findings are consistent with those of Schutz et al. [15] insofar as they confirm these authors’ suspicions that sexually dimorphic patterns of variation, mediated by alternative social roles, appear to be present in the cranium of large carnivore species as well as in aspects of the pelves (though we have been unable to find any published reports of quantitative, *C. lupus* pelvic-shape investigations). In a recent study using morphological indices of robusticity, Morris and Brandt [18] reported significant sexual dimorphic differences in wolf post-cranial forelimb and hindlimb elements as well as crania. These authors attributed the marked variation they observed in these characters to selection in males for improved prey-capture performance, a result that reiterates the findings of Hillis and Mallory [23] based on visceral and adipose parameters in gray wolves. We suspect this explanation probably accounts for the cranial shape differences we report here for Israeli wolves as well. Our findings also agree with those of Milenković et al. [16] insofar as they underscore the importance of the zygomatic arch as a locus of sex-specific shape differentiation in *C. lupus* crania while offering considerably more detail about the geometric character and distinctiveness of these differences than any linear distance or landmark-based assessment has to date.

As we have noted above, one potential criticism of the image-based methods we have employed is that pixel arrays lack a simple and straightforward procedure for tracking the migration of corresponding biological structures that many geometric morphometricians find attractive in landmark-based studies. This criticism is both true and false. In the case of simple diagrams such as triangles, there is no difference between the representation of size and/or shape differences as sets of Cartesian coordinates or as pixel locations. The Cartesian representation of the form is explicit in its landmark location values whereas, in the latter, it is implicit in the configuration of landmarks within a pixel frame, but in no way is it less realized or informative. Leaving aside the theoretically possible, but practically rare, case in which unique pixel-landmark vertices migrate to positions that correspond to the precise locations of alternative pixel-landmark vertices (e.g., an equilateral triangle rotated through 120° of arc about its centroid), these two representational conventions yield very similar results irrespective of the methods chosen to analyze them. Nonetheless, in complex images, the “distance” between images may be thought of as the sum of differences between the pixel brightness/color values across all locations in the pixel frame. These distances correspond in principle to the distances representing the displacement of corresponding pixel locations, but will tend to depart from one another as images increase in complexity or whenever images of forms that differ radically in their morphological structures are included in the same analysis. But these same representational constraints also apply to the landmark-based analysis where they can conspire to reduce the spatial resolution of landmark-based analyses owing to difficulties in finding a sufficient number of comparable landmark locations across all specimens in the sample. The ability of CNN systems to identify similar patterns of morphological variation irrespective of where they occur in an image frame and/or specimen orientation circumvents the entire issue of simplistic, point-by-point, image correspondences in the context of group-discrimination problems.

Landmark datasets, by their very nature, will always be restricted to the characterization of only a small subset of the morphological features present in complex organic structures, whereas image-pixel datasets are, by their very nature, sensitive to differences among all features present in the image frame that can be represented by pixel brightness/color values. The comprehensiveness of this representation is the primary point of using image-pixel data to quantify patterns of morphological variation. In terms of achieving class characterizations, and so facilitating reliable identifications (automated or otherwise), fine distinctions between what is being characterized and how these characterizations relate to the underlying biology are, in many senses, beside-the-point so long as the resulting identifications are correct, consistent, generalizable, and able to be achieved rapidly with little time taken up collecting the data on which the identifications are based. In such instances, the direct analysis of digital images presents many clear, compelling, and unambiguous advantages.

Irrespective of such considerations, these alternative approaches to the analysis of morphological data should not be seen as competitors. Both can be employed in a reciprocal manner (e.g., regions of high shape variation identified using image-pixel analysis can be probed in greater detail subsequently using a landmark, boundary outline semilandmark, or combined analysis) or used in isolation—whichever is most appropriate for the investigation at hand. In the same sense, machine-learning data-analysis techniques should not be seen as competitors to more traditional eigenvector-based approaches to multivariate data analysis. Either may be applied to the same data, as we have shown in our comparative analyses of the *C. lupus* outline data using the CVA and the NB algorithms. In our case, the machine-learning approach did a better job in finding consistent sex-mediated differences in our *C. lupus* data. But there was a (provisional) cost involved in terms of the manner in which the NB results could be visualized and interpreted. Nevertheless, it is the potential that such combined image/landmark-semilandmark strategies have for supporting morphological investigations, in addition to their “stand-alone” power, that we are most interested in highlighting here.

Although our investigation was conceived originally as an extension of geometric morphometrics that addresses some of the issues that arise routinely in the context of morphological research, the methods we have described also have clear implications for the emerging field of phenomics [36, 74–78]. Gene expression studies are beginning to unravel the complex interactions between genetic variation, environmental variation, and phenotypic variation [79, 80]. However, owing to the existence of complex pleiotropic effects, a wide range of morphologies across organismal bodies must be scanned in order to confirm causative correlations with patterns of genetic variation. Image pixel-based methods of morphological analysis are well-suited for phenomic studies, far more so than the selection of a few measurements or landmark locations in terms of achieving the comprehensive coverage necessary to facilitate such investigations. Similarly, the ease and rapidity with which data pertaining to the characterization of complex morphologies can be collected facilitate the assembly of large phenomic datasets. In this way, an image pixel-based approach to phenomic analysis can operationalize both the extensive and intensive phenotyping described by Houlé et al. [74]. The analysis of image-pixel data can also benefit directly from the machine-learning and automated-identification technologies that many believe are set to revolutionize much of biological research [81–83] as well as everyday life in the coming decades.

Finally, at their most general level, the results we have achieved, along with those of other recent image-based investigations (e.g., [36, 83]), suggest that morphometrics now has the ability to transcend its roots in biological morphology and address important questions in fields that have not been regarded traditionally as posing morphology-based problems. These include research programs in other areas of the biological sciences (e.g., [84]), the medical sciences (e.g., [85], the physical sciences (e.g., [86]), and possibly even in the social sciences and humanities (e.g., [87]).

Geometry is a fundamental aspect of the world in which we live. Owing to our own evolutionary history, we have an affinity for conceptualizing the patterns we observe in geometric terms. The tools of shape theory—which resulted in the development of geometric morphometrics—have provided the scientific community with a set of data-analysis procedures of truly unlimited potential. In order to push the morphometrics revolution forward in the twenty-first century, however, morphometricians need to understand the generalized nature of the tools they possess; incorporate new, non-linear data-analysis tools into their kit; and appreciate the geometric dimensions of interesting questions that exist in research fields far removed from morphometrics’ traditional “home turf” in systematic and evolutionary biology. By expanding the range of data that can be considered “morphometric,” the data-analysis tools available for the investigation of these data, and the scope of scientific problems that can be addressed by morphometric methods, morphometricians can not only make important contributions in areas far removed from their field’s local neighborhood, but provide important assistance in reconceptualizing both the problems of, and solutions to, issues across the broad scope of scientific research.

## Conclusions

This investigation has demonstrated the existence of statistically significant sex-specific cranial-shape dimorphism in a small sample drawn from the *C. lupus* populations of northern Israel and the Golan Heights. The fact that independent analyses involving alternative representations of cranial morphology and three different data-analysis procedures all resulted in the identification of sexually dimorphic differences provides the consistency expected from biologically significant aspects of variation as opposed to random statistical artifacts. In addition, the fact that all analytical indices demonstrated that male morphologies tended to be more rugged in terms of their typical shapes relative to females is consistent with the results of previous quantitative analyses of this species [13, 16, 23].

Although these contrasts are unquestionably present in the data derived from our relatively small wolf sample housed in Israeli museum collections, use of a non-parametric bootstrap approach to statistical hypothesis testing clarified their significance. Additionally, use of a relatively new machine-learning image-analysis strategy—the embedded CNN—resulted in a vast improvement in the focus and sensitivity of our analysis thereby providing a viable strategy through which the data that reside in small samples might be accessed for scientific study.

The extension of results obtained from this sample to its parent population is uncertain at present, especially since our sample, like most museum collections, was obtained in a manner that cannot be considered random. Nonetheless, the fact that differences as subtle as the ones we have documented would be judged significant if they were maintained in a larger sample is an important finding that, at the very least, suggests similar modes of cranial dimorphism may be present, not only in the northern Israeli gray wolf (*C. lupus*) populations, but perhaps in other populations of this species as well. It is often the case that regional samples of ecologically important species are small. This investigation provides an important illustration of how much can be done with such small samples, both in terms of the exploration of patterns of morphological variation and the statistical testing of morphological hypotheses. Methodologically, it also provides an outstanding example of how newer, more mathematically sophisticated, approaches to the analysis of morphological data can be used to extend, augment, refine, and in some instances replace those that are considered “state-of-the-art” currently. The results of our investigation also demonstrate how much useful information resides in morphological data that is not being accessed by geometric morphometric methods—much less Gestalt approaches to morphological analyses—and how much the continuing development of new, computation-intensive methods of data analysis will extend and improve our ability to access and understand this information in biological research contexts.

Generalized and multifaceted approaches to morphological data analysis offer a wide range of advantages of interest to morphologists in general and vertebrate biologists/paleontologists in particular, including the provision of baseline dataset for comparing size and shape variation in both wild and domestic populations and provision of a generalized framework for the identification and testing of correlates between specific morphological modifications and a variety of putative biological, ecological, and environmental drivers. Owing to the need for patterns to be identified and used in virtually all aspects of scientific research, in commercial development, and indeed in human life, we predict the role of morphometric analysis will continue to grow and diversify via the collection of new types of data and use of new data-analysis approaches.

## Methods

### Materials

The sample under consideration was composed of a set of gray wolf (*C. lupus*) crania held in the comparative zoological collections of the Hebrew University of Jerusalem and the Zoological Museum of Tel Aviv University (Additional file 1). Forty-six crania, 25 males and 21 females, were included. While this represents quite a small sample, it accurately reflects a common “real world” situation with regard to the number of specimens representing local populations that often reside in museum collections (see also [16]). All specimens exhibited full sets of permanent maxillary and mandibular teeth or tooth sockets, tooth wear that ranged from slight on the tooth tips to extensive on all cusps, and/or closed basioccipital-basisphenoid and basisphenoid-presphenoid joins. Accordingly, all specimens were considered sexually mature adults. In the absence of precise age data, the minimum age of our sample was estimated at ca. 4 years based on comparison with a Minnesota gray wolf population that included known-age wolves and others whose ages were known to within 1 year [88].

These specimens were collected between 1992 and 2010 from different regions of the Mediterranean phytogeographic zone: the Upper Jordan Valley, the Upper Galilee, and the Golan Heights. Israeli wolf populations follow a clinal north-south size gradient (incl. a pelage color gradient) with the heaviest and largest individuals occurring in the Golan Heights compared to those in the southern desert region. Mendelssohn and Yom-Tov [54] noted that specimens from the Galilee fell between the Golan and southern wolves in terms of size and pelage attributes. Skull length has been reported to range from 8.7 to 13.4% larger in Golan male and female wolves, respectively, relative to their southern Israeli counterparts [88]. Male Golan wolves have also been reported to be slightly heavier than females (with average weights of 29.6 kg and 25.4 kg, respectively) and larger in all body size measurements (see [54]: Table 1). These differences reflect these populations’ mtDNA structure insofar as Gray et al. [89], Wayne et al. [90], and Kahila et al. [91] all found Golan wolves to be more closely related to European gray wolves than to southern Israeli wolves.

According to Reichman and Salz [92], the size of the Golan gray wolf population was ca. 80–100 adults in 2005, but is increasing despite high pup mortality. Golan wolf packs are composed of a dominant pair (α-male, α-female) with other members typically ranging from two to seven individuals, excluding pups. The average Golan wolf home range is 46 km^2^ with an average foraging area of 9.1 km^2^. Golan wolf pack sizes change when foraging changes by season. Individuals commonly forage alone in summer and in pack groups during the winter. Emigration from the Golan to neighboring areas is low (ca. 9%), and dispersal distances are relatively short (15.6 km on average). In 1999, Mendelssohn and Yom-Tov [54] noted that the Galilee wolf population was extinct. However, since the 1990s, a wolf population has become established in the Upper Galilee which, by 2012, had grown to ca. 70–100 individuals [93].

The Upper Jordan Valley sample is composed of individuals from the Hula region. It is possible some individuals may have originated in the Golan or Upper Galilee, but no specific information is available as to their origin. Prior to the draining of the Lake Hula in the 1950s, wolves were counted as one of the wild species inhabiting this region [94].

Based on all the factors that have acted to restrict the population sizes of wolves from this region, it is clear that studies of its morphological variation patterns will likely always be restricted to small sample sizes. Regardless, our sample is representative of all the documented skeletal material available from this region.

All images used in this investigation were created from oriented 3D scans of *C. lupus* crania that were manipulated via software into standardized dorso-ventral and right lateral (= buccal) orientations, respectively. Orthographic projections of these 3D scans were then captured as 2D digital images using screen-capture software. Use of 3D scans was optimal for our purpose because many 3D reconstruction applications allow a high degree of control over specimen orientation, perspective adjustment, and illumination (see the “Methods” section). In addition, representation of the scanned morphology via a series of filled, semilandmark-defined polygons captures a high degree of surface detail while ensuring the morphological signal is not complicated by extraneous factors such as staining and/or variations in overall specimen color due to age, collection locality, preparation, storage history, etc.

### Methods

**Fig. 10:**
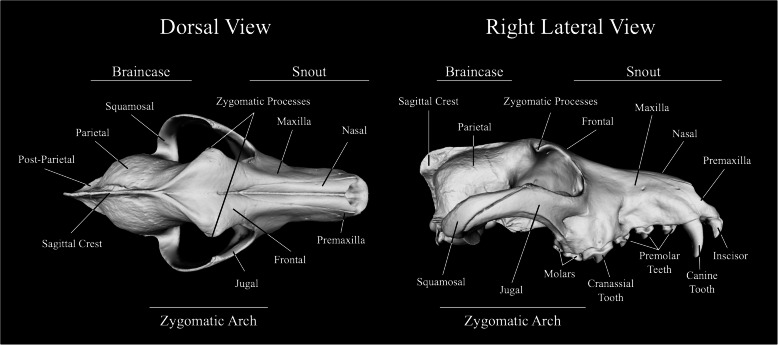
Gray wolf (*C. lupus*) cranial skeletal anatomy as represented by 2D images created from 3D scans. Specimen figured is M8193 ♀ from the collections of the National Natural History Collections, Hebrew University, Jerusalem, Israel

The mammalian cranium’s lateral aspect is illustrated most commonly in taxonomic publications as this exposes many features of the snout, braincase, and dental arcade simultaneously (Fig. 10). However, in this view, the zygomatic arch obscures all characteristics of the cranial morphology that lie behind this structure. In addition, the lateral vault, relative thickness and curvature of the zygomatic arch, degree of inflation and form of the braincase, width of the snout, and upper surface of the zygomatic arch are all difficult or impossible to assess accurately in lateral view (see [16] for an additional example). In order for these aspects of cranial morphology to be included in our analysis, both right lateral and dorso-ventral views of the same set of *C. lupus* crania were subjected to morphometric analysis. The cranium’s ventral (= bottom) view was not used as, in many museum specimens, teeth are frequently missing or broken as are the thin bones of the palate and basicranium.

While the data-collection methods we have applied are tolerant of a substantial amount of specimen damage, severely compromised specimens will, inevitably, be represented as form/shape outliers due solely to the amount of non-biological variation they record. Five crania in our sample had sustained substantial damage, exhibiting incomplete zygomatic arches (M07940 ♀, M07953 ♂, M07987 ♂, M08039 ♂) or braincase walls (M08200 ♂, see Supplementary File 1). After a preliminary assessment of left-right asymmetry in complete and well-preserved specimens failed to find significant shape differences between sides, whole-crania forms for the dorso-ventral views of these specimens were estimated by bisecting their images along the cranial mid-line and reflecting the undamaged half-crania across the axis of symmetry to create pseudo-complete forms.

This decision was not made lightly as we are aware of the many complications it induces and the advice against this procedure’s use (e.g., [24, 25]). Nevertheless, our preliminary analysis indicated these pseudo-complete specimens occupy positions in various multivariate ordination spaces consistent with the positions that would be expected if only half-cranial images were used. More importantly, exclusion of these damaged crania would further reduce the sample’s size while use of half-crania would inevitably have necessitated the reflection of some of the half-crania to opposite orientations as well as complicating image registration [24, 25]. Since the damage-correction procedure adopted here involved only a small number of individuals, preserved all of the valid information content of the entire sample, and since the preliminary results obtained failed to provide any indication that the gross morphologies of these “reconstructed” specimens projected to unusual positions within the various mathematical ordination spaces, we believe our decision was prudent and justified in the context of this particular analysis. However, we hasten to add that we do not advocate use of this procedure as a routine data-processing step. Readers may track these specimens in the discussion below as their registration numbers will be marked with a “♰” symbol.

Prior to further processing, all images were standardized for illumination direction and intensity. The 2D image datasets so produced were processed by placing the images on a uniform black background, standardizing the size and the aspect ratios of their digital image frames, reducing the size of this frame to 500 × 279 (dorso-ventral analysis) or 500 × 214 (lateral analysis) pixel matrices, standardizing the areal size of the images contained within the frame (to eliminate size variation), converting the color (RGB) pixel values to an 8-bit grayscale format, standardizing each image’s exposure so that each conformed to a consistent average pixel brightness value, and aligning each specimen’s image to the mean outline for each image set in a two-step procedure: (1) alignment of all images to a reference image and (2) a second re-aligned of all images (including the first) to the mean image of the first alignment set. Together, these image-based operations corrected for the traditional Procrustes parameters of translation, rotation, and scaling [71, 72].

Boundary outline semilandmark data were collected from the original (non-size standardized) images. A common resolution of 300 semilandmark points was used to represent the forms of the cranial outlines in both lateral and dorso-ventral views. In order to ensure comparability with the image dataset, the cranial forms in both dorso-ventral and lateral views were represented as complete outlines despite the fact that, in a number of cases, teeth were either missing or had been subject to extensive (presumably age-related) wear (see Supplementary File 1). This might be regarded as an obvious and somewhat artificial source of variation that could be eliminated prior to analysis either through image editing or via removal of these specimens. However, since a primary purpose of our investigation is to compare results that might be achieved via the application of different data-collection and data-analysis strategies, the retention of such “real-world” complications was deemed both necessary and instructive.

Centroid sizes were obtained for both lateral and dorso-ventral outline datasets and tested for structured male-female differences using the parametric two-sample *t* test and the non-parametric Mann-Whitney (M-W) test. Semilandmark data were then prepared for shape analysis by submitting them to EFA [95]. For both dorso-ventral and lateral datasets, cranial outline shapes were transformed into 45 sets of the four elliptical Fourier harmonic coefficients, the number of harmonics required to reconstruct each cranial outline to a minimum of 95% of its original shape. These data were combined into a 177-variable data matrix since, for each outline, three terms of the first elliptical Fourier harmonic are constants. Numerical data matrices representing lateral and dorso-ventral cranial variation were processed via a covariance-based PCA to repackage the observed shape variation into the smallest number of independent variables consistent with preservation of 95% of the shape structure present in the original sample.

While a PCA transform is useful for repackaging the content of a covariance matrix, PCA is not able to represent distinctions between a priori-determined groups of specimens accurately unless those distinctions happen to coincide with the major aspects of pooled-sample variation [30, 32]. In order to ensure accuracy in the characterization of sex-based differences, a secondary CVA was performed on a dataset composed of the projected scores of each set of outline EFA coefficients on each of the retained PC eigenvectors to create a linear discriminant space that maximized between-group separation relative to within-group dispersion (see [49, 50]). A mathematically equivalent procedure has been employed recently by ecologists where it has been referred to as “canonical analysis of principal coordinates” [96, 97].

Traditionally, the performance of discriminant function(s) is tested by using them to place members of a known validation set into classes via applications of the discriminant functions. Class affinity estimates are usually made based on proximity of the projected position of each observation set—in this case outline shape—to the position of the class centroids along the discriminant axes, and the results of this exercise summarized in a “confusion matrix.” Since our sample size was limited, an alternative test of discriminant axis performance was implemented via the leave-one-out jackknifed or cross-validation strategy [98]. Both standard and bootstrap variants of Hotelling’s *T*^2^ [99] and log likelihood ratio [100] tests were also used to obtain parametric and non-parametric estimates of the statistical significance of the training-set group separations. Image models for the CVA axes were calculated using the method described by MacLeod [101].

While results generated by an EFA-based analysis of outlines can be considered valid for the data that were analyzed, these data, like any conceivable set of landmarks or combinations of landmarks and semilandmarks, represent only a small subset of the morphological data available for analysis. Indeed, far more data, in the form of qualitative observations, are employed routinely by systematists to address a wide variety of biologically relevant issues. Moreover, even after designating cranial outlines as shapes of particular interest, the application of biometric procedures to such data can still result in substantial degrees of ambiguity regarding whether the differences identified by linear discriminant analysis are truly representative of the morphology as a whole, irrespective of their statistical significance [32, 36]. Alternative approaches to the analysis of morphological data, especially those derived from the fields of computer vision and machine learning, may represent more refined tools for use in such contexts.

In order to determine whether the EFA-PCA-CVA-based group-separation results and statistical significance values could be improved through utilization of a machine-learning approach to group characterization, the well-known Naïve Bayes (NB) identification procedure [102, 103] was also applied to the scores of the harmonic coefficient values on the retained PCA axes. The NB classifier is well-suited to analyzing PCA-score data insofar as it assumes intervariable independence [103] and is known to work well when the dimensionality of the identification problem is high, but the number of samples available for group characterization is relatively low [102, 103], as was the case with the sample investigated here.

A “deep learning” convolution neural network (CNN) analysis was also employed to analyze the image pixel sets directly in the form of the LeNet-5 architecture [104, 105]. Arguably, LeNet was the CNN that sparked initial interest in “deep learning” using convolution-based, multilayer artificial neural networks. The LeNet-5 architecture achieved 98.5% accuracy when tested on the 10,000 test images included in the 70,000-image Modified Nation Institute of Standards and Technology (MNIST) image database (see http://yann.lecun.com/exdb/mnist/) after being trained on the remaining 60,000 28 × 28 pixel digital images.

All CNNs consist of an input layer that receives the information to be processed (in our case images) and an output layer that makes the final allocation of the processed data into one of a number of categories or classes. Between these, a variable number of connected or “hidden” layers exist that process the data by (1) accepting the information from the input or previous layers, (2) evaluating this information for patterns that are consistent with those established by a previously identified training set that have been allocated to their appropriate categories (in our investigation, sex), and (3) passing this processed data on to the next layer. This layered design is used to overcome the problem of full connectivity which is impractical to apply to large images, but can be applied successfully to small images [30, 106, 107]. For our analysis, we adopted the standard LeNet default of autoencoding, or “stepping down” the input image resolutions to 28 × 28 8-bit grayscale pixel values as an initial processing step. Although LeNet-5 is but one of several advanced, gradient-descent CNN architectures for image-based automated identification applications (see https://resources.wolframcloud.com/NeuralNetRepository), it remains one of the most efficient, best understood, and most flexible of the CNN architectures available currently. The overall structure of the LeNet-5 architecture employed in this investigation is listed in Table 7.

**Table 7:** Layer structure of the LeNet-5 “deep learning” CNN employed in this investigation. Sizes refer to pixels for layers 1–7, variables for layers 8–10

Layers	Type	Parameters
		Image
1	Input	3-tensor (size 1 × 28 × 28)
2	Convolution	3-tensor (size 10 × 25 × 25)
3	Ramp	3-tensor (size 10 × 25 × 25)
4	Pooling	3-tensor (size 10 × 12 × 12)
5	Convolution	3-tensor (size 20 × 9 × 9)
6	Ramp	3-tensor (size 20 × 9 × 9)
7	Pooling	3-tensor (size 20 × 4 × 4)
8	Flatten	Vector (size 320)
9	Linear	Vector (size 2)
10	Output	Vector (size 2)

One of the limitations of CNN training is sample size. Owing to the number of interlayer weights whose values must be calculated recursively, CNNs are typically trained on datasets whose sizes are vast by morphological-research standards. A training set, such as ours, consisting of 46 individuals would be considered far too small by most data scientists. Such analyses typically result in overtrained systems that are unreliable when asked to identify genuine unknown specimens.

**Fig. 11:**
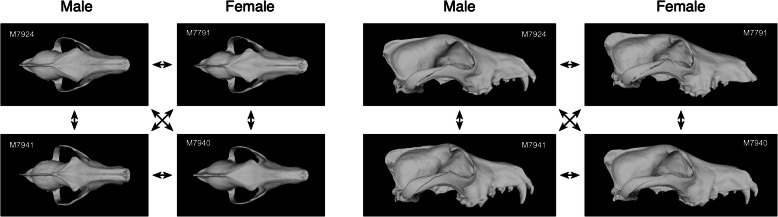
Example of embedded, paired comparison within the Israeli gray wolf (*C. lupus*) cranial skeletal anatomy data, including within-sex group (vertical arrows) and between-sex group (horizontal and diagonal arrows) orientations. The existence of a multitude of paired comparisons such as these, if they are used as the basis for a morphological assessment of within-group similarity and between-group difference, can, in many instances, counteract the effect of inherently small sample sizes. However, in order to be a valid model of population differences, care must be taken either to obtain a representative sample of morphological variation or to be circumspect in interpreting the results of data analyses

Potentially, this problem can be circumvented by opting for training as an embedded learning system, in which the object is not to learn the characteristics of a priori-defined groups themselves but, rather, patterns of explicit similarities and differences between pairs of images that either do or do not belong to the same training group (Fig. 11). Recent published applications of this strategy have focused on systems for describing differences between image pairs drawn from large datasets using text-based descriptors [108, 109] as well as image-based analyses [34, 36]. In terms of the analysis of small to modestly sized samples, there are many advantages to this approach, including relaxation of the use of single assessments of individual forms insofar as all, or most, pairwise comparisons between images in a dataset can be employed for CNN training. Despite the fact that our sample contains only 46 individuals, a total of 2116 pairwise comparisons can be drawn from it: 625 within-group male pairs, 441 within-group female pairs, and 1050 cross-group pairs. By focusing CNN training on differences among images of the same group, and between images of different groups, training can proceed more efficiently than would otherwise be possible.

## Supplementary information

**Additional file 1. **A plate of all 2D images of the *C. lupus* crania in both dorso-ventral and lateral views collected from the 3D scans and used as the proximal subjects of the investigation.

**Additional file 2.** A complete list of all information regarding the specimens used in the study including: collection locality, collection date, collection location coordinate, and catalogue no.

**Additional file 3.** An archive collection of the 3D scans of all specimens used in the study written in the STL format.

**Additional file 4.** An archive catalogue of all original datafiles and results output for the EFA, Naïve Bayes and embedded LeNet-5 CNN analyses for both dorso-ventral and lateral views.

**Additional file 5.** An archive of all code listings for all data procession/analysis software employed in this investigation.

## Data Availability

The image dataset supporting the conclusions of this article is included within the article and additional files documenting intermediate results, along with the Wolfram Mathematica™ scripts for all data-processing and analysis software used in this investigation which are included in the Supplementary Information. The specimens analyzed are all part of the Israeli National Natural History Collections of the Hebrew University of Jerusalem and the Mammalian Collections of the Steinhardt Museum of Natural History, Tel Aviv University. These are available for study or loan by agreement with these institutions.
