# Supplementary material for: Machine-learning strategies for testing patterns of morphological variation in small samples: sexual dimorphism in gray wolf (Canis lupus) crania
Source: BMC Biol. 2020 Sep 3;18:113. doi: 10.1186/s12915-020-00832-1 (PMC7470621; doi:10.1186/s12915-020-00832-1)
Supplement: Supplementary file 1 — Additional file 1. A plate of all 2D images of the C. lupus crania in both dorso-ventral and lateral views collected from the 3D scans and used as the proximal subjects of the investigation. [file 12915_2020_832_MOESM1_ESM.pdf]

SUPPLEMENTARY INFORMATION

Additional File 1

Images of the 46 Gray Wolf crania used in this investigation in dorsal view. Specimen numbers refer to the accession numbers of the National Natural History Collections, Hebrew University of Jerusalem, Israel. Gender identifications were based on museum records. Note damage or deformation has been sustained by specimens: M07940, M07953, M07987, M08039, M08058, M08200, M11108, M12211. This is typical of many museum specimens. In cases where the damage was confined to one side of the cranium the specimen was "healed" via reflection. In cases in which the damage was present on both sides the specimen was included in the analyses with the damaged region(s) intact.

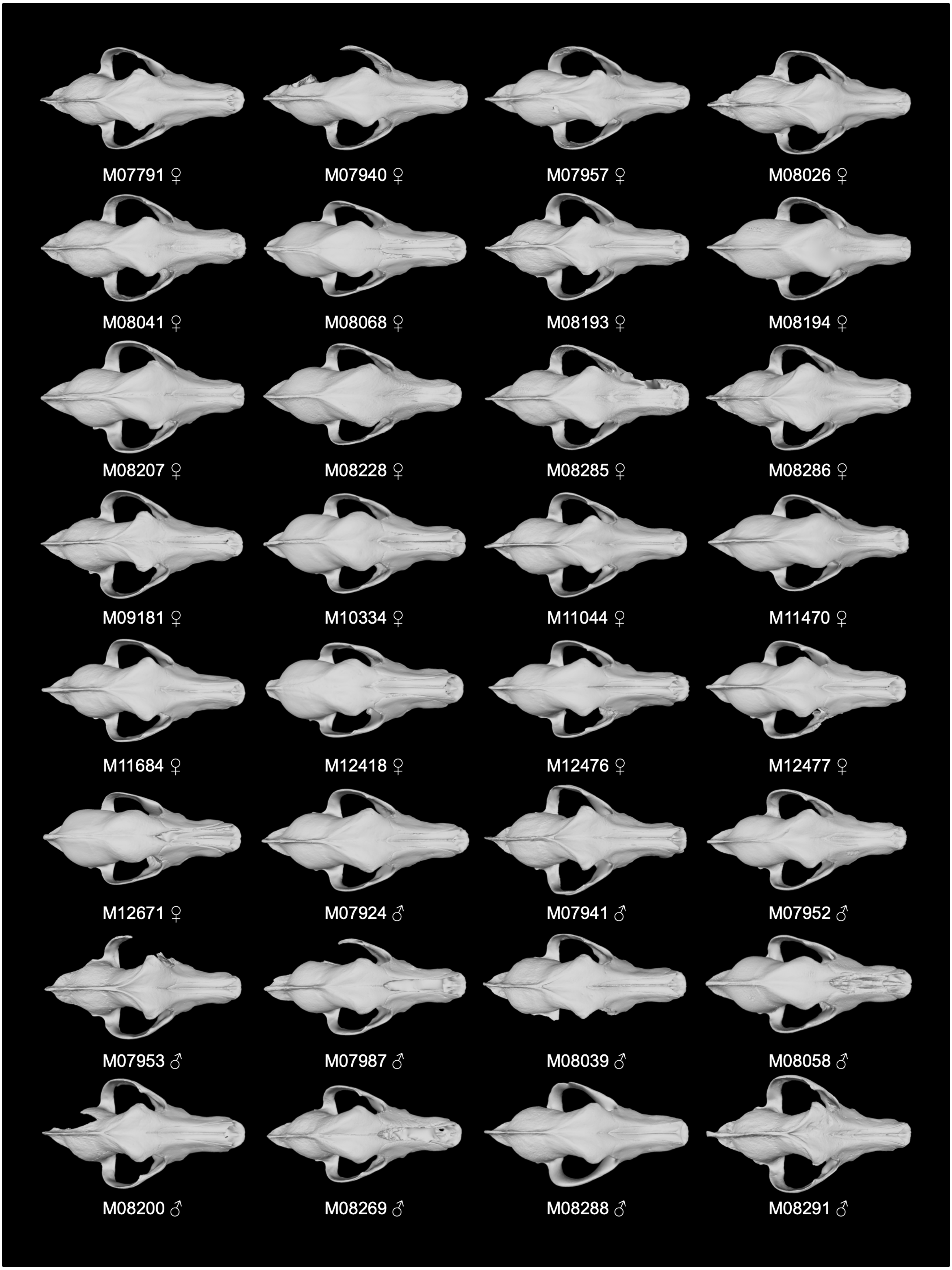
M07791 ♀
M07940 ♀
M07957 ♀
M08026 ♀
M08041 ♀
M08068 ♀
M08193 ♀
M08194 ♀
M08207 ♀
M08228 ♀
M08285 ♀
M08286 ♀
M09181 ♀
M10334 ♀
M11044 ♀
M11470 ♀
M11684 ♀
M12418 ♀
M12476 ♀
M12477 ♀
M12671 ♀
M07924 ♂
M07941 ♂
M07952 ♂
M07953 ♂
M07987 ♂
M08039 ♂
M08058 ♂
M08200 ♂
M08269 ♂
M08288 ♂
M08291 ♂

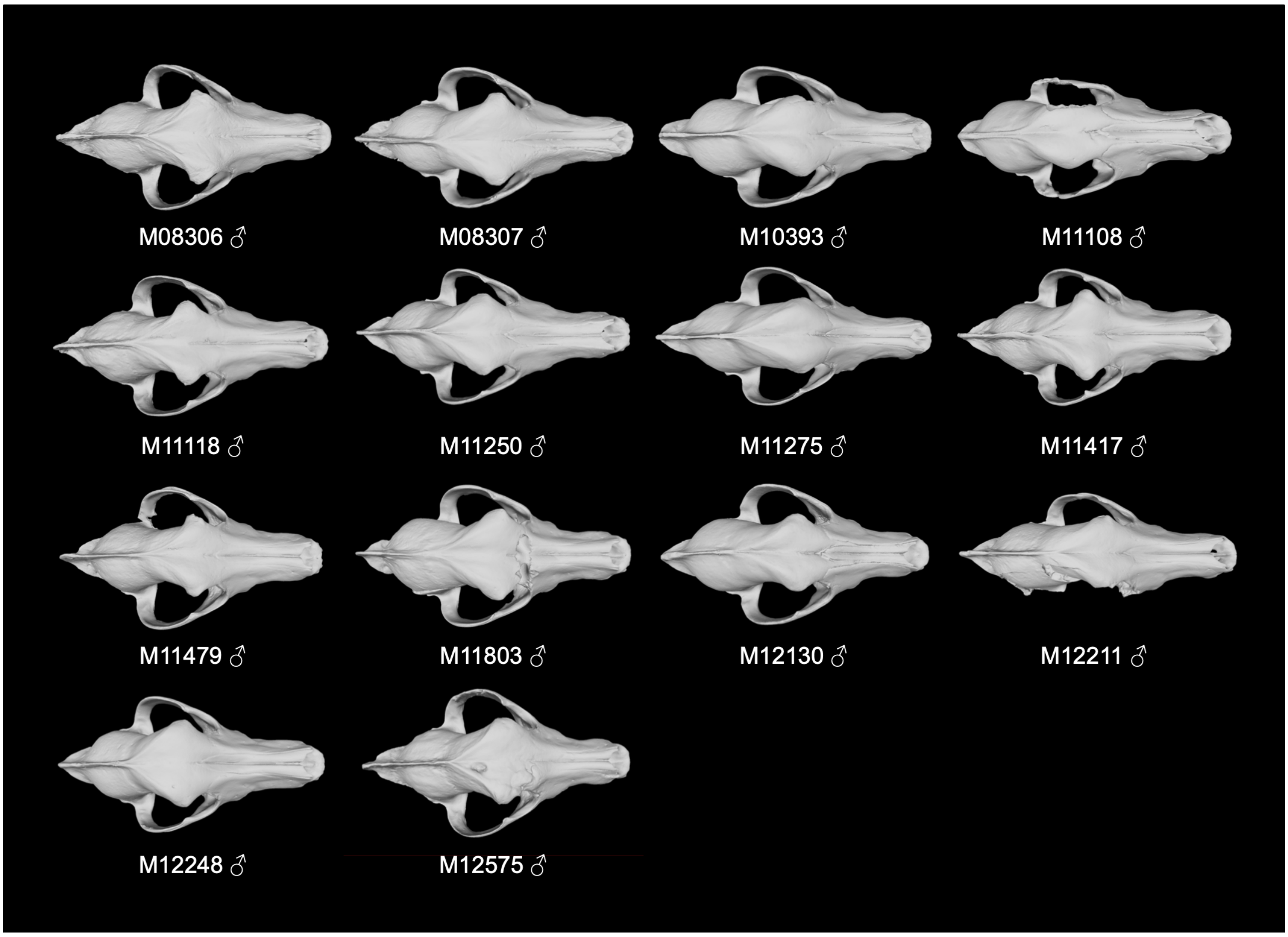
M08306 ♂
M08307 ♂
M10393 ♂
M11108 ♂
M11118 ♂
M11250 ♂
M11275 ♂
M11417 ♂
M11479 ♂
M11803 ♂
M12130 ♂
M12211 ♂
M12248 ♂
M12575 ♂

Images of the 46 Gray Wolf crania used in this investigation in lateral (buccal) view. Specimen numbers refer to the accession numbers of the National Natural History Collections, Hebrew University of Jerusalem, Israel. Gender identifications were based on museum records.

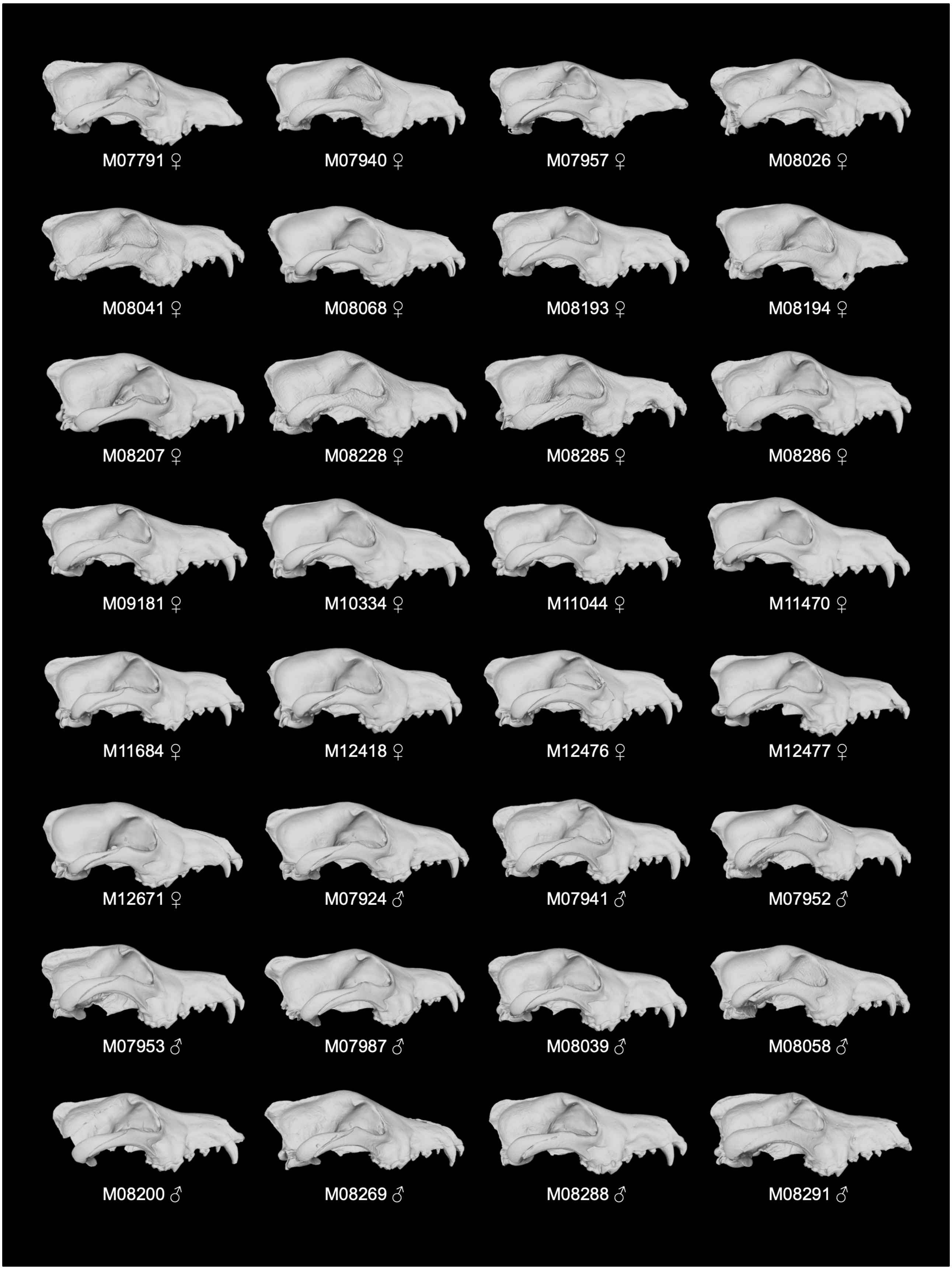
M07791 ♀
M07940 ♀
M07957 ♀
M08026 ♀
M08041 ♀
M08068 ♀
M08193 ♀
M08194 ♀
M08207 ♀
M08228 ♀
M08285 ♀
M08286 ♀
M09181 ♀
M10334 ♀
M11044 ♀
M11470 ♀
M11684 ♀
M12418 ♀
M12476 ♀
M12477 ♀
M12671 ♀
M07924 ♂
M07941 ♂
M07952 ♂
M07953 ♂
M07987 ♂
M08039 ♂
M08058 ♂
M08200 ♂
M08269 ♂
M08288 ♂
M08291 ♂

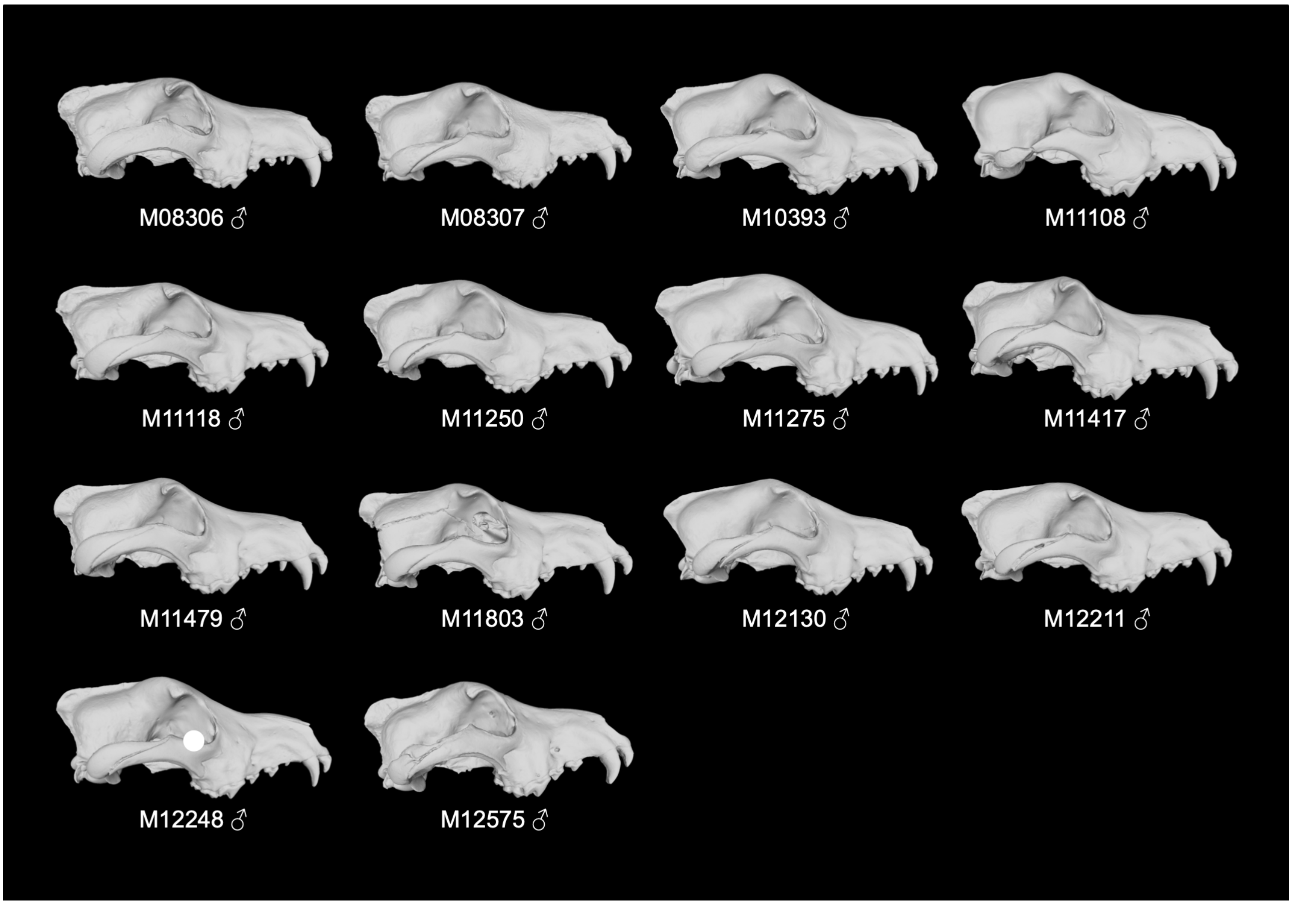

M08306 ♂
M08307 ♂
M10393 ♂
M11108 ♂
M11118 ♂
M11250 ♂
M11275 ♂
M11417 ♂
M11479 ♂
M11803 ♂
M12130 ♂
M12211 ♂
M12248 ♂
M12575 ♂
