# Supplementary material for: Machine-learning strategies for testing patterns of morphological variation in small samples: sexual dimorphism in gray wolf (Canis lupus) crania
Source: BMC Biol. 2020 Sep 3;18:113. doi: 10.1186/s12915-020-00832-1 (PMC7470621; doi:10.1186/s12915-020-00832-1)
Supplement: Supplementary file 2 — Additional file 2. A complete list of all information regarding the specimens used in the study including: collection locality, collection date, collection location coordinate, and catalogue no. [file 12915_2020_832_MOESM2_ESM.pdf]

Study Specimens

Institution	Museum No.	Sex	Collection Year	Collection Region
Hebrew University Jerusalem	7791	Female	1992	Upper Galilee
Hebrew University Jerusalem	7940	Female	1997	Upper Jordan Valley
Hebrew University Jerusalem	7957	Female	1997	Golan
Hebrew University Jerusalem	8026	Female	1997	Upper Galilee
Hebrew University Jerusalem	8041	Female	-	Golan
Hebrew University Jerusalem	8068	Female	1997	Golan
Hebrew University Jerusalem	8193	Female	2002	Golan
Hebrew University Jerusalem	8194	Female	2002	Golan
Hebrew University Jerusalem	8207	Female	1997	Golan
Hebrew University Jerusalem	8228	Female	1999	Golan
Hebrew University Jerusalem	8285	Female	2000	Golan
Hebrew University Jerusalem	8286	Female	2000	Golan
TelAviv University	9181	Female	1992	Upper Galilee
TelAviv University	10334	Female	2005	Upper Galilee
TelAviv University	11044	Female	2004	Upper Galilee
TelAviv University	11470	Female	2005	Upper Galilee
TelAviv University	11684	Female	2006	Upper Galilee
TelAviv University	12418	Female	2009	Upper Galilee
TelAviv University	12476	Female	2009	Upper Galilee
TelAviv University	12477	Female	2009	Upper Galilee
TelAviv University	12671	Female	2009	Upper Galilee
Hebrew University Jerusalem	7924	Male	1994	Upper Jordan Valley
Hebrew University Jerusalem	7941	Male	1994	Golan
Hebrew University Jerusalem	7952	Male	1994	Golan
Hebrew University Jerusalem	7953	Male	1995	Golan
Hebrew University Jerusalem	7987	Male	-	Hula
Hebrew University Jerusalem	8039	Male	1997	Upper Galilee
Hebrew University Jerusalem	8058	Male	1997	Golan
Hebrew University Jerusalem	8200	Male	2002	Golan
Hebrew University Jerusalem	8269	Male	2002	Golan
Hebrew University Jerusalem	8288	Male	2002	Golan
Hebrew University Jerusalem	8291	Male	2002	Golan

Institution	Museum No.	Sex	Collection Year	Collection Region
Hebrew University Jerusalem	8306	Male	2001	Upper Galilee
Hebrew University Jerusalem	8307	Male	2002	Golan
TelAviv University	10393	Male	2004	Upper Galilee
TelAviv University	11108	Male	2005	Upper Galilee
TelAviv University	11118	Male	2004	Upper Galilee
TelAviv University	11250	Male	2005	Upper Galilee
TelAviv University	11275	Male	2005	Upper Galilee
TelAviv University	11417	Male	2005	Upper Galilee
TelAviv University	11479	Male	2005	Upper Galilee
TelAviv University	11803	Male	2007	Upper Galilee
TelAviv University	12130	Male	2007	Upper Galilee
TelAviv University	12211	Male	2008	Upper Galilee
TelAviv University	12248	Male	2009	Upper Galilee
TelAviv University	12475	Male	2010	Upper Galilee
