# Supplementary figures and images for: Machine-learning strategies for testing patterns of morphological variation in small samples: sexual dimorphism in gray wolf (Canis lupus) crania
Source: BMC Biol. 2020 Sep 3;18:113. doi: 10.1186/s12915-020-00832-1 (PMC7470621; doi:10.1186/s12915-020-00832-1)

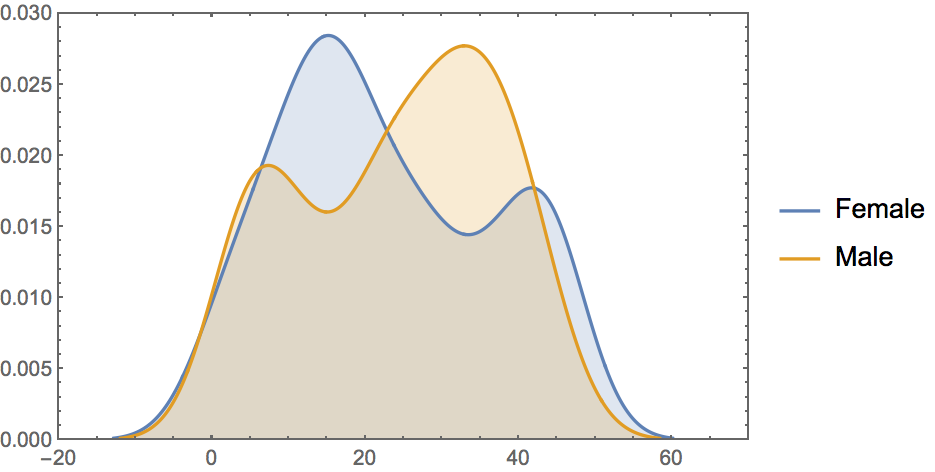

Supplement: Supplementary file 4 — Additional file 4. An archive catalogue of all original datafiles and results output for the EFA, Naïve Bayes and embedded LeNet-5 CNN analyses for both dorso-ventral and lateral views. [file 12915_2020_832_MOESM4_ESM.zip › SI FIle 4/Dorsal Analyses/Centroid Size Results/CS Histogram copy.tif]

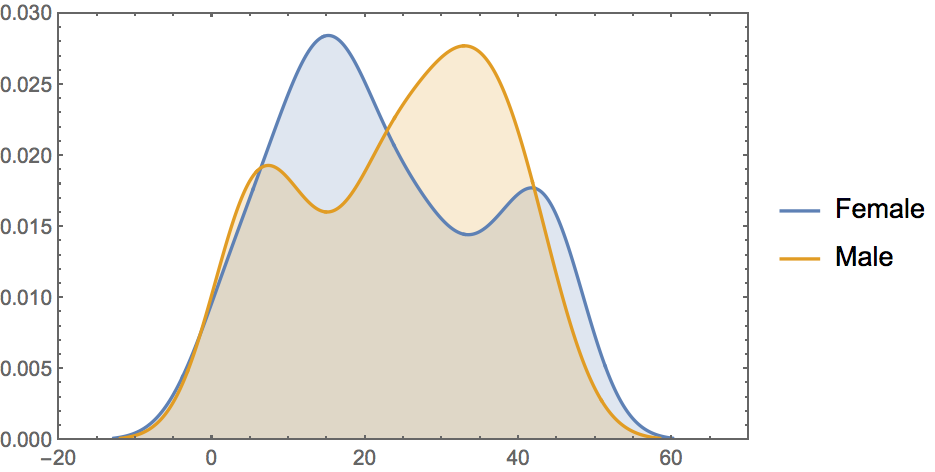

Supplement: Supplementary file 4 — Additional file 4. An archive catalogue of all original datafiles and results output for the EFA, Naïve Bayes and embedded LeNet-5 CNN analyses for both dorso-ventral and lateral views. [file 12915_2020_832_MOESM4_ESM.zip › SI FIle 4/Dorsal Analyses/Centroid Size Results/CS Histogram.tif]

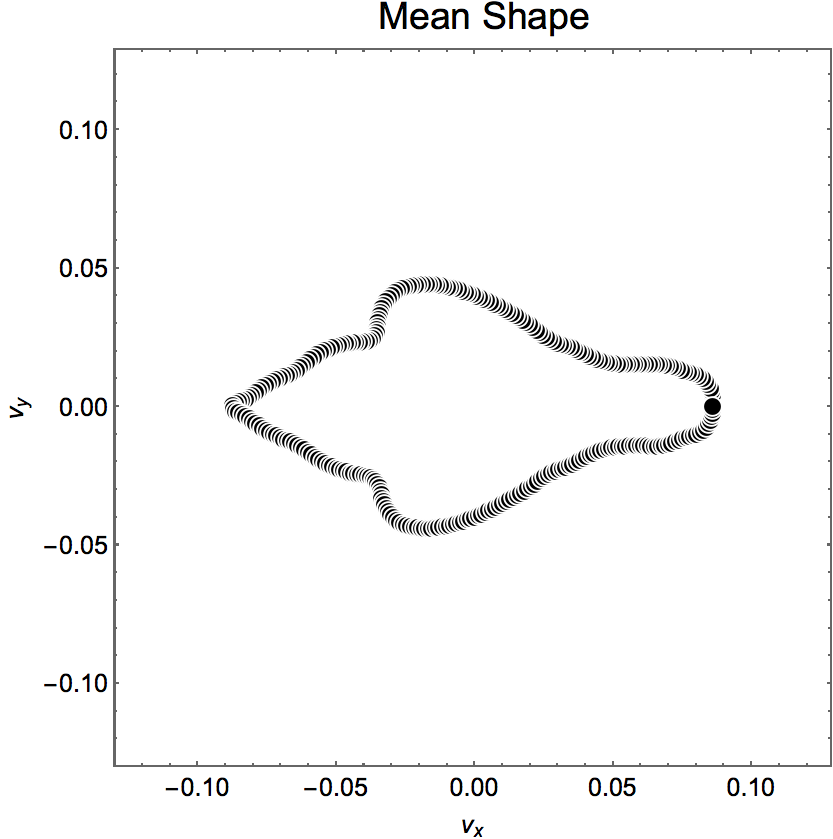

Supplement: Supplementary file 4 — Additional file 4. An archive catalogue of all original datafiles and results output for the EFA, Naïve Bayes and embedded LeNet-5 CNN analyses for both dorso-ventral and lateral views. [file 12915_2020_832_MOESM4_ESM.zip › SI FIle 4/Dorsal Analyses/EFourier Data & Results/Dorsal Outlines (Mean Shape).tif]

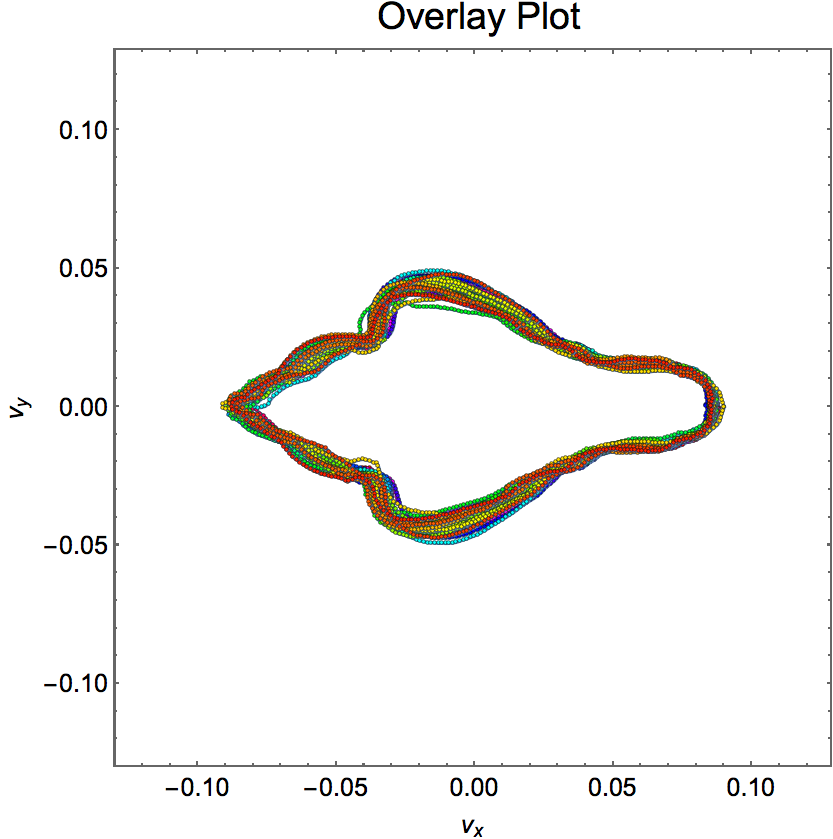

Supplement: Supplementary file 4 — Additional file 4. An archive catalogue of all original datafiles and results output for the EFA, Naïve Bayes and embedded LeNet-5 CNN analyses for both dorso-ventral and lateral views. [file 12915_2020_832_MOESM4_ESM.zip › SI FIle 4/Dorsal Analyses/EFourier Data & Results/Dorsal Outlines (Overlay Plot).tif]

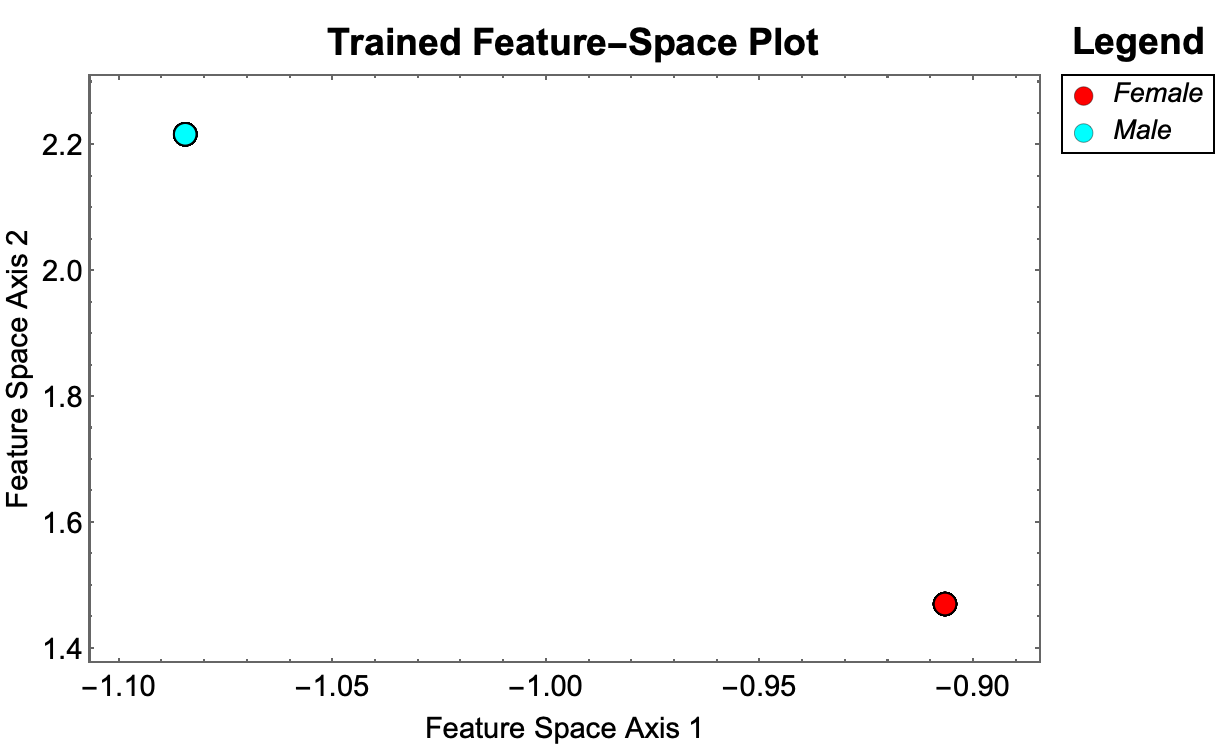

Supplement: Supplementary file 4 — Additional file 4. An archive catalogue of all original datafiles and results output for the EFA, Naïve Bayes and embedded LeNet-5 CNN analyses for both dorso-ventral and lateral views. [file 12915_2020_832_MOESM4_ESM.zip › SI FIle 4/Dorsal Analyses/LeNet (Embedded) Results/Trained Feature Space Plot.tif]

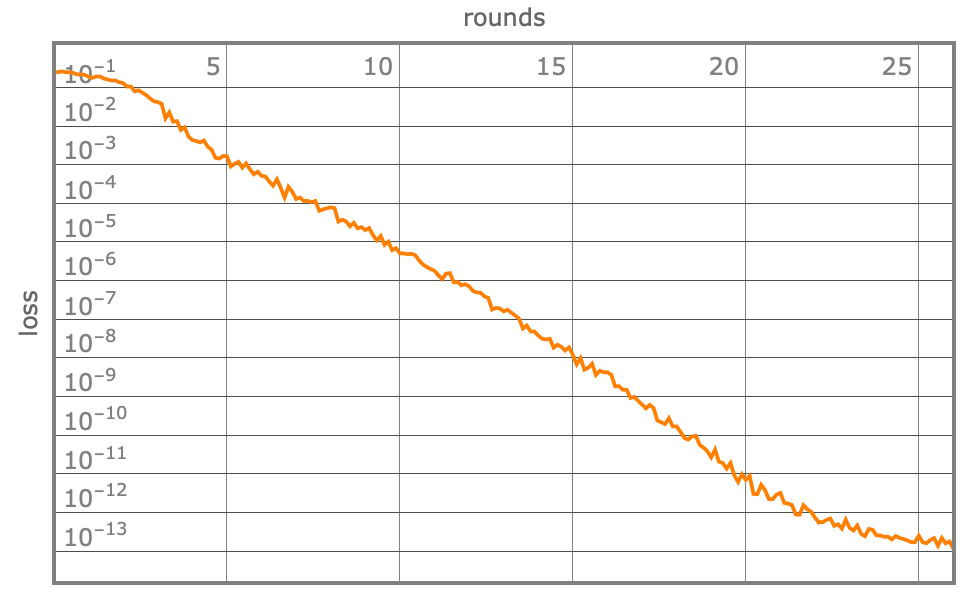

Supplement: Supplementary file 4 — Additional file 4. An archive catalogue of all original datafiles and results output for the EFA, Naïve Bayes and embedded LeNet-5 CNN analyses for both dorso-ventral and lateral views. [file 12915_2020_832_MOESM4_ESM.zip › SI FIle 4/Dorsal Analyses/LeNet (Embedded) Results/Training Log.tiff]

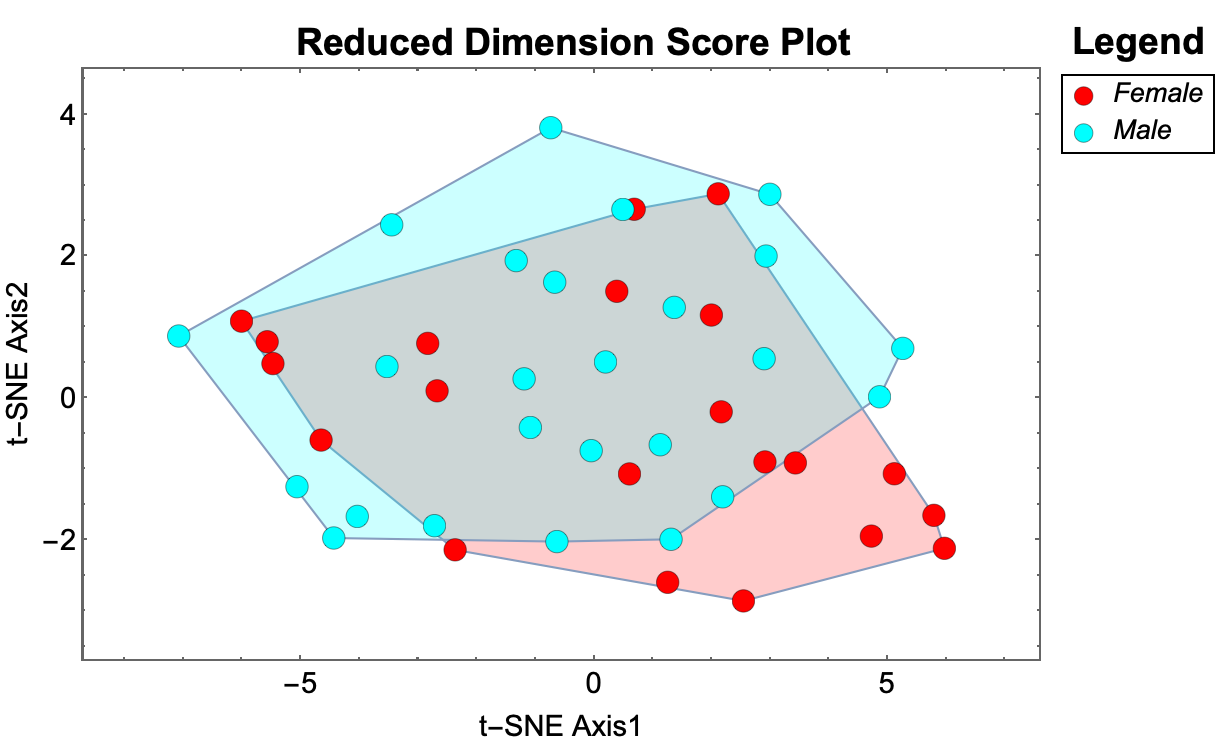

Supplement: Supplementary file 4 — Additional file 4. An archive catalogue of all original datafiles and results output for the EFA, Naïve Bayes and embedded LeNet-5 CNN analyses for both dorso-ventral and lateral views. [file 12915_2020_832_MOESM4_ESM.zip › SI FIle 4/Dorsal Analyses/LeNet (Embedded) Results/Untrained (TSNE) Feature Space Plot (w: Labels).tif]

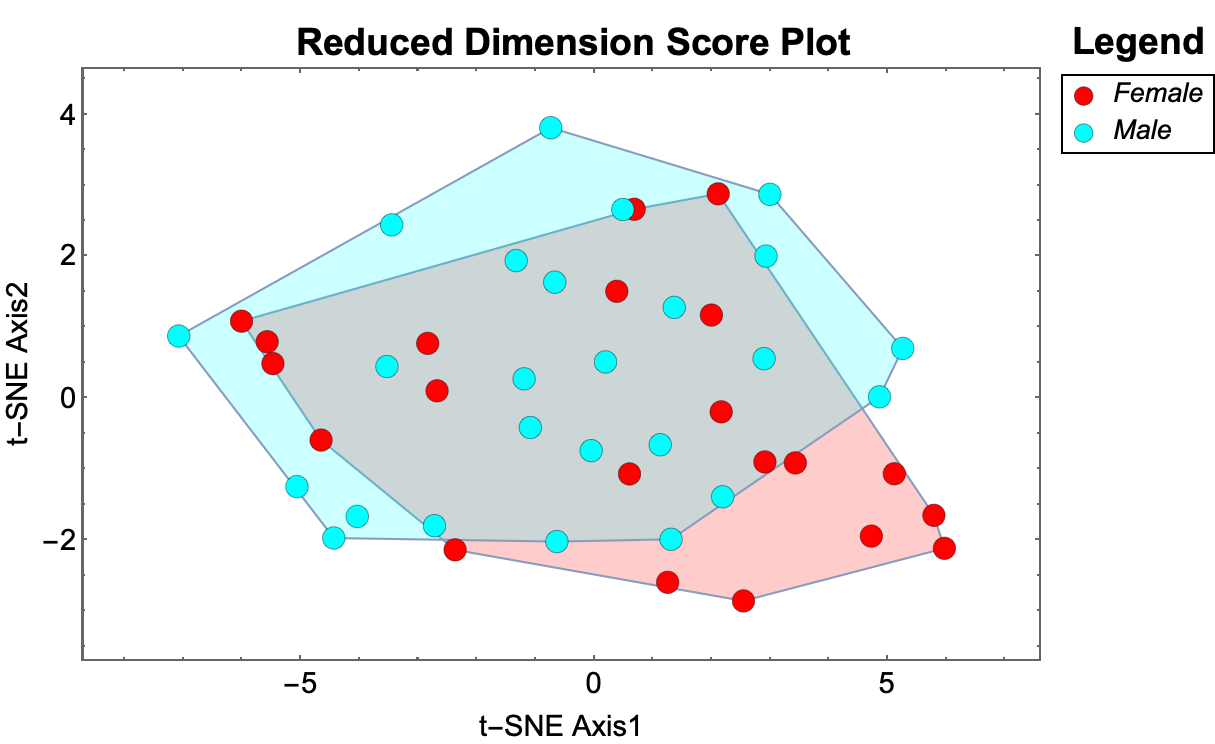

Supplement: Supplementary file 4 — Additional file 4. An archive catalogue of all original datafiles and results output for the EFA, Naïve Bayes and embedded LeNet-5 CNN analyses for both dorso-ventral and lateral views. [file 12915_2020_832_MOESM4_ESM.zip › SI FIle 4/Dorsal Analyses/LeNet (Embedded) Results/Untrained (TSNE) Feature Space Plot.tif]

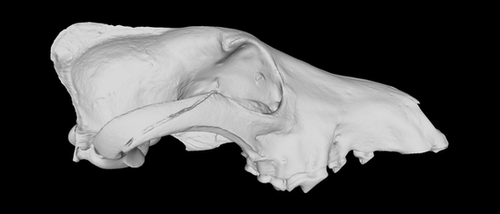

Supplement: Supplementary file 4 — Additional file 4. An archive catalogue of all original datafiles and results output for the EFA, Naïve Bayes and embedded LeNet-5 CNN analyses for both dorso-ventral and lateral views. [file 12915_2020_832_MOESM4_ESM.zip › SI FIle 4/Images Analyses/Dorsal/M07791 (ΓÖÇ).tif]

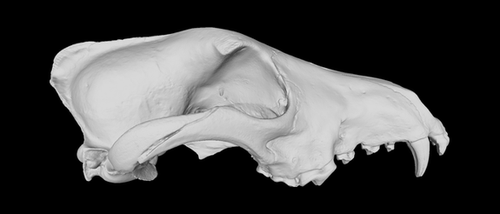

Supplement: Supplementary file 4 — Additional file 4. An archive catalogue of all original datafiles and results output for the EFA, Naïve Bayes and embedded LeNet-5 CNN analyses for both dorso-ventral and lateral views. [file 12915_2020_832_MOESM4_ESM.zip › SI FIle 4/Images Analyses/Dorsal/M07924 (ΓÖé).tif]

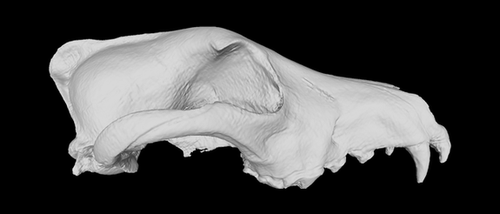

Supplement: Supplementary file 4 — Additional file 4. An archive catalogue of all original datafiles and results output for the EFA, Naïve Bayes and embedded LeNet-5 CNN analyses for both dorso-ventral and lateral views. [file 12915_2020_832_MOESM4_ESM.zip › SI FIle 4/Images Analyses/Dorsal/M07940 (ΓÖÇ).tif]

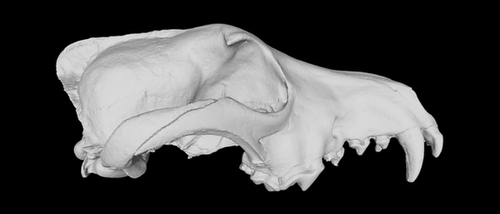

Supplement: Supplementary file 4 — Additional file 4. An archive catalogue of all original datafiles and results output for the EFA, Naïve Bayes and embedded LeNet-5 CNN analyses for both dorso-ventral and lateral views. [file 12915_2020_832_MOESM4_ESM.zip › SI FIle 4/Images Analyses/Dorsal/M07941 (ΓÖé).tif]

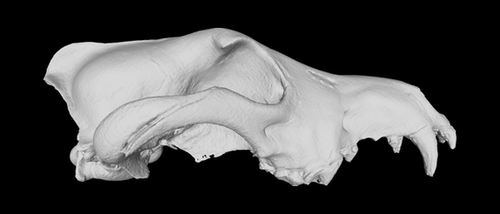

Supplement: Supplementary file 4 — Additional file 4. An archive catalogue of all original datafiles and results output for the EFA, Naïve Bayes and embedded LeNet-5 CNN analyses for both dorso-ventral and lateral views. [file 12915_2020_832_MOESM4_ESM.zip › SI FIle 4/Images Analyses/Dorsal/M07952 (ΓÖé).tif]

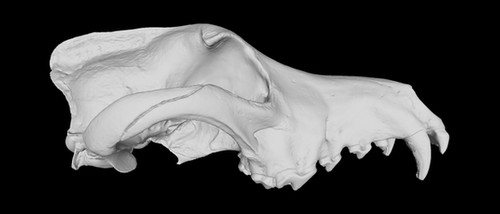

Supplement: Supplementary file 4 — Additional file 4. An archive catalogue of all original datafiles and results output for the EFA, Naïve Bayes and embedded LeNet-5 CNN analyses for both dorso-ventral and lateral views. [file 12915_2020_832_MOESM4_ESM.zip › SI FIle 4/Images Analyses/Dorsal/M07953 (ΓÖé).tif]

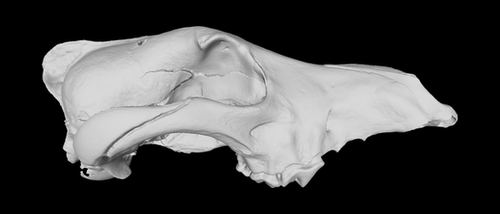

Supplement: Supplementary file 4 — Additional file 4. An archive catalogue of all original datafiles and results output for the EFA, Naïve Bayes and embedded LeNet-5 CNN analyses for both dorso-ventral and lateral views. [file 12915_2020_832_MOESM4_ESM.zip › SI FIle 4/Images Analyses/Dorsal/M07957 (ΓÖÇ).tif]

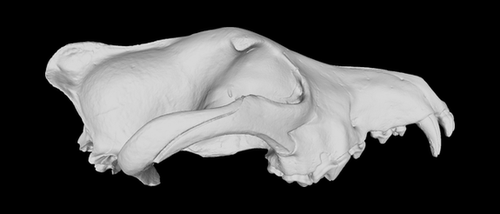

Supplement: Supplementary file 4 — Additional file 4. An archive catalogue of all original datafiles and results output for the EFA, Naïve Bayes and embedded LeNet-5 CNN analyses for both dorso-ventral and lateral views. [file 12915_2020_832_MOESM4_ESM.zip › SI FIle 4/Images Analyses/Dorsal/M07987 (ΓÖé).tif]

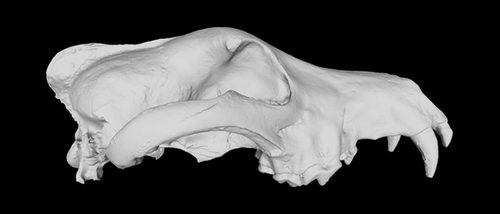

Supplement: Supplementary file 4 — Additional file 4. An archive catalogue of all original datafiles and results output for the EFA, Naïve Bayes and embedded LeNet-5 CNN analyses for both dorso-ventral and lateral views. [file 12915_2020_832_MOESM4_ESM.zip › SI FIle 4/Images Analyses/Dorsal/M08026 (ΓÖÇ).tif]

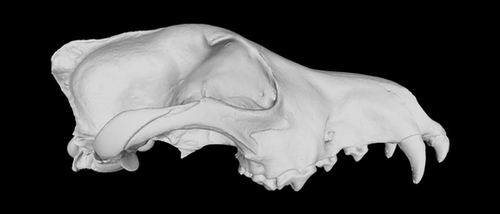

Supplement: Supplementary file 4 — Additional file 4. An archive catalogue of all original datafiles and results output for the EFA, Naïve Bayes and embedded LeNet-5 CNN analyses for both dorso-ventral and lateral views. [file 12915_2020_832_MOESM4_ESM.zip › SI FIle 4/Images Analyses/Dorsal/M08039 (ΓÖé).tif]

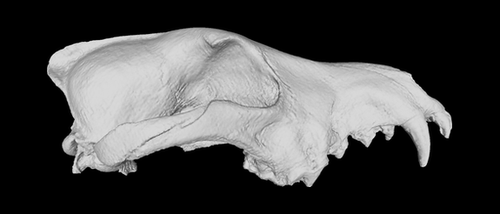

Supplement: Supplementary file 4 — Additional file 4. An archive catalogue of all original datafiles and results output for the EFA, Naïve Bayes and embedded LeNet-5 CNN analyses for both dorso-ventral and lateral views. [file 12915_2020_832_MOESM4_ESM.zip › SI FIle 4/Images Analyses/Dorsal/M08041 (ΓÖÇ).tif]

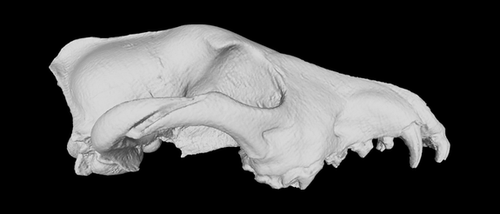

Supplement: Supplementary file 4 — Additional file 4. An archive catalogue of all original datafiles and results output for the EFA, Naïve Bayes and embedded LeNet-5 CNN analyses for both dorso-ventral and lateral views. [file 12915_2020_832_MOESM4_ESM.zip › SI FIle 4/Images Analyses/Dorsal/M08058 (ΓÖé).tif]

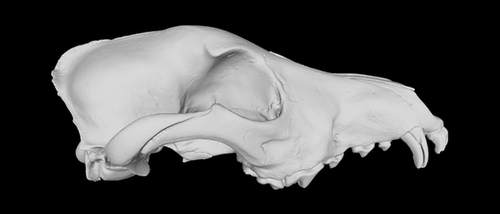

Supplement: Supplementary file 4 — Additional file 4. An archive catalogue of all original datafiles and results output for the EFA, Naïve Bayes and embedded LeNet-5 CNN analyses for both dorso-ventral and lateral views. [file 12915_2020_832_MOESM4_ESM.zip › SI FIle 4/Images Analyses/Dorsal/M08068 (ΓÖÇ).tif]

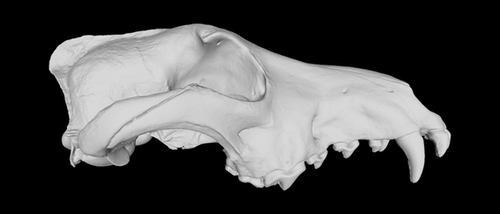

Supplement: Supplementary file 4 — Additional file 4. An archive catalogue of all original datafiles and results output for the EFA, Naïve Bayes and embedded LeNet-5 CNN analyses for both dorso-ventral and lateral views. [file 12915_2020_832_MOESM4_ESM.zip › SI FIle 4/Images Analyses/Dorsal/M08193 (ΓÖÇ).tif]

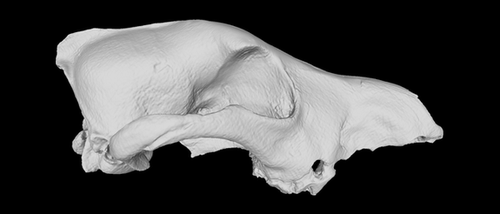

Supplement: Supplementary file 4 — Additional file 4. An archive catalogue of all original datafiles and results output for the EFA, Naïve Bayes and embedded LeNet-5 CNN analyses for both dorso-ventral and lateral views. [file 12915_2020_832_MOESM4_ESM.zip › SI FIle 4/Images Analyses/Dorsal/M08194 (ΓÖÇ).tif]

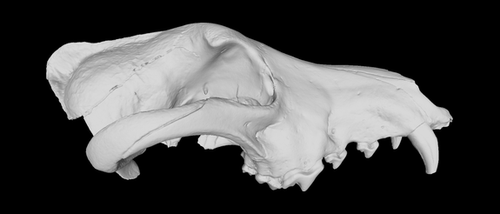

Supplement: Supplementary file 4 — Additional file 4. An archive catalogue of all original datafiles and results output for the EFA, Naïve Bayes and embedded LeNet-5 CNN analyses for both dorso-ventral and lateral views. [file 12915_2020_832_MOESM4_ESM.zip › SI FIle 4/Images Analyses/Dorsal/M08200 (ΓÖé).tif]

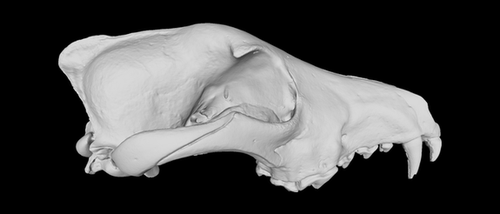

Supplement: Supplementary file 4 — Additional file 4. An archive catalogue of all original datafiles and results output for the EFA, Naïve Bayes and embedded LeNet-5 CNN analyses for both dorso-ventral and lateral views. [file 12915_2020_832_MOESM4_ESM.zip › SI FIle 4/Images Analyses/Dorsal/M08207 (ΓÖÇ).tif]

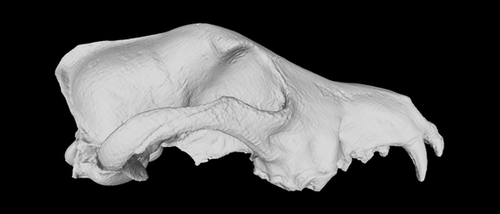

Supplement: Supplementary file 4 — Additional file 4. An archive catalogue of all original datafiles and results output for the EFA, Naïve Bayes and embedded LeNet-5 CNN analyses for both dorso-ventral and lateral views. [file 12915_2020_832_MOESM4_ESM.zip › SI FIle 4/Images Analyses/Dorsal/M08228 (ΓÖÇ).tif]

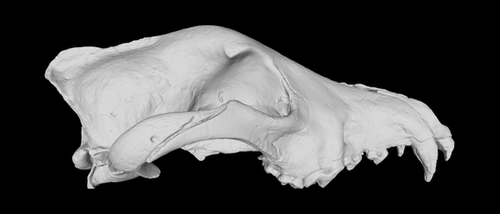

Supplement: Supplementary file 4 — Additional file 4. An archive catalogue of all original datafiles and results output for the EFA, Naïve Bayes and embedded LeNet-5 CNN analyses for both dorso-ventral and lateral views. [file 12915_2020_832_MOESM4_ESM.zip › SI FIle 4/Images Analyses/Dorsal/M08269 (ΓÖé).tif]

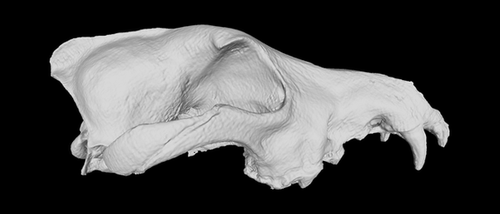

Supplement: Supplementary file 4 — Additional file 4. An archive catalogue of all original datafiles and results output for the EFA, Naïve Bayes and embedded LeNet-5 CNN analyses for both dorso-ventral and lateral views. [file 12915_2020_832_MOESM4_ESM.zip › SI FIle 4/Images Analyses/Dorsal/M08285 (ΓÖÇ).tif]

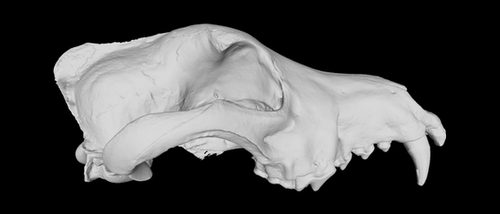

Supplement: Supplementary file 4 — Additional file 4. An archive catalogue of all original datafiles and results output for the EFA, Naïve Bayes and embedded LeNet-5 CNN analyses for both dorso-ventral and lateral views. [file 12915_2020_832_MOESM4_ESM.zip › SI FIle 4/Images Analyses/Dorsal/M08286 (ΓÖÇ).tif]

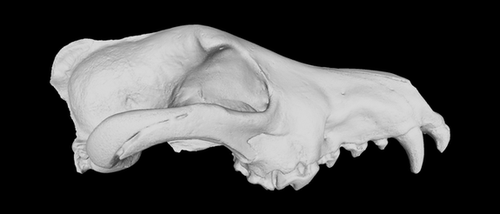

Supplement: Supplementary file 4 — Additional file 4. An archive catalogue of all original datafiles and results output for the EFA, Naïve Bayes and embedded LeNet-5 CNN analyses for both dorso-ventral and lateral views. [file 12915_2020_832_MOESM4_ESM.zip › SI FIle 4/Images Analyses/Dorsal/M08288 (ΓÖé).tif]

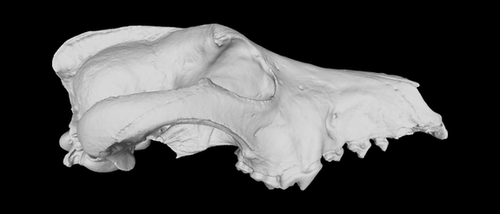

Supplement: Supplementary file 4 — Additional file 4. An archive catalogue of all original datafiles and results output for the EFA, Naïve Bayes and embedded LeNet-5 CNN analyses for both dorso-ventral and lateral views. [file 12915_2020_832_MOESM4_ESM.zip › SI FIle 4/Images Analyses/Dorsal/M08291 (ΓÖé).tif]

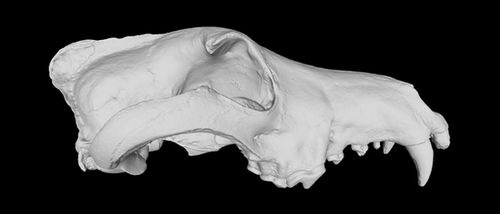

Supplement: Supplementary file 4 — Additional file 4. An archive catalogue of all original datafiles and results output for the EFA, Naïve Bayes and embedded LeNet-5 CNN analyses for both dorso-ventral and lateral views. [file 12915_2020_832_MOESM4_ESM.zip › SI FIle 4/Images Analyses/Dorsal/M08306 (ΓÖé).tif]

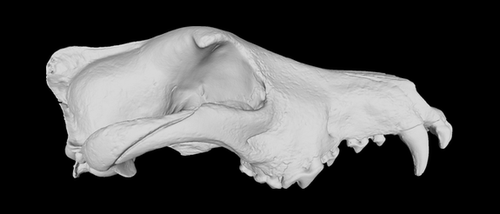

Supplement: Supplementary file 4 — Additional file 4. An archive catalogue of all original datafiles and results output for the EFA, Naïve Bayes and embedded LeNet-5 CNN analyses for both dorso-ventral and lateral views. [file 12915_2020_832_MOESM4_ESM.zip › SI FIle 4/Images Analyses/Dorsal/M08307 (ΓÖé).tif]

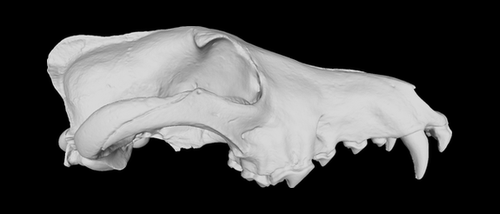

Supplement: Supplementary file 4 — Additional file 4. An archive catalogue of all original datafiles and results output for the EFA, Naïve Bayes and embedded LeNet-5 CNN analyses for both dorso-ventral and lateral views. [file 12915_2020_832_MOESM4_ESM.zip › SI FIle 4/Images Analyses/Dorsal/M09181 (ΓÖÇ).tif]

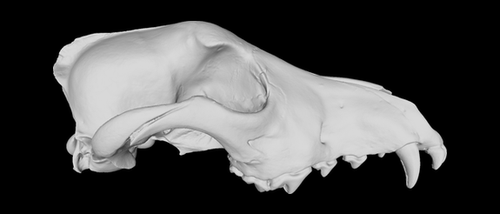

Supplement: Supplementary file 4 — Additional file 4. An archive catalogue of all original datafiles and results output for the EFA, Naïve Bayes and embedded LeNet-5 CNN analyses for both dorso-ventral and lateral views. [file 12915_2020_832_MOESM4_ESM.zip › SI FIle 4/Images Analyses/Dorsal/M10334 (ΓÖÇ).tif]

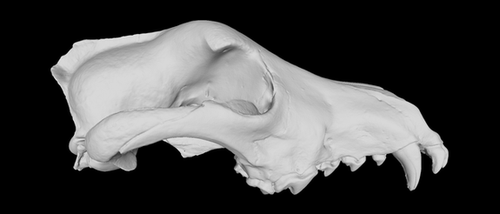

Supplement: Supplementary file 4 — Additional file 4. An archive catalogue of all original datafiles and results output for the EFA, Naïve Bayes and embedded LeNet-5 CNN analyses for both dorso-ventral and lateral views. [file 12915_2020_832_MOESM4_ESM.zip › SI FIle 4/Images Analyses/Dorsal/M10393 (ΓÖé).tif]

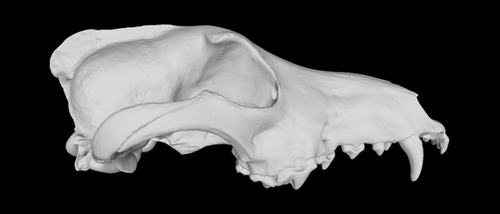

Supplement: Supplementary file 4 — Additional file 4. An archive catalogue of all original datafiles and results output for the EFA, Naïve Bayes and embedded LeNet-5 CNN analyses for both dorso-ventral and lateral views. [file 12915_2020_832_MOESM4_ESM.zip › SI FIle 4/Images Analyses/Dorsal/M11044 (ΓÖÇ).tif]

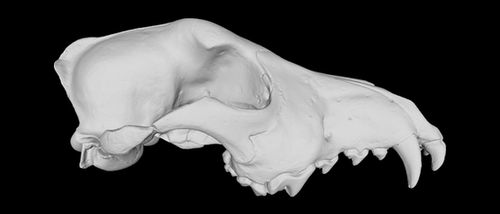

Supplement: Supplementary file 4 — Additional file 4. An archive catalogue of all original datafiles and results output for the EFA, Naïve Bayes and embedded LeNet-5 CNN analyses for both dorso-ventral and lateral views. [file 12915_2020_832_MOESM4_ESM.zip › SI FIle 4/Images Analyses/Dorsal/M11108 (ΓÖé).tif]

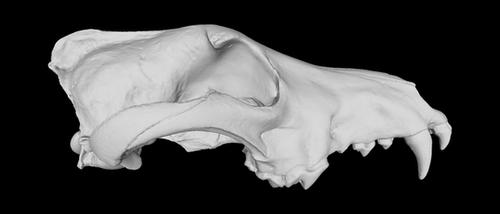

Supplement: Supplementary file 4 — Additional file 4. An archive catalogue of all original datafiles and results output for the EFA, Naïve Bayes and embedded LeNet-5 CNN analyses for both dorso-ventral and lateral views. [file 12915_2020_832_MOESM4_ESM.zip › SI FIle 4/Images Analyses/Dorsal/M11118 (ΓÖé).tif]

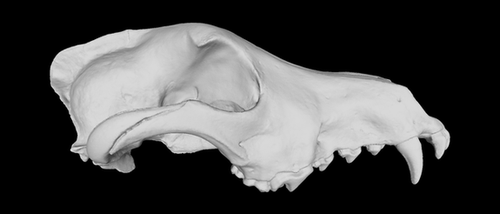

Supplement: Supplementary file 4 — Additional file 4. An archive catalogue of all original datafiles and results output for the EFA, Naïve Bayes and embedded LeNet-5 CNN analyses for both dorso-ventral and lateral views. [file 12915_2020_832_MOESM4_ESM.zip › SI FIle 4/Images Analyses/Dorsal/M11250 (ΓÖé).tif]

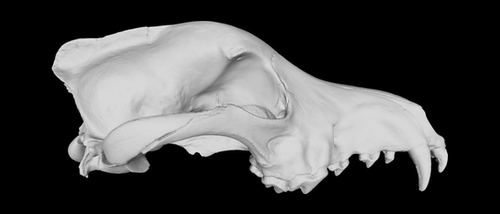

Supplement: Supplementary file 4 — Additional file 4. An archive catalogue of all original datafiles and results output for the EFA, Naïve Bayes and embedded LeNet-5 CNN analyses for both dorso-ventral and lateral views. [file 12915_2020_832_MOESM4_ESM.zip › SI FIle 4/Images Analyses/Dorsal/M11275 (ΓÖé).tif]

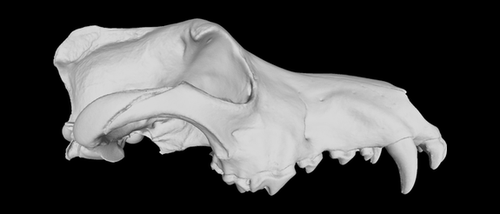

Supplement: Supplementary file 4 — Additional file 4. An archive catalogue of all original datafiles and results output for the EFA, Naïve Bayes and embedded LeNet-5 CNN analyses for both dorso-ventral and lateral views. [file 12915_2020_832_MOESM4_ESM.zip › SI FIle 4/Images Analyses/Dorsal/M11417 (ΓÖé).tif]

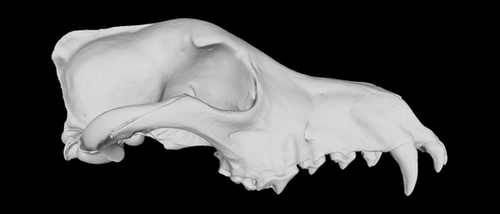

Supplement: Supplementary file 4 — Additional file 4. An archive catalogue of all original datafiles and results output for the EFA, Naïve Bayes and embedded LeNet-5 CNN analyses for both dorso-ventral and lateral views. [file 12915_2020_832_MOESM4_ESM.zip › SI FIle 4/Images Analyses/Dorsal/M11470 (ΓÖÇ).tif]

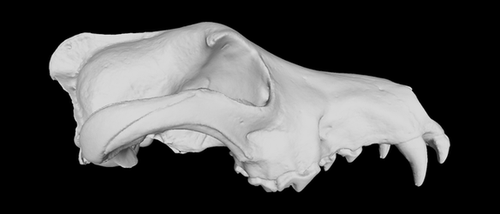

Supplement: Supplementary file 4 — Additional file 4. An archive catalogue of all original datafiles and results output for the EFA, Naïve Bayes and embedded LeNet-5 CNN analyses for both dorso-ventral and lateral views. [file 12915_2020_832_MOESM4_ESM.zip › SI FIle 4/Images Analyses/Dorsal/M11479 (ΓÖé).tif]

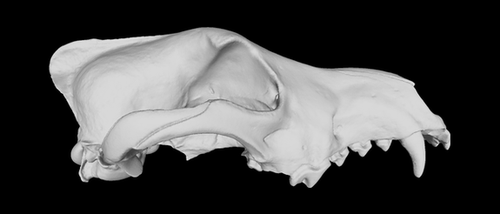

Supplement: Supplementary file 4 — Additional file 4. An archive catalogue of all original datafiles and results output for the EFA, Naïve Bayes and embedded LeNet-5 CNN analyses for both dorso-ventral and lateral views. [file 12915_2020_832_MOESM4_ESM.zip › SI FIle 4/Images Analyses/Dorsal/M11684 (ΓÖÇ).tif]

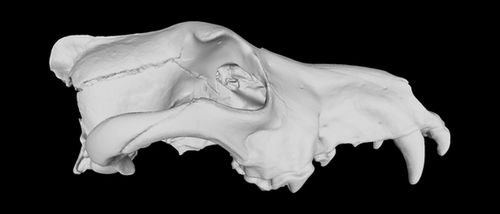

Supplement: Supplementary file 4 — Additional file 4. An archive catalogue of all original datafiles and results output for the EFA, Naïve Bayes and embedded LeNet-5 CNN analyses for both dorso-ventral and lateral views. [file 12915_2020_832_MOESM4_ESM.zip › SI FIle 4/Images Analyses/Dorsal/M11803 (ΓÖé).tif]

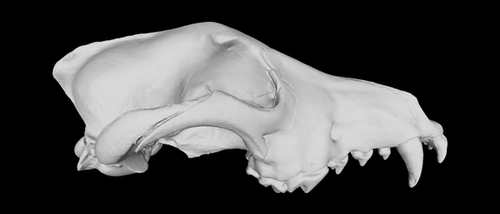

Supplement: Supplementary file 4 — Additional file 4. An archive catalogue of all original datafiles and results output for the EFA, Naïve Bayes and embedded LeNet-5 CNN analyses for both dorso-ventral and lateral views. [file 12915_2020_832_MOESM4_ESM.zip › SI FIle 4/Images Analyses/Dorsal/M12130 (ΓÖé).tif]

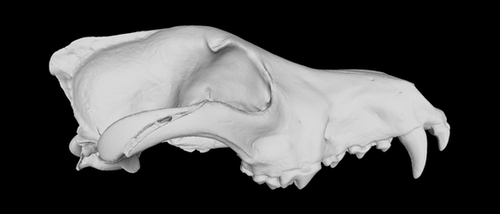

Supplement: Supplementary file 4 — Additional file 4. An archive catalogue of all original datafiles and results output for the EFA, Naïve Bayes and embedded LeNet-5 CNN analyses for both dorso-ventral and lateral views. [file 12915_2020_832_MOESM4_ESM.zip › SI FIle 4/Images Analyses/Dorsal/M12211 (ΓÖé).tif]

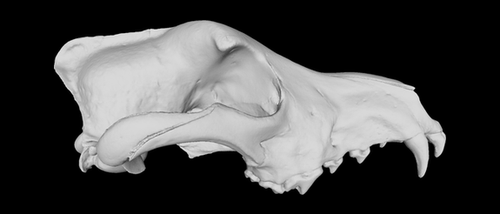

Supplement: Supplementary file 4 — Additional file 4. An archive catalogue of all original datafiles and results output for the EFA, Naïve Bayes and embedded LeNet-5 CNN analyses for both dorso-ventral and lateral views. [file 12915_2020_832_MOESM4_ESM.zip › SI FIle 4/Images Analyses/Dorsal/M12248 (ΓÖé).tif]

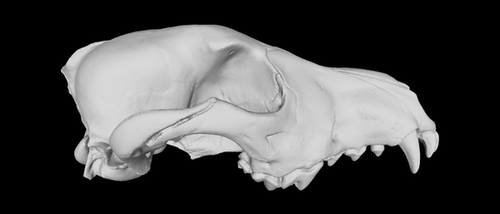

Supplement: Supplementary file 4 — Additional file 4. An archive catalogue of all original datafiles and results output for the EFA, Naïve Bayes and embedded LeNet-5 CNN analyses for both dorso-ventral and lateral views. [file 12915_2020_832_MOESM4_ESM.zip › SI FIle 4/Images Analyses/Dorsal/M12418 (ΓÖÇ).tif]

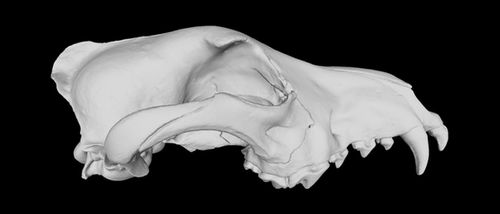

Supplement: Supplementary file 4 — Additional file 4. An archive catalogue of all original datafiles and results output for the EFA, Naïve Bayes and embedded LeNet-5 CNN analyses for both dorso-ventral and lateral views. [file 12915_2020_832_MOESM4_ESM.zip › SI FIle 4/Images Analyses/Dorsal/M12476 (ΓÖÇ).tif]

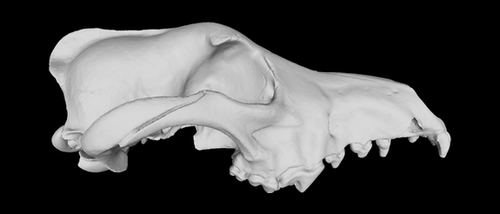

Supplement: Supplementary file 4 — Additional file 4. An archive catalogue of all original datafiles and results output for the EFA, Naïve Bayes and embedded LeNet-5 CNN analyses for both dorso-ventral and lateral views. [file 12915_2020_832_MOESM4_ESM.zip › SI FIle 4/Images Analyses/Dorsal/M12477 (ΓÖÇ).tif]

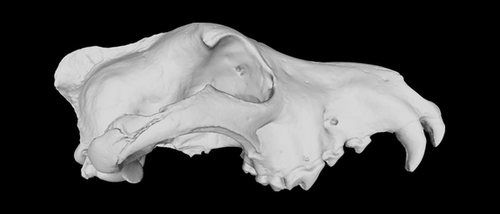

Supplement: Supplementary file 4 — Additional file 4. An archive catalogue of all original datafiles and results output for the EFA, Naïve Bayes and embedded LeNet-5 CNN analyses for both dorso-ventral and lateral views. [file 12915_2020_832_MOESM4_ESM.zip › SI FIle 4/Images Analyses/Dorsal/M12575 (ΓÖé).tif]

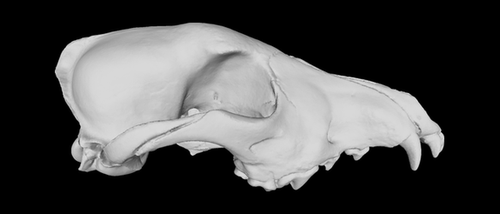

Supplement: Supplementary file 4 — Additional file 4. An archive catalogue of all original datafiles and results output for the EFA, Naïve Bayes and embedded LeNet-5 CNN analyses for both dorso-ventral and lateral views. [file 12915_2020_832_MOESM4_ESM.zip › SI FIle 4/Images Analyses/Dorsal/M12671 (ΓÖÇ).tif]

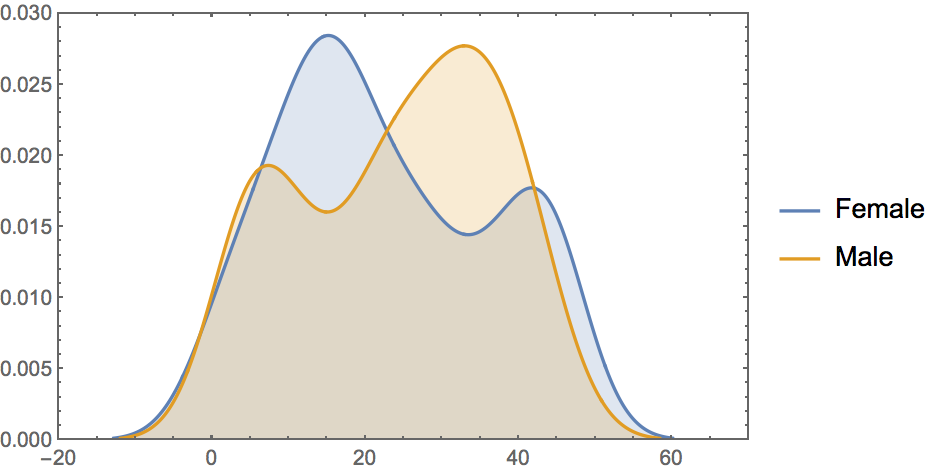

Supplement: Supplementary file 4 — Additional file 4. An archive catalogue of all original datafiles and results output for the EFA, Naïve Bayes and embedded LeNet-5 CNN analyses for both dorso-ventral and lateral views. [file 12915_2020_832_MOESM4_ESM.zip › SI FIle 4/Lateral Analyses/Centroid Size Results/CS Histogram.tif]

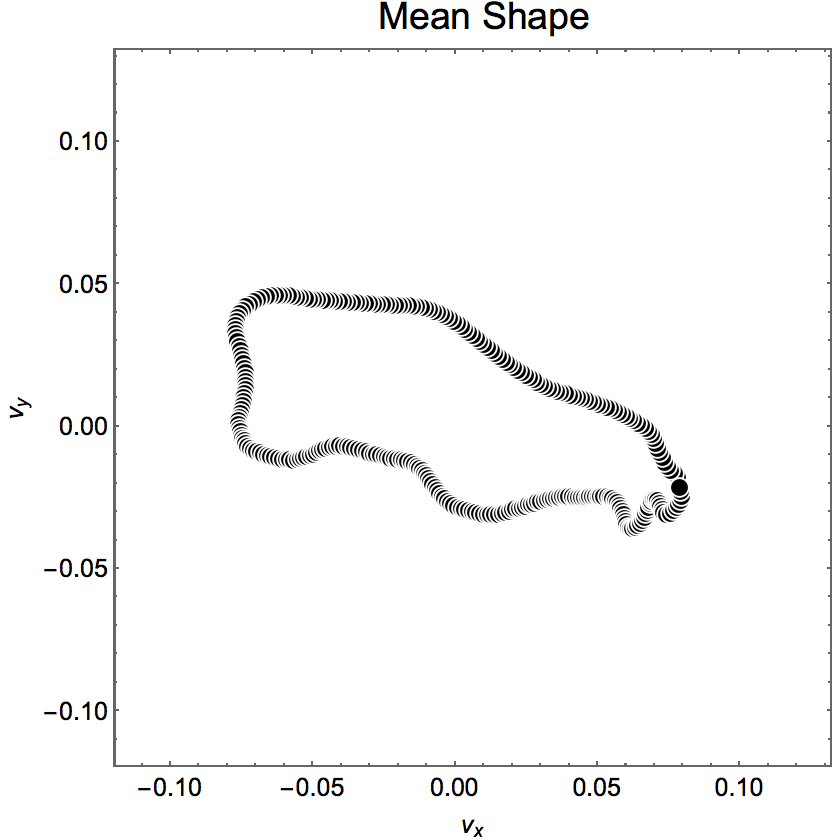

Supplement: Supplementary file 4 — Additional file 4. An archive catalogue of all original datafiles and results output for the EFA, Naïve Bayes and embedded LeNet-5 CNN analyses for both dorso-ventral and lateral views. [file 12915_2020_832_MOESM4_ESM.zip › SI FIle 4/Lateral Analyses/EFourier Data & Results/Lateral Outline (Mean Shape Plot).tif]

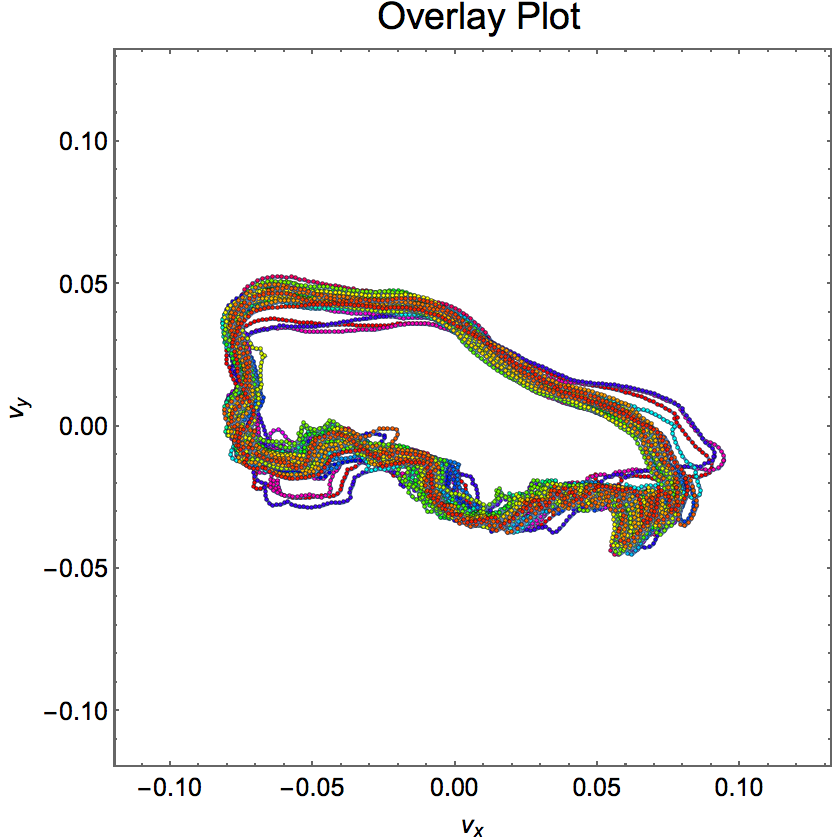

Supplement: Supplementary file 4 — Additional file 4. An archive catalogue of all original datafiles and results output for the EFA, Naïve Bayes and embedded LeNet-5 CNN analyses for both dorso-ventral and lateral views. [file 12915_2020_832_MOESM4_ESM.zip › SI FIle 4/Lateral Analyses/EFourier Data & Results/Lateral Outline (Overlay Plot).tif]

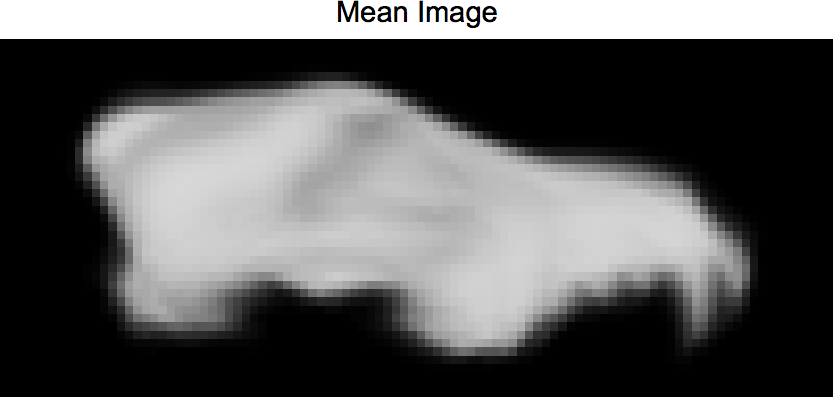

Supplement: Supplementary file 4 — Additional file 4. An archive catalogue of all original datafiles and results output for the EFA, Naïve Bayes and embedded LeNet-5 CNN analyses for both dorso-ventral and lateral views. [file 12915_2020_832_MOESM4_ESM.zip › SI FIle 4/Lateral Analyses/Eigenimage Data & Results/Lateral View (Mean Shape).tif]

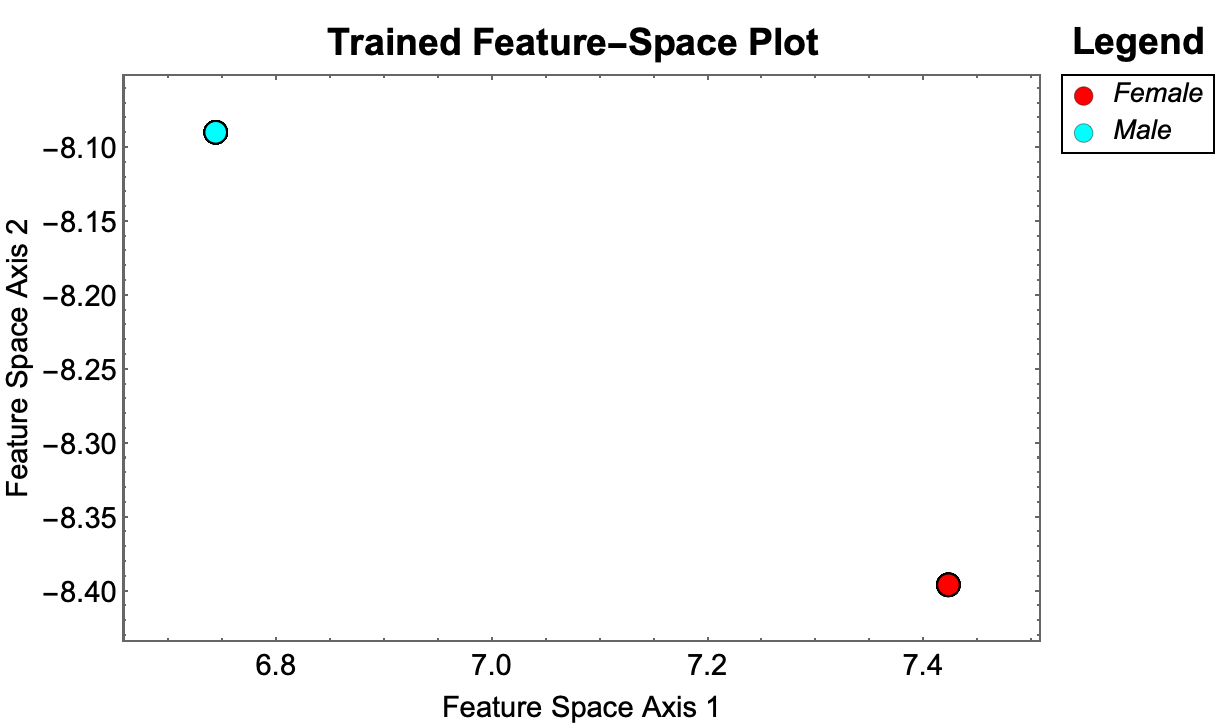

Supplement: Supplementary file 4 — Additional file 4. An archive catalogue of all original datafiles and results output for the EFA, Naïve Bayes and embedded LeNet-5 CNN analyses for both dorso-ventral and lateral views. [file 12915_2020_832_MOESM4_ESM.zip › SI FIle 4/Lateral Analyses/LeNet (Embedded) Results/Trained Feature Space Plot.tif]

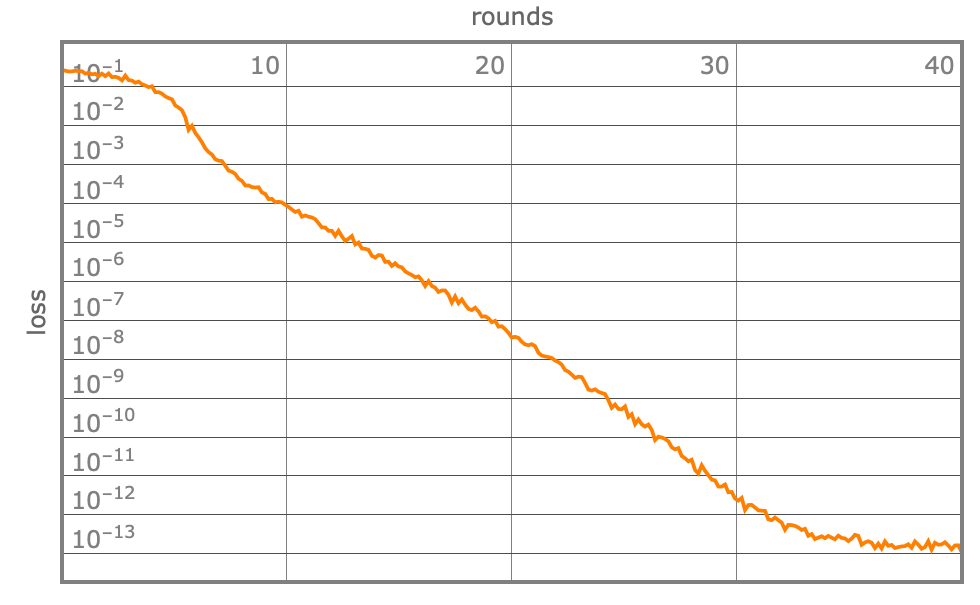

Supplement: Supplementary file 4 — Additional file 4. An archive catalogue of all original datafiles and results output for the EFA, Naïve Bayes and embedded LeNet-5 CNN analyses for both dorso-ventral and lateral views. [file 12915_2020_832_MOESM4_ESM.zip › SI FIle 4/Lateral Analyses/LeNet (Embedded) Results/Training History.tiff]

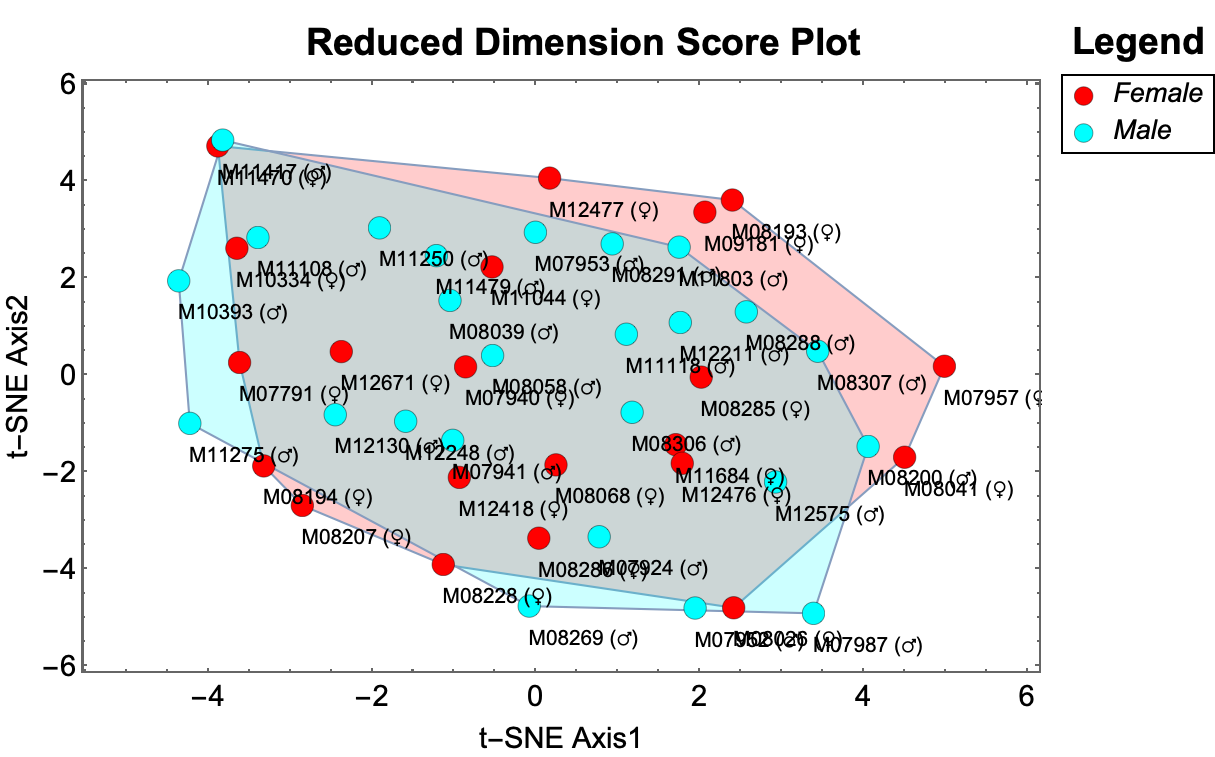

Supplement: Supplementary file 4 — Additional file 4. An archive catalogue of all original datafiles and results output for the EFA, Naïve Bayes and embedded LeNet-5 CNN analyses for both dorso-ventral and lateral views. [file 12915_2020_832_MOESM4_ESM.zip › SI FIle 4/Lateral Analyses/LeNet (Embedded) Results/Untrained (TSNE) Feature Space Plot (w: Lables).tif]

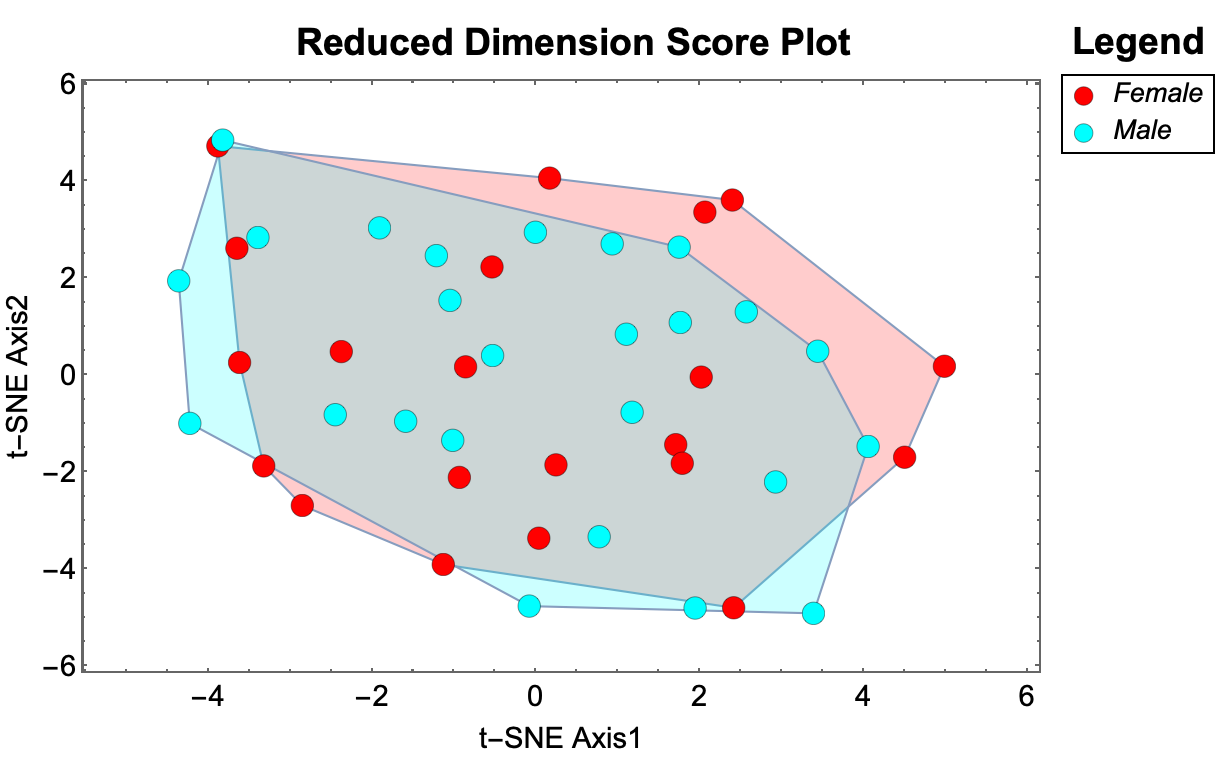

Supplement: Supplementary file 4 — Additional file 4. An archive catalogue of all original datafiles and results output for the EFA, Naïve Bayes and embedded LeNet-5 CNN analyses for both dorso-ventral and lateral views. [file 12915_2020_832_MOESM4_ESM.zip › SI FIle 4/Lateral Analyses/LeNet (Embedded) Results/Untrained (TSNE) Feature Space Plot.tif]

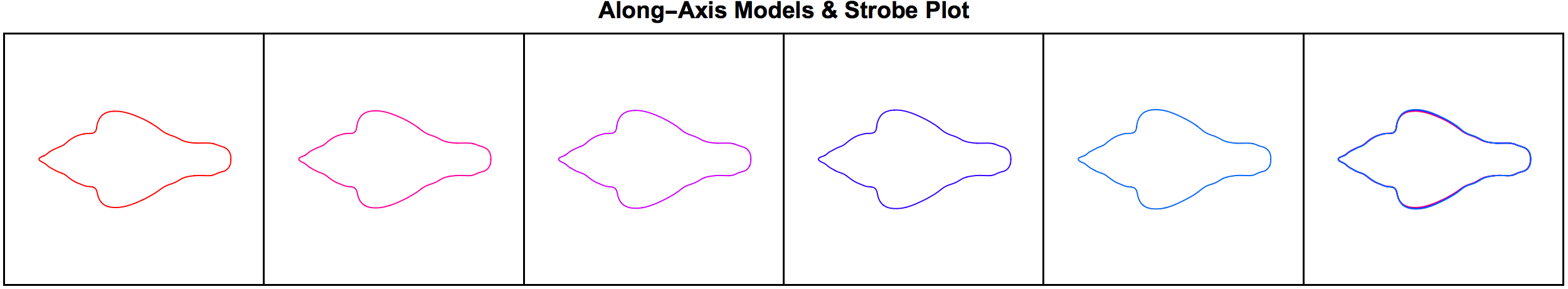

Supplement: Supplementary file 4 — Additional file 4. An archive catalogue of all original datafiles and results output for the EFA, Naïve Bayes and embedded LeNet-5 CNN analyses for both dorso-ventral and lateral views. [file 12915_2020_832_MOESM4_ESM.zip › SI FIle 4/Dorsal Analyses/EFourier Data & Results/CVA Results/Along-Axis Shape Plots (Grid).tif]

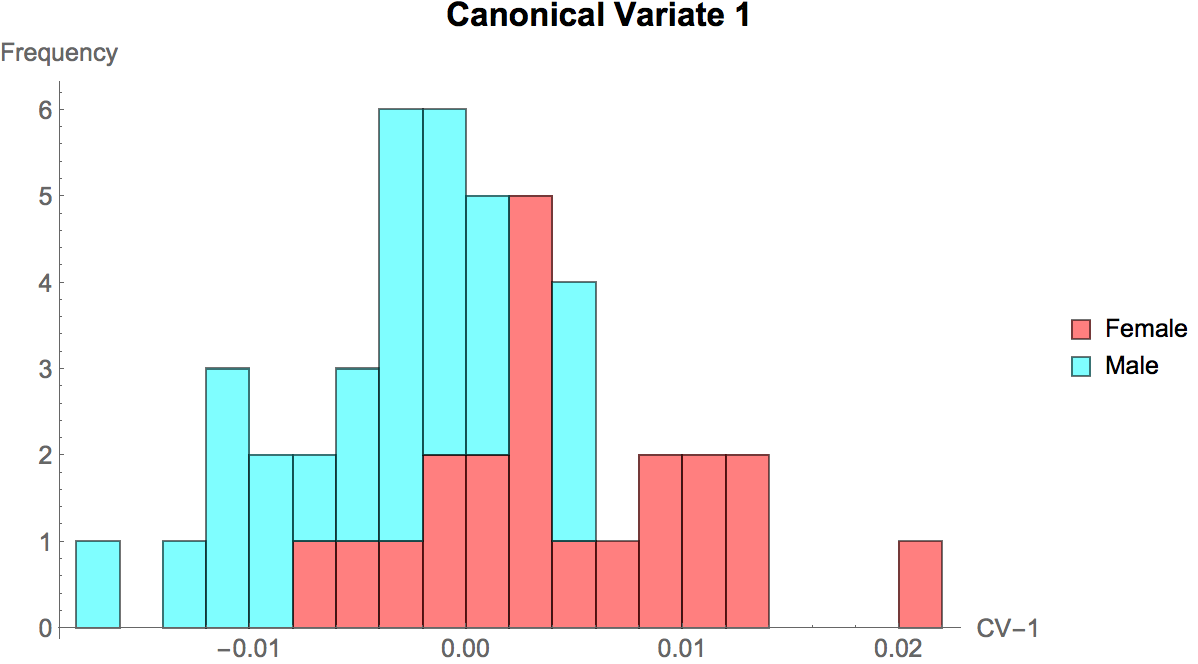

Supplement: Supplementary file 4 — Additional file 4. An archive catalogue of all original datafiles and results output for the EFA, Naïve Bayes and embedded LeNet-5 CNN analyses for both dorso-ventral and lateral views. [file 12915_2020_832_MOESM4_ESM.zip › SI FIle 4/Dorsal Analyses/EFourier Data & Results/CVA Results/CV-1 Histogram.tif]

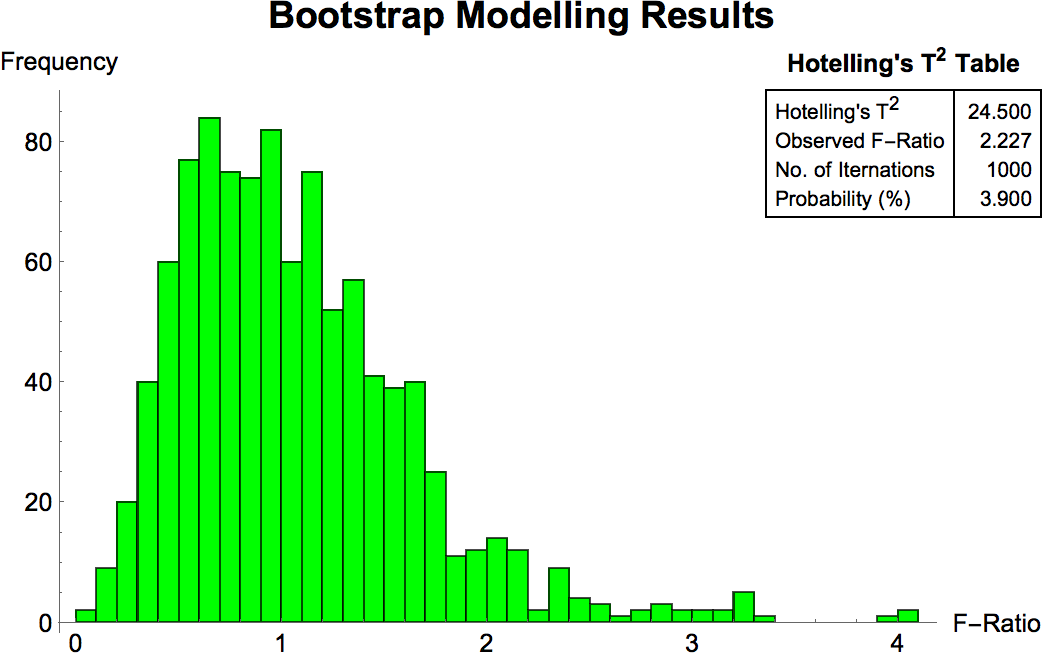

Supplement: Supplementary file 4 — Additional file 4. An archive catalogue of all original datafiles and results output for the EFA, Naïve Bayes and embedded LeNet-5 CNN analyses for both dorso-ventral and lateral views. [file 12915_2020_832_MOESM4_ESM.zip › SI FIle 4/Dorsal Analyses/EFourier Data & Results/CVA Results/Hotellings T2 Test (BS).tif]

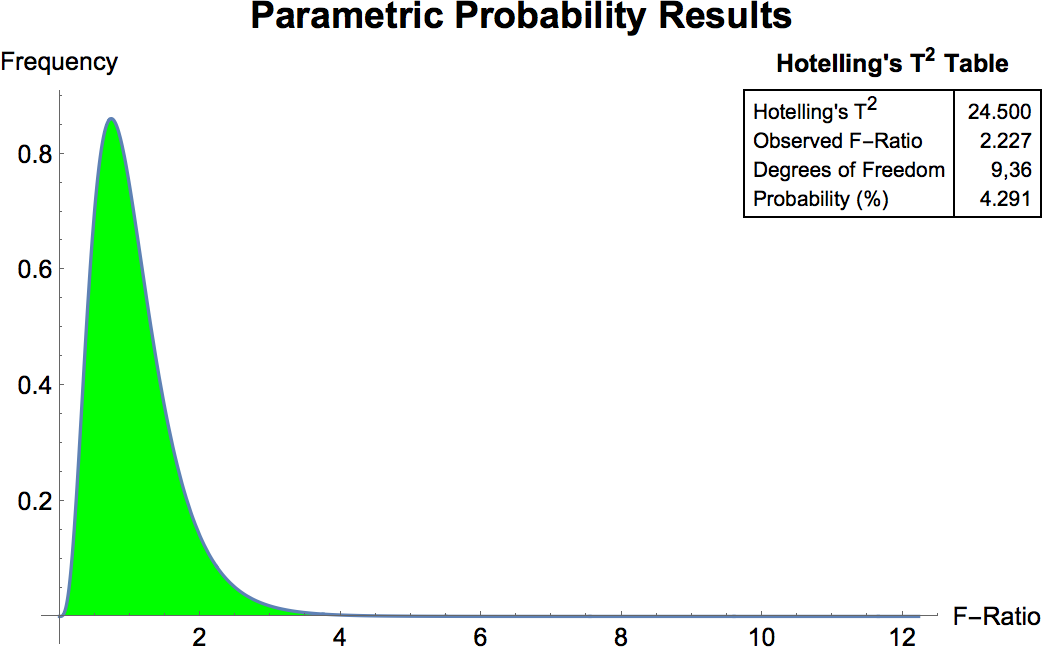

Supplement: Supplementary file 4 — Additional file 4. An archive catalogue of all original datafiles and results output for the EFA, Naïve Bayes and embedded LeNet-5 CNN analyses for both dorso-ventral and lateral views. [file 12915_2020_832_MOESM4_ESM.zip › SI FIle 4/Dorsal Analyses/EFourier Data & Results/CVA Results/Hotellings T2 Test (PM).tif]

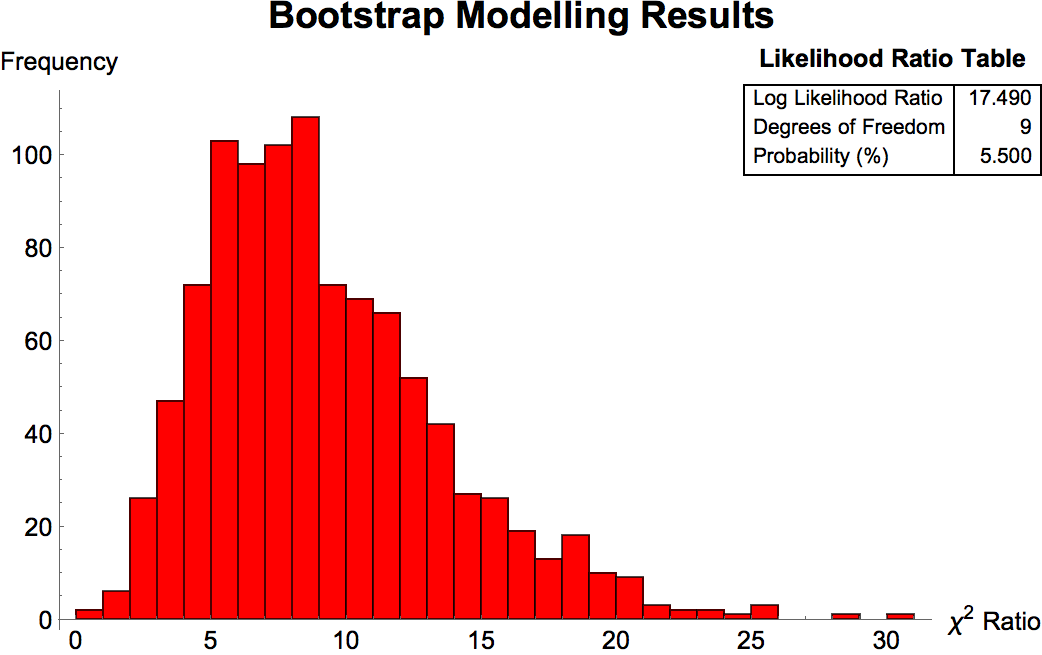

Supplement: Supplementary file 4 — Additional file 4. An archive catalogue of all original datafiles and results output for the EFA, Naïve Bayes and embedded LeNet-5 CNN analyses for both dorso-ventral and lateral views. [file 12915_2020_832_MOESM4_ESM.zip › SI FIle 4/Dorsal Analyses/EFourier Data & Results/CVA Results/Log Likelihood Ratio Test (BS).tif]

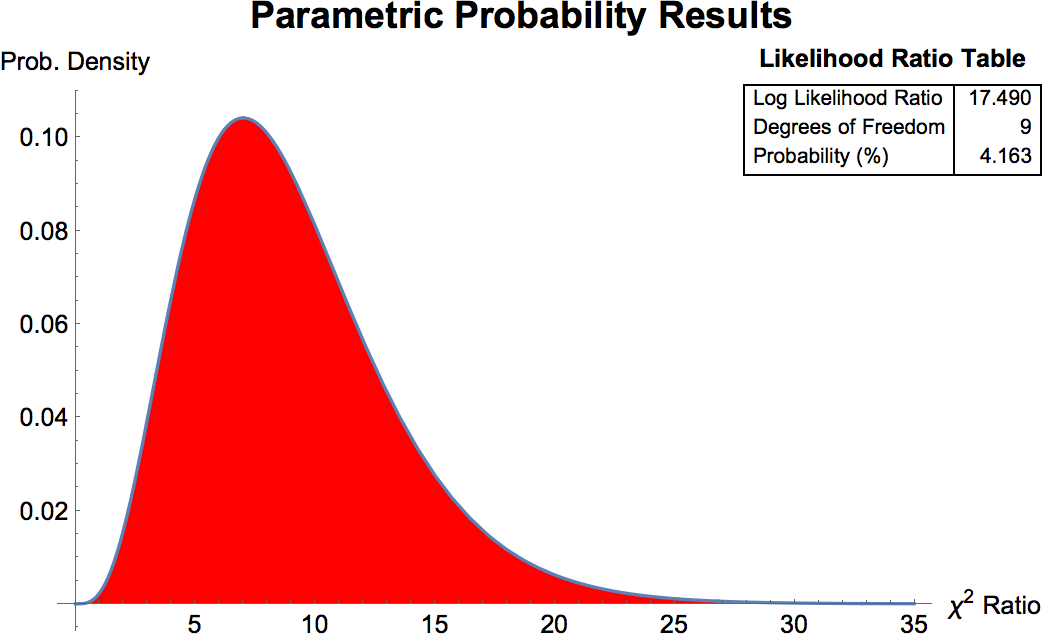

Supplement: Supplementary file 4 — Additional file 4. An archive catalogue of all original datafiles and results output for the EFA, Naïve Bayes and embedded LeNet-5 CNN analyses for both dorso-ventral and lateral views. [file 12915_2020_832_MOESM4_ESM.zip › SI FIle 4/Dorsal Analyses/EFourier Data & Results/CVA Results/Log Likelihood Ratio Test (PM).tif]

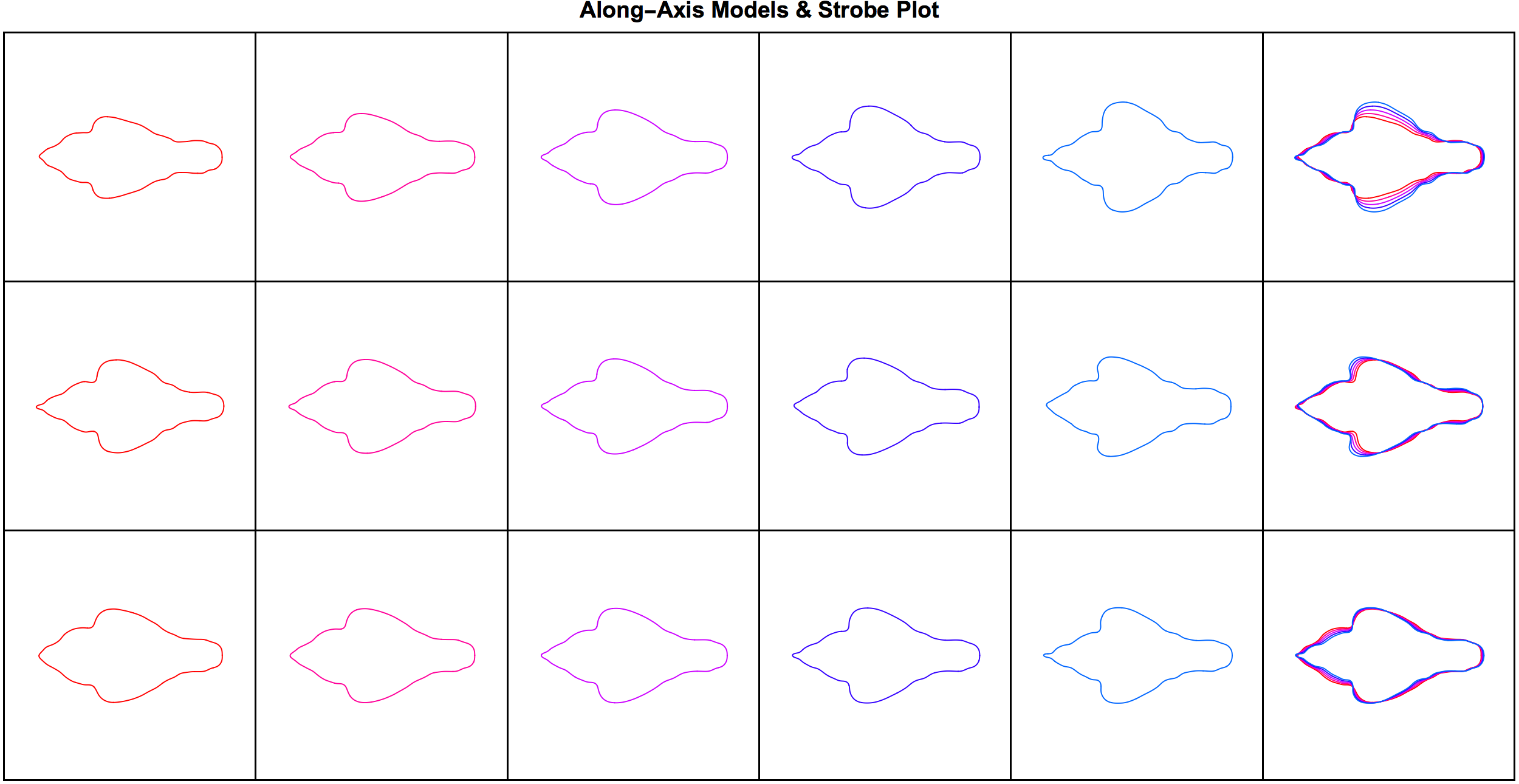

Supplement: Supplementary file 4 — Additional file 4. An archive catalogue of all original datafiles and results output for the EFA, Naïve Bayes and embedded LeNet-5 CNN analyses for both dorso-ventral and lateral views. [file 12915_2020_832_MOESM4_ESM.zip › SI FIle 4/Dorsal Analyses/EFourier Data & Results/PCA Results/Along-Axis Shape Models (Grid).tif]

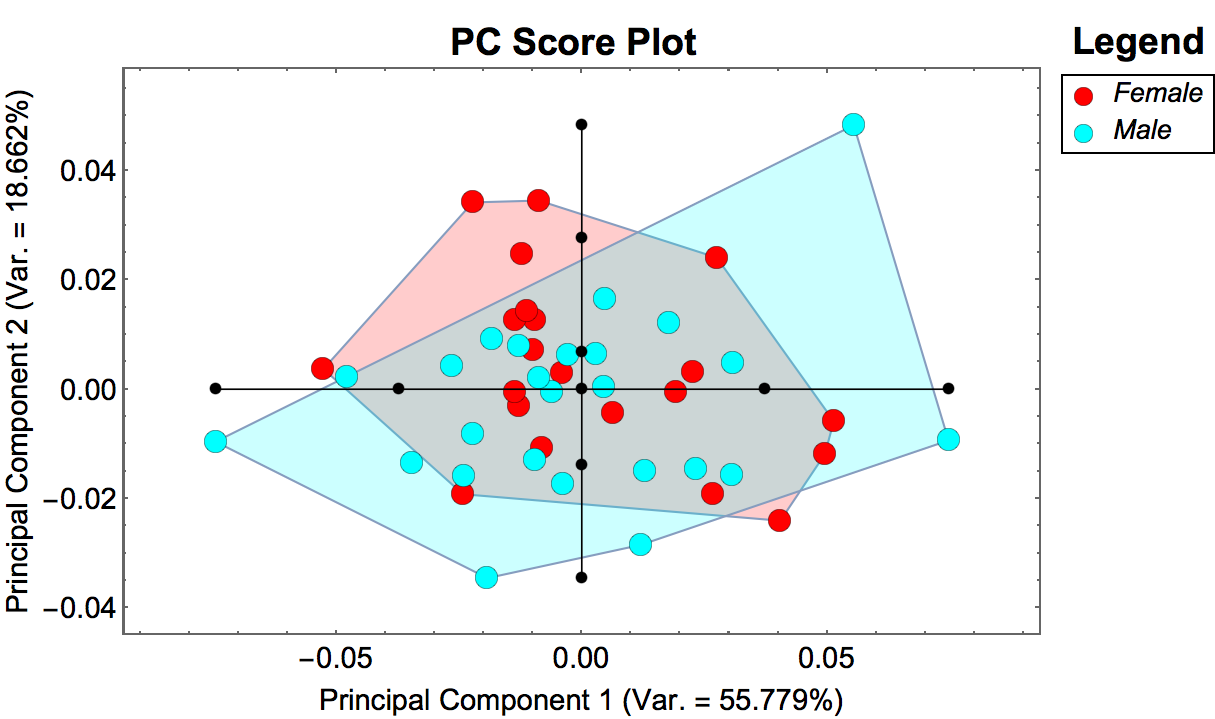

Supplement: Supplementary file 4 — Additional file 4. An archive catalogue of all original datafiles and results output for the EFA, Naïve Bayes and embedded LeNet-5 CNN analyses for both dorso-ventral and lateral views. [file 12915_2020_832_MOESM4_ESM.zip › SI FIle 4/Dorsal Analyses/EFourier Data & Results/PCA Results/PC-1 vs PC-2 (w: Model Coords).tif]

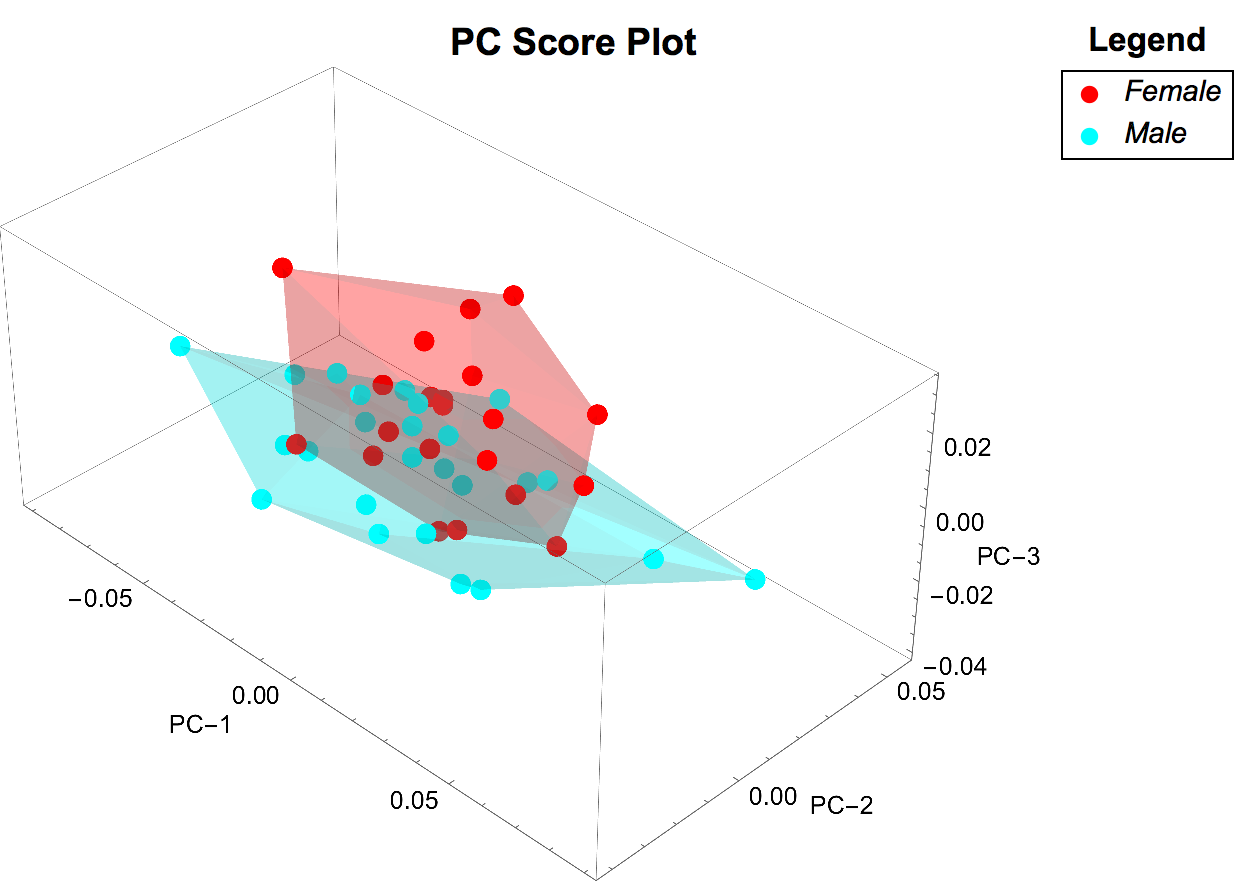

Supplement: Supplementary file 4 — Additional file 4. An archive catalogue of all original datafiles and results output for the EFA, Naïve Bayes and embedded LeNet-5 CNN analyses for both dorso-ventral and lateral views. [file 12915_2020_832_MOESM4_ESM.zip › SI FIle 4/Dorsal Analyses/EFourier Data & Results/PCA Results/PC-1 vs PC-2 vs PC-3.tif]

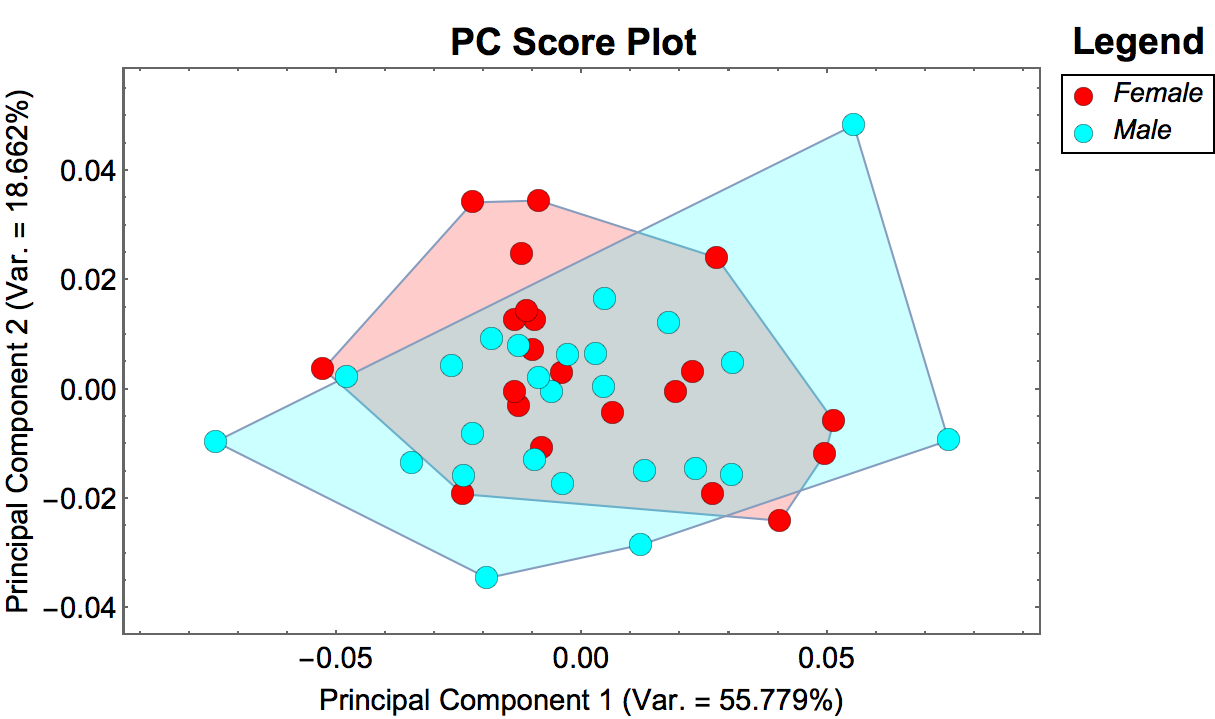

Supplement: Supplementary file 4 — Additional file 4. An archive catalogue of all original datafiles and results output for the EFA, Naïve Bayes and embedded LeNet-5 CNN analyses for both dorso-ventral and lateral views. [file 12915_2020_832_MOESM4_ESM.zip › SI FIle 4/Dorsal Analyses/EFourier Data & Results/PCA Results/PC-1 vs PC-2.tif]

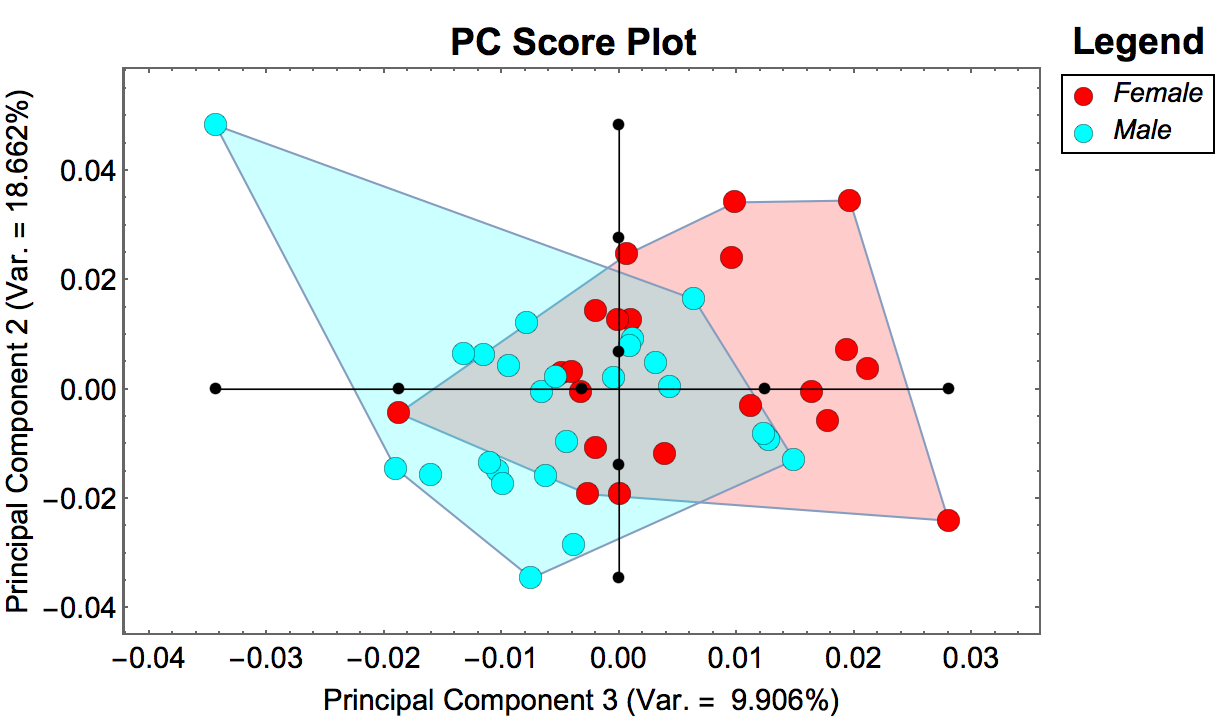

Supplement: Supplementary file 4 — Additional file 4. An archive catalogue of all original datafiles and results output for the EFA, Naïve Bayes and embedded LeNet-5 CNN analyses for both dorso-ventral and lateral views. [file 12915_2020_832_MOESM4_ESM.zip › SI FIle 4/Dorsal Analyses/EFourier Data & Results/PCA Results/PC-3 vs PC-2 (w: Model Coords).tif]

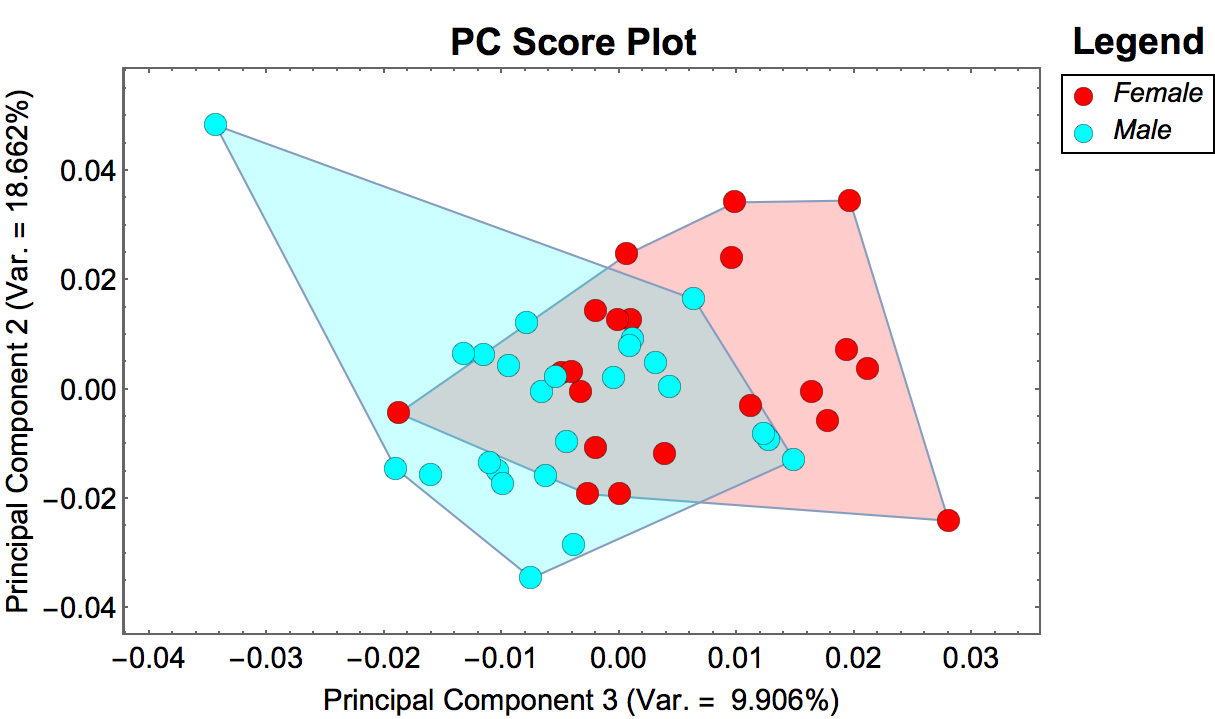

Supplement: Supplementary file 4 — Additional file 4. An archive catalogue of all original datafiles and results output for the EFA, Naïve Bayes and embedded LeNet-5 CNN analyses for both dorso-ventral and lateral views. [file 12915_2020_832_MOESM4_ESM.zip › SI FIle 4/Dorsal Analyses/EFourier Data & Results/PCA Results/PC-3 vs PC-2.tif]

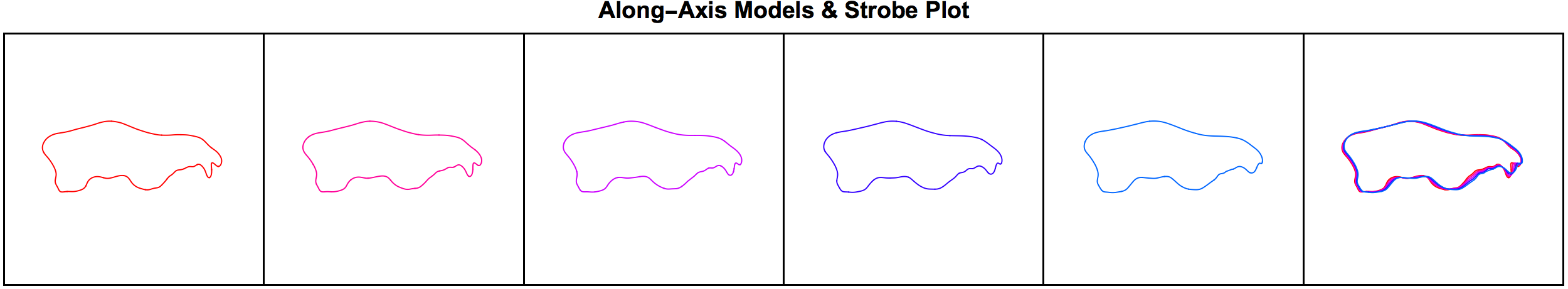

Supplement: Supplementary file 4 — Additional file 4. An archive catalogue of all original datafiles and results output for the EFA, Naïve Bayes and embedded LeNet-5 CNN analyses for both dorso-ventral and lateral views. [file 12915_2020_832_MOESM4_ESM.zip › SI FIle 4/Lateral Analyses/EFourier Data & Results/CVA (of PCA Scores)/Along-Axis Shape Models (Grid).tif]

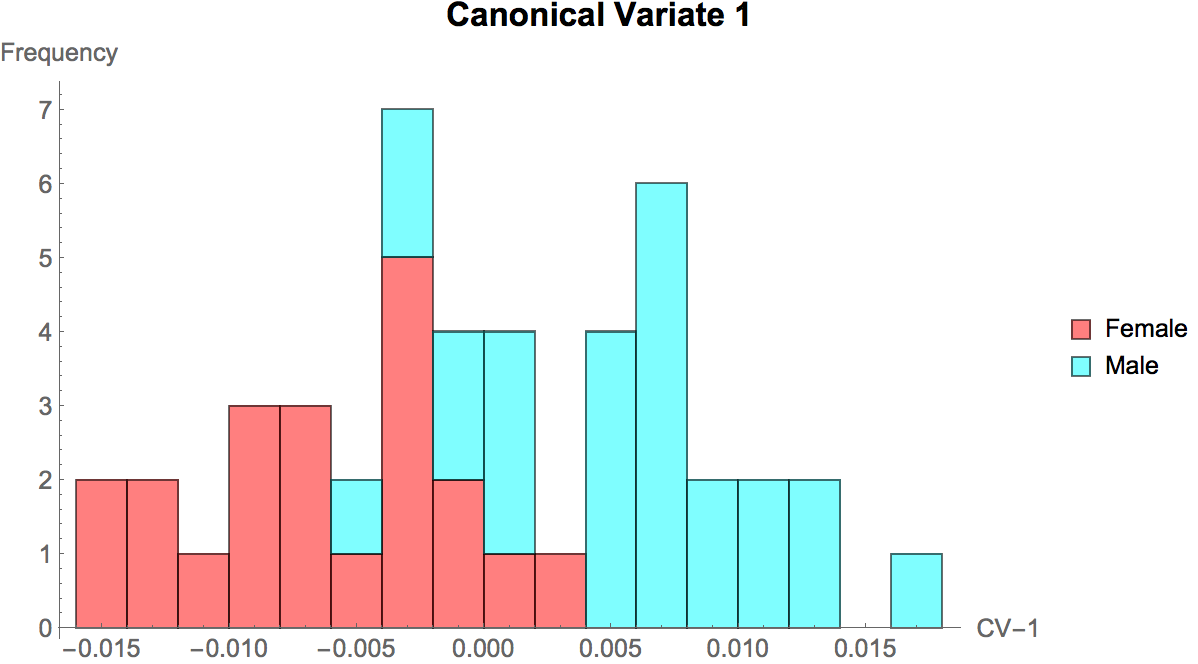

Supplement: Supplementary file 4 — Additional file 4. An archive catalogue of all original datafiles and results output for the EFA, Naïve Bayes and embedded LeNet-5 CNN analyses for both dorso-ventral and lateral views. [file 12915_2020_832_MOESM4_ESM.zip › SI FIle 4/Lateral Analyses/EFourier Data & Results/CVA (of PCA Scores)/CV-1 Histogram.tif]

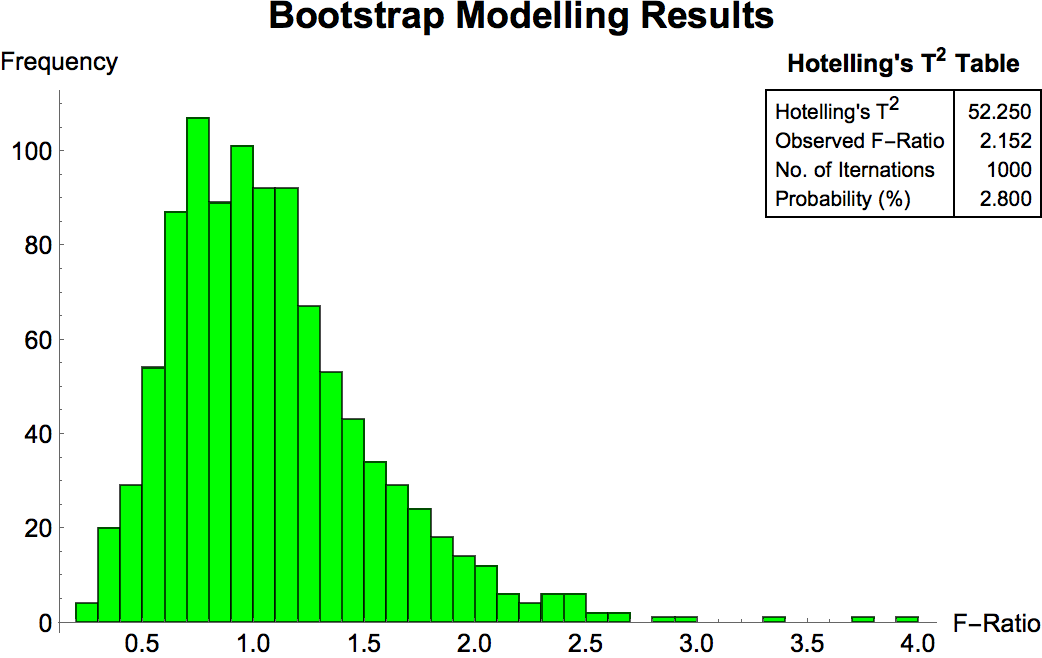

Supplement: Supplementary file 4 — Additional file 4. An archive catalogue of all original datafiles and results output for the EFA, Naïve Bayes and embedded LeNet-5 CNN analyses for both dorso-ventral and lateral views. [file 12915_2020_832_MOESM4_ESM.zip › SI FIle 4/Lateral Analyses/EFourier Data & Results/CVA (of PCA Scores)/Mean Vector Test (HT2-BS).tif]

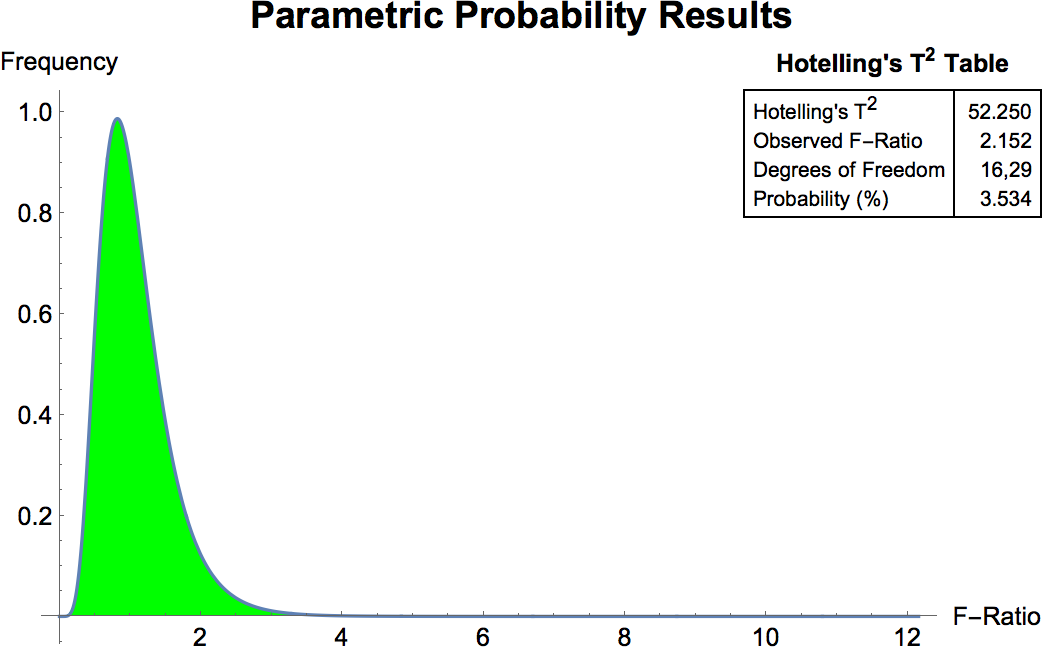

Supplement: Supplementary file 4 — Additional file 4. An archive catalogue of all original datafiles and results output for the EFA, Naïve Bayes and embedded LeNet-5 CNN analyses for both dorso-ventral and lateral views. [file 12915_2020_832_MOESM4_ESM.zip › SI FIle 4/Lateral Analyses/EFourier Data & Results/CVA (of PCA Scores)/Mean Vector Test (HT2-PM).tif]

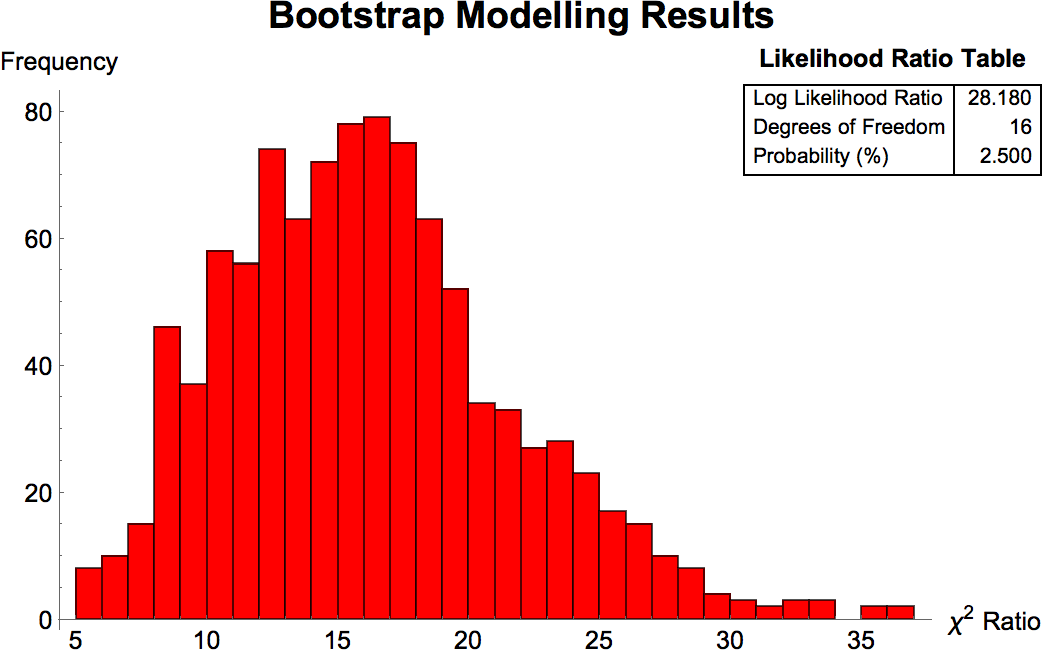

Supplement: Supplementary file 4 — Additional file 4. An archive catalogue of all original datafiles and results output for the EFA, Naïve Bayes and embedded LeNet-5 CNN analyses for both dorso-ventral and lateral views. [file 12915_2020_832_MOESM4_ESM.zip › SI FIle 4/Lateral Analyses/EFourier Data & Results/CVA (of PCA Scores)/Mean Vector Test (LLR-BS).tif]

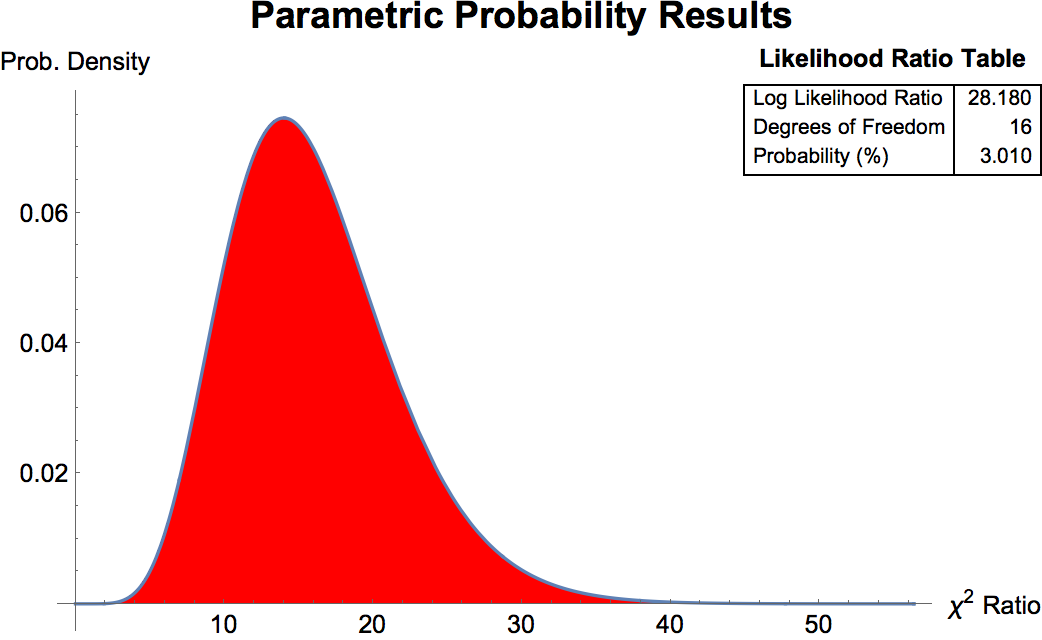

Supplement: Supplementary file 4 — Additional file 4. An archive catalogue of all original datafiles and results output for the EFA, Naïve Bayes and embedded LeNet-5 CNN analyses for both dorso-ventral and lateral views. [file 12915_2020_832_MOESM4_ESM.zip › SI FIle 4/Lateral Analyses/EFourier Data & Results/CVA (of PCA Scores)/Mean Vector Test (LLR-PM).tif]

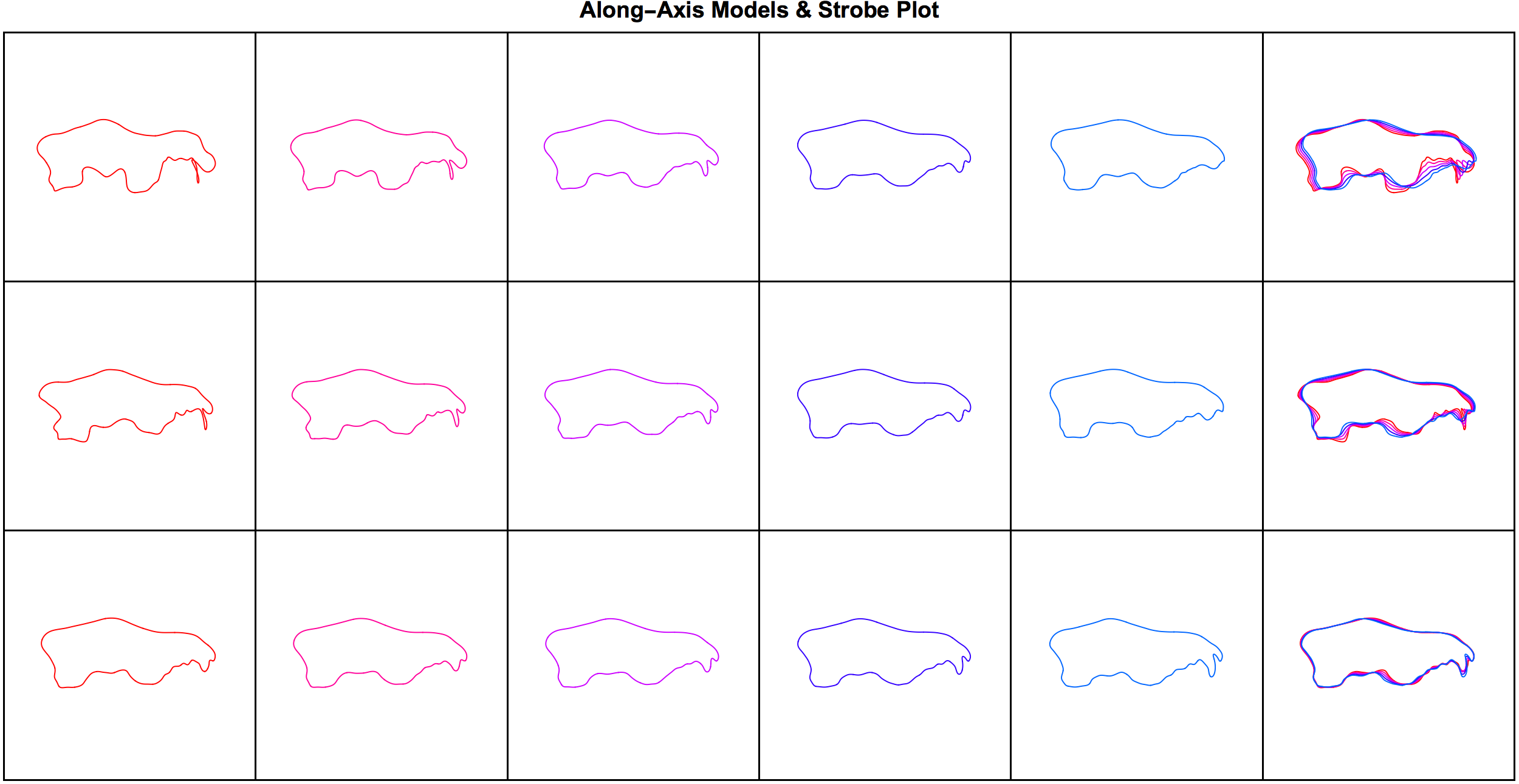

Supplement: Supplementary file 4 — Additional file 4. An archive catalogue of all original datafiles and results output for the EFA, Naïve Bayes and embedded LeNet-5 CNN analyses for both dorso-ventral and lateral views. [file 12915_2020_832_MOESM4_ESM.zip › SI FIle 4/Lateral Analyses/EFourier Data & Results/PCA Results/Along-Axis Shape Models (Grid).tif]

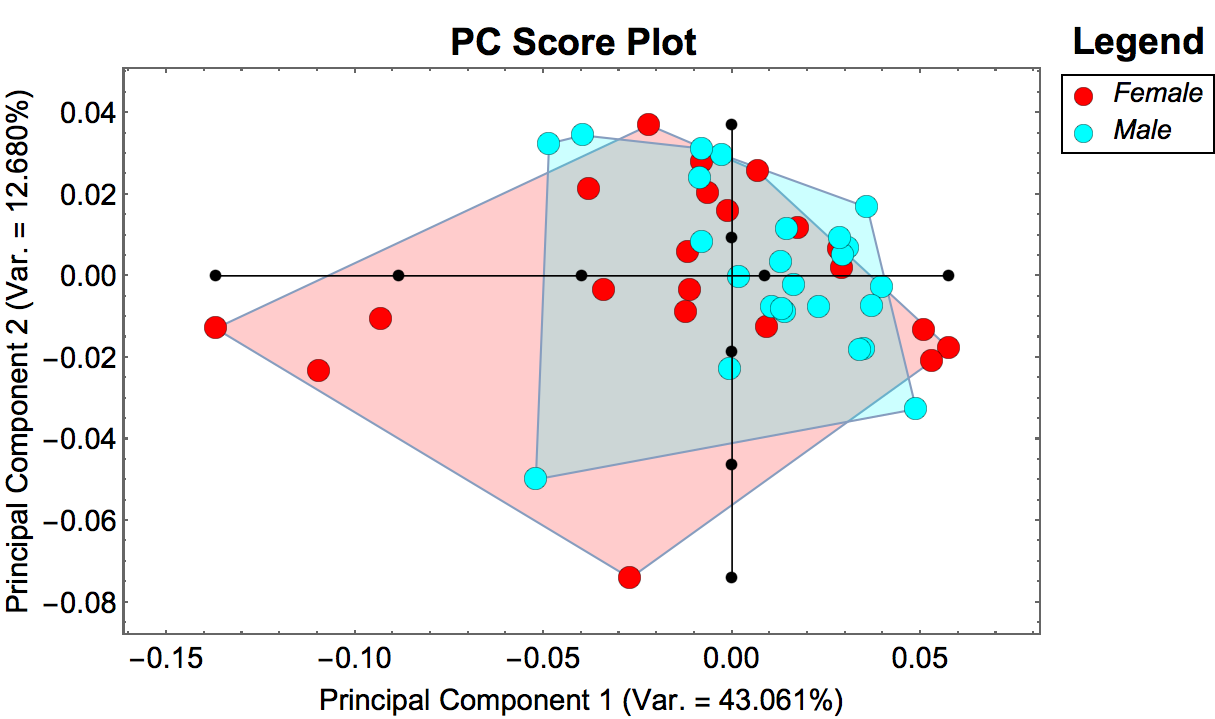

Supplement: Supplementary file 4 — Additional file 4. An archive catalogue of all original datafiles and results output for the EFA, Naïve Bayes and embedded LeNet-5 CNN analyses for both dorso-ventral and lateral views. [file 12915_2020_832_MOESM4_ESM.zip › SI FIle 4/Lateral Analyses/EFourier Data & Results/PCA Results/PC-1 vs PC-2 (w: Model Coords).tif]

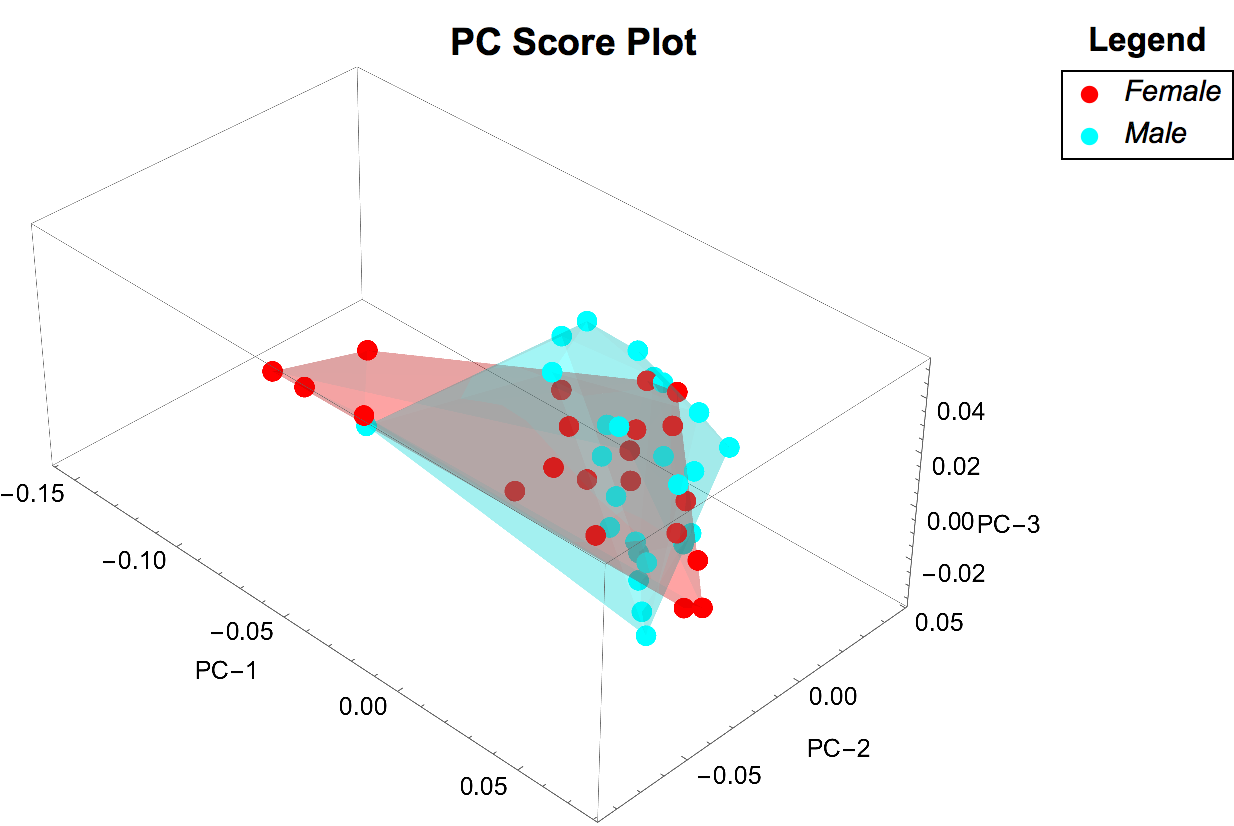

Supplement: Supplementary file 4 — Additional file 4. An archive catalogue of all original datafiles and results output for the EFA, Naïve Bayes and embedded LeNet-5 CNN analyses for both dorso-ventral and lateral views. [file 12915_2020_832_MOESM4_ESM.zip › SI FIle 4/Lateral Analyses/EFourier Data & Results/PCA Results/PC-1 vs PC-2 vs PC-3.tif]

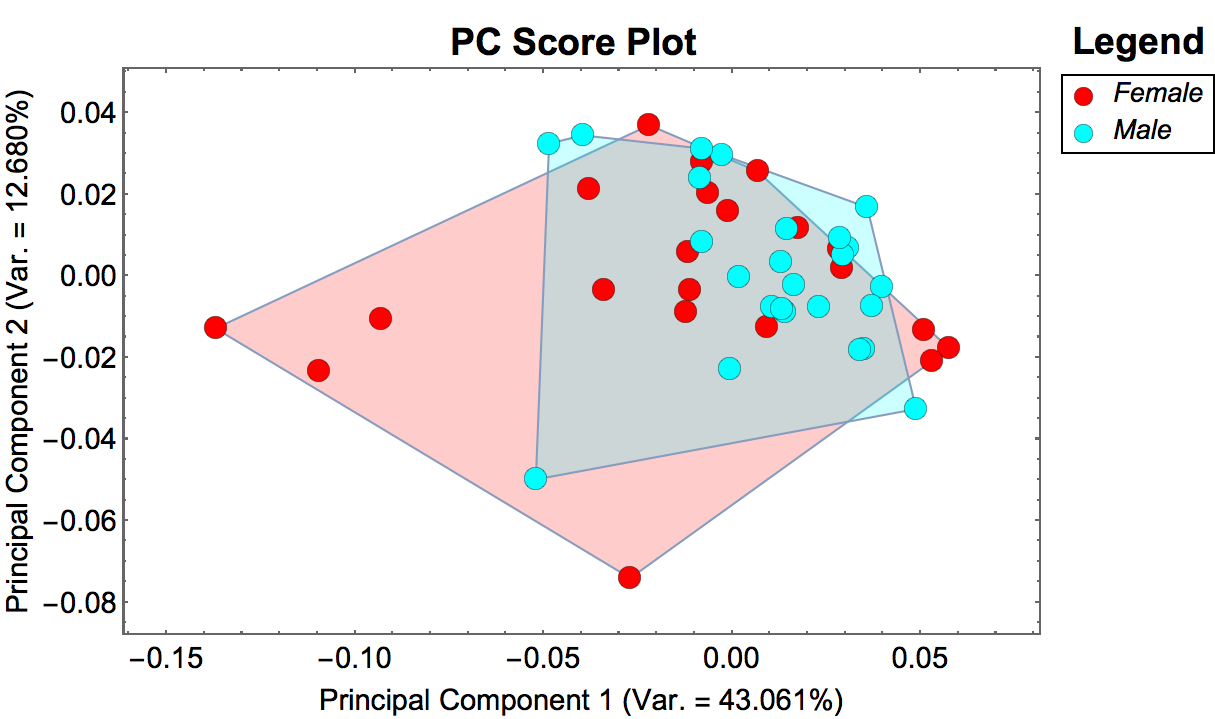

Supplement: Supplementary file 4 — Additional file 4. An archive catalogue of all original datafiles and results output for the EFA, Naïve Bayes and embedded LeNet-5 CNN analyses for both dorso-ventral and lateral views. [file 12915_2020_832_MOESM4_ESM.zip › SI FIle 4/Lateral Analyses/EFourier Data & Results/PCA Results/PC-1 vs PC-2.tif]

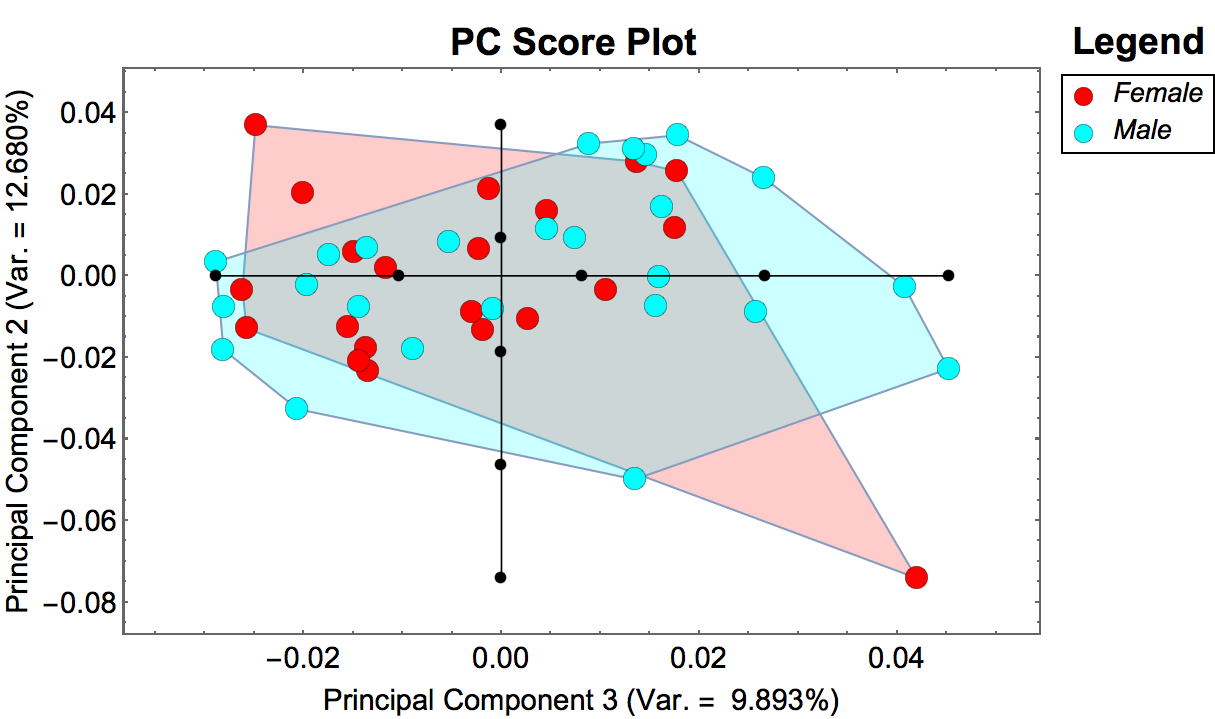

Supplement: Supplementary file 4 — Additional file 4. An archive catalogue of all original datafiles and results output for the EFA, Naïve Bayes and embedded LeNet-5 CNN analyses for both dorso-ventral and lateral views. [file 12915_2020_832_MOESM4_ESM.zip › SI FIle 4/Lateral Analyses/EFourier Data & Results/PCA Results/PC-3 vs PC-2 (w: Model Coords).tif]

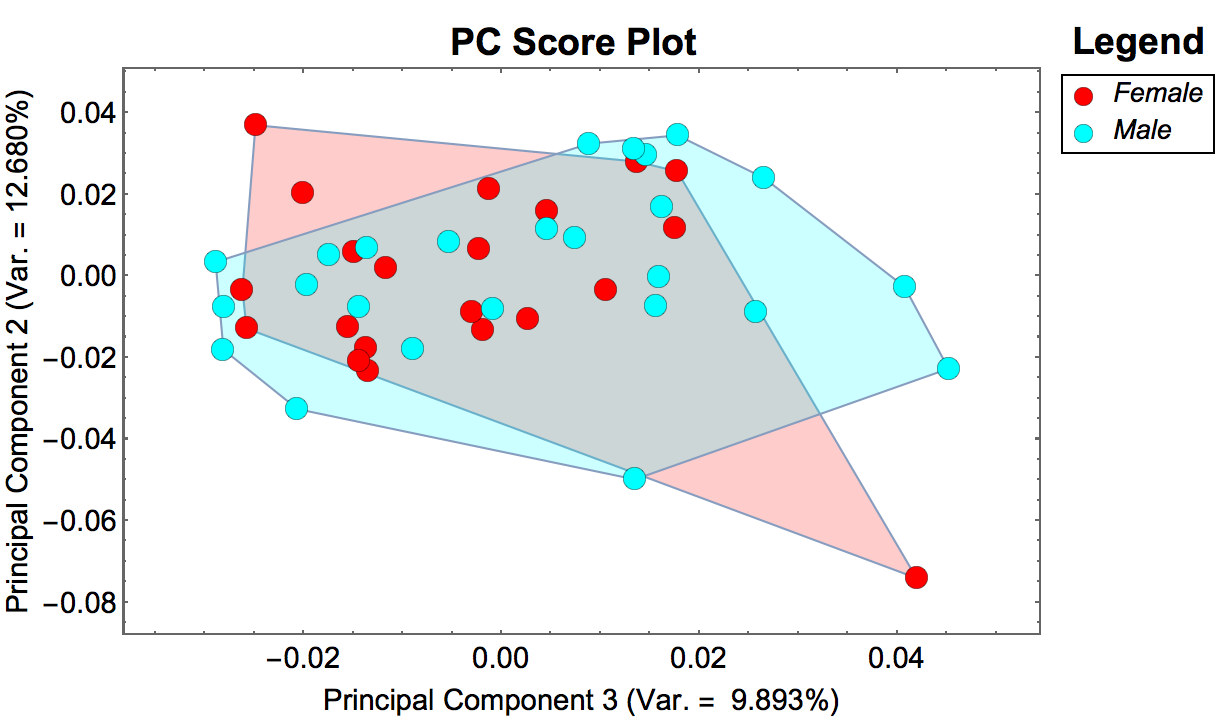

Supplement: Supplementary file 4 — Additional file 4. An archive catalogue of all original datafiles and results output for the EFA, Naïve Bayes and embedded LeNet-5 CNN analyses for both dorso-ventral and lateral views. [file 12915_2020_832_MOESM4_ESM.zip › SI FIle 4/Lateral Analyses/EFourier Data & Results/PCA Results/PC-3 vs PC-2.tif]
