# Supplementary material for: Machine-learning strategies for testing patterns of morphological variation in small samples: sexual dimorphism in gray wolf (Canis lupus) crania
Source: BMC Biol. 2020 Sep 3;18:113. doi: 10.1186/s12915-020-00832-1 (PMC7470621; doi:10.1186/s12915-020-00832-1)
Supplement: Supplementary file 5 — Additional file 5. An archive of all code listings for all data procession/analysis software employed in this investigation. [file 12915_2020_832_MOESM5_ESM.zip › SI File 5/CVA (vers. 1.40).pdf]

Canonical Variates Analysis

This program accepts a data matrix in standard format,
performs a CVA on the data columns, and allows the result to be viewed
as 2 D or 3 D (interactive) scatterplots. Full support is provided for tracking groups
that have been identified in the datafile.

Author : N. MacLeod
Version : 1.40
Date : 8 November 2019
Reference : MacLeod (2007)

Initialize libraries

In[∘]:=
```
<< ComputationalGeometry`
```

Read in data file & partition into datasets.

In[∘]:=
```
filenamein = SystemDialogInput["FileOpen"];
x1 = Import[filenamein, "CSV"];
filenamein

{n1, m1} = Dimensions[x1];

varNames = Flatten[Take[x1, 1]];
x2 = Drop[x1, 1];
varNames = Drop[varNames, 1];
varNames = Drop[varNames, 1];

objNames = Flatten[Take[x2, n1 - 1, 1]];
x2 = Drop[x2, 0, 1];

Group = Flatten[Take[x2, n1 - 1, 1]];
x2 = Drop[x2, 0, 1];
numGroups = Length[Union[Group]];

{n2, m2} = Dimensions[x2];

Print["No. of Objects: ", n2];
Print["No. of Variables: ", m2];
Print["No. of Groups: ", Length[Union[Group]]];
```

Data transformations (optional)

Specify global data transformations (if any).
Note : Remember you cannot subsequently take the logarithm of mean –

centered or standardized data. If you wish
to perform such an analysis you must shift the mean –
centered or standardized data by a constant (e.g., 1, 10)

In[∘]:=
```
Panel[Labeled[Column[
  {Row[{Panel[Labeled[PopupMenu[Dynamic[meanTrans], {1 → "No", 2 → "Yes"}],
       "Mean center data?", Top, LabelStyle →
        Directive[FontSize → 12, Bold, FontFamily → "Arial"]]], "  ",
     Panel[Labeled[PopupMenu[Dynamic[logTrans], {1 → "No", 2 → "Yes"}],
       "Log10-trasform data?", Top, LabelStyle →
        Directive[FontSize → 12, Bold, FontFamily → "Arial"]]], "  ",
     Panel[Labeled[PopupMenu[Dynamic[stdTrans], {1 → "No", 2 → "Yes"}],
       "Standardize data?", Top,
       LabelStyle → Directive[FontSize → 12, Bold, FontFamily → "Arial"]]]}],
   Row[{Panel[Labeled[PopupMenu[Dynamic[shiftTrans], {1 → "No", 2 → "Yes"}],
       "Shift data by a constant?", Top, LabelStyle →
        Directive[FontSize → 12, Bold, FontFamily → "Arial"]]], "  ",
     Panel[Labeled[InputField[Dynamic[knsnt], FieldSize → 5],
       "Enter shift constant value.", Top,
       LabelStyle → Directive[FontSize → 12, Bold, FontFamily → "Arial"]]]}]},
  Center], "Global Data Transform Options", Top, LabelStyle →
  Directive[FontSize → 18, Bold, FontFamily → "Arial"]]]
meanTrans = 1; logTrans = 1; stdTrans = 1; shiftTrans = 1; knsnt = 10;
```

Out[∘]=
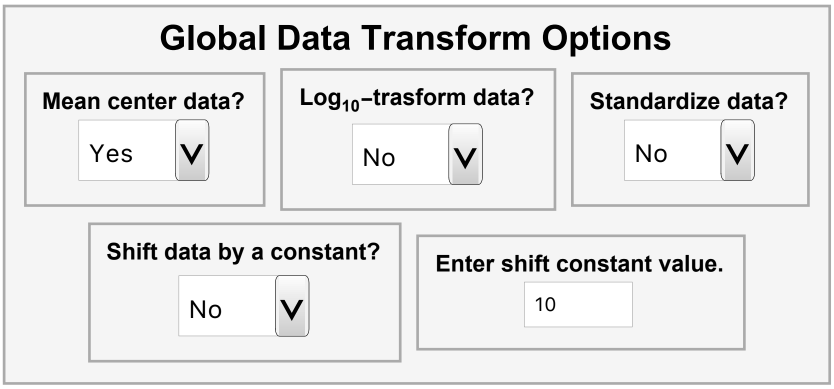

Perform global data transformation (optional)

In[∘]:=
```
If[meanTrans == 2, mVec = N[Mean[x2]];
  Do[x2[[i]] = x2[[i]] - mVec, {i, n2}]];
If[logTrans == 2, x2 = N[Log10[x2]]];
If[stdTrans == 2, x2 = Standardize[x2]];
If[shiftTrans == 2, x2 = x2 * knsnt];
```

Export mean vector (optional; may be useful in modelling).

```
filenameout = SystemDialogInput["FileSave"];
Export[filenameout, mVec, "csv"]
```

Export processed dataset (optional; may be useful in other analyses).

```
x2Trans = Table[" ", {n2 + 1}, {m2 + 2}];
x2Trans[[1, 1]] = "Object";
x2Trans[[1, 2]] = "Group";
Do[x2Trans[[1, j + 2]] = varNames[[j]], {j, m2}]
Do[x2Trans[[i + 1, 1]] = objNames[[i]], {i, n2}]
Do[x2Trans[[i + 1, 2]] = Group[[i]], {i, n2}]
Do[x2Trans[[i + 1, j + 2]] = x2[[i, j]], {i, n2}, {j, m2}]

filenameout = SystemDialogInput["FileSave"];
Export[filenameout, x2Trans, "CSV", "TextDelimiters" → ""]
```

Choose eigenanalysis method

```
In[∘]:= Panel[Labeled[PopupMenu[Dynamic[eMethod],
     {1 → "Std. Eigenanalysis", 2 → "Singular Value Decomposition"}],
   "Choose eigenanalysis method", Top,
   LabelStyle → Directive[FontSize → 12, Bold, FontFamily → "Arial"]]]
```

Out[∘]=

Choose eigenanalysis method

Std. Eigenanalysis ∨

Perform eigenanalysis

```
In[∘]:= pltTable = Table[" ", {numGroups}, {3}];
{gndMean, T, gNames, nGps, gpMeans, smpSize, W, B} = TWB[x2, n2, m2, Group];
kg = Length[Union[Group]];
WI = Inverse[W];
sCovar = WI.B;
If[n2 > m2, mvecs = m2, mvecs = n2];
If[n2 > m2, mvecs = m2, mvecs = n2];
If[eMethod == 1,
  eVals = Eigenvalues[sCovar];
  w = Eigenvectors[sCovar];
  eVecs = Transpose[w];
  eScores = x2.eVecs;
  eVecsTotal = eVecs];
If[eMethod == 2,
  {w, u, v} = SingularValueDecomposition[sCovar, mvecs];
  eVals = N[Diagonal[u], kg - 1];
```

```
    eVecs = Take[Transpose[w], mvecs];
    eScores = x2.w;
    eVecsTotal = eVecs];
eS2 = eScores;

noVals = Min[{nGps - 1, m2}];
noAxes = noVals;
eVals = Take[eVals, noVals];
s1 = Total[eVals];
evalTable = Table[0.0, {i, kg - 1}, {j, 4}];
Do[evalTable[[i, 1]] = i, {i, 1, kg - 1}];
Do[evalTable[[i, 2]] = eVals[[i]], {i, 1, kg - 1}];
Do[evalTable[[i, 3]] = eVals[[i]] 100.` / s1, {i, 1, kg - 1}];
Do[evalTable[[i, 4]] = evalTable[[i, 3]], {i, 1, kg - 1}];
Do[evalTable[[i, 4]] = evalTable[[i - 1, 4]] + evalTable[[i, 3]], {i, 2, kg - 1}];

tabHeads =
    {"Component", " Eigenvalue", "  Variance (%)", "  Cum. Variance (%)"};
t2 = Partition[Flatten[Join[tabHeads, evalTable]], 4];
t3 = Partition[Flatten[t2], 4];

Do[
  Do[
    t3[[i, j]] = PaddedForm[t3[[i, j]], {5, 3}], {i, 2, kg - 1 + 1}], {j, 2, 4}]

Labeled[Grid[t3, BaseStyle → (FontFamily → "Arial"),
    Alignment → {{Center, Right, Right, Right}},
    Frame → True, Dividers → {{True, True}, {True, True}}],
  "Eigenvalue Table", Top, LabelStyle → (FontFamily → "Arial")]
```

Display CVA Results (optional).

Display CV eigenvector loadings (optional).

```
In[•]:= evecTable = Table[0.0, {i, m2 + 1}, {j, noAxes + 1}];
Do[evecTable[[i + 1, 1]] = varNames[[i]], {i, m2}]
evecTable[[1, 1]] = "Variable";
Do[evecTable[[1, i + 1]] = StringJoin["CV-", ToString[i]], {i, noAxes}]
Do[Do[evecTable[[i + 1, j + 1]] = PaddedForm[eVecs[[i, j]], {5, 3}], {i, m2}],
 {j, noAxes}]
Labeled[Grid[evecTable, BaseStyle → (FontFamily → "Arial"),
  Alignment → {{Center, Right, Right, Right, Right, Right}}, Frame → True,
  Dividers → {{True, True}, {True, True}}], "Eigenvector Table", Top,
 LabelStyle → Directive[Black, Bold, FontSize → 14, FontFamily → "Arial"]]
```

Display CV scores (optional).

```
In[•]:= scrTable = Table[0.0, {i, n2 + 1}, {j, noAxes + 2}];
Do[scrTable[[i + 1, 1]] = objNames[[i]], {i, n2}]
scrTable[[1, 1]] = "Objects";
scrTable[[1, 2]] = "Groups";
Do[scrTable[[1, i + 2]] = StringJoin["CV-", ToString[i]], {i, noAxes}]
Do[scrTable[[i + 1, 2]] = Group[[i]], {i, n2}]
Do[scrTable[[i + 1, j + 2]] = PaddedForm[eScores[[i, j]], {5, 3}],
 {i, n2}, {j, noAxes}]
Labeled[Grid[scrTable, BaseStyle → (FontFamily → "Arial"),
  Alignment → {{Left, Left, Right, Right, Right, Right}}, Frame → True,
  Dividers → {{True, True, True}, {True, True}}], "Eigenscore Table", Top,
 LabelStyle → Directive[Black, Bold, FontSize → 14, FontFamily → "Arial"]]
```

Plot single – axis histogram.

Specify single – variable plot (histogram) options.
You must run this code after you read in the data so it can pick up the proper variable names.

In[∘]:=
```
cvNames = Table[StringJoin["CV-", ToString[i]], {i, noAxes}];
Panel[Labeled[Column[{
    Row[{Panel[Labeled[PopupMenu[Dynamic[axisName], cvNames],
        "Select variable to be plotted on x-Axis.", Top,
        LabelStyle → Directive[FontSize → 12, Bold, FontFamily → "Arial"]]],
      "  ", Panel[Labeled[PopupMenu[Dynamic[histType],
         {1 → "Stacked", 2 → "Overlapped"}], "Select histogram type.", Top,
        LabelStyle → Directive[FontSize → 12, Bold, FontFamily → "Arial"]]]}],
    Row[{Panel[Labeled[InputField[Dynamic[noBins], FieldSize → 5],
        "Enter no. of histogram bins.", Top, LabelStyle →
         Directive[FontSize → 12, Bold, FontFamily → "Arial"]]], "  ",
      Panel[Labeled[InputField[Dynamic[hSize], FieldSize → 5],
        "Enter histogram plot size.", Top,
        LabelStyle → Directive[FontSize → 12, Bold, FontFamily → "Arial"]]]}]},
   Center], "Single Axis (Histogram) Plot Options", Top,
  LabelStyle → Directive[FontSize → 16, Bold, FontFamily → "Arial"]]]
axisName = cvNames[[1]]; noBins = 15; hSize = 500; histType = 1;
```

··· Table: Iterator {i, noAxes} does not have appropriate bounds.

Out[∘]=

Single Axis (Histogram) Plot Options

Select variable to be plotted on x-Axis.
PopupMenu[PC−1, Table[CV− <> ToString[i], {i, noAxes}]]

Select histogram type.
Stacked

Enter no. of histogram bins.
15

Enter histogram plot size.
500

Constrruct and display histogram plot .

In[•]:=
```
Do[If[axisName == cvNames[[j]], axis = j], {j, noAxes}];
pltScores = Flatten[Take[eScores, All, {axis}]];
If[histType == 1, htype = "Stacked", htype = "Overlapped"];
gpNames = Union[Group];
groupPosns = Table[Flatten[Position[Group, gpNames[[i]], 1]], {i, numGroups}];
pltPoints = Table[pltScores[[groupPosns[[j]]]], {j, numGroups}];
hueList = Table[Hue[N[((numGroups + 1) - j) / numGroups]], {j, numGroups}];
h1 =
 Labeled[Histogram[pltPoints, noBins, ChartStyle → {hueList}, ChartLayout → htype,
   LabelStyle → Directive[FontSize → 12, FontFamily → "Arial"],
   AxesLabel → {StringJoin["CV-", ToString[axis]], "Frequency"},
   ImageSize → hSize, ChartLegends → gpNames],
  StringJoin["Canonical Variate ", ToString[axis]], Top,
  LabelStyle → Directive[FontSize → 16, Bold, FontFamily → "Arial"]]
```

Export current histogram.

In[•]:=
```
filenameout = SystemDialogInput["FileSave"];
Export[filenameout, h1, "TIFF", ImageResolution → 150]
```

Estimate statistical significance of group centroid separations.

Test Two – Group Separation w/ Hotelling's T^2 statistic.

Specify testing options

Standard (F – ratio) Distribution =

use this test if your data are normally distributed and the group covariance matrices are equal

Monte Carlo Simulated Distribution =

use this test if your data are not normally distributed and/or the group covariance matrices are not equal

Bootstrap Simulated Distribution =

use this test if your data are not normally distributed and/or the group covariance matrices are not equal

In[∘]:=
```
Panel[
 Labeled[Column[{Row[{Panel[Labeled[InputField[Dynamic[hSize2], FieldSize → 10],
        "Enter histogram size (in pixels).", Top,
        LabelStyle → Directive[FontSize → 12, Bold, FontFamily → "Arial"]]],
      " ", Panel[Labeled[InputField[Dynamic[m2], FieldSize → 10],
        "Enter number of variables.", Top, LabelStyle →
         Directive[FontSize → 12, Bold, FontFamily → "Arial"]]], " ",
      Panel[Labeled[InputField[Dynamic[n2], FieldSize → 10],
        "Enter number of objects.", Top,
        LabelStyle → Directive[FontSize → 12, Bold, FontFamily → "Arial"]]]}],
    Row[{Panel[Labeled[InputField[Dynamic[iterT2], FieldSize → 10],
        "No. of replicate iterations (Monte Carlo or bootstrapped only).",
        Top, LabelStyle → Directive[FontSize → 12,
          Bold, FontFamily → "Arial"]]], " ",
      Panel[Labeled[PopupMenu[Dynamic[statTestT2],
         {1 → "Standard Parametric (F-ratio) Distributions",
          2 → "Monte Carlo Simulated (F-ratio) Distributions ",
          3 → "Bootstrapped Modelled (F-ratio) Distributions"}],
        "Choose estimation procedure.", Top, LabelStyle →
         Directive[FontSize → 12, Bold, FontFamily → "Arial"]]]}]}, Center],
  "Hotelling's T^2 Mean Vector Test Control Parameters",
  Top,
  LabelStyle →
   Directive[FontSize → 14,
    Bold, FontFamily → "Arial"]]]
hSize2 = 500; nobins = 25; iterT2 = 1000; statTestT2 = 1;
```

Out[∘]=

Hotelling's T^2 Mean Vector Test Control Parameters

Enter histogram size (in pixels).	Enter number of variables.	Enter number of objects.
hSize2	5400	25

No. of replicate iterations (Monte Carlo or bootstrapped only).

iterT2

Choose estimation procedure.

Standard Parametric (F–ratio) Distributions ∨

Calculate and display probability statistics

In[∘]:=
```
groupNames = Union[Group];
numGroups = Length[groupNames];
groupPosns =
```

```
  Table[Flatten[Position[Group, groupNames[[i]], 1]], {i, numGroups}];
groups = N[Table[x2[[groupPosns[[j]]]], {j, numGroups}]];
{gpn1, m2} = Dimensions[groups[[1]]];
{gpn2, m2} = Dimensions[groups[[2]]];
c1 = Covariance[groups[[1]]];
c2 = Covariance[groups[[2]]];
c = (((gpn1 - 1) * c1) + ((gpn2 - 1) * c2)) / (gpn1 + gpn2 - 2);
cinv = PseudoInverse[c];
meanG1 = Mean[groups[[1]]];
meanG2 = Mean[groups[[2]]];
t1 = gpn1 * gpn2;
t2 = meanG1 - meanG2;
t4 = meanG1 - meanG2;
t5 = gpn1 + gpn2;
T2 = ((t1 * t2).cinv.t4) / t5;
FT2 = (gpn1 + gpn2 - m2 - 1) * T2 / ((gpn1 + gpn2 - 2) * m2);
num = m2;
denom = gpn1 + gpn2 - m2 - 1;
probT2 = N[(1 - CDF[FRatioDistribution[num, denom], FT2])];
pcentProbT2 = probT2 * 100;

If[statTestT2 == 1,
  FTable = Table[0, {4}, {2}];
  FTable[[1, 1]] = "Hotelling's T^2";
  FTable[[2, 1]] = "Observed F-Ratio";
  FTable[[3, 1]] = "Degrees of Freedom";
  FTable[[4, 1]] = "Probability (%)";
  FTable[[1, 2]] = PaddedForm[T2, {4, 3}];
  FTable[[2, 2]] = PaddedForm[FT2, {5, 3}];
  FTable[[3, 2]] =
   StringJoin[ToString[Round[num]], ",", ToString[Round[denom]]];
  FTable[[4, 2]] = PaddedForm[pcentProbT2, {5, 3}];
  If[pcentProbT2 ≤ 5.0, hcolor = Green, hcolor = Red];
  hLegend = Labeled[
    Grid[FTable, BaseStyle → Directive[FontSize → 10, FontFamily → "Arial"],
     Alignment → {{Left, Right}}, Frame → True,
     Dividers → {{True, True}, {True}}], "Hotelling's T^2 Table", Top,
    LabelStyle → Directive[FontSize → 12, Bold, FontFamily → "Arial"]];
  plotT2 = Plot[PDF[FRatioDistribution[num, denom], x] // Evaluate,
    {x, 0, FT2 + 10}, PlotRange → Full, Filling → Axis, Exclusions → None,
    ImageSize → hSize2, Filling → Bottom, FillingStyle → hcolor,
    PlotRange → Full, AxesLabel → {"F-Ratio", "Frequency"},
    LabelStyle → Directive[Black, FontSize → 12, FontFamily → "Arial"]]];

Panel[
 Labeled[ProgressIndicator[Dynamic[it], {1, iterT2}], "Calculation Progress",
  Top, LabelStyle → Directive[FontSize → 12, Bold, FontFamily → "Arial"]]]
```

```
If[statTestT2 > 1,
  avg = Mean[x2];
  stDev = N[StandardDeviation[x2]];
  groupNames = Union[Group];
  numGroups = Length[groupNames];
  groupPosns =
   Table[Flatten[Position[Group, groupNames[[i]], 1]], {i, numGroups}];
  x3 = Table[0.0, {n1}, {m1}];
  simFRatioT2 = Table[0.0, {iterT2}];

  Do[
   If[statTestT2 == 2,
    Do[
     Do[
      Do[
       in = groupPosns[[k, i]];
       x3[[in, j]] = RandomReal[NormalDistribution[avg[[j]], stDev[[j]]]],
       {j, m1}], {i, smpSize[[k]]}], {k, kg}]];
   If[statTestT2 == 3,
    x3 = x2;
    Do[x3[[i]] = N[x2[[RandomInteger[{1, n2}]]]], {i, n2}]];
   simgp1 = Table[x3[[groupPosns[[j]]]], {j, numGroups}];
   {simgpn1, m2} = Dimensions[simgp1[[1]]];
   {simgpn2, m2} = Dimensions[simgp1[[2]]];
   simc1 = Covariance[simgp1[[1]]];
   simc2 = Covariance[simgp1[[2]]];
   simc =
    (((simgpn1 - 1) * simc1) + ((simgpn2 - 1) * simc2)) / (simgpn1 + simgpn2 - 2);
   simcinv = PseudoInverse[simc];
   simmeanG1 = Mean[simgp1[[1]]];
   simmeanG2 = Mean[simgp1[[2]]];
   simt1 = simgpn1 * simgpn2;
   simt2 = simmeanG1 - simmeanG2;
   simt4 = simmeanG1 - simmeanG2;
   simt5 = simgpn1 + simgpn2;
   simT2 = ((simt1 * simt2).simcinv.simt4) / simt5;
   simFT2 = (simgpn1 + simgpn2 - m2 - 1) * simT2 / ((simgpn1 + simgpn2 - 2) * m2);
   simFRatioT2[[it]] = simFT2, {it, iterT2}];

  simFRatioT2 = Sort[simFRatioT2];
  knt = 0;
  Do[If[simFRatioT2[[i]] > FT2, knt = knt + 1], {i, iterT2}];
  simProbT2 = (N[knt / iterT2]) * 100.0;

  FTable = Table[0, {5}, {2}];
  FTable[[1, 1]] = "Hotelling's T^2";
```

```
  FTable[[2, 1]] = "Observed F-Ratio";
  FTable[[3, 1]] = "Degrees of Freedom";
  FTable[[4, 1]] = "No. of Iterations";
  FTable[[5, 1]] = "Probability (%)";
  FTable[[1, 2]] = PaddedForm[T2, {4, 3}];
  FTable[[2, 2]] = PaddedForm[FT2, {5, 3}];
  FTable[[3, 2]] =
   StringJoin[ToString[Round[num]], ",", ToString[Round[denom]]];
  FTable[[4, 2]] = iterT2;
  FTable[[5, 2]] = PaddedForm[simProbT2, {5, 3}];
  If[simProbT2 ≤ 5.0, hcolor = Green, hcolor = Red];
  hLegend = Labeled[
    Grid[FTable, BaseStyle → Directive[FontSize → 10, FontFamily → "Arial"],
     Alignment → {{Left, Right}}, Frame → True,
     Dividers → {{True, True}, {True}}], "Hotelling's T^2 Table", Top,
    LabelStyle → Directive[FontSize → 12, Bold, FontFamily → "Arial"]];
  plotT2 = Histogram[simFRatioT2, "FreedmanDiaconis",
    ChartStyle → hcolor, ImageSize → hSize2, PlotRange → Full,
    LabelStyle → Directive[FontSize → 12, Black, FontFamily → "Arial"],
    AxesLabel → {"F-Ratio", "Frequency"}, ImageSize → hSize]];

label1 = "Parametric Probability Results";
label2 = "Monte Carlo Simulation Results";
label3 = "Bootstrap Modelling Results";
probPlot1 = Labeled[Overlay[{plotT2, hLegend}, Alignment → Right],
  ToExpression[StringJoin["label", ToString[statTestT2]]], Top,
  LabelStyle → Directive[FontSize → 18, Bold, FontFamily → "Arial"]]
```

Out[•]= **Calculation Progress**

Export mean vector test results

```
In[•]:= filenameout = SystemDialogInput["FileSave"];
Export[filenameout, probPlot1, "TIFF", ImageResolution → 150]
```

```
Out[•]= /Users/n.macleod/Desktop/EIO-IMed/CVA Results/Hotellings T2 Test (BS).tif
```

Export mean vector simulation values (Monte Carlo & bootstrap procedures only)

```
In[•]:= filenameout = SystemDialogInput["FileSave"];
Export[filenameout, simFRatioT2, "CSV", "TextDelimiters" → ""]
```

```
Out[•]= /Users/n.macleod/Desktop/PalAss 2018/Dried Leaf Results/Single Species
  Results I/Aesculus chinensis/CVA Results/Hotelling's T2 Test (BS).tif
```

Test Two – Group Separation w/ the Log – Likelihood statistic.

Specify testing options

Standard (χ^2 – ratio) Distribution =

use this test if your data are normally distributed and the group covariance matrices are equal

Monte Carlo Simulated Distribution =

use this test if your data are not normally distributed and/or the group covariance matrices are not equal

Bootstrap Simulated Distribution =

use this test if your data are not normally distributed and/or the group covariance matrices are not equal

In[•]:=
```
Panel[
 Labeled[Column[{Row[{Panel[Labeled[InputField[Dynamic[hSize2], FieldSize → 10],
       "Enter histogram size (in pixels).", Top,
       LabelStyle → Directive[FontSize → 12, Bold, FontFamily → "Arial"]]],
     " ", Panel[Labeled[InputField[Dynamic[m2], FieldSize → 10],
       "Enter number of variables.", Top, LabelStyle →
        Directive[FontSize → 12, Bold, FontFamily → "Arial"]]], " ",
     Panel[Labeled[InputField[Dynamic[n2], FieldSize → 10],
       "Enter number of objects.", Top,
       LabelStyle → Directive[FontSize → 12, Bold, FontFamily → "Arial"]]]}],
    Row[{Panel[Labeled[InputField[Dynamic[iterLLR], FieldSize → 10],
       "No. of replicate iterations (Monte Carlo or bootstrapped only).",
       Top, LabelStyle → Directive[FontSize → 12,
         Bold, FontFamily → "Arial"]]], " ",
     Panel[Labeled[PopupMenu[Dynamic[statTestLLR],
        {1 → "Standard Parametric (χ^2-ratio) Distributions",
         2 → "Monte Carlo Simulated (χ^2-ratio) Distributions ",
         3 → "Bootstrapped Modelled (χ^2-ratio) Distributions"}],
       "Choose estimation procedure.", Top, LabelStyle →
        Directive[FontSize → 12, Bold, FontFamily → "Arial"]]]}]}, Center],
  "Log-Likelihood Mean Vector Test Control Parameters",
  Top,
  LabelStyle →
   Directive[FontSize → 14,
    Bold, FontFamily → "Arial"]]]
hSize2 = 500; nobins = 25; iterLLR = 1000; statTestLLR = 1;
```

Out[•]=

Log–Likelihood Mean Vector Test Control Parameters

Enter histogram size (in pixels).	Enter number of variables.	Enter number of objects.
hSize2	5400	25

No. of replicate iterations (Monte Carlo or bootstrapped only).

iterLLR

Choose estimation procedure.

Standard Parametric (χ^2–ratio) Distributions

Calculate Log – Likelihood Ratio

In[•]:=
```
If[statTestLLR == 1,
 phi = (n2 - 1 - (0.5 * (m2 + nGps))) * (Log[(Det[T] / Det[W])]);
```

```
    dof = m2 * (nGps - 1);
    prob = N[(1 - CDF[ChiSquareDistribution[m2 * (nGps - 1)], phi]) * 100];
    FTable = Table[" ", {3}, {2}];
    FTable[[1, 1]] = "Log Likelihood Ratio";
    FTable[[2, 1]] = "Degrees of Freedom";
    FTable[[3, 1]] = "Probability (%)";
    FTable[[1, 2]] = PaddedForm[phi, {4, 3}];
    FTable[[2, 2]] = dof;
    FTable[[3, 2]] = PaddedForm[prob, {5, 3}];
    rng = phi * 2;
    phiLegend = Labeled[
      Grid[FTable, BaseStyle → Directive[FontSize → 10, FontFamily → "Arial"],
       Alignment → {{Left, Right}}, Frame → True,
       Dividers → {{True, True}, {True}}], "Likelihood Ratio Table", Top,
      LabelStyle → Directive[FontSize → 12, Bold, FontFamily → "Arial"]];
    plot5 = Plot[PDF[ChiSquareDistribution[dof], x], {x, 0, rng},
      ImageSize → hSize2, Filling → Bottom, FillingStyle → Red,
      PlotRange → Full, AxesLabel → {"χ² Ratio", "Prob. Density"},
      LabelStyle → Directive[Black, FontSize → 12, FontFamily → "Arial"]];
    probPlot5 = Overlay[{plot5, phiLegend}, Alignment → Right];
    label = "Parametric Probability Results"];

  If[statTestLLR > 1, Panel[
    Labeled[ProgressIndicator[Dynamic[it], {1, iterLLR}], "Calculation Progress",
     Top, LabelStyle → Directive[FontSize → 12, Bold, FontFamily → "Arial"]]]]

  If[statTestLLR > 1,
    avg = Mean[x2];
    stDev = N[StandardDeviation[x2]];
    groupNames = Union[Group];
    numGroups = Length[groupNames];
    groupPosns = Table[Flatten[Position[Group, groupNames[[i]], 1]], {i, kg}];
    x3 = Table[0.0, {n2}, {m2}];
    simRatios = Table[0.0, {iterLLR}];

    phiTest = (n2 - 1 - (0.5 * (m2 + kg))) * (Log[(Det[T] / Det[W])]);
    chi2Phi = phiTest;

    Do[
     Label[123];
     If[statTestLLR == 2,
      Do[
       Do[
        Do[
         inum = groupPosns[[k, i]];
         x3[[inum, j]] = RandomReal[NormalDistribution[avg[[j]], stDev[[j]]]],
```

```
        {i, smpSize[[k]]}], {j, m2}], {k, kg}]];
    If[statTestLLR == 3,
     x3 = x2;
     Do[x3[[i]] = N[x2[[RandomInteger[{1, n2}]]]], {i, n2}]];
    {gndMean, simT, gNames, nGps, gpMeans, smpSize, simW, simB} =
     TWB[x3, n2, m2, Group];
    simPhi = (n2 - 1 - (0.5 * (m2 + kg))) * (Log[(Det[simT] / Det[simW])]);
    simRatios[[it]] = simPhi;
    If[Head[simRatios[[it]]] == Complex || simRatios[[it]] < 0.0, Goto[123]],
    {it, iterLLR}];

   simRatios = Sort[simRatios];
   simProb = Table[0.0, {3}];
   simProb[[1]] = phiTest;
   simProb[[2]] = chi2Phi;
   knt = 0;
   Do[If[simRatios[[i]] > phiTest, knt = knt + 1], {i, iterLLR}];
   simProb[[3]] = N[knt / iterLLR] * 100;

   FTable = Table[" ", {4}, {2}];
   FTable[[1, 1]] = "Log Likelihood Ratio";
   FTable[[2, 1]] = "Degrees of Freedom";
   FTable[[3, 1]] = "No. of Iterations";
   FTable[[4, 1]] = "Probability (%)";
   FTable[[1, 2]] = PaddedForm[phiTest, {4, 3}];
   FTable[[2, 2]] = m2 * (kg - 1);
   FTable[[3, 2]] = iterLLR;
   FTable[[4, 2]] = PaddedForm[simProb[[3]], {5, 3}];

   If[simProb[[3]] ≥ 5.0, rng = phiTest * 2, rng = phiTest];
   phiLegend = Labeled[
     Grid[FTable, BaseStyle → Directive[FontSize → 10, FontFamily → "Arial"],
      Alignment → {{Left, Right}}, Frame → True,
      Dividers → {{True, True}, {True}}], "Likelihood Ratio Table", Top,
     LabelStyle → Directive[FontSize → 12, Bold, FontFamily → "Arial"]];
   plot5 = Histogram[simRatios, "FreedmanDiaconis",
     ChartStyle → {Red}, ImageSize → hSize2, PlotRange → Full,
     LabelStyle → Directive[FontSize → 12, Black, FontFamily → "Arial"],
     AxesLabel → {"χ² Ratio", "Frequency"}, ImageSize → hSizeMVT];
   probPlot5 = Overlay[{plot5, phiLegend}, Alignment → Right];
   If[statTestLLR == 2, label = "Monte Carlo Simulation Results"];
   If[statTestLLR == 3, label = "Bootstrap Modelling Results"]];

 probPlot = Labeled[probPlot5, label, Top,
   LabelStyle → Directive[FontSize → 18, Bold, FontFamily → "Arial"]]
```

Export mean vector test results

In[•]:=
```
filenameout = SystemDialogInput["FileSave"];
Export[filenameout, probPlot, "TIFF", ImageResolution → 150]
```

Out[•]=
```
/Users/n.macleod/Desktop/Israel_Med_Q2/CVA Results/Log Likelihood Test (PM).tif
```

Export mean vector simulation values (Monte Carlo & bootstrap procedures only)

In[•]:=
```
filenameout = SystemDialogInput["FileSave"];
Export[filenameout, simRatios, "CSV", "TextDelimiters" → ""]
```

Out[•]=
```
/Users/nm/Desktop/Lupus II/Dorsal/Eigenimage
   (Dorsal)/CVA Results/Log Likelihood phi (BS) values.csv
```

Create 2 D scatterplot (use only for datasets containing three or more groups).

Specify 2 D plot options.
You must run this code after you read in the data so it can pick up the proper variable names.

```
In[•]:= cvNames = Table[StringJoin["CV-", ToString[i]], {i, noAxes}];
Panel[
 Labeled[Column[{Row[{Panel[Labeled[PopupMenu[Dynamic[xAxisName], cvNames],
       "Select variable to be plotted on x-Axis.", Top, LabelStyle →
        Directive[FontSize → 12, Bold, FontFamily → "Arial"]]], "  ",
     Panel[Labeled[PopupMenu[Dynamic[yAxisName], cvNames],
       "Select variable to be plotted on y-Axis.", Top,
       LabelStyle → Directive[FontSize → 12, Bold, FontFamily → "Arial"]]]}],
   Row[{
     Panel[Labeled[PopupMenu[Dynamic[pltAspect],
        {1 → "Golden Ratio Plot", 2 → "Square Plot (equi-length axes)",
         3 → "True-Scale Plot (actual axis scales)"}],
       "Enter plot aspect ratio type.", Top, LabelStyle →
        Directive[FontSize → 12, Bold, FontFamily → "Arial"]]], "  ",
     Panel[Labeled[PopupMenu[Dynamic[lch], {1 → "Simple scatterplot",
         2 → "Scatterplot w/ convex hulls"}],
       "Show group domians?", Top, LabelStyle →
        Directive[FontSize → 12, Bold, FontFamily → "Arial"]]], "  ",
     Panel[Labeled[PopupMenu[Dynamic[ptsJoin], {1 → "No", 2 → "Yes"}],
       "Join datapoints?", Top,
       LabelStyle → Directive[FontSize → 12, Bold, FontFamily → "Arial"]]]}],
   Row[{Panel[Labeled[InputField[Dynamic[pltSize], FieldSize → 10],
       "Enter plot size value.", Top,
       LabelStyle → Directive[FontSize → 12, Bold, FontFamily → "Arial"]]],
     "  ", Panel[Labeled[InputField[Dynamic[pltPad], FieldSize → 10],
       "Enter plot margin padding value.", Top, LabelStyle →
        Directive[FontSize → 12, Bold, FontFamily → "Arial"]]], "  ",
     Panel[Labeled[InputField[Dynamic[iconSize], FieldSize → 10],
       "Enter plot icon size value.", Top, LabelStyle →
        Directive[FontSize → 12, Bold, FontFamily → "Arial"]]]}]}, Center],
  "2D Plot Options", Top, LabelStyle → Directive[FontSize → 18,
    Bold, FontFamily → "Arial"]]]
pltSize = 500; iconSize = 0.03; pltPad = 0.1; xAxisName = cvNames[[1]];
yAxisName = cvNames[[2]];
 ptsJoin = 1; dataTrans = 1;  pltAspect = 1 ; lch = 2;
```

Out[∘]=

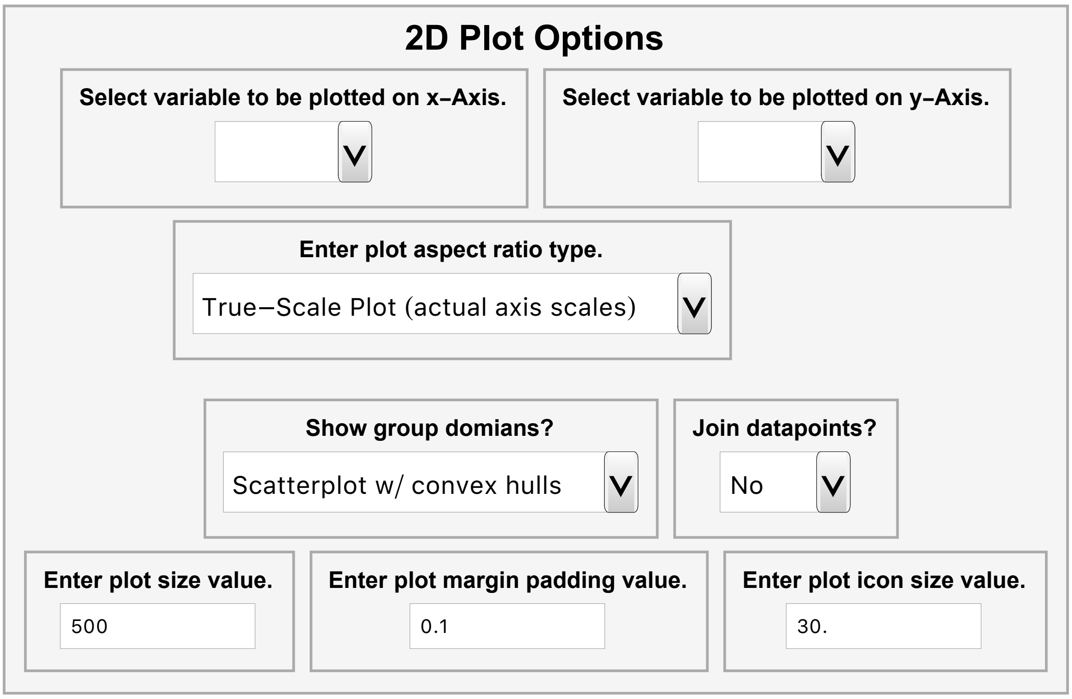

Plot script

In[∘]:=

```
Do[If[xAxisName == cvNames[[j]], axis1 = j], {j, noAxes}];
Do[If[yAxisName == cvNames[[j]], axis2 = j], {j, noAxes}];

groupNames = Union[Group];
numGroups = Length[groupNames];
groupPosns =
  Table[Flatten[Position[Group, groupNames[[i]], 1]], {i, numGroups}];
eScoresT = Transpose[eScores];

xAxis = eScoresT[[axis1]]; yAxis = eScoresT[[axis2]];
lab1 = StringJoin[{"Canonical Variate ", ToString[axis1]},
   {" (Var. =", ToString[t3[[axis1 + 1, 3]]]}, {"%)"}];
lab2 = StringJoin[{"Canonical Variate ", ToString[axis2]},
   {" (Var. =", ToString[t3[[axis2 + 1, 3]]]}, {"%)"}];
maxx =
 Max[
  xAxis];
minx = Min[xAxis];
maxy = Max[yAxis];
miny = Min[yAxis];

If[pltAspect == 1 || pltAspect == 3,
  xPlotLow = minx; xPlotHi = maxx; yPlotLow = miny; yPlotHi = maxy];
If[pltAspect == 2,
  If[minx > miny,
   xPlotLow = miny; yPlotLow = miny,
```

```
    xPlotLow = minx; yPlotLow = minx ]];
If[pltAspect == 2,
   If[maxx < maxy,
    xPlotHi = maxy; yPlotHi = maxy,
    xPlotHi = maxx; yPlotHi = maxx]];
If[pltAspect == 1, aRatio = 1 / N[GoldenRatio]];
If[pltAspect == 2, aRatio = 1];
If[pltAspect == 3, aRatio = Automatic];

tmpPoints = Transpose[List[xAxis, yAxis]];
pltPoints = Table[tmpPoints[[groupPosns[[j]]]], {j, numGroups}];
iconList = Flatten[Table[
     {Graphics[{EdgeForm[{Thickness[0.005], Black}],
         Hue[N[((numGroups + 1) - j) / numGroups]],
         Disk[{0, 0}, Scaled[iconSize]]}]}, {j, numGroups}]];

If[lch == 1 || ptsJoin == 1,
  Do[
   pltTable[[k, 1]] = pTmp =
     ListPlot[pltPoints[[k]], AspectRatio → aRatio, Frame → True, Joined → False,
      Axes → False, PlotRange → {{xPlotLow, xPlotHi}, {yPlotLow, yPlotHi}},
      PlotRangePadding → Scaled[pltPad], Ticks → Automatic, FrameLabel →
       {lab1, lab2}, PlotMarkers → iconList[[k]], ImageSize → pltSize, LabelStyle →
       Directive[FontSize → 14, Black, FontFamily → "Arial"]], {k, numGroups}],
  Do[
   pltTable[[k, 1]] =
    ListPlot[pltPoints[[k]], Frame → True, Axes → False, AspectRatio → aRatio,
     PlotRange → {{xPlotLow, xPlotHi}, {yPlotLow, yPlotHi}}, PlotRangePadding →
      Scaled[pltPad], Ticks → Automatic, FrameLabel → {lab1, lab2},
     LabelStyle → Directive[Black, FontSize → 14, FontFamily → "Arial"],
     ImageSize → pltSize, PlotStyle → Directive[Disk[],
       Hue[N[((numGroups + 1) - k) / numGroups]], EdgeForm[{Thickness[1.0], Black}],
       PointSize[Scaled[iconSize - 0.009]]]], {k, numGroups}]]

If[ptsJoin == 2,
   Do[
    pltTable[[k, 2]] = ListLinePlot[pltPoints[[k]],
      AspectRatio → aRatio, Frame → True, Joined → True, Axes → False,
      PlotStyle → Directive[Hue[N[((numGroups + 1) - k) / numGroups]], Thin],
      PlotRange → {{xPlotLow, xPlotHi}, {yPlotLow, yPlotHi}},
      PlotRangePadding → Scaled[pltPad], Ticks → Automatic,
      FrameLabel → {lab1, lab2}, ImageSize → pltSize, LabelStyle →
       Directive[FontSize → 14, Black, FontFamily → "Arial"]], {k, numGroups}]];

If[lch == 2,
   Do[
    hull = ConvexHullMesh[pltPoints[[k]]];
```

```
    pltTable[[k, 3]] = HighlightMesh[hull,
      Style[2, Opacity[0.2], Hue[N[((numGroups + 1) - k) / numGroups]]],
      Frame → True, Axes → False, AspectRatio → aRatio,
      PlotRange → {{xPlotLow, xPlotHi}, {yPlotLow, yPlotHi}}, PlotRangePadding →
       Scaled[pltPad], Ticks → Automatic, FrameLabel → {lab1, lab2},
      LabelStyle → Directive[Black, FontSize → 14, FontFamily → "Arial"],
      ImageSize → pltSize], {k, numGroups}]];

If[ptsJoin == 1 && lch == 1, p0 = Show[pltTable[[All, 1]]]];
If[ptsJoin == 2 && lch == 1, p0 = Show[pltTable[[All, 2]], pltTable[[All, 1]]]];
If[ptsJoin == 1 && lch == 2, p0 = Show[pltTable[[All, 3]], pltTable[[All, 1]]]];
If[ptsJoin == 2 && lch == 2,
  p0 = Show[pltTable[[All, 3]], pltTable[[All, 2]], pltTable[[All, 1]]]];

p1 = Labeled[p0, "          CV Score Plot", Top,
    LabelStyle → Directive[FontSize → 18, Bold, FontFamily → "Arial"]];
g1 = Grid[Table[
     {Graphics[{EdgeForm[{Thin, Black}], Hue[N[((numGroups + 1) - j) / numGroups]],
        Disk[]}]}, {j, numGroups}], Frame → False, ItemSize → 0.9];
g2 = Grid[Partition[groupNames, 1], Alignment → Left,
    BaseStyle → {FontFamily → "Arial", FontSize → 13, Italic}];
p2 = Labeled[Text[Grid[{{g1, g2}}, Alignment → Bottom, Frame → True]], "Legend",
    Top, LabelStyle → Directive[Black, FontSize → 18, Bold, FontFamily → "Arial"]];

plt2D = Grid[{{p1, p2}}, BaselinePosition → Top, Alignment → Top]
```

Export current 2 D plot.

```
In[∘]:= filenameout = SystemDialogInput["FileSave"];
        Export[filenameout, plt2D, "TIFF", ImageResolution → 150]

Out[∘]= /Users/n.macleod/Desktop/Africa-Spain/CVA Results/CV-3 vs CV-2.tif
```

Reverse eigenvector axes & recalculate scores.

```
If[eMethod == 1, eVecs = eVecs * -1, eVecs = w * -1];
eScores = x2.eVecs;
```

Label plotted points.

In[•]:=
```
pn1 = p0;
namePoints = tmpPoints;
tempPointsT = Transpose[tmpPoints];
mxY = Max[tempPointsT[[2]]];
mnY = Min[tempPointsT[[2]]];
incY = N[(mxY - mnY) / 15];
Do[namePoints[[i, 2]] = tmpPoints[[i, 2]] - incY, {i, n2}]
nPointsTable = Table[{Text[objNames[[i]], namePoints[[i]], {-1, 0}]}, {i, n2}];
pn2 = Graphics[nPointsTable, Frame → True, AspectRatio → aRatio , Axes → False,
   FrameLabel  → {lab1, lab2}, PlotRangePadding → Scaled[pltPad], BaseStyle →
    Directive[FontSize → 12, FontFamily → "Arial"], ImageSize → pltSize];

p1 = Labeled[Show[pn1, pn2, BaseStyle → {FontFamily → "Arial"}],
   "           CV Score Plot", Top,
   LabelStyle → Directive[FontSize → 18, Bold, FontFamily → "Arial"]];

Plt2D = Grid[{{p1, p2}}, BaselinePosition → Top, Alignment → Top]
```

Export current 2 D plot.

In[•]:=
```
filenameout = SystemDialogInput["FileSave"];
Export[filenameout, Plt2D, "TIFF", ImageResolution → 150]
```

Create 3 D scatterplot (use only for datasets containing four or more groups).

Specify 3 D plot options.
You must run this code after you read in the data so it can pick up the proper variable names.

```
In[•]:= cvNames = Table[StringJoin["CV-", ToString[i]], {i, noAxes}];
Panel[
 Labeled[Column[{Row[{Panel[Labeled[PopupMenu[Dynamic[xAxisName], cvNames],
        "Select variable to be plotted on x-Axis.", Top, LabelStyle →
         Directive[FontSize → 12, Bold, FontFamily → "Arial"]]], "  ",
      Panel[Labeled[PopupMenu[Dynamic[yAxisName], cvNames],
        "Select variable to be plotted on y-Axis.", Top, LabelStyle →
         Directive[FontSize → 12, Bold, FontFamily → "Arial"]]], "  ",
      Panel[Labeled[PopupMenu[Dynamic[zAxisName], cvNames],
        "Select variable to be plotted on z-Axis.", Top,
        LabelStyle → Directive[FontSize → 12, Bold, FontFamily → "Arial"]]]}],
    Row[{
      Panel[Labeled[PopupMenu[Dynamic[pltAspect],
          {1 → "Golden Ratio Plot", 2 → "Square Plot (equi-length axes)",
           3 → "True-Scale Plot (actual axis scales)"}],
        "Enter plot aspect ratio type.", Top, LabelStyle →
         Directive[FontSize → 12, Bold, FontFamily → "Arial"]]], "  ",
      Panel[Labeled[PopupMenu[Dynamic[lch], {1 → "Simple scatterplot",
           2 → "Scatterplot w/ convex hulls"}],
        "Show group domians?", Top, LabelStyle →
         Directive[FontSize → 12, Bold, FontFamily → "Arial"]]], " ",
      Panel[Labeled[PopupMenu[Dynamic[ptsJoin], {1 → "No", 2 → "Yes"}],
        "Join datapoints?", Top,
        LabelStyle → Directive[FontSize → 12, Bold, FontFamily → "Arial"]]]}],
    Row[{Panel[Labeled[InputField[Dynamic[pltSize], FieldSize → 5],
        "Enter plot size value.", Top,
        LabelStyle → Directive[FontSize → 12, Bold, FontFamily → "Arial"]]],
      " ", Panel[Labeled[InputField[Dynamic[pltPad], FieldSize → 5],
        "Enter plot margin padding value.", Top, LabelStyle →
         Directive[FontSize → 12, Bold, FontFamily → "Arial"]]], " ",
      Panel[Labeled[InputField[Dynamic[iconSize3D], FieldSize → 5],
        "Enter plot icon size value.", Top, LabelStyle →
         Directive[FontSize → 12, Bold, FontFamily → "Arial"]]]}]}, Center],
  "3D Plot Options", Top, LabelStyle → Directive[FontSize → 18,
    Bold, FontFamily → "Arial"]]]
pltSize = 500; iconSize3D = 60; pltPad = 0.1; xAxisName = cvNames[[1]];
 yAxisName = cvNames[[2]];
zAxisName = cvNames[[3]]; ptsJoin = 1;
dataTrans = 1;
pltAspect = 3 ; lch = 2;
```

Out[•]=

3D Plot Options

Select variable to be plotted on x-Axis.

Select variable to be plotted on y-Axis.

Select variable to be plotted on z-Axis.

Enter plot aspect ratio type.

True–Scale Plot (actual axis scales)

Show group domians?

Scatterplot w/ convex hulls

Join datapoints?

No

Enter plot size value.

500

Enter plot margin padding value.

0.1

Enter plot icon size value.

10

Plot script

In[•]:=

```
Do[If[xAxisName == cvNames[[j]], axis1 = j], {j, noAxes}]
Do[If[yAxisName == cvNames[[j]], axis2 = j], {j, noAxes}]
Do[If[zAxisName == cvNames[[j]], axis3 = j], {j, noAxes}]
iconSize = iconSize3D - 50.0;

groupNames = Union[Group];
numGroups = Length[groupNames];
groupPosns =
  Table[Flatten[Position[Group, groupNames[[i]], 1]], {i, numGroups}];
eScoresT = Transpose[eScores];

xAxis = eScoresT[[axis1]];
yAxis = eScoresT[[axis2]];
zAxis = eScoresT[[axis3]];
lab1 = StringJoin["CV-", ToString[axis1]];
lab2 = StringJoin["CV-", ToString[axis2]];
lab3 = StringJoin["CV-", ToString[axis3]];
maxx = Max[xAxis];
minx = Min[xAxis];
maxy = Max[yAxis];
miny = Min[yAxis];
maxz = Max[zAxis]; minz = Min[zAxis];
```

```
If[pltAspect == 1 || pltAspect == 3,
  xPlotLow = minx; xPlotHi = maxx; yPlotLow = miny; yPlotHi = maxy];
If[pltAspect == 2,
  If[minx > miny,
   xPlotLow = miny; yPlotLow = miny,
   xPlotLow = minx; yPlotLow = minx ]];
If[pltAspect == 2,
  If[maxx < maxy,
   xPlotHi = maxy; yPlotHi = maxy,
   xPlotHi = maxx; yPlotHi = maxx]];

If[pltAspect == 1, bRatio = {1.61803, 1, 1}];
If[pltAspect == 2, bRatio = {1, 1, 1}];
If[pltAspect == 3, bRatio = Automatic];

points3 = Transpose[List[xAxis, yAxis, zAxis]];
gp1 = groupPosns;
h = Table[0, {n2}];
Do[
 tmp = gp1[[i]];
 itr = Length[tmp];
 Do[h[[tmp[[j]]]] = Hue[N[((numGroups + 1) - i) / numGroups]], {j, itr}],
 {i, numGroups}]

If[lch == 1 && pltAspect == 3,
  pltPoints = Table[{h[[i]], Sphere[points3[[i]], iconSize3D / 55]}, {i, n2}],
  pltPoints =
   Table[{h[[i]], AbsolutePointSize[iconSize], Point[points3[[i]]]}, {i, n2}]];
p0 = Graphics3D[pltPoints, Axes → True, Boxed → True,
   PlotRangePadding → Scaled[pltPad],
   LabelStyle → Directive[FontSize → 12, Black, FontFamily → "Arial"],
   AxesLabel → {lab1, lab2, lab3}, ImageSize → pltSize, BoxRatios → bRatio];

If[ptsJoin == 2,
  pltLineTable = Table[" ", {numGroups}];
  gpPoints = Table[points3[[groupPosns[[j]]]], {j, numGroups}];
  Do[
   pltLineTable[[k]] =
    Graphics3D[{Hue[N[((numGroups + 1) - k) / numGroups]], Line[gpPoints[[k]]]},
     Axes → True, Boxed → True, PlotRangePadding → Scaled[pltPad],
     LabelStyle → Directive[FontSize → 12, Black, FontFamily → "Arial"],
     AxesLabel → {lab1, lab2, lab3}, ImageSize → pltSize,
     BoxRatios → bRatio, ViewPoint -> {xax, yax, zax}], {k, numGroups}];
  pTmp = Graphics3D[pltPoints, Axes → True, Boxed → True,
    PlotRangePadding → Scaled[pltPad],
    LabelStyle → Directive[FontSize → 12, Black, FontFamily → "Arial"],
```

```
    AxesLabel → {lab1, lab2, lab3}, ImageSize → pltSize,
    BoxRatios → bRatio, ViewPoint -> {xax, yax, zax}];
  p0 = Show[{pltLineTable, pTmp}]];

If[lch == 2,
  pltMeshTable = Table[" ", {numGroups}];
  gpPoints = Table[points3[[groupPosns[[j]]]], {j, numGroups}];
  Do[
   {n3, m3} = Dimensions[gpPoints[[k]]];
   pltPoints = Table[{Hue[N[((numGroups + 1) - k) / numGroups]],
       AbsolutePointSize[iconSize3D], Point[gpPoints[[k, i]]]}, {i, n3}];
   pTmp = Graphics3D[pltPoints, Axes → True, Boxed → True,
     PlotRangePadding → Scaled[pltPad],
     LabelStyle → Directive[FontSize → 12, Black, FontFamily → "Arial"],
     AxesLabel → {lab1, lab2, lab3}, ImageSize → pltSize,
     BoxRatios → bRatio, ViewPoint -> {xax, yax, zax}];
   cHull3D = ConvexHullMesh[gpPoints[[k]], BaseStyle → {EdgeForm[]},
     Boxed → True, Axes → True, PlotRangePadding → Scaled[pltPad],
     LabelStyle → Directive[FontSize → 12, Black, FontFamily → "Arial"],
     AxesLabel → {lab1, lab2, lab3}, ImageSize → pltSize,
     BoxRatios → bRatio, ViewPoint -> {xax, yax, zax}];
   pltMeshTable[[k]] = Show[{HighlightMesh[cHull3D, Style[2, Opacity[0.2],
        Hue[N[((numGroups + 1) - k) / numGroups]]]], p0}], {k, kg}];
  p0 = Show[pltMeshTable]];

p1 = Labeled[p0, "           CV Score Plot", Top,
   LabelStyle → Directive[FontSize → 18, Bold, FontFamily → "Arial"]];
If[lch == 1 && pltAspect == 3, g1 = Table[Graphics[
     {Inset[Graphics3D[{Hue[N[((numGroups + 1) - i) / numGroups]], Sphere[]},
        Boxed → False]]}], {i, numGroups}],
  g1 = Table[Graphics[{Inset[Graphics[{Hue[N[((numGroups + 1) - i) / numGroups]],
         Disk[{0, 0}, Scaled[0.3]]}]]}], {i, numGroups}]];
g2 = Labeled[Text[Grid[Transpose[Partition[Join[g1, groupNames], numGroups]],
     ItemSize → {{Scaled[0.03], Automatic}}, Alignment → Left, BaseStyle →
      {FontSize → 12, FontFamily → "Arial", Italic}, Frame → True]], "Legend",
   Top, LabelStyle → Directive[FontSize → 14, Bold, FontFamily → "Arial"]];

plt3D = Grid[{{p1, g2}}, BaselinePosition → Top, Alignment → Top]
```

Adjust orientation of 3 D plot (if necessary).
You must replot the data to activate the changes. These changes will be able to be exported using the script below.

In[∘]:=
```
Panel[
 Labeled[Row[{Labeled[Slider[Dynamic[xax], {-10, 10}, Appearance → "Labeled"],
      "x-Axis Viewpoint", Top,
      LabelStyle → Directive[FontSize → 10, Bold, FontFamily → "Arial"]] ×
     Labeled[Slider[Dynamic[yax], {-10, 10}, Appearance → "Labeled"],
      "y-Axis Viewpoint", Top,
      LabelStyle → Directive[FontSize → 10, Bold, FontFamily → "Arial"]] ×
     Labeled[Slider[Dynamic[zax], {-10, 10}, Appearance → "Labeled"],
      "z-Axis Viewpoint", Top,
      LabelStyle → Directive[FontSize → 10, Bold, FontFamily → "Arial"]]}],
  "3D Plot Orientation Controls", Top, LabelStyle →
   Directive[FontSize → 14, Bold, FontFamily → "Ariel"]]]
xax = 2.5; yax = -2.5; zax = 2.5;
```

Out[∘]=

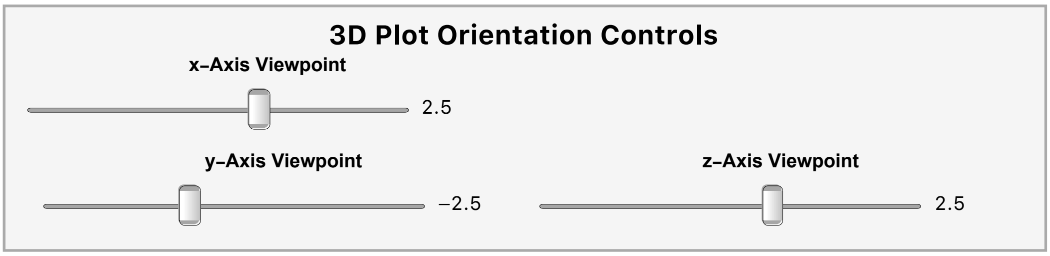

Export current 3 D plot.

In[∘]:=
```
filenameout = SystemDialogInput["FileSave"];
Export[filenameout, plt3D, "TIFF", ImageResolution → 150]
```

Estimate statistical significance of group mean –vector differences using multi –group Wilks λ,
Roy' s Maximum Root,
Pillai' s Trace, Hotelling – Lawes Trace and Likelihood Ratio tests

Specify testing options

Standard (F – ratio) Distribution =
use this test if your data are normally distributed and the group covariance matrices are equal

Monte Carlo Simulated Distribution =
use this test if your data are not normally distributed and/or the group covariance matrices are not equal

Bootstrap Simulated Distribution =
use this test if your data are not normally distributed and/or the group covariance matrices are not equal

In[∘]:=
```
Panel[
 Labeled[
  Column[{Row[{Panel[Labeled[InputField[Dynamic[hSizeMVT], FieldSize → 10],
        "Enter histogram size (in pixels).", Top,
        LabelStyle → Directive[FontSize → 12, Bold, FontFamily → "Arial"]]],
      " ", Panel[Labeled[InputField[Dynamic[m2], FieldSize → 10],
        "Enter number of variables.", Top, LabelStyle →
         Directive[FontSize → 12, Bold, FontFamily → "Arial"]]], " ",
      Panel[Labeled[InputField[Dynamic[n2], FieldSize → 10],
        "Enter number of objects.", Top,
        LabelStyle → Directive[FontSize → 12, Bold, FontFamily → "Arial"]]]}],
    Row[{Panel[Labeled[InputField[Dynamic[iter], FieldSize → 10],
        "No. of replicate iterations (Monte Carlo or bootstrapped only).",
        Top, LabelStyle → Directive[FontSize → 12,
          Bold, FontFamily → "Arial"]]], " ",
      Panel[Labeled[PopupMenu[Dynamic[statTest],
         {1 → "Standard Parametric (F-ratio) Distributions",
          2 → "Monte Carlo Simulated (F-ratio) Distributions ",
          3 → "Bootstrapped Modelled (F-ratio) Distributions"}],
        "Choose estimation procedure.", Top, LabelStyle →
         Directive[FontSize → 12, Bold, FontFamily → "Arial"]]]}]}, Center],
  "Multi-Group Mean Vector Test Control Parameters", Top,
  LabelStyle →
   Directive[FontSize → 14,
    Bold, FontFamily → "Arial"]]]
hSizeMVT = 300; nobins = 25; iter = 1000; statTest = 1;
```

Out[∘]=

Multi–Group Mean Vector Test Control Parameters

Enter histogram size (in pixels).	Enter number of variables.	Enter number of objects.
hSizeMVT	5400	25

No. of replicate iterations (Monte Carlo or bootstrapped only).

iter

Choose estimation procedure.

Standard Parametric (F–ratio) Distributions

Calculate and display probability statistics

In[∘]:=
```
If[statTest == 1,
  lambda = Det[W] / Det[T];
  df1 = m2 * (kg - 1);
```

```
wCoef = N[n2 - 1 - (m2 + kg) / 2];
tCoef = N[Sqrt[(df1^2 - 4) / (m2^2 + (kg - 1)^2 - 5)]];
df2 = (wCoef * tCoef) - (df1 / 2) + 1;
Flambda = N[((1 - lambda^(1/tCoef)) / lambda^(1/tCoef)) * (df2 / df1)];
prob = N[(1 - CDF[FRatioDistribution[df1, df2], Flambda]) * 100];
FTable = Table[" ", {4}, {2}];
FTable[[1, 1]] = "Wilk's Lambda";
FTable[[2, 1]] = "Observed F-Ratio";
FTable[[3, 1]] = "Degrees of Freedom";
FTable[[4, 1]] = "Probability (%)";
FTable[[1, 2]] = PaddedForm[lambda, {4, 3}];
FTable[[2, 2]] = PaddedForm[Flambda, {5, 3}];
FTable[[3, 2]] =
 StringJoin[ToString[Round[df1]], ",", ToString[Round[df2]]];
FTable[[4, 2]] = PaddedForm[prob, {5, 3}];
hLegend = Labeled[
   Grid[FTable, BaseStyle → Directive[FontSize → 10, FontFamily → "Arial"],
    Alignment → {{Left, Right}}, Frame → True,
    Dividers → {{True, True}, {True}}], "Wilk's λ Table", Top,
   LabelStyle → Directive[FontSize → 12, Bold, FontFamily → "Arial"]];
plot1 = Plot[Table[PDF[FRatioDistribution[n, df2], x], {n, {df1}}] // Evaluate,
   {x, 0, Flambda}, PlotRange → {{0.0, 50.0}, {0.0, 1.0}},
   Filling → Axis, Exclusions → None, ImageSize → hSizeMVT,
   Filling → Bottom, FillingStyle → Directive[Opacity[1.0], Red],
   AxesLabel → {"F-Ratio", "Prob. Density"},
   LabelStyle → Directive[Black, FontSize → 12, FontFamily → "Arial"]];
probPlot1 = Overlay[{plot1, hLegend}, Alignment → Right];

rmr = eVals[[1]];
d = Max[m2, kg - 1];
df1 = m2;
df2 = n2 - kg - d - 1;
Frmr = (df2 / df1) * rmr;
prob = N[(1 - CDF[FRatioDistribution[df1, df2], Frmr]) * 100];
FTable = Table[" ", {4}, {2}];
FTable[[1, 1]] = "Roy's Maximum Root";
FTable[[2, 1]] = "Observed F-Ratio";
FTable[[3, 1]] = "Degrees of Freedom";
FTable[[4, 1]] = "Probability (%)";
FTable[[1, 2]] = PaddedForm[rmr, {4, 3}];
FTable[[2, 2]] = PaddedForm[Frmr, {5, 3}];
FTable[[3, 2]] =
 StringJoin[ToString[Round[df1]], ",", ToString[Round[df2]]];
FTable[[4, 2]] = PaddedForm[prob, {5, 3}];
hLegend = Labeled[
```

```
  Grid[FTable, BaseStyle → Directive[FontSize → 10, FontFamily → "Arial"],
   Alignment → {{Left, Right}}, Frame → True,
   Dividers → {{True, True}, {True}}], "Roy's Max. Root Table", Top,
  LabelStyle → Directive[FontSize → 12, Bold, FontFamily → "Arial"]];
plot2 = Plot[Table[PDF[FRatioDistribution[n, df2], x], {n, {df1}}] // Evaluate,
  {x, 0, Frmr}, PlotRange → {{0.0, 50.0}, {0.0, 1.0}},
  Filling → Axis, Exclusions → None, ImageSize → hSizeMVT,
  Filling → Bottom, FillingStyle → Directive[Opacity[1.0], Magenta],
  AxesLabel → {"F-Ratio", "Prob. Density"},
  LabelStyle → Directive[Black, FontSize → 12, FontFamily → "Arial"]];
probPlot2 = Overlay[{plot2, hLegend}, Alignment → Right];

V = 0.0;
Do[V = V + (eVals[[j]] / (1 + eVals[[j]])), {j, noVals}];
s = Min[m2, kg - 1];
d = Max[m2, kg - 1];
df1 = s * d;
df2 = s * (n2 - kg - m2 + s);
FV = ((n2 - kg - m2 + s) * V) / (d (s - V));
prob = N[(1 - CDF[FRatioDistribution[df1, df2], FV]) * 100];
FTable = Table[" ", {4}, {2}];
FTable[[1, 1]] = "Pillai's Trace";
FTable[[2, 1]] = "Observed F-Ratio";
FTable[[3, 1]] = "Degrees of Freedom";
FTable[[4, 1]] = "Probability (%)";
FTable[[1, 2]] = PaddedForm[V, {4, 3}];
FTable[[2, 2]] = PaddedForm[FV, {5, 3}];
FTable[[3, 2]] =
 StringJoin[ToString[Round[df1]], ",", ToString[Round[df2]]];
FTable[[4, 2]] = PaddedForm[prob, {5, 3}];
hLegend = Labeled[
  Grid[FTable, BaseStyle → Directive[FontSize → 10, FontFamily → "Arial"],
   Alignment → {{Left, Right}}, Frame → True,
   Dividers → {{True, True}, {True}}], "Pillai's Trace Table", Top,
  LabelStyle → Directive[FontSize → 12, Bold, FontFamily → "Arial"]];
plot3 = Plot[Table[PDF[FRatioDistribution[n, df2], x], {n, {df1}}] // Evaluate,
  {x, 0, FV}, PlotRange → {{0.0, 50.0}, {0.0, 1.0}}, Filling → Axis,
  Exclusions → None, ImageSize → hSizeMVT, Filling → Bottom, FillingStyle →
   Directive[Opacity[1.0], Blue], AxesLabel → {"F-Ratio", "Prob. Density"},
  LabelStyle → Directive[Black, FontSize → 12, FontFamily → "Arial"]];
probPlot3 = Overlay[{plot3, hLegend}, Alignment → Right];

U = 0.0;
Do[U = U + eVals[[j]], {j, noVals}];
s = Min[m2, kg - 1];
A = N[(Abs[kg - m2 - 1] - 1) / 2];
B = N[(n2 - kg - m2 - 1) / 2];
```

```
df1 = s * ((2 * A) + s + 1);
df2 = 2 * ((s * B) + 1);
FU = (df2 * U) / (s * df1);
prob = N[(1 - CDF[FRatioDistribution[df1, df2], FU]) * 100];
FTable = Table[" ", {4}, {2}];
FTable[[1, 1]] = "Lawes-Hotelling Trace";
FTable[[2, 1]] = "Observed F-Ratio";
FTable[[3, 1]] = "Degrees of Freedom";
FTable[[4, 1]] = "Probability (%)";
FTable[[1, 2]] = PaddedForm[U, {4, 3}];
FTable[[2, 2]] = PaddedForm[FU, {5, 3}];
FTable[[3, 2]] =
 StringJoin[ToString[Round[df1]], ",", ToString[Round[df2]]];
FTable[[4, 2]] = PaddedForm[prob, {5, 3}];
hLegend = Labeled[
  Grid[FTable, BaseStyle → Directive[FontSize → 10, FontFamily → "Arial"],
   Alignment → {{Left, Right}}, Frame → True, Dividers → {{True, True}, {True}}],
  "Lawes-Hotelling Trace Table", Top,
  LabelStyle → Directive[FontSize → 12, Bold, FontFamily → "Arial"]];
plot4 = Plot[Table[PDF[FRatioDistribution[n, df2], x], {n, {df1}}] // Evaluate,
  {x, 0, FU}, PlotRange → {{0.0, 50.0}, {0.0, 1.0}}, Filling → Axis,
  Exclusions → None, ImageSize → hSizeMVT, Filling → Bottom, FillingStyle →
   Directive[Opacity[1.0], Green], AxesLabel → {"F-Ratio", "Prob. Density"},
  LabelStyle → Directive[Black, FontSize → 12, FontFamily → "Arial"]];
probPlot4 = Overlay[{plot4, hLegend}, Alignment → Right];

phi = (n2 - 1 - (0.5 * (m2 + nGps))) * (Log[(Det[T] / Det[W])]);
dof = m2 * (nGps - 1);
prob = N[(1 - CDF[ChiSquareDistribution[m2 * (nGps - 1)], phi]) * 100];
FTable = Table[" ", {4}, {2}];
FTable[[1, 1]] = "Log Likelihood Ratio";
FTable[[2, 1]] = "Observed χ² Ratio";
FTable[[3, 1]] = "Degrees of Freedom";
FTable[[4, 1]] = "Probability (%)";
FTable[[1, 2]] = PaddedForm[phi, {4, 3}];
FTable[[2, 2]] = PaddedForm[phi, {4, 3}];
FTable[[3, 2]] = dof;
FTable[[4, 2]] = PaddedForm[prob, {5, 3}];
If[prob ≥ 5.0, rng = phi * 2, rng = phi];
phiLegend = Labeled[
  Grid[FTable, BaseStyle → Directive[FontSize → 10, FontFamily → "Arial"],
   Alignment → {{Left, Right}}, Frame → True,
   Dividers → {{True, True}, {True}}], "Likelihood Ratio Table", Top,
  LabelStyle → Directive[FontSize → 12, Bold, FontFamily → "Arial"]];
plot5 = Plot[PDF[ChiSquareDistribution[dof], x], {x, 0, rng},
  ImageSize → hSizeMVT, Filling → Bottom, FillingStyle → Yellow,
  PlotRange → Full, AxesLabel → {"χ² Ratio", "Prob. Density"},
```

```
    LabelStyle → Directive[Black, FontSize → 12, FontFamily → "Arial"]];
  probPlot5 = Overlay[{plot5, phiLegend}, Alignment → Right]];

Panel[Labeled[ProgressIndicator[Dynamic[it], {1, iter}], "Calculation Progress",
  Top, LabelStyle → Directive[FontSize → 12, Bold, FontFamily → "Arial"]]]

If[statTest > 1,
  avg = Mean[x2];
  stDev = N[StandardDeviation[x2]];
  groupNames = Union[Group];
  numGroups = Length[groupNames];
  groupPosns = Table[Flatten[Position[Group, groupNames[[i]], 1]], {i, kg}];
  x3 = Table[0.0, {n2}, {m2}];
  simRatios = Table[0.0, {iter}, {5}];

  lambda = Det[W] / Det[T];
  df1 = m2 * (kg - 1);
  wCoef = N[n2 - 1 - (m2 + kg) / 2];
  tCoef = N[√((df1^2 - 4) / (m2^2 + (kg - 1)^2 - 5))];
  df2 = (wCoef * tCoef) - (df1 / 2) + 1;
  Flambda = N[((1 - lambda^(1/tCoef)) / lambda^(1/tCoef)) * (df2 / df1)];

  rmr = eVals[[1]];
  d = Max[m2, kg - 1];
  df1 = m2;
  df2 = n2 - kg - d - 1;
  Frmr = (df2 / df1) * rmr;

  V = 0.0;
  Do[V = V + (eVals[[j]] / (1 + eVals[[j]])), {j, noVals}];
  s = Min[m2, kg - 1];
  d = Max[m2, kg - 1];
  df1 = s * d;
  df2 = s * (n2 - kg - m2 + s);
  FV = ((n2 - kg - m2 + s) * V) / (d (s - V));

  U = 0.0;
  Do[U = U + eVals[[j]], {j, noVals}];
  s = Min[m2, kg - 1];
  A = N[(Abs[kg - m2 - 1] - 1) / 2];
  B = N[(n2 - kg - m2 - 1) / 2];
  df1 = s * ((2 * A) + s + 1);
  df2 = 2 * ((s * B) + 1);
  FU = (df2 * U) / (s * df1);
```

```
phiTest = (n2 - 1 - (0.5 * (m2 + kg))) * (Log[(Det[T] / Det[W])]);
chi2Phi = phiTest;

Do[
 Label[123];
 If[statTest == 2,
  Do[
   Do[
    Do[
     inum = groupPosns[[k, i]];
     x3[[inum, j]] = RandomReal[NormalDistribution[avg[[j]], stDev[[j]]]],
     {i, smpSize[[k]]}], {j, m2}], {k, kg}]];
 If[statTest == 3,
  Do[x3[[i]] = N[x2[[RandomInteger[{1, n2}]]]], {i, n2}]];
 {gndMean, simT, gNames, nGps, gpMeans, smpSize, simW, simB} =
  TWB[x3, n2, m2, Group];

 simWI = PseudoInverse[simW];
 simCovar = simWI.simB;
 If[n2 > m2, mvecs = m2, mvecs = n2];
 If[eMethod == 1,
  simeVals = Eigenvalues[simCovar]];
 If[eMethod == 2,
  {w, u, v} = SingularValueDecomposition[simCovar, mvecs];
  simeVals = N[Diagonal[u], kg - 1]];
 simNoVals = Min[{nGps - 1, m2}];

 simlambda = Det[simW] / Det[simT];
 simdf1 = m2 * (kg - 1);
 simwCoef = N[n2 - 1 - (m2 + kg) / 2];
 simtCoef = N[Sqrt[(simdf1^2 - 4) / (m2^2 + (kg - 1)^2 - 5)]];
 simdf2 = (simwCoef * simtCoef) - (simdf1 / 2) + 1;
 simFlambda =
  N[((1 - simlambda^(1/simtCoef)) / simlambda^(1/simtCoef)) * (simdf2 / simdf1)];

 simrmr = simeVals[[1]];
 simd = Max[m2, kg - 1];
 simdf1 = m2;
 simdf2 = n2 - kg - d - 1;
 simFrmr = (simdf2 / simdf1) * simrmr;

 simV = 0.0;
 Do[simV = simV + (simeVals[[j]] / (1 + simeVals[[j]])), {j, simNoVals}];
 sims = Min[m2, kg - 1];
 simd = Max[m2, kg - 1];
```

```
 simdf1 = sims * simd;
 simdf2 = s * (n2 - kg - m2 + sims);
 simFV = ((n2 - kg - m2 + sims) * simV) / (simd (sims - simV));

 simU = 0.0;
 Do[simU = simU + simeVals[[j]], {j, simNoVals}];
 sims = Min[m2, kg - 1];
 simA = N[(Abs[kg - m2 - 1] - 1) / 2];
 simB = N[(n2 - kg - m2 - 1) / 2];
 simdf1 = sims * ((2 * simA) + sims + 1);
 simdf2 = 2 * ((sims * simB) + 1);
 simFU = (simdf2 * simU) / (sims * simdf1);

 simPhi = (n2 - 1 - (0.5 * (m2 + kg))) * (Log[(Det[simT] / Det[simW])]);

 simRatios[[it, 1]] = simFlambda;
 simRatios[[it, 2]] = simFrmr;
 simRatios[[it, 3]] = simFV;
 simRatios[[it, 4]] = simFU;
 simRatios[[it, 5]] = simPhi;
 Do[
  If[Head[simRatios[[it, j]]] == Complex, Goto[123]], {j, 5}], {it, iter}];

simRatiosT = Transpose[simRatios];
Do[simRatiosT[[j]] = Sort[simRatiosT[[j]]], {j, 5}];

simProb = Table[0.0, {5}, {3}];
simProb[[1, 1]] = lambda;
simProb[[2, 1]] = rmr;
simProb[[3, 1]] = V;
simProb[[4, 1]] = U;
simProb[[5, 1]] = phiTest;
simProb[[1, 2]] = Flambda;
simProb[[2, 2]] = Frmr;
simProb[[3, 2]] = FV;
simProb[[4, 2]] = FU;
simProb[[5, 2]] = chi2Phi;
Do[
 knt = 0;
 Do[If[simRatiosT[[j, i]] > simProb[[j, 2]], knt = knt + 1], {i, iter}];
 simProb[[j, 3]] = N[knt / iter] * 100, {j, 5}];
noBins = 40;

FTable = Table[" ", {4}, {2}];
FTable[[1, 1]] = "Wilk's Lambda";
FTable[[2, 1]] = "Observed F-Ratio";
FTable[[3, 1]] = "No. Iterations";
```

```
FTable[[4, 1]] = "Probability (%)";
FTable[[1, 2]] = PaddedForm[lambda, {4, 3}];
FTable[[2, 2]] = PaddedForm[Flambda, {5, 3}];
FTable[[3, 2]] = iter;
FTable[[4, 2]] = PaddedForm[simProb[[1, 3]], {5, 3}];
hLegend = Labeled[
   Grid[FTable, BaseStyle → Directive[FontSize → 10, FontFamily → "Arial"],
    Alignment → {{Left, Right}}, Frame → True,
    Dividers → {{True, True}, {True}}], "Wilk's λ Table", Top,
   LabelStyle → Directive[FontSize → 12, Bold, FontFamily → "Arial"]];
plot1 = Histogram[simRatiosT[[1]], noBins, ChartStyle → {Red},
   ImageSize → hSizeMVT, PlotRange → Automatic,
   LabelStyle → Directive[FontSize → 12, Black, FontFamily → "Arial"],
   AxesLabel → {"F Ratio", "Frequency"}, ImageSize → hSize];
probPlot1 = Overlay[{plot1, hLegend}, Alignment → Right];

FTable = Table[" ", {4}, {2}];
FTable[[1, 1]] = "Roy's Max. Root";
FTable[[2, 1]] = "Observed F-Ratio";
FTable[[3, 1]] = "No. Iterations";
FTable[[4, 1]] = "Probability (%)";
FTable[[1, 2]] = PaddedForm[rmr, {4, 3}];
FTable[[2, 2]] = PaddedForm[Frmr, {5, 3}];
FTable[[3, 2]] = iter;
FTable[[4, 2]] = PaddedForm[simProb[[2, 3]], {5, 3}];
hLegend = Labeled[
   Grid[FTable, BaseStyle → Directive[FontSize → 10, FontFamily → "Arial"],
    Alignment → {{Left, Right}}, Frame → True,
    Dividers → {{True, True}, {True}}], "Roy's Max. Root Table", Top,
   LabelStyle → Directive[FontSize → 12, Bold, FontFamily → "Arial"]];
plot2 = Histogram[simRatiosT[[2]], noBins, ChartStyle → {Magenta},
   ImageSize → hSizeMVT, PlotRange → Automatic,
   LabelStyle → Directive[FontSize → 12, Black, FontFamily → "Arial"],
   AxesLabel → {"F Ratio", "Frequency"}, ImageSize → hSizeMVT];
probPlot2 = Overlay[{plot2, hLegend}, Alignment → Right];

FTable = Table[" ", {4}, {2}];
FTable[[1, 1]] = "Pillai's Trace";
FTable[[2, 1]] = "Observed F-Ratio";
FTable[[3, 1]] = "No. Iterations";
FTable[[4, 1]] = "Probability (%)";
FTable[[1, 2]] = PaddedForm[V, {4, 3}];
FTable[[2, 2]] = PaddedForm[FV, {5, 3}];
FTable[[3, 2]] = iter;
FTable[[4, 2]] = PaddedForm[simProb[[3, 3]], {5, 3}];
hLegend = Labeled[
   Grid[FTable, BaseStyle → Directive[FontSize → 10, FontFamily → "Arial"],
```

```
    Alignment → {{Left, Right}}, Frame → True,
    Dividers → {{True, True}, {True}}], "Pillai's Trace Table", Top,
   LabelStyle → Directive[FontSize → 12, Bold, FontFamily → "Arial"]];
plot3 = Histogram[simRatiosT[[3]], noBins, ChartStyle → {Blue},
   ImageSize → hSizeMVT, PlotRange → Automatic,
   LabelStyle → Directive[FontSize → 12, Black, FontFamily → "Arial"],
   AxesLabel → {"F Ratio", "Frequency"}, ImageSize → hSizeMVT];
probPlot3 = Overlay[{plot3, hLegend}, Alignment → Right];

FTable = Table[" ", {4}, {2}];
FTable[[1, 1]] = "Lawes-Hotelling Trace";
FTable[[2, 1]] = "Observed F-Ratio";
FTable[[3, 1]] = "No. Iterations";
FTable[[4, 1]] = "Probability (%)";
FTable[[1, 2]] = PaddedForm[U, {4, 3}];
FTable[[2, 2]] = PaddedForm[FU, {5, 3}];
FTable[[3, 2]] = iter;
FTable[[4, 2]] = PaddedForm[simProb[[4, 3]], {5, 3}];
hLegend = Labeled[
   Grid[FTable, BaseStyle → Directive[FontSize → 10, FontFamily → "Arial"],
    Alignment → {{Left, Right}}, Frame → True, Dividers → {{True, True}, {True}}],
   "Lawes-Hotelling Trace Table", Top,
   LabelStyle → Directive[FontSize → 12, Bold, FontFamily → "Arial"]];
plot4 = Histogram[simRatiosT[[4]], noBins, ChartStyle → {Green},
   ImageSize → hSizeMVT, PlotRange → Automatic,
   LabelStyle → Directive[FontSize → 12, Black, FontFamily → "Arial"],
   AxesLabel → {"F-Ratio", "Frequency"}, ImageSize → hSizeMVT];
probPlot4 = Overlay[{plot4, hLegend}, Alignment → Right];

FTable = Table[" ", {4}, {2}];
FTable[[1, 1]] = "Log Likelihood Ratio (χ²)";
FTable[[2, 1]] = "Degrees of Freedom";
FTable[[3, 1]] = "Iterations";
FTable[[4, 1]] = "Probability (%)";
FTable[[1, 2]] = PaddedForm[phiTest, {4, 3}];
FTable[[2, 2]] = m2 * (kg - 1);
FTable[[3, 2]] = iter;
FTable[[4, 2]] = PaddedForm[simProb[[5, 3]], {5, 3}];
If[simProb[[5, 3]] ≥ 5.0, rng = phiTest * 2, rng = phiTest];
phiLegend = Labeled[
   Grid[FTable, BaseStyle → Directive[FontSize → 10, FontFamily → "Arial"],
    Alignment → {{Left, Right}}, Frame → True,
    Dividers → {{True, True}, {True}}], "Likelihood Ratio Table", Top,
   LabelStyle → Directive[FontSize → 12, Bold, FontFamily → "Arial"]];
plot5 = Histogram[simRatiosT[[5]], noBins, ChartStyle → {Yellow},
   ImageSize → hSizeMVT, PlotRange → Automatic,
   LabelStyle → Directive[FontSize → 12, Black, FontFamily → "Arial"],
```

```
    AxesLabel → {"χ² Ratio", "Frequency"}, ImageSize → hSizeMVT];
  probPlot5 = Overlay[{plot5, phiLegend}, Alignment → Right]];

label1 = "Parametric Probability Results";
label2 = "Monte Carlo Simulation Results";
label3 = "Bootstrap Modelling Results";
probGrid = Labeled[
  GraphicsGrid[{{probPlot1, probPlot2}, {probPlot3, probPlot4}, {probPlot5}},
   ImageSize → 700, AspectRatio → 1 / 0.80],
  ToExpression[StringJoin["label", ToString[statTest]]], Top,
  LabelStyle → Directive[FontSize → 18, Bold, FontFamily → "Arial"]]
```

Out[•]= **Calculation Progress**

Export mean vector test results

```
In[•]:= filenameout = SystemDialogInput["FileSave"];
Export[filenameout, probGrid, "TIFF", ImageResolution → 150]
```

```
Out[•]= /Users/n.macleod/Desktop/Africa-Spain/CVA Results/Mean Vector Tests (BS).tif
```

Export list of simulated mean vector test index values

```
simRatiosOut = Table[" ", {iter + 1}, {5}];
simRatiosOut[[1, 1]] = "Wilk's λ";
simRatiosOut[[1, 2]] = "Roys Maximum Root";
simRatiosOut[[1, 3]] = "Pillai's Trace";
simRatiosOut[[1, 4]] = "Laws-Hotelling Trace";
simRatiosOut[[1, 5]] = "Likelihood Ratio";
Do[simRatiosOut[[i + 1, j]] = simRatiosT[[j, i]], {i, iter}, {j, 5}]

filenameout = SystemDialogInput["FileSave"];
Export[filenameout, simRatiosOut, "CSV", "TextDelimiters" → ""]
```

Export CVA results.

Export canonical variate eigenvalues.

```
In[•]:= filenameout = SystemDialogInput["FileSave"];
Export[filenameout, t2, "CSV", "TextDelimiters" → ""]
```

Export canonical variate loadings.

```
In[•]:= filenameout = SystemDialogInput["FileSave"];
Export[filenameout, eVecs, "CSV", "TextDelimiters" → ""]
```

Export canonical variate scores.

```
In[•]:= outScores = Table[" ", {n2 + 1}, {noVals + 2}];
outScores[[1, 1]] = "Object";
outScores[[1, 2]] = "Group";
Do[outScores[[1, j + 2]] = StringJoin["CV-", ToString[j]], {j, noVals}]
Do[outScores[[i + 1, 1]] = objNames[[i]], {i, n2}]
Do[outScores[[i + 1, 2]] = Group[[i]], {i, n2}]
Do[outScores[[i + 1, j + 2]] = eScores[[i, j]], {i, n2}, {j, noVals}]

filenameout = SystemDialogInput["FileSave"];
Export[filenameout, outScores, "CSV", "TextDelimiters" → ""]
```

Calculate discriminant performance measures.

Calculate distance table.

```
In[•]:= gpCVMeans = Table[0.0, {nGps}, {noVals}];
mDist = Table[0.0, {n2}, {nGps}];
distTable = Table[0.0, {n2 + 1}, {nGps + 2}];
distTableOut = Table[0.0, {n2 + 1}, {nGps + 2}];
eScrs = Take[eScores, All, noVals];

Do[
 Do[
   sum = 0.0;
   Do[
    If[Group[[i]] == gNames[[k]],
     sum = sum + eScores[[i, j]],
     sum = sum],
    {i, n2}];
   gpCVMeans[[k, j]] = sum / smpSize[[k]],
   {j, noVals}];,
 {k, nGps}]
gpCVMeans;

Do[
 Do[
  mDist[[i, k]] = EuclideanDistance[gpCVMeans[[k]], eScrs[[i]]],
```

```
   {i, n2}],
  {k, nGps}]
 mDist;

 pDist = mDist;
 Do[
  n = 0;
  ref = Min[pDist[[k]]];
  Do[
   If[pDist[[k, j]] == ref, pDist[[k, j]] = Style[pDist[[k, j]], Bold]],
   {j, nGps}],
  {k, n2}]

 distTable[[1, 1]] = "Object";
 distTableOut[[1, 1]] = "Object";
 distTable[[1, 2]] = "Group";
 distTableOut[[1, 2]] = "Group";
 Do[distTable[[1, j + 2]] = Text[Rotate[gNames[[j]], 90 Degree]], {j, nGps}]
 Do[distTableOut[[1, j + 2]] = gNames[[j]], {j, nGps}]
 Do[
  distTable[[i + 1, 1]] = objNames[[i]];
  distTableOut[[i + 1, 1]] = objNames[[i]];
  distTable[[i + 1, 2]] = Group[[i]];
  distTableOut[[i + 1, 2]] = Group[[i]];
  Do[distTable[[i + 1, j + 2]] = PaddedForm[pDist[[i, j]], {4, 3}], {j, nGps}];
  Do[distTableOut[[i + 1, j + 2]] = mDist[[i, j]], {j, nGps}],
  {i, n2}]

 distances = Labeled[Grid[distTable, BaseStyle → (FontFamily → "Arial"),
     Alignment → {{Left, Center, Center, Center, Center, Center, Center, Center},
       {Bottom, Baseline}}, Frame → True,
     Dividers → {{True, True, True}, {True, True}}], "Distance Table", Top,
   LabelStyle → Directive[Black, Bold, FontSize → 14, FontFamily → "Arial"]]
```

Export group distance table.

In[•]:=
```
filenameout = SystemDialogInput["FileSave"];
Export[filenameout, distTableOut, "CSV", "TextDelimiters" → ""]
```

Calculate confusion matrix.

In[•]:=
```
cMat = Table[0, {nGps}, {nGps}];
gPos = Table[Flatten[Position[Group, gNames[[i]], 1]], {i, nGps}];
pDist = mDist;

Do[
 Do[
```

```
   Do[
    gPos[[k, ig]];
    pDist[[gPos[[k, ig]]]];
    ref = Min[pDist[[gPos[[k, ig]]]]];
    pDist[[gPos[[k, ig]], j]];
    If[pDist[[gPos[[k, ig]], j]] == ref,
     cMat[[k, j]] = cMat[[k, j]] + 1, cMat[[k, j]] = cMat[[k, j]]],
    {j, nGps}],
   {ig, smpSize[[k]]}],
  {k, nGps}]
cMat;

cmTable = Table[0, {nGps + 4}, {nGps + 4}];
cmOut = Table[0, {nGps + 4}, {nGps + 4}];
cmTable[[1, 1]] = "Groups";
cmOut[[1, 1]] = "Groups";
Do[cmTable[[1, j + 1]] = Text[Rotate[gNames[[j]], 90 Degree]], {j, nGps}];
Do[cmOut[[1, j + 1]] = gNames[[j]], {j, nGps}];
Do[cmTable[[j + 1, 1]] = gNames[[j]], {j, nGps}];
Do[cmOut[[j + 1, 1]] = gNames[[j]], {j, nGps}];
cmTable[[nGps + 2, 1]] = "Total Correct";
cmOut[[nGps + 2, 1]] = "Total Correct";
cmTable[[nGps + 3, 1]] = "Total Estimated";
cmOut[[nGps + 3, 1]] = "Total Estimated";
cmTable[[nGps + 4, 1]] = "Percent Estimated Correctly";
cmOut[[nGps + 4, 1]] = "Percent Estimated Correctly";
cmTable[[1, nGps + 3]] = Text[Rotate["Group Totals", 90 Degree]];
cmOut[[1, nGps + 3]] = "Group Totals";
cmTable[[1, nGps + 2]] = Text[Rotate["Total Correct", 90 Degree]];
cmOut[[1, nGps + 2]] = "Total Correct";
cmTable[[1, nGps + 4]] = Text[Rotate["Percent Correct", 90 Degree]];
cmOut[[1, nGps + 4]] = "Percent Correct";
Do[cmTable[[i + 1, j + 1]] = cMat[[i, j]], {i, nGps}, {j, nGps}];
Do[cmOut[[i + 1, j + 1]] = cMat[[i, j]], {i, nGps}, {j, nGps}];
cTot = Total[cMat];
rTot = Diagonal[cMat];
gpTot = Total[cMat, {2}];
gTot = Total[cTot];

Do[cmTable[[i + 1, nGps + 2]] = rTot[[i]], {i, nGps}];
Do[cmOut[[i + 1, nGps + 2]] = rTot[[i]], {i, nGps}];
Do[cmTable[[i + 1, nGps + 3]] = gpTot[[i]], {i, nGps}];
Do[cmOut[[i + 1, nGps + 3]] = gpTot[[i]], {i, nGps}];
Do[cmTable[[i + 1, nGps + 4]] =
   PaddedForm[N[(rTot[[i]] / gpTot[[i]]) * 100.0], {4, 2}], {i, nGps}];
Do[cmOut[[i + 1, nGps + 4]] = N[(rTot[[i]] / gpTot[[i]]) * 100.0], {i, nGps}];
cmTable[[nGps + 2, nGps + 3]] = gTot;
```

```
cmOut[[nGps + 2, nGps + 3]] = gTot;
cmTable[[nGps + 2, nGps + 2]] = Total[rTot];
cmOut[[nGps + 2, nGps + 2]] = Total[rTot];
cmTable[[nGps + 2, nGps + 4]] =
  PaddedForm[N[(Total[rTot] / gTot) * 100.0], {4, 2}];
cmOut[[nGps + 2, nGps + 4]] = N[(Total[rTot] / gTot) * 100.0];

Do[cmTable[[nGps + 2, i + 1]] = rTot[[i]], {i, nGps}]
Do[cmOut[[nGps + 2, i + 1]] = rTot[[i]], {i, nGps}]
Do[cmTable[[nGps + 3, i + 1]] = cTot[[i]], {i, nGps}]
Do[cmOut[[nGps + 3, i + 1]] = cTot[[i]], {i, nGps}]
Do[cmTable[[nGps + 4, i + 1]] =
   PaddedForm[N[(rTot[[i]] / cTot[[i]]) * 100.0], {4, 2}], {i, nGps}];
Do[cmOut[[nGps + 4, i + 1]] = N[(rTot[[i]] / cTot[[i]]) * 100.0], {i, nGps}];
cmTable[[nGps + 3, nGps + 2]] = Total[cTot];
cmOut[[nGps + 3, nGps + 2]] = Total[cTot];
cmTable[[nGps + 4, nGps + 2]] =
  PaddedForm[N[(Total[rTot] / Total[cTot]) * 100.0], {4, 2}];
cmOut[[nGps + 4, nGps + 2]] = N[(Total[rTot] / Total[cTot]) * 100.0];

cmTable[[nGps + 3, nGps + 3]] = " ";
cmTable[[nGps + 3, nGps + 4]] = " ";
cmTable[[nGps + 4, nGps + 3]] = " ";
cmTable[[nGps + 4, nGps + 4]] = " ";
cmOut[[nGps + 3, nGps + 3]] = " ";
cmOut[[nGps + 3, nGps + 4]] = " ";
cmOut[[nGps + 4, nGps + 3]] = " ";
cmOut[[nGps + 4, nGps + 4]] = " ";

confusion = Labeled[Grid[cmTable, Frame → True,
   Dividers → {{2 → True, -4 → True}, {2 → True, -4 → True}},
   BaseStyle → (FontFamily → "Arial"),
   Alignment → {{Right, Center}, {Bottom, Baseline}}], "Raw Confusion Matrix",
  Top, LabelStyle → Directive[FontSize → 14, Bold, FontFamily → "Arial"]]
```

Export confusion matrix table.

In[•]:=
```
filenameout = SystemDialogInput["FileSave"];
Export[filenameout, cmOut, "CSV", "TextDelimiters" → ""]
```

Modelling Section (optional).

Calculate along – axis models.

Specify axis modelling options.

In[•]:=
```
Panel[Labeled[Row[{Panel[Labeled[InputField[Dynamic[modelAxes], FieldSize → 5],
      "Enter no. of axes to be modeled.", Top,
      LabelStyle → Directive[FontSize → 12, FontFamily → "Arial"]]], , ,
    Panel[Labeled[InputField[Dynamic[modelsPerAxis], FieldSize → 5],
      "Enter no. models to be plotted per axis.", Top,
      LabelStyle → Directive[FontSize → 12, FontFamily → "Arial"]]]}],
  "Along-Axis Modeling Parameters", Top, LabelStyle →
   Directive[FontSize → 16, Bold, FontFamily → "Arial"]]]
modelsPerAxis = 5; modelAxes = noAxes;
```

Out[•]=

Along–Axis Modeling Parameters

Enter no. of axes to be modeled.	Enter no. models to be plotted per axis.
3	5

Perform axis model location calculations.

In[•]:=
```
modSet = 1;
models = Table[0.0, {modelsPerAxis}, {modelAxes}];
modTable = Table[0.0, {modelAxes}, {modelsPerAxis}, {m2}];

scrMeans = Mean[eScores];

eScoresT = Transpose[eScores];
Do[
  xMax = Max[eScoresT[[k]]];
  xMin = Min[eScoresT[[k]]];
  xRng = xMax - xMin;
  xInt = xRng / (modelsPerAxis - 1);
  xBase = xMin - xInt;
  Do[models[[i, k]] = xBase + (xInt * i), {i, modelsPerAxis}];
  Do[modTable[[k, i, k]] = xBase + (xInt * i), {i, modelsPerAxis}],
  {k, modelAxes}];
modTable = Partition[Flatten[Chop[modTable]], m2];

If[meanTrans == 1,
 Do[
  If[modTable[[i, j]] == 0,
   modTable[[i, j]] = scrMeans[[j]]], {i, modelsPerAxis * modelAxes}, {j, m2}]]
```

Calculate group-difference models.

Specify group – difference modelling options.

In[∘]:=
```
Panel[Labeled[Panel[Labeled[InputField[Dynamic[modelsPerAxis], FieldSize → 5],
    "Enter no. models to be plotted per axis.", Top,
    LabelStyle → Directive[FontSize → 12, FontFamily → "Arial"]]],
  "Group-Difference Modeling Parameters", Top,
  LabelStyle → Directive[FontSize → 16, Bold, FontFamily → "Arial"]]]
modelsPerAxis = 5;
```

Out[∘]=

Group–Difference Modeling Parameters

Enter no. models to be plotted per axis.

5

Perform group – difference model location calculations.

In[∘]:=
```
modSet = 2;
groupNames = Union[Group];
numGroups = Length[groupNames];
numCV = numGroups - 1;
groupPosns =
  Table[Flatten[Position[Group, groupNames[[i]], 1]], {i, numGroups}];
cVariates = Take[eScores, All, numCV];

meanCV = Table[0.0, {numGroups}, {numCV}];
Do[
  ng = Length[groupPosns[[k]]];
  gp = Table[0.0, {ng}, {numCV}];
  Do[gp[[i, j]] = cVariates[[groupPosns[[k, i]], j]], {i, ng}, {j, numCV}];
  meanCV[[k]] = Mean[gp], {k, numGroups}];

meanCV2 = Append[meanCV, meanCV[[1]]];
models = Table[0.0, {numGroups}, {modelsPerAxis}, {numCV}];
modTable = Table[0.0, {numGroups * modelsPerAxis}, {m2}];
Do[
  dif = meanCV2[[k + 1]] - meanCV2[[k]];
  dif = dif / (modelsPerAxis - 1);
  models[[k, 1]] = meanCV[[k]];
  Do[models[[k, i + 1]] = models[[k, 1]] + (dif * i), {i, modelsPerAxis - 1}],
  {k, numGroups}];
models = Partition[Flatten[models], numGroups - 1];
Do[modTable[[i, j]] = models[[i, j]], {i, numGroups * modelsPerAxis}, {j, numCV}];
```

Calculate planar subspace models

Specify subspace modelling options.

In[∘]:=
```
Panel[
 Labeled[Column[{Row[{Panel[Labeled[PopupMenu[Dynamic[xAxisName], cvNames],
        "Select canonical variate to be modeled on x-axis.", Top,
        LabelStyle → Directive[FontSize → 12, FontFamily → "Arial"]]], , ,
      Panel[Labeled[InputField[Dynamic[xModelNum], FieldSize → 5],
        "Enter no. of models along the x-axis.", Top,
        LabelStyle → Directive[FontSize → 12, FontFamily → "Arial"]]]}],
    Row[{Panel[Labeled[PopupMenu[Dynamic[yAxisName], cvNames],
        "Select canonical variate to be modeled on y-axis.", Top,
        LabelStyle → Directive[FontSize → 12, FontFamily → "Arial"]]], , ,
      Panel[Labeled[InputField[Dynamic[yModelNum], FieldSize → 5],
        "Enter no. of models along the y-axis.", Top,
        LabelStyle → Directive[FontSize → 12, FontFamily → "Arial"]]]}]}],
  "Subspace Modeling Parameters", Top, LabelStyle →
   Directive[FontSize → 18, Bold, FontFamily → "Arial"]]]
xAxisName = cvNames[[1]];
yAxisName = cvNames[[2]];
xModelNum = 5;
yModelNum = 4;
```

Out[∘]=

Subspace Modeling Parameters

Select canonical variate to be modeled on x–axis.<br>PopupMenu[PC–1, Table[CV–<>ToString[i], {i, noAxes}]]	Enter no. of models along the x–axis.<br>5
Select canonical variate to be modeled on y–axis.<br>PopupMenu[PC–2, Table[CV–<>ToString[i], {i, noAxes}]]	Enter no. of models along the y–axis.<br>4

Perform subspace model location calculations

In[•]:=
```
modSet = 3;
models = Table[0.0, {yModelNum}, {xModelNum}, {2}];
eScoresT = Transpose[eScores];
Do[If[xAxisName == cvNames[[j]], axis1 = j], {j, noAxes}]
Do[If[yAxisName == cvNames[[j]], axis2 = j], {j, noAxes}]
xMax = Max[eScoresT[[axis1]]];
xMin = Min[eScoresT[[axis1]]];
xRng = xMax - xMin;
xInt = xRng / (xModelNum - 1);
yMax = Max[eScoresT[[axis2]]];
yMin = Min[eScoresT[[axis2]]];
yRng = yMax - yMin;
yInt = yRng / (yModelNum - 1);

xBase = xMin - xInt;
yBase = yMin - yInt;
Do[
 yVal = yBase + (yInt * j);
 Do[
  models[[j, i, 1]] = xBase + (xInt * i);
  models[[j, i, 2]] = yVal, {i, xModelNum}], {j, yModelNum}]
models = Partition[Flatten[Reverse[models]], 2];

nrows = xModelNum * yModelNum;
modTable = Table[0.0, {nrows}, {m2}];
Do[
  modTable[[i, axis1]] = mTable[[i, 1]];
  modTable[[i, axis2]] = mTable[[i, 2]], {i, nrows}];
```

Import external set of model coordinates
(total must match along – axis model specifications [above] and contain the complete variable set).

```
filenamein = SystemDialogInput["FileOpen"];
modTable = Import[filenamein, "CSV"];
```

Plot model coordinates in the CV space.

Specify 2 D plot options.
You must run this code after you read in the data so it can pick up the proper variable names.

```
In[•]:= cvNames = Table[StringJoin["CV-", ToString[i]], {i, noAxes}];
Panel[Labeled[Column[
   {Panel[Labeled[RadioButtonBar[Dynamic[modSet], {1 → "Along-axis Models",
        2 → "Group-Difference Models", 3 → "Subspace Models"}],
      "Enter type of model set.", Top, LabelStyle →
       Directive[FontSize → 12, Bold, FontFamily → "Arial"]]],
    Row[{Panel[Labeled[PopupMenu[Dynamic[xAxisName], cvNames],
        "Select variable to be plotted on x-axis.", Top, LabelStyle →
         Directive[FontSize → 12, Bold, FontFamily → "Arial"]]], "  ",
      Panel[Labeled[PopupMenu[Dynamic[yAxisName], cvNames],
        "Select variable to be plotted on y-axis.", Top,
        LabelStyle → Directive[FontSize → 12, Bold, FontFamily → "Arial"]]]}],
    Row[{
      Panel[Labeled[PopupMenu[Dynamic[pltAspect],
          {1 → "Golden Ratio Plot", 2 → "Square Plot (equi-length axes)",
           3 → "True-Scale Plot (actual axis scales)"}],
        "Enter plot aspect ratio type.", Top, LabelStyle →
         Directive[FontSize → 12, Bold, FontFamily → "Arial"]]], "  ",
      Panel[Labeled[PopupMenu[Dynamic[lch], {1 → "Simple scatterplot",
           2 → "Scatterplot w/ convex hulls"}],
        "Show group domians?", Top, LabelStyle →
         Directive[FontSize → 12, Bold, FontFamily → "Arial"]]], "  ",
      Panel[Labeled[PopupMenu[Dynamic[ptsJoin], {1 → "No", 2 → "Yes"}],
        "Join datapoints?", Top,
        LabelStyle → Directive[FontSize → 12, Bold, FontFamily → "Arial"]]]}],
    Row[{Panel[Labeled[InputField[Dynamic[pltSz], FieldSize → 5],
        "Enter plot size value.", Top, LabelStyle →
         Directive[FontSize → 12, Bold, FontFamily → "Arial"]]], "  ",
      Panel[Labeled[InputField[Dynamic[iconSizeMods], FieldSize → 5],
        "Enter plot icon size value.", Top, LabelStyle →
         Directive[FontSize → 12, Bold, FontFamily → "Arial"]]], "  ",
      Panel[Labeled[InputField[Dynamic[pltPad], FieldSize → 5],
        "Enter plot margin padding value.", Top,
        LabelStyle → Directive[FontSize → 12, Bold, FontFamily → "Arial"]]]}]},
   Center], "Model Coordinate Options", Top, LabelStyle →
   Directive[FontSize → 18, Bold, FontFamily → "Arial"]]]
pltSize = 500; iconSizeMods = 0.03; pltPad = 0.1;
xAxisName = cvNames[[1]];
pltSz = 500; yAxisName = cvNames[[2]]; ptsJoin = 1;
dataTrans = 1; pltAspect = 1 ;
lch = 2;
```

Out[•]=

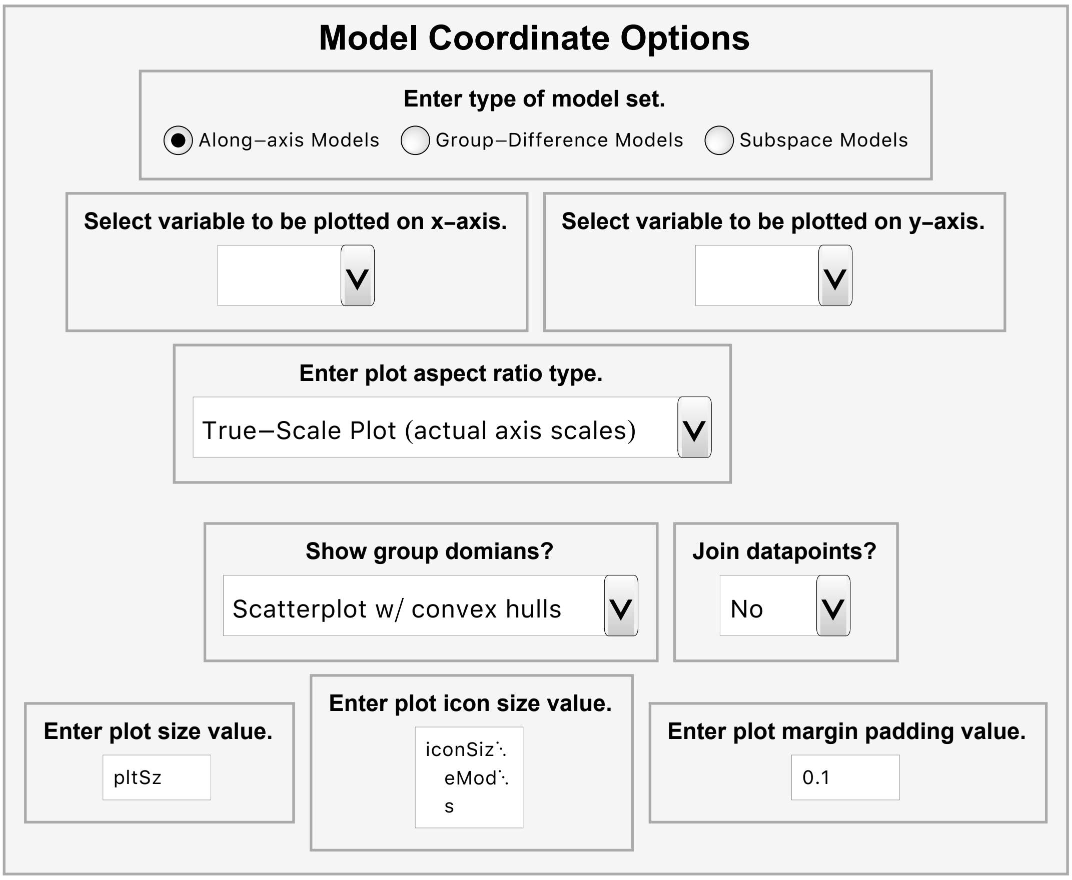

Plot model coordinates in space of a CVA plane (optional).

In[•]:=

```
axisPlots = Table[" ", {2}];
Do[If[xAxisName == cvNames[[j]], axis1 = j], {j, noAxes}]
Do[If[yAxisName == cvNames[[j]], axis2 = j], {j, noAxes}]

If[modSet == 1,
  nrows = modelsPerAxis;
  modpts = Table[0.0, {2}, {nrows}, {2}];
  Do[modpts[[1, i, j]] = scrMeans[[axis2]], {i, nrows}, {j, 2}];
  Do[modpts[[2, i, j]] = scrMeans[[axis1]], {i, nrows}, {j, 2}];
  Do[modpts[[1, i, 1]] = models[[i, axis1]], {i, nrows}];
  Do[modpts[[2, i, 2]] = models[[i, axis2]], {i, nrows}];
 ];

If[modSet == 2,
  nrows = numGroups * modelsPerAxis;
  modpts = models];

If[modSet == 3,
  nrows = numGroups * modelsPerAxis;
  modpts = models];

groupNames = Union[Group];
```

```
numGroups = Length[groupNames];
numCV = numGroups - 1;
groupPosns =
  Table[Flatten[Position[Group, groupNames[[i]], 1]], {i, numGroups}];
eScoresT = Transpose[eScores];

xAxis = eScoresT[[axis1]]; yAxis = eScoresT[[axis2]];
lab1 = StringJoin[{"Canonical Variate ", ToString[axis1]},
   {" (Var. =", ToString[t3[[axis1 + 1, 3]]]}, {"%)"}];
lab2 = StringJoin[{"Canonical Variate ", ToString[axis2]},
   {" (Var. =", ToString[t3[[axis2 + 1, 3]]]}, {"%)"}];
maxx =
 Max[
  xAxis];
minx = Min[xAxis];
maxy = Max[yAxis];
miny = Min[yAxis];

If[pltAspect == 1 || pltAspect == 3,
  xPlotLow = minx; xPlotHi = maxx; yPlotLow = miny; yPlotHi = maxy];
If[pltAspect == 2,
  If[minx > miny,
   xPlotLow = miny; yPlotLow = miny,
   xPlotLow = minx; yPlotLow = minx ]];
If[pltAspect == 2,
  If[maxx < maxy,
   xPlotHi = maxy; yPlotHi = maxy,
   xPlotHi = maxx; yPlotHi = maxx]];
If[pltAspect == 1, aRatio = 1 / N[GoldenRatio]];
If[pltAspect == 2, aRatio = 1];
If[pltAspect == 3, aRatio = Automatic];

tmpPoints = Transpose[List[xAxis, yAxis]];
pltPoints = Table[tmpPoints[[groupPosns[[j]]]], {j, numGroups}];
iconList = Flatten[Table[
    {Graphics[{EdgeForm[{Thickness[0.005], Black}],
       Hue[N[((numGroups + 1) - j) / numGroups]],
       Disk[{0, 0}, Scaled[iconSizeMods]]}]}, {j, numGroups}]];

If[lch == 1 || ptsJoin == 1,
  Do[
   pltTable[[k, 1]] = pTmp = ListPlot[pltPoints[[k]],
      AspectRatio → aRatio, Frame → True, Joined → False, Axes → False,
      PlotRange → {{xPlotLow, xPlotHi}, {yPlotLow, yPlotHi}}, PlotRangePadding →
       Scaled[pltPad], Ticks → Automatic, FrameLabel → {lab1, lab2},
      PlotMarkers → iconList[[k]], ImageSize → pltSize, LabelStyle →
       Directive[FontSize → 14, Black, FontFamily → "Arial"]], {k, kg}],
```

```
  Do[
   pltTable[[k, 1]] =
    ListPlot[pltPoints[[k]], Frame → True, Axes → False, AspectRatio → aRatio,
     PlotRange → {{xPlotLow, xPlotHi}, {yPlotLow, yPlotHi}}, PlotRangePadding →
      Scaled[pltPad], Ticks → Automatic, FrameLabel → {lab1, lab2},
     LabelStyle → Directive[Black, FontSize → 14, FontFamily → "Arial"],
     ImageSize → pltSize, PlotStyle → Directive[Disk[],
       Hue[N[((+1) - k) / kg]], EdgeForm[{Thickness[1.0], Black}],
       PointSize[Scaled[iconSize - 0.009]]]], {k, kg}]];

If[ptsJoin == 2,
  Do[
   pltTable[[k, 2]] = ListLinePlot[pltPoints[[k]],
     AspectRatio → aRatio, Frame → True, Joined → True, Axes → False,
     PlotStyle → Directive[Hue[N[((+1) - k) / kg]], Thin],
     PlotRange → {{xPlotLow, xPlotHi}, {yPlotLow, yPlotHi}},
     PlotRangePadding → Scaled[pltPad], Ticks → Automatic,
     FrameLabel → {lab1, lab2}, ImageSize → pltSize, LabelStyle →
      Directive[FontSize → 14, Black, FontFamily → "Arial"]], {k, kg}]];

If[lch == 2,
  Do[
   hull = ConvexHullMesh[pltPoints[[k]]];
   pltTable[[k, 3]] =
    HighlightMesh[hull, Style[2, Opacity[0.2], Hue[N[((+1) - k) / kg]]],
     Frame → True, Axes → False, AspectRatio → aRatio,
     PlotRange → {{xPlotLow, xPlotHi}, {yPlotLow, yPlotHi}}, PlotRangePadding →
      Scaled[pltPad], Ticks → Automatic, FrameLabel → {lab1, lab2},
     LabelStyle → Directive[Black, FontSize → 14, FontFamily → "Arial"],
     ImageSize → pltSize], {k, kg}]];

If[ptsJoin == 1 && lch == 1, p0 = Show[pltTable[[All, 1]]]];
If[ptsJoin == 2 && lch == 1, p0 = Show[pltTable[[All, 2]], pltTable[[All, 1]]]];
If[ptsJoin == 1 && lch == 2, p0 = Show[pltTable[[All, 3]], pltTable[[All, 1]]]];
If[ptsJoin == 2 && lch == 2,
  p0 = Show[pltTable[[All, 3]], pltTable[[All, 2]], pltTable[[All, 1]]]];

iconList = Flatten[Table[
    {Graphics[{EdgeForm[{Thin, Black}],
       Black, Disk[{0, 0}, Scaled[iconSizeMods / 2]]}]}, {j, 1}]];

If[modSet == 1,
  Do[
   axisPlots[[i]] = ListPlot[modpts[[i]], AspectRatio → aRatio,
     Frame → True, Joined → True, Axes → False, PlotRange → All,
     PlotStyle → Directive[Black, AbsoluteThickness[0.8]],
     PlotRangePadding → Scaled[pltPad], Ticks → Automatic,
```

```
      FrameLabel → {lab1, lab2}, PlotMarkers → iconList], {i, 2}];
   p4 = Show[axisPlots]];
If[modSet == 2,
   p4 = ListPlot[modpts, AspectRatio → aRatio,
      Frame → True, Joined → True, Axes → False, PlotRange → All,
      PlotStyle → Directive[Black, AbsoluteThickness[0.8]],
      PlotRangePadding → Scaled[pltPad], Ticks → Automatic,
      FrameLabel → {lab1, lab2}, PlotMarkers → iconList]];
If[modSet == 3,
   subSpacePlot = ListPlot[modpts, AspectRatio → aRatio,
      Frame → True, Joined → False, Axes → False, PlotRange → All,
      PlotStyle → Directive[Black, AbsoluteThickness[0.8]],
      PlotRangePadding → Scaled[pltPad], Ticks → Automatic,
      FrameLabel → {lab1, lab2}, PlotMarkers → iconList];
   p4 = subSpacePlot];
p1 = Labeled[Show[p0, p4], "           CV Score Plot", Top,
    LabelStyle → Directive[FontSize → 18, Bold, FontFamily → "Arial"]];
g1 = Grid[Table[
      {Graphics[{EdgeForm[{Thin, Black}], Hue[N[((numGroups + 1) - j) / numGroups]],
         Disk[]}]}, {j, numGroups}], Frame → False, ItemSize → 0.9];
g2 = Grid[Partition[groupNames, 1], Alignment → Left,
    BaseStyle → {FontFamily → "Arial", FontSize → 13, Italic}];
p2 = Labeled[Text[Grid[{{g1, g2}}, Alignment → Bottom, Frame → True]], "Legend",
    Top, LabelStyle → Directive[Black, FontSize → 18, Bold, FontFamily → "Arial"]];

plt2D = Grid[{{p1, p2}}, BaselinePosition → Top, Alignment → Top]
```

Export plot (optional).

In[∘]:=
```
filenameout = SystemDialogInput["FileSave"];
Export[filenameout, Plt2D, "TIFF", ImageResolution → 150]
```

Export model coordinates in the CV space.

In[∘]:=
```
If[modSet ≤ 2, partNum = noAxes, partNum = 2];
modelsOut = Take[Partition[Flatten[modTable], m2], All, partNum];
filenameout = SystemDialogInput["FileSave"];
Export[filenameout, modelsOut, "CSV", "TextDelimiters" → ""]
```

Back – project the model coordinates into the space of the orginal variables.

In[∘]:=
```
eVecsInv = Inverse[eVecsTotal];
modMatrix = modTable;
modCoords = modMatrix.eVecsInv;
```

Plot model coordinates in the space of the original variables (optional).

Specify 2 D plot options.
You must run this code after you read in the data so it can pick up the proper variable names.

```
In[•]:= Panel[Labeled[Column[
    {Panel[Labeled[RadioButtonBar[Dynamic[modSet], {1 → "Along-Axis Models",
         2 → "Group-Difference Models", 3 → "Subspace models"}],
       "Enter type of model set.", Top, LabelStyle →
        Directive[FontSize → 12, Bold, FontFamily → "Arial"]]],
     Row[{Panel[Labeled[PopupMenu[Dynamic[xAxisName], varNames],
         "Select variable to be plotted on x-axis.", Top, LabelStyle →
          Directive[FontSize → 12, Bold, FontFamily → "Arial"]]], "  ",
       Panel[Labeled[PopupMenu[Dynamic[yAxisName], varNames],
         "Select variable to be plotted on y-axis.", Top,
         LabelStyle → Directive[FontSize → 12, Bold, FontFamily → "Arial"]]]}],
     Row[{
       Panel[Labeled[PopupMenu[Dynamic[pltAspect],
          {1 → "Golden Ratio Plot", 2 → "Square Plot (equi-length axes)",
           3 → "True-Scale Plot (actual axis scales)"}],
         "Enter plot aspect ratio type.", Top, LabelStyle →
          Directive[FontSize → 12, Bold, FontFamily → "Arial"]]], "  ",
       Panel[Labeled[PopupMenu[Dynamic[lch], {1 → "Simple scatterplot",
           2 → "Scatterplot w/ convex hulls"}],
         "Show group domians?", Top, LabelStyle →
          Directive[FontSize → 12, Bold, FontFamily → "Arial"]]], "  ",
       Panel[Labeled[PopupMenu[Dynamic[ptsJoin], {1 → "No", 2 → "Yes"}],
         "Join datapoints?", Top,
         LabelStyle → Directive[FontSize → 12, Bold, FontFamily → "Arial"]]]}],
     Row[{Panel[Labeled[InputField[Dynamic[pltSz], FieldSize → 5],
         "Enter plot size value.", Top, LabelStyle →
          Directive[FontSize → 12, Bold, FontFamily → "Arial"]]], "  ",
       Panel[Labeled[InputField[Dynamic[iconSize], FieldSize → 5],
         "Enter plot icon size value.", Top, LabelStyle →
          Directive[FontSize → 12, Bold, FontFamily → "Arial"]]], "  ",
       Panel[Labeled[InputField[Dynamic[pltPad], FieldSize → 5],
         "Enter plot margin padding value.", Top,
         LabelStyle → Directive[FontSize → 12, Bold, FontFamily → "Arial"]]]}]},
    Center], "Model Coordinate Options", Top, LabelStyle →
    Directive[FontSize → 18, Bold, FontFamily → "Arial"]]]
pltSize = 500; iconSize = 0.03; pltPad = 0.1;
xAxisName = varNames[[1]];
pltSz = 500; yAxisName = varNames[[2]]; ptsJoin = 1;
dataTrans = 1; pltAspect = 1 ;
lch = 2;
```

Out[∘]=

Model Coordinate Options

Enter type of model set.

(●) Along–Axis Models ( ) Group–Difference Models ( ) Subspace models

Select variable to be plotted on x–axis.

PC–1 ∨

Select variable to be plotted on y–axis.

PC–2 ∨

Enter plot aspect ratio type.

True–Scale Plot (actual axis scales) ∨

Show group domians?

Scatterplot w/ convex hulls ∨

Join datapoints?

No ∨

Enter plot size value.

pltSz

Enter plot icon size value.

30.

Enter plot margin padding value.

0.1

Plot model coordinates in space of the original variables (optional).

In[∘]:=

```
If[modSet == 1, modpts = Table[0.0, {numGroups * modelsPerAxis}, {2}]];
If[modSet == 2, modpts = Table[0.0, {numGroups * modelsPerAxis}, {2}]];
If[modSet == 3, modpts = Table[0.0, {xModelNum * yModelNum}, {2}]];
Do[If[xAxisName == varNames[[j]], axis1 = j], {j, m2}];
Do[If[yAxisName == varNames[[j]], axis2 = j], {j, m2}];

If[modSet == 1,
 axisPlots = Table[" ", {modelAxes}];
 modptsProj = Partition[modCoords, modelsPerAxis];
 modpts = Table[0.0, {modelAxes}, {modelsPerAxis}, {2}];
 Do[
  modpts[[k, i, 1]] = modptsProj[[k, i, axis1]];
  modpts[[k, i, 2]] = modptsProj[[k, i, axis2]],
  {i, modelsPerAxis}, {j, 2}, {k, modelAxes}]]

If[modSet == 2, modpts = Table[0.0, {numGroups * modelsPerAxis}, {2}]];
If[modSet == 3, modpts = Table[0.0, {xModelNum * yModelNum}, {2}]];
Do[If[xAxisName == varNames[[j]], axis1 = j], {j, m2}];
Do[If[yAxisName == varNames[[j]], axis2 = j], {j, m2}];

If [modSet == 2,
 Do[modpts[[i, 1]] = modCoords[[i, axis1]];
  modpts[[i, 2]] = modCoords[[i, axis2]], {i, numGroups * modelsPerAxis}]]
```

```
If[modSet == 3,
  Do[modpts[[i, 1]] = modCoords[[i, axis1]];
   modpts[[i, 2]] = modCoords[[i, axis2]], {i, nrows}]];

groupNames = Union[Group];
numGroups = Length[groupNames];
groupPosns =
  Table[Flatten[Position[Group, groupNames[[i]], 1]], {i, numGroups}];
eScoresT = Transpose[eScores];

x2T = Transpose[x2];
xAxis = x2T[[axis1]]; yAxis = x2T[[axis2]];
lab1 = varNames[[axis1]];
lab2 = varNames[[axis2]];
maxx = Max[xAxis];
minx = Min[xAxis];
maxy = Max[yAxis];
miny = Min[yAxis];

If[pltAspect == 1 || pltAspect == 3,
  xPlotLow = minx; xPlotHi = maxx; yPlotLow = miny; yPlotHi = maxy];
If[pltAspect == 2,
  If[minx > miny,
   xPlotLow = miny; yPlotLow = miny,
   xPlotLow = minx; yPlotLow = minx ]];
If[pltAspect == 2,
  If[maxx < maxy,
   xPlotHi = maxy; yPlotHi = maxy,
   xPlotHi = maxx; yPlotHi = maxx]];
If[pltAspect == 1, aRatio = 1 / N[GoldenRatio]];
If[pltAspect == 2, aRatio = 1];
If[pltAspect == 3, aRatio = Automatic];

tmpPoints = Transpose[List[xAxis, yAxis]];
pltPoints = Table[tmpPoints[[groupPosns[[j]]]], {j, numGroups}];
iconList = Flatten[Table[
    {Graphics[{EdgeForm[{Thickness[0.005], Black}],
       Hue[N[((numGroups + 1) - j) / numGroups]],
       Disk[{0, 0}, Scaled[iconSize]]}]}, {j, numGroups}]];

If[lch == 1 || ptsJoin == 1,
 Do[
  pltTable[[k, 1]] = pTmp =
    ListPlot[pltPoints[[k]], AspectRatio → aRatio, Frame → True, Joined → False,
     Axes → False, PlotRange → {{xPlotLow, xPlotHi}, {yPlotLow, yPlotHi}},
     PlotRangePadding → Scaled[pltPad], Ticks → Automatic, FrameLabel →
      {lab1, lab2}, PlotMarkers → iconList[[k]], ImageSize → pltSize, LabelStyle →
```

```
      Directive[FontSize → 14, Black, FontFamily → "Arial"]], {k, kg}],
 Do[
  pltTable[[k, 1]] = ListPlot[pltPoints[[k]], Frame → True, Axes → False,
    AspectRatio → aRatio, PlotRange → {{xPlotLow, xPlotHi}, {yPlotLow, yPlotHi}},
    PlotRangePadding → Scaled[pltPad], Ticks → Automatic,
    FrameLabel → {lab1, lab2}, LabelStyle →
     Directive[Black, FontSize → 14, FontFamily → "Arial"], ImageSize → pltSize,
    PlotStyle → Directive[Disk[], Hue[N[((kg + 1) - k) / kg]], EdgeForm[
       {Thickness[1.0], Black}], PointSize[Scaled[iconSize - 0.009]]]], {k, kg}]]

If[ptsJoin == 2,
  Do[
   pltTable[[k, 2]] = ListLinePlot[pltPoints[[k]],
     AspectRatio → aRatio, Frame → True, Joined → True, Axes → False,
     PlotStyle → Directive[Hue[N[((kg + 1) - k) / kg]], Thin],
     PlotRange → {{xPlotLow, xPlotHi}, {yPlotLow, yPlotHi}},
     PlotRangePadding → Scaled[pltPad], Ticks → Automatic,
     FrameLabel → {lab1, lab2}, ImageSize → pltSize, LabelStyle →
      Directive[FontSize → 14, Black, FontFamily → "Arial"]], {k, kg}]];

If[lch == 2,
  Do[
   hull = ConvexHullMesh[pltPoints[[k]]];
   pltTable[[k, 3]] =
    HighlightMesh[hull, Style[2, Opacity[0.2], Hue[N[((kg + 1) - k) / kg]]],
     Frame → True, Axes → False, AspectRatio → aRatio,
     PlotRange → {{xPlotLow, xPlotHi}, {yPlotLow, yPlotHi}}, PlotRangePadding →
      Scaled[pltPad], Ticks → Automatic, FrameLabel → {lab1, lab2},
     LabelStyle → Directive[Black, FontSize → 14, FontFamily → "Arial"],
     ImageSize → pltSize], {k, kg}]];

If[ptsJoin == 1 && lch == 1, p0 = Show[pltTable[[All, 1]]]];
If[ptsJoin == 2 && lch == 1, p0 = Show[pltTable[[All, 2]], pltTable[[All, 1]]]];
If[ptsJoin == 1 && lch == 2, p0 = Show[pltTable[[All, 3]], pltTable[[All, 1]]]];
If[ptsJoin == 2 && lch == 2,
  p0 = Show[pltTable[[All, 3]], pltTable[[All, 2]], pltTable[[All, 1]]]];

iconList = Flatten[Table[
    {Graphics[{EdgeForm[{Thin, Black}],
       Black, Disk[{0, 0}, Scaled[iconSize / 2]]}]}, {j, 1}]];

If[modSet == 1,
  Do[
   axisPlots[[i]] = ListPlot[modpts[[i]], AspectRatio → 1 / GoldenRatio,
     Frame → True, Joined → True, Axes → False, PlotRange → Full,
     PlotStyle → Directive[Black, AbsoluteThickness[0.8]],
     PlotRangePadding → Scaled[pltPad], Ticks → Automatic,
```

```
      FrameLabel → {lab1, lab2}, PlotMarkers → iconList], {i, modelAxes}];
   p4 = Show[axisPlots]];

If[modSet == 2,
   p4 = ListPlot[modpts, AspectRatio → 1 / GoldenRatio,
     Frame → True, Joined → True, Axes → False, PlotRange → Full,
     PlotStyle → Directive[Black, AbsoluteThickness[0.8]],
     PlotRangePadding → Scaled[pltPad], Ticks → Automatic,
     FrameLabel → {lab1, lab2}, PlotMarkers → iconList]];

If[modSet == 3,
   p4 = ListPlot[modpts, AspectRatio → 1 / GoldenRatio,
     Frame → True, Joined → False, Axes → False, PlotRange → Full,
     PlotStyle → Directive[Black, AbsoluteThickness[0.8]],
     PlotRangePadding → Scaled[pltPad], Ticks → Automatic,
     FrameLabel → {lab1, lab2}, PlotMarkers → iconList]];

p1 = Labeled[Show[p0, p4], "           Original Data Plot", Top,
    LabelStyle → Directive[FontSize → 18, Bold, FontFamily → "Arial"]];
g1 = Grid[Table[
     {Graphics[{EdgeForm[{Thin, Black}], Hue[N[((numGroups + 1) - j) / numGroups]],
        Disk[]}]}, {j, numGroups}], Frame → False, ItemSize → 0.9];
g2 = Grid[Partition[groupNames, 1], Alignment → Left,
    BaseStyle → {FontFamily → "Arial", FontSize → 13, Italic}];
p2 = Labeled[Text[Grid[{{g1, g2}}, Alignment → Bottom, Frame → True]], "Legend",
    Top, LabelStyle → Directive[Black, FontSize → 18, Bold, FontFamily → "Arial"]];

plt2D = Grid[{{p1, p2}}, BaselinePosition → Top, Alignment → Top]
```

Export plot.

```
In[•]:= filenameout = SystemDialogInput["FileSave"];
Export[filenameout, plt2D, "TIFF", ImageResolution → 150]
```

Export CVA model coordinates in space of original variables.

```
In[•]:= filenameout = SystemDialogInput["FileSave"];
Export[filenameout, modCoords, "CSV", "TextDelimiters" → ""]
```

Jackknife discrimination performance section

Calculate jackknifed estimate of discriminant function performance.

```
In[•]:= eScoresJ = Table[0.0, {n2}, {m2}];
eScrsJ = Table[0.0, {m2}];
gps = Length[Union[Group]];
noVals = Min[{gps - 1, m2}];
gMeans = Table[0.0, {gps}, {noVals}];
mDist = Table[0.0, {n2}, {gps}];
distTable = Table[0.0, {n2 + 1}, {gps + 2}];
distTableOut = Table[0.0, {n2 + 1}, {gps + 2}];
n3 = n2 - 1;
m3 = m2;
smpSize2 = smpSize;

Panel[Labeled[ProgressIndicator[Dynamic[k], {1, n2}], "Calculation Progress",
  Top, LabelStyle → Directive[FontSize → 12, Bold, FontFamily → "Arial"]]]

Do[

 x3 = Drop[x2, {k}];
 xt = Take[x2, {k}];
 g3 = Drop[Group, {k}];
 gt = Take[Group, {k}];

 {gndMean, T, gNames, nGps, gpMeans, smpSize, W, B} = TWB[x3, n3, m3, Group];

 WI = PseudoInverse[W];
 sCovar = WI.B;
 eVals = Eigenvalues[sCovar];

 noVals = Min[{nGps - 1, m2}];
 eVals = Take[eVals, noVals];

 eVecs = Transpose[Eigenvectors[sCovar]];
 eScores = x3.eVecs;

 eScrs = Take[eScores, All, noVals];

 eScrsJ = xt.eVecs;
 eScoresJ[[k]] = eScrsJ;
 eScrsJ = Flatten[eScrsJ];
 eScrsJ = Take[eScrsJ, noVals];

 Do[
  Do[
    sum = 0.0;
    Do[
     If[Group[[i]] == gNames[[k3]],
      sum = sum + eScores[[i, j]],
```

```
      sum = sum],
     {i, n3}];
    gMeans[[k3, j]] = sum / smpSize[[k3]],
    {j, noVals}];,
  {k3, nGps}];
 gMeans;

 Do[
  mDist[[k, k3]] = EuclideanDistance[gMeans[[k3]], eScrsJ],
  {k3, nGps}];
 mDist, {k, 1, n2}]

eScoresJ = Partition[Flatten[eScoresJ], m2];
mDist;

pDist = mDist;
Do[
 n = 0;
 ref = Min[pDist[[k]]];
 Do[
  If[pDist[[k, j]] == ref, pDist[[k, j]] = Style[pDist[[k, j]], Bold]],
  {j, nGps}], {k, n2}]

distTable[[1, 1]] = "Object";
distTableOut[[1, 1]] = "Object";
distTable[[1, 2]] = "Group";
distTableOut[[1, 2]] = "Group";
Do[distTable[[1, j + 2]] = Text[Rotate[gNames[[j]], 90 Degree]], {j, nGps}]
Do[distTableOut[[1, j + 2]] = gNames[[j]], {j, nGps}]
Do[
 distTable[[i + 1, 1]] = objNames[[i]];
 distTableOut[[i + 1, 1]] = objNames[[i]];
 distTable[[i + 1, 2]] = Group[[i]];
 distTableOut[[i + 1, 2]] = Group[[i]];
 Do[distTable[[i + 1, j + 2]] = PaddedForm[pDist[[i, j]], {4, 3}], {j, nGps}];
 Do[distTableOut[[i + 1, j + 2]] = pDist[[i, j]], {j, nGps}],
 {i, n2}]

distances = Labeled[Grid[distTable, BaseStyle → (FontFamily → "Arial"),
   Alignment → {{Left, Center, Center, Center, Center, Center, Center, Center},
     {Bottom, Baseline}}, Frame → True,
   Dividers → {{True, True, True}, {True, True}}], "Jackknifed Distance Table",
  Top, LabelStyle → Directive[FontSize → 14, Bold, FontFamily → "Arial"]]
Print[" "]

cMat = Table[0, {nGps}, {nGps}];
gPos = Table[Flatten[Position[Group, gNames[[i]], 1]], {i, nGps}];
```

```
pDist = mDist;

Do[
 Do[
  Do[
   gPos[[k, ig]];
   pDist[[gPos[[k, ig]]]];
   ref = Min[pDist[[gPos[[k, ig]]]]];
   pDist[[gPos[[k, ig]], j]];
   If[pDist[[gPos[[k, ig]], j]] == ref,
    cMat[[k, j]] = cMat[[k, j]] + 1, cMat[[k, j]] = cMat[[k, j]]],
   {j, nGps}],
  {ig, smpSize2[[k]]}],
 {k, nGps}]
cMat;

cmTable = Table[0, {nGps + 4}, {nGps + 4}];
cmOut = Table[0, {nGps + 4}, {nGps + 4}];
cmTable[[1, 1]] = "Groups";
cmOut[[1, 1]] = "Groups";
Do[cmTable[[1, j + 1]] = Text[Rotate[gNames[[j]], 90 Degree]], {j, nGps}];
Do[cmOut[[1, j + 1]] = gNames[[j]], {j, nGps}];
Do[cmTable[[j + 1, 1]] = gNames[[j]], {j, nGps}];
Do[cmOut[[j + 1, 1]] = gNames[[j]], {j, nGps}];
cmTable[[nGps + 2, 1]] = "Total Correct";
cmOut[[nGps + 2, 1]] = "Total Correct";
cmTable[[nGps + 3, 1]] = "Total Estimated";
cmOut[[nGps + 3, 1]] = "Total Estimated";
cmTable[[nGps + 4, 1]] = "Percent Estimated Correctly";
cmOut[[nGps + 4, 1]] = "Percent Estimated Correctly";
cmTable[[1, nGps + 3]] = Text[Rotate["Group Totals", 90 Degree]];
cmOut[[1, nGps + 3]] = "Group Totals";
cmTable[[1, nGps + 2]] = Text[Rotate["Total Correct", 90 Degree]];
cmOut[[1, nGps + 2]] = "Total Correct";
cmTable[[1, nGps + 4]] = Text[Rotate["Percent Correct", 90 Degree]];
cmOut[[1, nGps + 4]] = "Percent Correct";
Do[cmTable[[i + 1, j + 1]] = cMat[[i, j]], {i, nGps}, {j, nGps}];
Do[cmOut[[i + 1, j + 1]] = cMat[[i, j]], {i, nGps}, {j, nGps}];
cTot = Total[cMat];
rTot = Diagonal[cMat];
gpTot = Total[cMat, {2}];
gTot = Total[cTot];

Do[cmTable[[i + 1, nGps + 2]] = rTot[[i]], {i, nGps}];
Do[cmOut[[i + 1, nGps + 2]] = rTot[[i]], {i, nGps}];
Do[cmTable[[i + 1, nGps + 3]] = gpTot[[i]], {i, nGps}];
Do[cmOut[[i + 1, nGps + 3]] = gpTot[[i]], {i, nGps}];
```

```
Do[cmTable[[i + 1, nGps + 4]] =
   PaddedForm[N[(rTot[[i]] / gpTot[[i]]) * 100.0], {4, 2}], {i, nGps}];
Do[cmOut[[i + 1, nGps + 4]] = N[(rTot[[i]] / gpTot[[i]]) * 100.0], {i, nGps}];
cmTable[[nGps + 2, nGps + 3]] = gTot;
cmOut[[nGps + 2, nGps + 3]] = gTot;
cmTable[[nGps + 2, nGps + 2]] = Total[rTot];
cmOut[[nGps + 2, nGps + 2]] = Total[rTot];
cmTable[[nGps + 2, nGps + 4]] =
  PaddedForm[N[(Total[rTot] / gTot) * 100.0], {4, 2}];
cmOut[[nGps + 2, nGps + 4]] = N[(Total[rTot] / gTot) * 100.0];

Do[cmTable[[nGps + 2, i + 1]] = rTot[[i]], {i, nGps}]
Do[cmOut[[nGps + 2, i + 1]] = rTot[[i]], {i, nGps}]
Do[cmTable[[nGps + 3, i + 1]] = cTot[[i]], {i, nGps}]
Do[cmOut[[nGps + 3, i + 1]] = cTot[[i]], {i, nGps}]
Do[cmTable[[nGps + 4, i + 1]] =
   PaddedForm[N[(rTot[[i]] / cTot[[i]]) * 100.0], {4, 2}], {i, nGps}];
Do[cmOut[[nGps + 4, i + 1]] = N[(rTot[[i]] / cTot[[i]]) * 100.0], {i, nGps}];
cmTable[[nGps + 3, nGps + 2]] = Total[cTot];
cmOut[[nGps + 3, nGps + 2]] = Total[cTot];
cmTable[[nGps + 4, nGps + 2]] =
  PaddedForm[N[(Total[rTot] / Total[cTot]) * 100.0], {4, 2}];
cmOut[[nGps + 4, nGps + 2]] = N[(Total[rTot] / Total[cTot]) * 100.0];

cmTable[[nGps + 3, nGps + 3]] = " ";
cmTable[[nGps + 3, nGps + 4]] = " ";
cmTable[[nGps + 4, nGps + 3]] = " ";
cmTable[[nGps + 4, nGps + 4]] = " ";
cmOut[[nGps + 3, nGps + 3]] = " ";
cmOut[[nGps + 3, nGps + 4]] = " ";
cmOut[[nGps + 4, nGps + 3]] = " ";
cmOut[[nGps + 4, nGps + 4]] = " ";
Print[" "];

confusion = Labeled[Grid[cmTable, Frame → True,
   Dividers → {{2 → True, -4 → True}, {2 → True, -4 → True}},
   BaseStyle → (FontFamily → "Arial"), Alignment →
    {{Right, Center}, {Bottom, Baseline}}], "Jackknifed Confusion Matrix",
  Top, LabelStyle → Directive[FontSize → 14, Bold, FontFamily → "Arial"]]
```

Out[∘]=

Calculation Progress

Export jackknifed distance table.

```
In[•]:= filenameout = SystemDialogInput["FileSave"];
Export[filenameout, distTableOut, "CSV", "TextDelimiters" → ""]
```

Export jackknifed confusion matrix table.

```
In[•]:= filenameout = SystemDialogInput["FileSave"];
Export[filenameout, cmOut, "CSV", "TextDelimiters" → ""]
```

Data Projection Module

This routine allows you to import data for objects that were not used to contruct the CVA space and project them into the CVA space with full support for data visualization graphics.

Read in projection datafile & partition into datasets.

In[•]:=
```
filenamein = SystemDialogInput["FileOpen"];
x3 = Import[filenamein, "CSV"];
filenamein

{nProj, mProj} = Dimensions[x3];
varNames = Flatten[Take[x3, 1]];
x4 = Drop[x3, 1];
varNames = Drop[varNames, 1];
varNames = Drop[varNames, 1];

projObjectNames = Flatten[Take[x4, nProj - 1, 1]];
x4 = Drop[x4, 0, 1];

projGroup = Flatten[Take[x4, nProj - 1, 1]];
x4 = Drop[x4, 0, 1];
numProjGroups = Length[Union[projGroup]];

{n4, m4} = Dimensions[x4];
If[meanTrans == 2, Do[x4[[i]] = x4[[i]] - mVec, {i, n4}]];
If[logTrans == 2, x2 = N[Log10[x4]]];
If[stdTrans == 2, x2 = Standardize[x4]];
If[shiftTrans == 2, x4 = x4 + knsnt];
projEScores = x4.eVecs;

Print["No. of groups: ", Length[Union[Group]]];
Print["No. of objects: ", n4];
Print["No. of variables: ", m4];
```

Projection Plot section

Plot single – axis histogram.

Specify single – variable plot (histogram) options.

In[∘]:=
```
Panel[Labeled[Column[{
    Row[{Panel[Labeled[InputField[Dynamic[cvAxis], FieldSize → 5],
        "Enter no. of CVA axis to be plotted.", Top,
        LabelStyle → Directive[FontSize → 12, Bold, FontFamily → "Arial"]]],
      "  ", Panel[Labeled[PopupMenu[Dynamic[histType],
         {1 → "Stacked", 2 → "Overlapped"}], "Select histogram type.", Top,
        LabelStyle → Directive[FontSize → 12, Bold, FontFamily → "Arial"]]]}],
    Row[{Panel[Labeled[InputField[Dynamic[noBins], FieldSize → 5],
        "Enter no. of histogram bins.", Top, LabelStyle →
         Directive[FontSize → 12, Bold, FontFamily → "Arial"]]], "  ",
      Panel[Labeled[InputField[Dynamic[hSize], FieldSize → 5],
        "Enter histogram plot size.", Top,
        LabelStyle → Directive[FontSize → 12, Bold, FontFamily → "Arial"]]]}]},
   Center], "Single Axis (Histogram) Plot Options", Top,
  LabelStyle → Directive[FontSize → 16, Bold, FontFamily → "Arial"]]]
cvAxis = 1; noBins = 15; hSize = 500; histType = 1;
```

Out[∘]=

Single Axis (Histogram) Plot Options

**Enter no. of CVA axis to be plotted.**	**Select histogram type.**
cvAxis	Stacked ⋁
**Enter no. of histogram bins.**	**Enter histogram plot size.**
15	500

Constrruct and display histogram plot .

```
projPltScores = Flatten[Take[projEScores, All, {cvAxis}]];
If[histType == 1, htype = "Stacked", htype = "Overlapped"];
projGpNames = Union[projGroup];
projGroupPosns =
  Table[Flatten[Position[projGroup, projGpNames[[i]], 1]], {i, numProjGroups}];
projPltPoints = Table[projPltScores[[projGroupPosns[[j]]]], {j, numProjGroups}];
hueList =
  Table[Hue[N[((numProjGroups + 1) - j) / numProjGroups]], {j, numProjGroups}];
projh1 = Labeled[Histogram[projPltPoints, nobins,
   ChartStyle → {hueList}, ChartLayout → htype,
   LabelStyle → Directive[FontSize → 12, FontFamily → "Arial"],
   AxesLabel → {"Discrim. Score", "Frequency"},
   ImageSize → hSize, ChartLegends → projGpNames],
  StringJoin["Canonical Variate ", ToString[cvAxis]], Top,
  LabelStyle → Directive[FontSize → 16, Bold, FontFamily → "Arial"]]
```

Export data projection histogram.

```
filenameout = SystemDialogInput["FileSave"];
Export[filenameout, projh1, "TIFF"]
```

Create 2 D scatterplot (use only for datasets containing three groups or more).

Specify 2 D plot options.
You must run this code after you read in the data so it can pick up the proper variable names.

In[•]:=
```
cvNames = Table[StringJoin["CV-", ToString[i]], {i, noAxes}];
Panel[
 Labeled[Column[{Row[{Panel[Labeled[PopupMenu[Dynamic[xAxisName], cvNames],
        "Select variable to be plotted on x-Axis.", Top, LabelStyle →
         Directive[FontSize → 12, Bold, FontFamily → "Arial"]]], "  ",
      Panel[Labeled[PopupMenu[Dynamic[yAxisName], cvNames],
        "Select variable to be plotted on y-Axis.", Top,
        LabelStyle → Directive[FontSize → 12, Bold, FontFamily → "Arial"]]]}],
    Row[{
      Panel[Labeled[PopupMenu[Dynamic[pltAspect],
          {1 → "Golden Ratio Plot", 2 → "Square Plot (equi-length axes)",
           3 → "True-Scale Plot (actual axis scales)"}],
        "Enter plot aspect ratio type.", Top, LabelStyle →
         Directive[FontSize → 12, Bold, FontFamily → "Arial"]]], "  ",
      Panel[Labeled[PopupMenu[Dynamic[lch], {1 → "Simple scatterplot",
           2 → "Scatterplot w/ convex hulls"}],
        "Show group domians?", Top, LabelStyle →
         Directive[FontSize → 12, Bold, FontFamily → "Arial"]]], "  ",
      Panel[Labeled[PopupMenu[Dynamic[ptsJoin], {1 → "No", 2 → "Yes"}],
        "Join datapoints?", Top,
        LabelStyle → Directive[FontSize → 12, Bold, FontFamily → "Arial"]]]}],
    Row[{Panel[Labeled[InputField[Dynamic[pltSize], FieldSize → 10],
        "Enter plot size value.", Top,
        LabelStyle → Directive[FontSize → 12, Bold, FontFamily → "Arial"]]],
      "  ", Panel[Labeled[InputField[Dynamic[pltPad], FieldSize → 10],
        "Enter plot margin padding value.", Top, LabelStyle →
         Directive[FontSize → 12, Bold, FontFamily → "Arial"]]], "  ",
      Panel[Labeled[InputField[Dynamic[iconSize], FieldSize → 10],
        "Enter plot icon size value.", Top, LabelStyle →
         Directive[FontSize → 12, Bold, FontFamily → "Arial"]]]}]}, Center],
  "2D Plot Options", Top, LabelStyle → Directive[FontSize → 18,
    Bold, FontFamily → "Arial"]]]
pltSize = 500; iconSize = 0.04; pltPad = 0.1; xAxisName = cvNames[[1]];
yAxisName = cvNames[[2]];
 ptsJoin = 1; dataTrans = 1;  pltAspect = 1 ; lch = 1;
```

Table: Iterator {i, noAxes} does not have appropriate bounds.

Out[∘]=

2D Plot Options

Select variable to be plotted on x–Axis.

PopupMenu[PC–1, Table[CV– <> ToString[i], {i, noAxes}]]

Select variable to be plotted on y–Axis.

PopupMenu[PC–2, Table[CV– <> ToString[i], {i, noAxes}]]

Enter plot aspect ratio type.

True–Scale Plot (actual axis scales)

Show group domians?

Scatterplot w/ convex hulls

Join datapoints?

No

Enter plot size value.

500

Enter plot margin padding value.

0.1

Enter plot icon size value.

30.

Plot script

In[∘]:=

```
projPltTable = Table[" ", {numProjGroups}, {3}];
Do[If[xAxisName == cvNames[[j]], axis1 = j], {j, noAxes}];
Do[If[yAxisName == cvNames[[j]], axis2 = j], {j, noAxes}];

projGroupNames = Union[projGroup];
numGroups = Length[groupNames];
projGroupPosns = Table[
   Flatten[Position[projGroup, projGroupNames[[i]], 1]], {i, numProjGroups}];
projEScoresT = Transpose[projEScores];

xAxis = projEScoresT[[axis1]]; yAxis = projEScoresT[[axis2]];
lab1 = StringJoin[{"Canonical Variate ", ToString[axis1]},
   {" (Var. =", ToString[t3[[axis1 + 1, 3]]]}, {"%)"}];
lab2 = StringJoin[{"Canonical Variate ", ToString[axis2]},
   {" (Var. =", ToString[t3[[axis2 + 1, 3]]]}, {"%)"}];
maxx =
 Max[
  xAxis];
minx = Min[xAxis];
maxy = Max[yAxis];
miny = Min[yAxis];

If[pltAspect == 1 || pltAspect == 3,
  xPlotLow = minx; xPlotHi = maxx; yPlotLow = miny; yPlotHi = maxy];
```

```
If[pltAspect == 2,
  If[minx > miny,
   xPlotLow = miny; yPlotLow = miny,
   xPlotLow = minx; yPlotLow = minx ]];
If[pltAspect == 2,
  If[maxx < maxy,
   xPlotHi = maxy; yPlotHi = maxy,
   xPlotHi = maxx; yPlotHi = maxx]];
If[pltAspect == 1, aRatio = 1 / N[GoldenRatio]];
If[pltAspect == 2, aRatio = 1];
If[pltAspect == 3, aRatio = Automatic];

projTmpPoints = Transpose[List[xAxis, yAxis]];
projPltPoints =
  Table[projTmpPoints[[projGroupPosns[[j]]]], {j, numProjGroups}];
iconList = Flatten[Table[
    {Graphics[
      {EdgeForm[{Thin, Black}], Hue[N[((numProjGroups + 1) - j) / numProjGroups]],
       Disk[{0, 0}, Scaled[iconSize]]}]}, {j, numProjGroups}]];

If[lch == 1 || ptsJoin == 1,
 Do[
  projPltTable[[k, 1]] =
   pTmp = ListPlot[projPltPoints[[k]], AspectRatio → aRatio, Frame → True, Joined →
      False, Axes → False, PlotRange → {{xPlotLow, xPlotHi}, {yPlotLow, yPlotHi}},
     PlotRangePadding → Scaled[pltPad], Ticks → Automatic,
     FrameLabel → {lab1, lab2}, PlotMarkers → iconList[[k]],
     ImageSize → pltSize, LabelStyle → Directive[FontSize → 14,
       Black, FontFamily → "Arial"]], {k, numProjGroups}],
 Do[
  projPltTable[[k, 1]] = ListPlot[projPltPoints[[k]], Frame → True, Axes → False,
    AspectRatio → aRatio, PlotRange → {{xPlotLow, xPlotHi}, {yPlotLow, yPlotHi}},
    PlotRangePadding → Scaled[pltPad], Ticks → Automatic,
    FrameLabel → {lab1, lab2}, LabelStyle →
     Directive[Black, FontSize → 14, FontFamily → "Arial"], ImageSize → pltSize,
    PlotStyle → Directive[Disk[], Hue[N[((numProjGroups + 1) - k) / numProjGroups]],
      EdgeForm[{Thickness[1.0], Black}],
      PointSize[Scaled[iconSize - 0.009]]]], {k, numProjGroups}]]

If[ptsJoin == 2,
  Do[
   projPltTable[[k, 2]] = ListLinePlot[projPltPoints[[k]],
     AspectRatio → aRatio, Frame → True, Joined → True, Axes → False,
     PlotStyle → Directive[Hue[N[((numGroups + 1) - k) / numGroups]], Thin],
     PlotRange → {{xPlotLow, xPlotHi}, {yPlotLow, yPlotHi}},
     PlotRangePadding → Scaled[pltPad], Ticks → Automatic,
     FrameLabel → {lab1, lab2}, ImageSize → pltSize, LabelStyle → Directive[
```

```
      FontSize → 14, Black, FontFamily → "Arial"]], {k, numProjGroups}]];

If[lch == 2,
  Do[
   hull = ConvexHullMesh[projPltPoints[[k]]];
   projPltTable[[k, 3]] = HighlightMesh[hull,
     Style[2, Opacity[0.2], Hue[N[((numProjGroups + 1) - k) / numProjGroups]]],
     Frame → True, Axes → False, AspectRatio → aRatio,
     PlotRange → {{xPlotLow, xPlotHi}, {yPlotLow, yPlotHi}}, PlotRangePadding →
      Scaled[pltPad], Ticks → Automatic, FrameLabel → {lab1, lab2},
     LabelStyle → Directive[Black, FontSize → 14, FontFamily → "Arial"],
     ImageSize → pltSize], {k, numProjGroups}]];

If[ptsJoin == 1 && lch == 1, p0 = Show[projPltTable[[All, 1]]]];
If[ptsJoin == 2 && lch == 1,
  p0 = Show[projPltTable[[All, 2]], projPltTable[[All, 1]]]];
If[ptsJoin == 1 && lch == 2,
  p0 = Show[projPltTable[[All, 3]], projPltTable[[All, 1]]]];
If[ptsJoin == 2 && lch == 2, p0 = Show[projPltTable[[All, 3]],
    projPltTable[[All, 2]], projPltTable[[All, 1]]]];

p1 = Labeled[p0, "           CV Score Plot", Top,
   LabelStyle → Directive[FontSize → 18, Bold, FontFamily → "Arial"]];
g1 = Grid[Table[
    {Graphics[{EdgeForm[{Thin, Black}],
       Hue[N[((numProjGroups + 1) - j) / numProjGroups]], Disk[]}]},
    {j, numProjGroups}], Frame → False, ItemSize → 0.9];
g2 = Grid[Partition[projGroupNames, 1], Alignment → Left,
   BaseStyle → {FontFamily → "Arial", FontSize → 13, Italic}];
p2 = Labeled[Text[Grid[{{g1, g2}}, Alignment → Bottom, Frame → True]], "Legend",
   Top, LabelStyle → Directive[Black, FontSize → 18, Bold, FontFamily → "Arial"]];

Plt2D = Grid[{{p1, p2}}, BaselinePosition → Top, Alignment → Top]
```

Export current 2 D plot.

```
filenameout = SystemDialogInput["FileSave"];
Export[filenameout, Plt2D, "TIFF", ImageResolution → 150]
```

Label plotted points.

```
pn1 = p0;
projNamePoints = projTmpPoints;
projTempPointsT = Transpose[projTmpPoints];
mxY = Max[projTempPointsT[[2]]];
mnY = Min[projTempPointsT[[2]]];
incY = N[(mxY - mnY) / 15];
Do[projNamePoints[[i, 2]] = projTmpPoints[[i, 2]] - incY, {i, n4}]
nPointsTable =
  Table[{Text[objNames[[i]], projNamePoints[[i]], {-1, 0}]}, {i, n4}];
pn2 = Graphics[nPointsTable, Frame → True, AspectRatio → aRatio , Axes → False,
   FrameLabel  → {lab1, lab2}, PlotRangePadding → Scaled[pltPad], BaseStyle →
    Directive[FontSize → 12, FontFamily → "Arial"], ImageSize → pltSize];

p1 = Labeled[Show[pn1, pn2, BaseStyle → {FontFamily → "Arial"}],
   "           CV Score Plot", Top,
   LabelStyle → Directive[FontSize → 18, Bold, FontFamily → "Arial"]];

Plt2D = Grid[{{p1, p2}}, BaselinePosition → Top, Alignment → Top]
```

Export current 2 D plot.

```
filenameout = SystemDialogInput["FileSave"];
Export[filenameout, Plt2D, "TIFF", ImageResolution → 150]
```

Create 3 D scatterplot (use only for datasets containing four groups or more).

Specify 3 D plot options.
You must run this code after you read in the data so it can pick up the proper variable names.

```
In[•]:= cvNames = Table[StringJoin["CV-", ToString[i]], {i, noAxes}];
Panel[
 Labeled[Column[{Row[{Panel[Labeled[PopupMenu[Dynamic[xAxisName], cvNames],
        "Select variable to be plotted on x-Axis.", Top, LabelStyle →
         Directive[FontSize → 12, Bold, FontFamily → "Arial"]]], "  ",
      Panel[Labeled[PopupMenu[Dynamic[yAxisName], cvNames],
        "Select variable to be plotted on y-Axis.", Top, LabelStyle →
         Directive[FontSize → 12, Bold, FontFamily → "Arial"]]], "  ",
      Panel[Labeled[PopupMenu[Dynamic[zAxisName], cvNames],
        "Select variable to be plotted on z-Axis.", Top,
        LabelStyle → Directive[FontSize → 12, Bold, FontFamily → "Arial"]]]}],
    Row[{
      Panel[Labeled[PopupMenu[Dynamic[pltAspect],
          {1 → "Golden Ratio Plot", 2 → "Square Plot (equi-length axes)",
           3 → "True-Scale Plot (actual axis scales)"}],
        "Enter plot aspect ratio type.", Top, LabelStyle →
         Directive[FontSize → 12, Bold, FontFamily → "Arial"]]], "  ",
      Panel[Labeled[PopupMenu[Dynamic[lch], {1 → "Simple scatterplot",
           2 → "Scatterplot w/ convex hulls"}],
        "Show group domians?", Top, LabelStyle →
         Directive[FontSize → 12, Bold, FontFamily → "Arial"]]], " ",
      Panel[Labeled[PopupMenu[Dynamic[ptsJoin], {1 → "No", 2 → "Yes"}],
        "Join datapoints?", Top,
        LabelStyle → Directive[FontSize → 12, Bold, FontFamily → "Arial"]]]}],
    Row[{Panel[Labeled[InputField[Dynamic[pltSize], FieldSize → 5],
        "Enter plot size value.", Top,
        LabelStyle → Directive[FontSize → 12, Bold, FontFamily → "Arial"]]],
      " ", Panel[Labeled[InputField[Dynamic[pltPad], FieldSize → 5],
        "Enter plot margin padding value.", Top, LabelStyle →
         Directive[FontSize → 12, Bold, FontFamily → "Arial"]]], " ",
      Panel[Labeled[InputField[Dynamic[iconSize3D], FieldSize → 5],
        "Enter plot icon size value.", Top, LabelStyle →
         Directive[FontSize → 12, Bold, FontFamily → "Arial"]]]}]}, Center],
  "3D Plot Options", Top, LabelStyle → Directive[FontSize → 18,
    Bold, FontFamily → "Arial"]]]
pltSize = 500; iconSize3D = 60; pltPad = 0.1; xAxisName = cvNames[[1]];
 yAxisName = cvNames[[2]];
zAxisName = cvNames[[3]]; ptsJoin = 1;
dataTrans = 1; pltAspect = 3 ;
lch = 1;
```

Table: Iterator {i, noAxes} does not have appropriate bounds.

Out[ ]=

3D Plot Options

Select variable to be plotted on x–Axis.
PopupMenu[PC–1, Table[CV– <> ToString[i], {i, noAxes}]]

Select variable to be plotted on y–Axis.
PopupMenu[PC–2, Table[CV– <> ToString[i], {i, noAxes}]]

Select variable to be plotted on z–Axis.
PopupMenu[PC–3, Table[CV– <> ToString[i], {i, noAxes}]]

Enter plot aspect ratio type.
True–Scale Plot (actual axis scales)

Show group domians?
Scatterplot w/ convex hulls

Join datapoints?
No

Enter plot size value.
500

Enter plot margin padding value.
0.1

Enter plot icon size value.
10

Part: Part 3 of Table[CV– <> ToString[i], {i, noAxes}] does not exist.

Plot script

```
Do[If[xAxisName == cvNames[[j]], axis1 = j], {j, noAxes}]
Do[If[yAxisName == cvNames[[j]], axis2 = j], {j, noAxes}]
Do[If[zAxisName == cvNames[[j]], axis3 = j], {j, noAxes}]
iconSize = iconSize3D - 50.0;

projGroupNames = Union[projGroup];
numProjGroups = Length[projGroupNames];
projGroupPosns = Table[
   Flatten[Position[projGroup, projGroupNames[[i]], 1]], {i, numProjGroups}];
projEScoresT = Transpose[projEScores];

xAxis = projEScoresT[[axis1]];
yAxis = projEScoresT[[axis2]];
zAxis = projEScoresT[[axis3]];
lab1 = StringJoin["CV-", ToString[axis1]];
lab2 = StringJoin["CV-", ToString[axis2]];
lab3 = StringJoin["CV-", ToString[axis3]];
maxx = Max[xAxis];
minx = Min[xAxis];
maxy = Max[yAxis];
```

```
miny = Min[yAxis];
maxz = Max[zAxis]; minz = Min[zAxis];

If[pltAspect == 1 || pltAspect == 3,
  xPlotLow = minx; xPlotHi = maxx; yPlotLow = miny; yPlotHi = maxy];
If[pltAspect == 2,
  If[minx > miny,
   xPlotLow = miny; yPlotLow = miny,
   xPlotLow = minx; yPlotLow = minx ]];
If[pltAspect == 2,
  If[maxx < maxy,
   xPlotHi = maxy; yPlotHi = maxy,
   xPlotHi = maxx; yPlotHi = maxx]];

If[pltAspect == 1, bRatio = {1.61803, 1, 1}];
If[pltAspect == 2, bRatio = {1, 1, 1}];
If[pltAspect == 3, bRatio = Automatic];

projPoints3 = Transpose[List[xAxis, yAxis, zAxis]];
projGp1 = projGroupPosns;
ProjH = Table[0, {n4}];
Do[
 tmp = projGp1[[i]];
 itr = Length[tmp];
 Do[h[[tmp[[j]]]] = Hue[N[((numProjGroups + 1) - i) / numProjGroups]], {j, itr}],
 {i, numProjGroups}]

If[lch == 1 && pltAspect == 3,
  projPltPoints =
   Table[{h[[i]], Sphere[projPoints3[[i]], iconSize3D / 55]}, {i, n4}],
  projPltPoints = Table[{h[[i]], AbsolutePointSize[iconSize],
     Point[projPoints3[[i]]]}, {i, n4}]];
p0 = Graphics3D[projPltPoints, Axes → True, Boxed → True,
   PlotRangePadding → Scaled[pltPad],
   LabelStyle → Directive[FontSize → 12, Black, FontFamily → "Arial"],
   AxesLabel → {lab1, lab2, lab3}, ImageSize → pltSize, BoxRatios → bRatio];

If[ptsJoin == 2,
  projPltLineTable = Table[" ", {numProjGroups}];
  gpPoints = Table[projPoints3[[projGroupPosns[[j]]]], {j, numProjGroups}];
  Do[
   projPltLineTable[[k]] =
    Graphics3D[{Hue[N[((numGroups + 1) - k) / numGroups]], Line[gpPoints[[k]]]},
     Axes → True, Boxed → True, PlotRangePadding → Scaled[pltPad],
     LabelStyle → Directive[FontSize → 12, Black, FontFamily → "Arial"],
     AxesLabel → {lab1, lab2, lab3}, ImageSize → pltSize,
     BoxRatios → bRatio, ViewPoint -> {xax, yax, zax}], {k, numGroups}];
```

```
  pTmp = Graphics3D[pltPoints, Axes → True, Boxed → True,
    PlotRangePadding → Scaled[pltPad],
    LabelStyle → Directive[FontSize → 12, Black, FontFamily → "Arial"],
    AxesLabel → {lab1, lab2, lab3}, ImageSize → pltSize,
    BoxRatios → bRatio, ViewPoint -> {xax, yax, zax}];
  p0 = Show[{pltLineTable, pTmp}]];

If[lch == 2,
  projPltMeshTable = Table[" ", {numGroups}];
  projGpPoints =
   Table[projPoints3[[projGroupPosns[[j]]]], {j, numProjGroups}];
  Do[
   {n5, m5} = Dimensions[projGpPoints[[k]]];
   pltPoints = Table[{Hue[N[((numProjGroups + 1) - k) / numProjGroups]],
      AbsolutePointSize[iconSize3D], Point[projGpPoints[[k, i]]]}, {i, n5}];
   pTmp = Graphics3D[projPltPoints, Axes → True, Boxed → True,
     PlotRangePadding → Scaled[pltPad],
     LabelStyle → Directive[FontSize → 12, Black, FontFamily → "Arial"],
     AxesLabel → {lab1, lab2, lab3}, ImageSize → pltSize,
     BoxRatios → bRatio, ViewPoint -> {xax, yax, zax}];
   cHull3D = ConvexHullMesh[projGpPoints[[k]], BaseStyle → {EdgeForm[]},
     Boxed → True, Axes → True, PlotRangePadding → Scaled[pltPad],
     LabelStyle → Directive[FontSize → 12, Black, FontFamily → "Arial"],
     AxesLabel → {lab1, lab2, lab3}, ImageSize → pltSize,
     BoxRatios → bRatio, ViewPoint -> {xax, yax, zax}];
   projPltMeshTable[[k]] = Show[{HighlightMesh[cHull3D, Style[2, Opacity[0.2],
        Hue[N[((numGroups + 1) - k) / numGroups]]]], p0}], {k, kg}];
  p0 = Show[projPltMeshTable]];

p1 = Labeled[p0, "           CV Score Plot", Top,
   LabelStyle → Directive[FontSize → 18, Bold, FontFamily → "Arial"]];
If[lch == 1 && pltAspect == 3, g1 = Table[
    Graphics[{Inset[Graphics3D[{Hue[N[((numProjGroups + 1) - i) / numProjGroups]],
         Sphere[]}, Boxed → False]]}], {i, numProjGroups}],
  g1 = Table[Graphics[{Inset[Graphics[
        {Hue[N[((numProjGroups + 1) - i) / numProjGroups]],
         Disk[{0, 0}, Scaled[0.3]]}]]}], {i, numProjGroups}]];
g2 = Labeled[Text[Grid[Transpose[Partition[Join[g1, projGroupNames],
        numProjGroups]], ItemSize → {{Scaled[0.03], Automatic}}, Alignment → Left,
     BaseStyle → {FontSize → 14, FontFamily → "Arial", Italic},
     Frame → True]], "Legend", Top,
   LabelStyle → Directive[FontSize → 16, Bold, FontFamily → "Arial"]];

plt3D = Grid[{{p1, g2}}, BaselinePosition → Top, Alignment → Top]
```

Adjust orientation of 3 D plot (if necessary).
You must replot the data to activate the changes. These changes will be able to be exported using the script below.

In[•]:=
```
Panel[
 Labeled[Row[{Labeled[Slider[Dynamic[xax], {-10, 10}, Appearance → "Labeled"],
      "x-Axis Viewpoint", Top,
      LabelStyle → Directive[FontSize → 10, Bold, FontFamily → "Arial"]] ×
     Labeled[Slider[Dynamic[yax], {-10, 10}, Appearance → "Labeled"],
      "y-Axis Viewpoint", Top,
      LabelStyle → Directive[FontSize → 10, Bold, FontFamily → "Arial"]] ×
     Labeled[Slider[Dynamic[zax], {-10, 10}, Appearance → "Labeled"],
      "z-Axis Viewpoint", Top,
      LabelStyle → Directive[FontSize → 10, Bold, FontFamily → "Arial"]]}],
   "3D Plot Orientation Controls", Top, LabelStyle →
    Directive[FontSize → 14, Bold, FontFamily → "Ariel"]]]
xax = 2.5; yax = -2.5; zax = 2.5;
```

Out[•]=

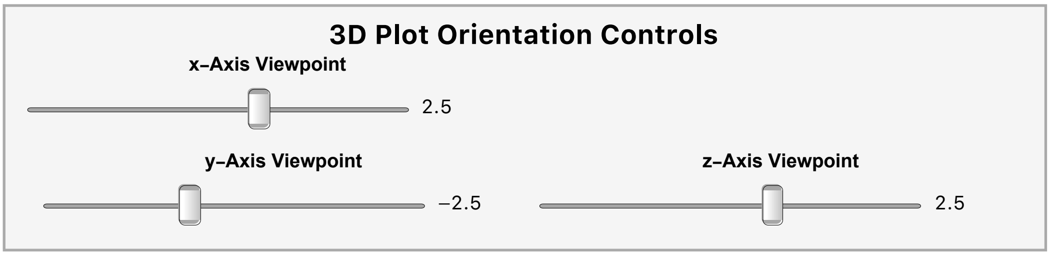

Export current 3 D plot.

```
filenameout = SystemDialogInput["FileSave"];
Export[filenameout, plt3D, "TIFF", ImageResolution → 150]
```

Calculate automated identifications.

Calculate distance table and ID summary (optional).

In[•]:=
```
projMDist = Table[0.0, {n4}, {numGroups}];
projDistTable = Table[0.0, {n4 + 1}, {numGroups + 3}];
projEScores = Take[projEScores, All, noAxes];

Do[
  Do[
   projMDist[[i, k]] = EuclideanDistance[gpCVMeans[[k]], projEScores[[i]]],
   {i, n4}],
  {k, numGroups}];
```

```
pDist = projMDist;
Do[
 n = 0;
 ref = Min[pDist[[k]]];
 Do[
  If[pDist[[k, j]] == ref, pDist[[k, j]] = Style[pDist[[k, j]], Bold]],
  {j, numGroups}],
 {k, n4}]

projDistTable[[1, 1]] = "Object";
projDistTable[[1, 2]] = "Given Group";
projDistTable[[1, 3]] = "Predicted Group";
Do[projDistTable[[1, j + 3]] = groupNames[[j]], {j, numGroups}];
Do[
 projDistTable[[i + 1, 1]] = projObjectNames[[i]];
 projDistTable[[i + 1, 2]] = projGroup[[i]];
 Do[
  ptr = 0;
  idCol = Min[projMDist[[i]]];
  Do[If[projMDist[[i, j]] == idCol, ptr = j], {j, numGroups}];
  projDistTable[[i + 1, 3]] = groupNames[[ptr]], {i, n4}];
 Do[projDistTable[[i + 1, j + 3]] =
   PaddedForm[pDist[[i, j]], {4, 3}], {j, numGroups}],
 {i, n4}]

distances = Labeled[
  Grid[projDistTable, BaseStyle → (FontFamily → "Arial"), Alignment → {Center},
   Frame → True, Dividers → {{True, True, True, True}, {True, True}}],
  "Projected Objects Distance Table", Top,
  LabelStyle → Directive[FontSize → 16, Bold, FontFamily → "Arial"]]

sumTable = Table[0, {3}, {numGroups + 1}];
sumTable[[1, 1]] = " ";
sumTable[[2, 1]] = "Raw Count";
sumTable[[3, 1]] = "Percentage";
Do[sumTable[[1, j + 1]] = groupNames[[j]], {j, numGroups}]
Do[Do[If[Min[projMDist[[i]]] == projMDist[[i, j]],
   sumTable[[2, j + 1]] = sumTable[[2, j + 1]] + 1], {j, numGroups}], {i, n4}]
Do[sumTable[[3, j + 1]] = N[(sumTable[[2, j + 1]] / n4) * 100, 4], {j, numGroups}]

summary = Labeled[
  Grid[sumTable, BaseStyle → (FontFamily → "Arial"), Frame → True, Dividers → All,
   Alignment → {{Left, Center}, {Left, Center}}], "ID Summary Table",
  Top, LabelStyle → Directive[FontSize → 16, Bold, FontFamily → "Arial"]]
```

Export group distance table.

```
filenameout = SystemDialogInput["FileSave"];
Export[filenameout, projDistTable, "CSV", "TextDelimiters" → ""]
```

Export unknown ID summary table.

```
filenameout = SystemDialogInput["FileSave"];
Export[filenameout, sumTable, "CSV", "TextDelimiters" → ""]
```

Executable modules

TWB : Calculates the Total [T] , Within [W], and Between [B] SSQ matrices

```
TWB[x2_, n1_, m1_, Group_] :=
  Module[{gndMean, T, sum, gNames, nGps, gpMeans, smpSize, knt, wDiff, W, B},
   gndMean = Mean[x2];
   T = Table[0.0, {m1}, {m1}];
   Do[
    Do[
      sum = 0.0;
      Do[
       sum = sum + ((x2[[i, j1]] - gndMean[[j1]]) * (x2[[i, j2]] - gndMean[[j2]])),
       {i, n1}];
      T[[j1, j2]] = sum,
      {j2, m1}];,
    {j1, m1}];

   grp = Group;
   gNames = DeleteDuplicates[Group];
   nGps = Length[gNames];
   gpMeans = Table[0.0, {nGps}, {m1}];
   smpSize = Table[0.0, {nGps}];

   Do[
     knt = 0;
     Do[
      If[Group[[i]] == gNames[[k]], knt = knt + 1, knt = knt],
      {i, n1}];
     smpSize[[k]] = knt,
     {k, nGps}] ×
    smpSize;

   Do[
```

```
    Do[
      sum = 0.0;
      Do[
       If[Group[[i]] == gNames[[k]],
        sum = sum + x2[[i, j]],
        sum = sum],
       {i, n1}];
      gpMeans[[k, j]] = sum / smpSize[[k]],
      {j, m1}];,
    {k, nGps}] ×
   gpMeans;

  wDiff = x2;
  Do[
    Do[
      Do[
        If[Group[[i]] == gNames[[k]],
         wDiff[[i, j]] = wDiff[[i, j]] - gpMeans[[k, j]],
         wDiff[[i, j]] = wDiff[[i, j]]],
        {i, n1}];,
      {j, m1}];,
    {k, nGps}] ×
   wDiff;

  W = Table[0.0, {m1}, {m1}];
  Do[
    Do[
      sum = 0.0;
      Do[
       sum = sum + (wDiff[[i, j1]] * wDiff[[i, j2]]),
       {i, n1}];
      W[[j1, j2]] = sum,
      {j2, m1}];,
    {j1, m1}] ×
   W;

  B = T - W;

  Return[{gndMean, T, gNames, nGps, gpMeans, smpSize, W, B}]
 ];
```
