# Supplementary material for: Machine-learning strategies for testing patterns of morphological variation in small samples: sexual dimorphism in gray wolf (Canis lupus) crania
Source: BMC Biol. 2020 Sep 3;18:113. doi: 10.1186/s12915-020-00832-1 (PMC7470621; doi:10.1186/s12915-020-00832-1)
Supplement: Supplementary file 5 — Additional file 5. An archive of all code listings for all data procession/analysis software employed in this investigation. [file 12915_2020_832_MOESM5_ESM.zip › SI File 5/Data Classification-ML (vers. 1.2).pdf]

Machine Learning – Group Classification

This program accepts a data matrix in standard format and applies a variety of machine – learning procedures to the data
in an effort to discriminate between groups.

Author : N. MacLeod
Version : 1.2
Date : 17 February 2018
Reference : xxx

Initialize libraries.

In[•]:=
```
<< ComputationalGeometry`
```

Read in data file & partition into datasets.

In[•]:=
```
filenamein = SystemDialogInput["FileOpen"];
x1 = Import[filenamein, "CSV"];
filenamein

{n1, m1} = Dimensions[x1];

varNames = Flatten[Take[x1, 1]];
x2 = Drop[x1, 1];
varNames = Drop[varNames, 1];
varNames = Drop[varNames, 1];

objNames = Flatten[Take[x2, n1 - 1, 1]];
x2 = Drop[x2, 0, 1];

Group = Flatten[Take[x2, n1 - 1, 1]];
x2 = Drop[x2, 0, 1];
numGroups = Length[Union[Group]];

{n2, m2} = Dimensions[x2];

Print["No. of groups: ", Length[Union[Group]]];
Print["No. of objects: ", n2];
Print["No. of variables: ", m2]
```

Data transformations (optional)

Specify global data transformations (if any).
Note : Remember you cannot subsequently take the logarithm of mean –

centered or standardized data. If you wish
to perform such an analysis you must shift the mean –
centered or standardized data by a constant (e.g., 1, 10)

In[•]:=
```
Panel[Labeled[Column[
   {Row[{Panel[Labeled[PopupMenu[Dynamic[meanTrans], {1 → "No", 2 → "Yes"}],
         "Mean center data?", Top, LabelStyle →
          Directive[FontSize → 12, Bold, FontFamily → "Arial"]]], "  ",
       Panel[Labeled[PopupMenu[Dynamic[logTrans], {1 → "No", 2 → "Yes"}],
         "Log10-trasform data?", Top, LabelStyle →
          Directive[FontSize → 12, Bold, FontFamily → "Arial"]]], "  ",
       Panel[Labeled[PopupMenu[Dynamic[stdTrans], {1 → "No", 2 → "Yes"}],
         "Standardize data?", Top,
         LabelStyle → Directive[FontSize → 12, Bold, FontFamily → "Arial"]]]}],
    Row[{Panel[Labeled[PopupMenu[Dynamic[shiftTrans], {1 → "No", 2 → "Yes"}],
         "Shift data by a constant?", Top, LabelStyle →
          Directive[FontSize → 12, Bold, FontFamily → "Arial"]]], "  ",
       Panel[Labeled[InputField[Dynamic[knsnt], FieldSize → 5],
         "Enter shift constant value.", Top,
         LabelStyle → Directive[FontSize → 12, Bold, FontFamily → "Arial"]]]}]},
   Center], "Global Data Transform Options", Top, LabelStyle →
   Directive[FontSize → 18, Bold, FontFamily → "Arial"]]]
meanTrans = 1; logTrans = 1; stdTrans = 1; shiftTrans = 1; knsnt = 10;
```

Out[•]=

**Global Data Transform Options**

| Mean center data? | $Log_{10}$-trasform data? | Standardize data? |
|---|---|---|
| Yes | No | No |

| Shift data by a constant? | Enter shift constant value. |
|---|---|
| No | 10 |

Perform global data transformation (optional)

In[•]:=
```
If[meanTrans == 2, mVec = N[Mean[x2]];
  Do[x2[[i]] = x2[[i]] - mVec, {i, n2}]];
If[logTrans == 2, x2 = N[Log10[x2]]];
If[stdTrans == 2, x2 = Standardize[x2]];
If[shiftTrans == 2, x2 = x2 * knsnt];
```

Export mean vector (optional;  may be useful in modelling).

```
filenameout = SystemDialogInput["FileSave"];
Export[filenameout, mVec, "csv"]
```

Export processed dataset (optional; may be useful in other analyses).

```
x2Trans = Table[" ", {n2 + 1}, {m2 + 2}];
x2Trans[[1, 1]] = "Object";
x2Trans[[1, 2]] = "Group";
Do[x2Trans[[1, j + 2]] = varNames[[j]], {j, m2}]
Do[x2Trans[[i + 1, 1]] = objNames[[i]], {i, n2}]
Do[x2Trans[[i + 1, 2]] = Group[[i]], {i, n2}]
Do[x2Trans[[i + 1, j + 2]] = x2[[i, j]], {i, n2}, {j, m2}]

filenameout = SystemDialogInput["FileSave"];
Export[filenameout, x2Trans, "CSV", "TextDelimiters" → ""]
```

Plot single – axis histogram.

Specify single – variable plot (histogram) options.

In[∘]:=
```
Panel[Labeled[Column[{
    Row[{Panel[Labeled[PopupMenu[Dynamic[axisName], varNames],
        "Select variable to be plotted on x-Axis.", Top,
        LabelStyle → Directive[FontSize → 12, Bold, FontFamily → "Arial"]]],
      "  ", Panel[Labeled[PopupMenu[Dynamic[histType],
         {1 → "Stacked", 2 → "Overlapped"}], "Select histogram type.", Top,
        LabelStyle → Directive[FontSize → 12, Bold, FontFamily → "Arial"]]]}],
    Row[{Panel[Labeled[InputField[Dynamic[noBins], FieldSize → 5],
        "Enter no. of histogram bins.", Top, LabelStyle →
         Directive[FontSize → 12, Bold, FontFamily → "Arial"]]], "  ",
      Panel[Labeled[InputField[Dynamic[hSize], FieldSize → 5],
        "Enter histogram plot size.", Top,
        LabelStyle → Directive[FontSize → 12, Bold, FontFamily → "Arial"]]]}]},
   Center], "Single Axis (Histogram) Plot Options", Top,
  LabelStyle → Directive[FontSize → 16, Bold, FontFamily → "Arial"]]]
axisName = varNames[[1]]; noBins = 15; hSize = 500; histType = 1;
```

Out[∘]=

**Single Axis (Histogram) Plot Options**

| Select variable to be plotted on x-Axis. | Select histogram type. |
|---|---|
| PopupMenu[PC–1, varNames] | Stacked |
| **Enter no. of histogram bins.** | **Enter histogram plot size.** |
| 15 | 500 |

Construct and display histogram plot .

```
Do[If[axisName == varNames[[j]], axis = j], {j, m2}];
pltScores = Flatten[Take[x2, All, {axis}]];
If[histType == 1, htype = "Stacked", htype = "Overlapped"];
gpNames = Union[Group];
groupPosns = Table[Flatten[Position[Group, gpNames[[i]], 1]], {i, numGroups}];
pltPoints = Table[pltScores[[groupPosns[[j]]]], {j, numGroups}];
hueList = Table[Hue[N[((numGroups + 1) - j) / numGroups]], {j, numGroups}];
h1 =
  Labeled[Histogram[pltPoints, noBins, ChartStyle → {hueList}, ChartLayout → htype,
    LabelStyle → Directive[FontSize → 12, Black, FontFamily → "Arial"],
    AxesLabel → {"Discrim. Score", "Frequency"}, ImageSize → hSize,
    ChartLegends → gpNames], varNames[[axis]], Top,
   LabelStyle → Directive[FontSize → 16, Bold, FontFamily → "Arial"]]
```

Export current histogram.

```
filenameout = SystemDialogInput["FileSave"];
Export[filenameout, h1, "TIFF", ImageResolution → 150]
```

```
/Users/nm/Desktop/Varotis Results/CVA Results/CV-1.tif
```

Create 2 D scatterplot (use only for datasets containing three or more groups).

Specify 2 D plot options.
You must run this code after you read in the data so it can pick up the proper variable names.

```
In[•]:= Panel[
  Labeled[Column[{Row[{Panel[Labeled[PopupMenu[Dynamic[xAxisName], varNames],
        "Select variable to be plotted on x-Axis.", Top, LabelStyle →
         Directive[FontSize → 12, Bold, FontFamily → "Arial"]]], "  ",
      Panel[Labeled[PopupMenu[Dynamic[yAxisName], varNames],
        "Select variable to be plotted on y-Axis.", Top,
        LabelStyle → Directive[FontSize → 12, Bold, FontFamily → "Arial"]]]}],
    Row[{
      Panel[Labeled[PopupMenu[Dynamic[pltAspect],
         {1 → "Golden Ratio Plot", 2 → "Square Plot (equi-length axes)",
          3 → "True-Scale Plot (actual axis scales)"}],
        "Enter plot aspect ratio type.", Top, LabelStyle →
         Directive[FontSize → 12, Bold, FontFamily → "Arial"]]], "  ",
      Panel[Labeled[PopupMenu[Dynamic[lch], {1 → "Simple scatterplot",
          2 → "Scatterplot w/ convex hulls"}],
        "Show group domians?", Top, LabelStyle →
         Directive[FontSize → 12, Bold, FontFamily → "Arial"]]], "  ",
      Panel[Labeled[PopupMenu[Dynamic[ptsJoin], {1 → "No", 2 → "Yes"}],
        "Join datapoints?", Top,
        LabelStyle → Directive[FontSize → 12, Bold, FontFamily → "Arial"]]]}],
    Row[{Panel[Labeled[InputField[Dynamic[pltSize], FieldSize → 10],
        "Enter plot size value.", Top,
        LabelStyle → Directive[FontSize → 12, Bold, FontFamily → "Arial"]]],
      "  ", Panel[Labeled[InputField[Dynamic[pltPad], FieldSize → 10],
        "Enter plot margin padding value.", Top, LabelStyle →
         Directive[FontSize → 12, Bold, FontFamily → "Arial"]]], "  ",
      Panel[Labeled[InputField[Dynamic[iconSize], FieldSize → 10],
        "Enter plot icon size value.", Top, LabelStyle →
         Directive[FontSize → 12, Bold, FontFamily → "Arial"]]]}]}, Center],
   "2D Plot Options", Top, LabelStyle → Directive[FontSize → 18,
     Bold, FontFamily → "Arial"]]]
pltSize = 500; iconSize = 0.03; pltPad = 0.1; xAxisName = varNames[[1]];
yAxisName = varNames[[2]];
 ptsJoin = 1; dataTrans = 1;  pltAspect = 1 ; lch = 1;
```

Out[∘]=

## 2D Plot Options

**Select variable to be plotted on x–Axis.** PC–1

**Select variable to be plotted on y–Axis.** PC–2

**Enter plot aspect ratio type.** True–Scale Plot (actual axis scales)

**Show group domians?** Scatterplot w/ convex hulls

**Join datapoints?** No

**Enter plot size value.** 500

**Enter plot margin padding value.** 0.1

**Enter plot icon size value.** 30.

Plot script

In[∘]:=

```
pltTable = Table[" ", {numGroups}, {3}];
Do[If[xAxisName == varNames[[j]], axis1 = j], {j, m2}];
Do[If[yAxisName == varNames[[j]], axis2 = j], {j, m2}];

groupNames = Union[Group];
numGroups = Length[groupNames];
groupPosns =
  Table[Flatten[Position[Group, groupNames[[i]], 1]], {i, numGroups}];
x2T = Transpose[x2];

xAxis = x2T[[axis1]]; yAxis = x2T[[axis2]];
lab1 = varNames[[axis1]];
lab2 = varNames[[axis2]];
maxx = Max[xAxis];
minx = Min[xAxis];
maxy = Max[yAxis];
miny = Min[yAxis];

If[pltAspect == 1 || pltAspect == 3,
  xPlotLow = minx; xPlotHi = maxx; yPlotLow = miny; yPlotHi = maxy];
If[pltAspect == 2,
  If[minx > miny,
   xPlotLow = miny; yPlotLow = miny,
   xPlotLow = minx; yPlotLow = minx ]];
If[pltAspect == 2,
  If[maxx < maxy,
```

```
    xPlotHi = maxy; yPlotHi = maxy,
    xPlotHi = maxx; yPlotHi = maxx]];
If[pltAspect == 1, aRatio = 1 / N[GoldenRatio]];
If[pltAspect == 2, aRatio = 1];
If[pltAspect == 3, aRatio = Automatic];

tmpPoints = Transpose[List[xAxis, yAxis]];
pltPoints = Table[tmpPoints[[groupPosns[[j]]]], {j, numGroups}];
iconList = Flatten[Table[
    {Graphics[{EdgeForm[{Thin, Black}], Hue[N[((numGroups + 1) - j) / numGroups]],
        Disk[{0, 0}, Scaled[iconSize]]}]}, {j, numGroups}]];

If[lch == 1 || ptsJoin == 1,
 Do[
  pltTable[[k, 1]] = pTmp =
    ListPlot[pltPoints[[k]], AspectRatio → aRatio, Frame → True, Joined → False,
     Axes → False, PlotRange → {{xPlotLow, xPlotHi}, {yPlotLow, yPlotHi}},
     PlotRangePadding → Scaled[pltPad], Ticks → Automatic, FrameLabel →
      {lab1, lab2}, PlotMarkers → iconList[[k]], ImageSize → pltSize, LabelStyle →
      Directive[FontSize → 14, Black, FontFamily → "Arial"]], {k, numGroups}],
 Do[
  pltTable[[k, 1]] =
   ListPlot[pltPoints[[k]], Frame → True, Axes → False, AspectRatio → aRatio,
    PlotRange → {{xPlotLow, xPlotHi}, {yPlotLow, yPlotHi}}, PlotRangePadding →
     Scaled[pltPad], Ticks → Automatic, FrameLabel → {lab1, lab2},
    LabelStyle → Directive[Black, FontSize → 14, FontFamily → "Arial"],
    ImageSize → pltSize, PlotStyle → Directive[Disk[],
      Hue[N[((numGroups + 1) - k) / numGroups]], EdgeForm[{Thickness[1.0], Black}],
      PointSize[Scaled[iconSize - 0.009]]]], {k, numGroups}]]

If[ptsJoin == 2,
  Do[
   pltTable[[k, 2]] = ListLinePlot[pltPoints[[k]],
     AspectRatio → aRatio, Frame → True, Joined → True, Axes → False,
     PlotStyle → Directive[Hue[N[((numGroups + 1) - k) / numGroups]], Thin],
     PlotRange → {{xPlotLow, xPlotHi}, {yPlotLow, yPlotHi}},
     PlotRangePadding → Scaled[pltPad], Ticks → Automatic,
     FrameLabel → {lab1, lab2}, ImageSize → pltSize, LabelStyle →
      Directive[FontSize → 14, Black, FontFamily → "Arial"]], {k, numGroups}]];

If[lch == 2,
  Do[
   hull = ConvexHullMesh[pltPoints[[k]]];
   pltTable[[k, 3]] = HighlightMesh[hull,
     Style[2, Opacity[0.2], Hue[N[((numGroups + 1) - k) / numGroups]]],
     Frame → True, Axes → False, AspectRatio → aRatio,
     PlotRange → {{xPlotLow, xPlotHi}, {yPlotLow, yPlotHi}}, PlotRangePadding →
```

```
     Scaled[pltPad], Ticks → Automatic, FrameLabel → {lab1, lab2},
     LabelStyle → Directive[Black, FontSize → 14, FontFamily → "Arial"],
     ImageSize → pltSize], {k, numGroups}]];

If[ptsJoin == 1 && lch == 1, p0 = Show[pltTable[[All, 1]]]];
If[ptsJoin == 2 && lch == 1, p0 = Show[pltTable[[All, 2]], pltTable[[All, 1]]]];
If[ptsJoin == 1 && lch == 2, p0 = Show[pltTable[[All, 3]], pltTable[[All, 1]]]];
If[ptsJoin == 2 && lch == 2,
  p0 = Show[pltTable[[All, 3]], pltTable[[All, 2]], pltTable[[All, 1]]]];

p1 = Labeled[p0, "          Variable Score Plot", Top,
   LabelStyle → Directive[FontSize → 18, Bold, FontFamily → "Arial"]];
g1 = Grid[Table[
    {Graphics[{EdgeForm[{Thin, Black}], Hue[N[((numGroups + 1) - j) / numGroups]],
       Disk[]}]}, {j, numGroups}], Frame → False, ItemSize → 0.9];
g2 = Grid[Partition[groupNames, 1], Alignment → Left,
   BaseStyle → {FontFamily → "Arial", FontSize → 13, Italic}];
p2 = Labeled[Text[Grid[{{g1, g2}}, Alignment → Bottom, Frame → True]], "Legend",
   Top, LabelStyle → Directive[Black, FontSize → 18, Bold, FontFamily → "Arial"]];

plt2D = Grid[{{p1, p2}}, BaselinePosition → Top, Alignment → Top]
```

Export current 2 D plot.

```
filenameout = SystemDialogInput["FileSave"];
Export[filenameout, plt2D, "TIFF", ImageResolution → 150]
```

Label plotted points.

```
pn1 = p0;
namePoints = tmpPoints;
tempPointsT = Transpose[tmpPoints];
mxY = Max[tempPointsT[[2]]];
mnY = Min[tempPointsT[[2]]];
incY = N[(mxY - mnY) / 15];
Do[namePoints[[i, 2]] = tmpPoints[[i, 2]] - incY, {i, n2}]
nPointsTable = Table[{Text[objNames[[i]], namePoints[[i]], {-1, 0}]}, {i, n2}];
pn2 = Graphics[nPointsTable, Frame → True, AspectRatio → aRatio , Axes → False,
    FrameLabel  → {lab1, lab2}, PlotRangePadding → Scaled[pltPad], BaseStyle →
     Directive[FontSize → 12, FontFamily → "Arial"], ImageSize → pltSize];

p1 = Labeled[Show[pn1, pn2, BaseStyle → {FontFamily → "Arial"}],
    "          Variable Score Plot", Top,
    LabelStyle → Directive[FontSize → 18, Bold, FontFamily → "Arial"]];

Plt2D = Grid[{{p1, p2}}, BaselinePosition → Top, Alignment → Top]
```

Export current 2 D plot.

```
filenameout = SystemDialogInput["FileSave"];
Export[filenameout, Plt2D, "TIFF", ImageResolution → 150]
```

Create 3 D scatterplot (use only for datasets containing four or more groups).

Specify 3 D plot options.
You must run this code after you read in the data so it can pick up the proper variable names.

In[•]:=
```
Panel[
 Labeled[Column[{Row[{Panel[Labeled[PopupMenu[Dynamic[xAxisName], varNames],
       "Select variable to be plotted on x-Axis.", Top, LabelStyle →
        Directive[FontSize → 12, Bold, FontFamily → "Arial"]]], "  ",
     Panel[Labeled[PopupMenu[Dynamic[yAxisName], varNames],
       "Select variable to be plotted on y-Axis.", Top, LabelStyle →
        Directive[FontSize → 12, Bold, FontFamily → "Arial"]]], "  ",
     Panel[Labeled[PopupMenu[Dynamic[zAxisName], varNames],
       "Select variable to be plotted on z-Axis.", Top,
       LabelStyle → Directive[FontSize → 12, Bold, FontFamily → "Arial"]]]}],
   Row[{
     Panel[Labeled[PopupMenu[Dynamic[pltAspect],
        {1 → "Golden Ratio Plot", 2 → "Square Plot (equi-length axes)",
         3 → "True-Scale Plot (actual axis scales)"}],
       "Enter plot aspect ratio type.", Top, LabelStyle →
        Directive[FontSize → 12, Bold, FontFamily → "Arial"]]], "  ",
     Panel[Labeled[PopupMenu[Dynamic[lch], {1 → "Simple scatterplot",
         2 → "Scatterplot w/ convex hulls"}],
       "Show group domians?", Top, LabelStyle →
        Directive[FontSize → 12, Bold, FontFamily → "Arial"]]], " ",
     Panel[Labeled[PopupMenu[Dynamic[ptsJoin], {1 → "No", 2 → "Yes"}],
       "Join datapoints?", Top,
       LabelStyle → Directive[FontSize → 12, Bold, FontFamily → "Arial"]]]}],
   Row[{Panel[Labeled[InputField[Dynamic[pltSize], FieldSize → 5],
       "Enter plot size value.", Top,
       LabelStyle → Directive[FontSize → 12, Bold, FontFamily → "Arial"]]],
     " ", Panel[Labeled[InputField[Dynamic[pltPad], FieldSize → 5],
       "Enter plot margin padding value.", Top, LabelStyle →
        Directive[FontSize → 12, Bold, FontFamily → "Arial"]]], " ",
     Panel[Labeled[InputField[Dynamic[iconSize3D], FieldSize → 5],
       "Enter plot icon size value.", Top, LabelStyle →
        Directive[FontSize → 12, Bold, FontFamily → "Arial"]]]}]}, Center],
  "3D Plot Options", Top, LabelStyle → Directive[FontSize → 18,
    Bold, FontFamily → "Arial"]]]
pltSize = 500; iconSize3D = 60; pltPad = 0.1; xAxisName = varNames[[1]];
 yAxisName = varNames[[2]];
zAxisName = varNames[[3]]; ptsJoin = 1;
dataTrans = 1; pltAspect = 3 ;
lch = 1;
```

Out[ ]=

**3D Plot Options**

**Select variable to be plotted on x–Axis.**
PopupMenu[PC–1, varNames]

**Select variable to be plotted on y–Axis.**
PopupMenu[PC–2, varNames]

**Select variable to be plotted on z–Axis.**
PopupMenu[PC–3, varNames]

**Enter plot aspect ratio type.**
True–Scale Plot (actual axis scales)

**Show group domians?**
Scatterplot w/ convex hulls

**Join datapoints?**
No

**Enter plot size value.**
500

**Enter plot margin padding value.**
0.1

**Enter plot icon size value.**
10

## Plot script

```
Do[If[xAxisName == varNames[[j]], axis1 = j], {j, m2}]
Do[If[yAxisName == varNames[[j]], axis2 = j], {j, m2}]
Do[If[zAxisName == varNames[[j]], axis3 = j], {j, m2}]
iconSize = iconSize3D - 50.0;

groupNames = Union[Group];
numGroups = Length[groupNames];
groupPosns =
  Table[Flatten[Position[Group, groupNames[[i]], 1]], {i, numGroups}];
x2T = Transpose[x2];

xAxis = x2T[[axis1]]; yAxis = x2T[[axis2]]; zAxis = x2T[[axis3]];
lab1 = varNames[[axis1]];
lab2 = varNames[[axis2]];
lab3 = varNames[[axis3]];
maxx = Max[xAxis];
minx = Min[xAxis];
maxy = Max[yAxis];
miny = Min[yAxis];
maxz = Max[zAxis]; minz = Min[zAxis];

If[pltAspect == 1 || pltAspect == 3,
  xPlotLow = minx; xPlotHi = maxx; yPlotLow = miny; yPlotHi = maxy];
If[pltAspect == 2,
```

```
    If[minx > miny,
     xPlotLow = miny; yPlotLow = miny,
     xPlotLow = minx; yPlotLow = minx ]];
  If[pltAspect == 2,
    If[maxx < maxy,
     xPlotHi = maxy; yPlotHi = maxy,
     xPlotHi = maxx; yPlotHi = maxx]];

  If[pltAspect == 1, bRatio = {1.61803, 1, 1}];
  If[pltAspect == 2, bRatio = {1, 1, 1}];
  If[pltAspect == 3, bRatio = Automatic];

  points3 = Transpose[List[xAxis, yAxis, zAxis]];
  gp1 = groupPosns;
  h = Table[0, {n2}];
  Do[
   tmp = gp1[[i]];
   itr = Length[tmp];
   Do[h[[tmp[[j]]]] = Hue[N[((numGroups + 1) - i) / numGroups]], {j, itr}],
   {i, numGroups}]

  If[lch == 1 && pltAspect == 3,
    pltPoints = Table[{h[[i]], Sphere[points3[[i]], iconSize3D / 55]}, {i, n2}],
    pltPoints =
     Table[{h[[i]], AbsolutePointSize[iconSize], Point[points3[[i]]]}, {i, n2}]];
  p0 = Graphics3D[pltPoints, Axes → True, Boxed → True,
     PlotRangePadding → Scaled[pltPad],
     LabelStyle → Directive[FontSize → 12, Black, FontFamily → "Arial"],
     AxesLabel → {lab1, lab2, lab3}, ImageSize → pltSize, BoxRatios → bRatio];

  If[ptsJoin == 2,
    pltLineTable = Table[" ", {numGroups}];
    gpPoints = Table[points3[[groupPosns[[j]]]], {j, numGroups}];
    Do[
     pltLineTable[[k]] =
      Graphics3D[{Hue[N[((numGroups + 1) - k) / numGroups]], Line[gpPoints[[k]]]},
       Axes → True, Boxed → True, PlotRangePadding → Scaled[pltPad],
       LabelStyle → Directive[FontSize → 12, Black, FontFamily → "Arial"],
       AxesLabel → {lab1, lab2, lab3}, ImageSize → pltSize,
       BoxRatios → bRatio, ViewPoint -> {xax, yax, zax}], {k, numGroups}];
    pTmp = Graphics3D[pltPoints, Axes → True, Boxed → True,
      PlotRangePadding → Scaled[pltPad],
      LabelStyle → Directive[FontSize → 12, Black, FontFamily → "Arial"],
      AxesLabel → {lab1, lab2, lab3}, ImageSize → pltSize,
      BoxRatios → bRatio, ViewPoint -> {xax, yax, zax}];
    p0 = Show[{pltLineTable, pTmp}]];
```

```
If[lch == 2,
  pltMeshTable = Table[" ", {numGroups}];
  gpPoints = Table[points3[[groupPosns[[j]]]], {j, numGroups}];
  Do[
   {n3, m3} = Dimensions[gpPoints[[k]]];
   pltPoints = Table[{Hue[N[((numGroups + 1) - k) / numGroups]],
      AbsolutePointSize[iconSize3D], Point[gpPoints[[k, i]]]}, {i, n3}];
   pTmp = Graphics3D[pltPoints, Axes → True, Boxed → True,
     PlotRangePadding → Scaled[pltPad],
     LabelStyle → Directive[FontSize → 12, Black, FontFamily → "Arial"],
     AxesLabel → {lab1, lab2, lab3}, ImageSize → pltSize,
     BoxRatios → bRatio, ViewPoint -> {xax, yax, zax}];
   cHull3D = ConvexHullMesh[gpPoints[[k]], BaseStyle → {EdgeForm[]},
     Boxed → True, Axes → True, PlotRangePadding → Scaled[pltPad],
     LabelStyle → Directive[FontSize → 12, Black, FontFamily → "Arial"],
     AxesLabel → {lab1, lab2, lab3}, ImageSize → pltSize,
     BoxRatios → bRatio, ViewPoint -> {xax, yax, zax}];
   pltMeshTable[[k]] = Show[{HighlightMesh[cHull3D, Style[2, Opacity[0.2],
        Hue[N[((numGroups + 1) - k) / numGroups]]]], p0}], {k, numGroups}];
  p0 = Show[pltMeshTable];

p1 = Labeled[p0, "           Variable Score Plot", Top,
   LabelStyle → Directive[FontSize → 18, Bold, FontFamily → "Arial"]];
If[lch == 1 && pltAspect == 3, g1 = Table[Graphics[
     {Inset[Graphics3D[{Hue[N[((numGroups + 1) - i) / numGroups]], Sphere[]},
        Boxed → False]]}], {i, numGroups}],
  g1 = Table[Graphics[{Inset[Graphics[{Hue[N[((numGroups + 1) - i) / numGroups]],
          Disk[{0, 0}, Scaled[0.3]]}]]}], {i, numGroups}]];
g2 = Labeled[Text[Grid[Transpose[Partition[Join[g1, groupNames], numGroups]],
     ItemSize → {{Scaled[0.03], Automatic}}, Alignment → Left, BaseStyle →
      {FontSize → 14, FontFamily → "Arial", Italic}, Frame → True]], "Legend",
   Top, LabelStyle → Directive[FontSize → 16, Bold, FontFamily → "Arial"]];

plt3D = Grid[{{p1, g2}}, BaselinePosition → Top, Alignment → Top]
```

Adjust orientation of 3 D plot (if necessary).

You must replot the data to activate the changes. These changes will be able to be exported using the script below.

In[∘]:=
```
Panel[
 Labeled[Row[{Labeled[Slider[Dynamic[xax], {-10, 10}, Appearance → "Labeled"],
      "x-Axis Viewpoint", Top,
      LabelStyle → Directive[FontSize → 10, Bold, FontFamily → "Arial"]] ×
     Labeled[Slider[Dynamic[yax], {-10, 10}, Appearance → "Labeled"],
      "y-Axis Viewpoint", Top,
      LabelStyle → Directive[FontSize → 10, Bold, FontFamily → "Arial"]] ×
     Labeled[Slider[Dynamic[zax], {-10, 10}, Appearance → "Labeled"],
      "z-Axis Viewpoint", Top,
      LabelStyle → Directive[FontSize → 10, Bold, FontFamily → "Arial"]]}],
  "3D Plot Orientation Controls", Top, LabelStyle →
   Directive[FontSize → 14, Bold, FontFamily → "Ariel"]]]
xax = 2.5; yax = -2.5; zax = 2.5;
```

Out[∘]=

**3D Plot Orientation Controls**

x–Axis Viewpoint 2.5

y–Axis Viewpoint −2.5

z–Axis Viewpoint 2.5

Export current 3 D plot.

```
filenameout = SystemDialogInput["FileSave"];
Export[filenameout, plt3D, "TIFF", ImageResolution → 150]
```

```
/Users/nm/Projects/Storage/MacLeod/Manuscripts/
  Submitted/Morphometric Assessment of Archaeological Object
  Groups/New Manuscript/Data & Results/Eigenimage Results/No &
  MInor Damage Data & Results/CVA Results/CV-1 vs CV-2 vs CV-3.tif
```

Allow Mathematica to estimate the best classification method for your data.

In[∘]:=
```
class2 = Classify[x2 → Group];
cm = ClassifierMeasurements[class2, x2 → Group]
cm["ConfusionMatrixPlot"]
cm["Accuracy"] * 100
```

Out[∘]= ClassifierMeasurementsObject[ Classifier: NearestNeighbors
Number of test examples: 180 ]

Select classification method.

In[•]:=
```
Panel[Labeled[PopupMenu[Dynamic[cMethod], {1 → "Logistic Regression (Linear)",
     2 → "Naïve Bayes (Independent Features)",
     3 → "Nearest Neighbors (Linear)", 4 → "Neural Network (Non-Linear)",
     5 → "Random Forest (Non-Linear)", 6 → "Support Vectors (Non-Linear)"},
    7 → "Gradient Boosted Trees (Non-Linear)"], "Choose Classification Method",
  Top, LabelStyle → Directive[FontSize → 12, Bold, FontFamily → "Arial"]]]
cMethod = 1;
```

Out[•]=

**Choose Classification Method**

Logistic Regression (Linear)

## Calculate classification functions

In[•]:=
```
If[cMethod == 1, cf = "LogisticRegression"];
If[cMethod == 2, cf = "NaiveBayes"];
If[cMethod == 3, cf = "NearestNeighbors"];
If[cMethod == 4, cf = "NeuralNetwork"];
If[cMethod == 5, cf = "RandomForest"];
If[cMethod == 6, cf = "SupportVectorMachine"];
If[cMethod == 7, cf = "GradientBoostedTrees"];

class = Classify[x2 → Group, Method → cf]
```

Out[•]=
```
ClassifierFunction[ Input type: NumericalVector (length: 91)
                    Number of classes: 7 ]
```

## Calculate and display raw training set confusion matrix.

In[•]:=
```
groupNames = Union[Group];
numGroups = Length[groupNames];
nGroups = numGroups;
gpNames = groupNames;
cmat = ClassifierMeasurements[class, x2 → Group, "ConfusionMatrix"];
cmat = Take[cmat, All, nGroups];

cmatOut = Table[" ", {nGroups + 4}, {nGroups + 4}];
cmatOut[[1, 1]] = "Groups";
Do[cmatOut[[i + 1, 1]] = gpNames[[i]];
  cmatOut[[1, i + 1]] = gpNames[[i]], {i, nGroups}]
Do[cmatOut[[i + 1, j + 1]] = cmat[[i, j]], {i, nGroups}, {j, nGroups}]
cmatOut[[1, nGroups + 2]] = "No. Correct";
cmatOut[[1, nGroups + 3]] = "Actual";
cmatOut[[1, nGroups + 4]] = "Percent Correct";
cmatOut[[nGroups + 2, 1]] = "No. Correct";
cmatOut[[nGroups + 3, 1]] = "Predicted";
```

```
cmatOut[[nGroups + 4, 1]] = "Predicted Correct";
Do[cmatOut[[i + 1, nGroups + 2]] = cmat[[i, i]], {i, nGroups}]
Do[cmatOut[[i + 1, nGroups + 3]] = Total[cmat[[i]]], {i, nGroups}]
Do[
 If[cmatOut[[i + 1, nGroups + 3]] == 0,
  cmatOut[[i + 1, nGroups + 4]] = PaddedForm[0.0, {4, 2}],
  cmatOut[[i + 1, nGroups + 4]] =
   PaddedForm[(cmatOut[[i + 1, nGroups + 2]] / cmatOut[[i + 1, nGroups + 3]]) * 100.0,
    {4, 2}]], {i, nGroups}]
Do[cmatOut[[nGroups + 2, i + 1]] = cmat[[i, i]], {i, nGroups}]
Do[cmatOut[[nGroups + 3, i + 1]] = Total[cmat[[All, i]]], {i, nGroups}]
Do[
 If[cmatOut[[nGroups + 3, i + 1]] == 0,
  cmatOut[[nGroups + 4, i + 1]] = PaddedForm[0.0, {4, 2}],
  cmatOut[[nGroups + 4, i + 1]] =
   PaddedForm[(cmatOut[[nGroups + 2, i + 1]] / cmatOut[[nGroups + 3, i + 1]]) * 100.0,
    {4, 2}]], {i, nGroups}]
cmatOut[[nGroups + 2, nGroups + 2]] = Total[Diagonal[cmat]];
cmatOut[[nGroups + 2, nGroups + 3]] = Sum[Total[cmat[[All, i]]], {i, nGroups}];
cmatOut[[nGroups + 3, nGroups + 2]] = Sum[Total[cmat[[i, All]]], {i, nGroups}];
cmatOut[[nGroups + 4, nGroups + 2]] = PaddedForm[
   (cmatOut[[nGroups + 2, nGroups + 2]] / cmatOut[[nGroups + 3, nGroups + 2]]) * 100.0,
   {4, 2}];
If[cmatOut[[nGroups + 2, nGroups + 3]] == 0,
  cmatOut[[nGroups + 2, nGroups + 4]] = PaddedForm[0.0, {4, 2}],
  cmatOut[[nGroups + 2, nGroups + 4]] = PaddedForm[
    (cmatOut[[nGroups + 2, nGroups + 2]] / cmatOut[[nGroups + 2, nGroups + 3]]) *
     100.0, {4, 2}]];

Labeled[Grid[cmatOut,
  BaseStyle → (FontFamily → "Arial"), Alignment → {{Right}, {Center}},
  Dividers → {{2 → True, -4 → True}, {2 → True, -4 → True}}, Frame → True,
  BaseStyle → Directive[FontSize → 12, FontFamily → "Arial"]],
 "Confusion Matrix: Training Set", Top,
 LabelStyle → Directive[FontSize → 16, Bold, FontFamily → "Arial"]]
```

Export raw training set confusion matrix (optional).

In[∘]:=
```
outTab = cmatOut;
Do[
 outTab[[i + 1, nGroups + 4]] =
  (cmatOut[[i + 1, nGroups + 2]] / cmatOut[[i + 1, nGroups + 3]]) * 100.0;
 outTab[[nGroups + 4, i + 1]] =
  (cmatOut[[nGroups + 2, i + 1]] / cmatOut[[nGroups + 3, i + 1]]) * 100.0, {i, nGroups}]
outTab[[nGroups + 4, nGroups + 2]] =
  (cmatOut[[nGroups + 2, nGroups + 2]] / cmatOut[[nGroups + 3, nGroups + 2]]) * 100.0;
outTab[[nGroups + 2, nGroups + 4]] =
  (cmatOut[[nGroups + 2, nGroups + 2]] / cmatOut[[nGroups + 2, nGroups + 3]]) * 100.0;
filenameout = SystemDialogInput["FileSave"];
Export[filenameout, outTab, "CSV", "TextDelimiters" → ""]
```

### Calculate parameteric estimate of log – likelihood ratio index

In[∘]:=
```
cm = ClassifierMeasurements[class, x2 → Group];
cm["LogLikelihood"]
cm["MisclassifiedExamples"]
```

### Set parameters for bootstrapped estimate of log – likelihood ratio index

In[∘]:=
```
Panel[Labeled[Row[{Panel[Labeled[InputField[Dynamic[iter], FieldSize → 10],
      "Enter number of pseudoreplicate iterations.", Top,
      LabelStyle → Directive[FontSize → 12, FontFamily → "Arial"]]], "   ",
    Panel[Labeled[InputField[Dynamic[hsize], FieldSize → 10],
      "Enter histogram size.", Top,
      LabelStyle → Directive[FontSize → 12, FontFamily → "Arial"]]]}],
  "Log-Likelihood Ratio Test", Top, LabelStyle →
   Directive[FontSize → 14, Bold, FontFamily → "Arial"]]]
hsize = 500; iter = 1000;
```

Out[∘]=

**Log–Likelihood Ratio Test**

| Enter number of pseudoreplicate iterations. | Enter histogram size. |
|---|---|
| iter | hsize |

### Calculate bootstrapped estimate of log – likelihood ratio test.

In[∘]:=
```
s1 = Table[0, {n2}];
phi = ClassifierMeasurements[class, x2 → Group, "LogLikelihood"];
phiTable = Table[0.0, {iter}];
x3 = x2;
Panel[Labeled[ProgressIndicator[Dynamic[k], {1, iter}], "Calculation Progress",
  Top, LabelStyle → Directive[FontSize → 12, FontFamily → "Arial"]]]
Do[
 Do[s1[[i]] = RandomInteger[{1, n2}], {i, n2}];
 Do[x3[[i]] = x2[[s1[[i]]]], {i, n2}];

 classBS = Classify[x3 → Group, Method → cf, TrainingProgressReporting → None];
 phiTable[[k]] = ClassifierMeasurements[classBS, x3 → Group, "LogLikelihood"],
 {k, iter}]

Print[" "];
phi = phi * -1;
phiTable = phiTable * -1;
phiTable = Sort[phiTable];
mk = 0;
Do[If[phiTable[[i]] ≤ phi, mk = mk + 1];, {i, iter}];
prob = (1 - N[mk / iter]) * 100;

pTable = Table [" ", {3}, {2}];
pTable [[1, 1]] = "Observed ϕ-value";
pTable[[2, 1]] = "Pseudoreplicate Iterations";
pTable[[3, 1]] = "Probability (%)";
pTable[[1, 2]] = PaddedForm[phi, {4, 3}];
pTable[[2, 2]] = iter;
pTable[[3, 2]] = prob;

hPlot = Labeled[Histogram[phiTable, "FreedmanDiaconis", ChartStyle → {Green},
     LabelStyle → Directive[FontSize → 12, FontFamily → "Arial"],
     AxesLabel → {"ϕ-Value", "Frequency"}, ImageSize → 500],
   "Bootstrapped Log-Likelihood Ratio Distribution", Top,
   LabelStyle → Directive[FontSize → 16, FontFamily → "Arial"]];
hLegend = Labeled[Grid[pTable, BaseStyle → (FontFamily → "Arial"),
     Alignment → {{Left, Right}}, Frame → True, Dividers → {{True, True}, {True}}],
   "Probability Table", Top, LabelStyle → (FontFamily → "Arial")];
probPlot = Grid[{{hPlot, hLegend}}, BaselinePosition → Top, Alignment → Top]
```

Out[∘]=

Calculation Progress

## Export log – likelihood (ϕ) values (optional)

In[•]:=
```
filenameout = SystemDialogInput["FileSave"];
Export[filenameout, phiTable, "CSV", "TextDelimiters" → ""]
```

Out[•]=
```
/Users/nm/Desktop/Lupus II/Lateral/EFourier Data &
  Results/Naïve Bayes Results/Log-Likelihood Ratio Test (BS).tif
```

## Export log – likelihood test result (optional)

```
filenameout = SystemDialogInput["FileSave"];
Export[filenameout, probPlot, "TIFF", ImageResolution → 150]
```

```
/Users/nm/Desktop/Lupus II/Dorsal/ML (PCA Scores
  - Random Forest) Results/Log Likelihood Ratio (BS) Test.tif
```

## Calculate jackknifed estimate of classification function performance.

In[•]:=
```
idComp = Table[" ", {n2}, {2}];
Do[idComp[[i, 1]] = Group[[i]], {i, n2}];

Panel[Labeled[ProgressIndicator[Dynamic[k], {1, n1}], "Calculation Progress",
  Top, LabelStyle → Directive[FontSize → 12, Bold, FontFamily → "Arial"]]]

Do[x2Out = Take[x2[[k]]];
  groupOut = Take[Group[[k]]];
  x2Test = Drop[x2, {k}];
  groupTest = Drop[Group, {k}];
  classTest =
   Classify[x2Test → groupTest, Method → cf, TrainingProgressReporting → None];
  idComp[[k, 2]] = classTest[x2Out], {k, n2}];

nClasses = Length[gpNames];
jacMat = Table[0.0, {nClasses}, {nClasses}];
Do[
  Do[
   If[idComp[[i, 1]] == gpNames[[j]] && idComp[[i, 2]] == idComp[[i, 1]],
    jacMat[[j, j]] = jacMat[[j, j]] + 1];
   If[idComp[[i, 1]] == gpNames[[j]] && idComp[[i, 2]] ≠ idComp[[i, 1]],
    colPointer = 0;
    Do[If[idComp[[i, 2]] == gpNames[[j2]], colPointer = j2], {j2, nClasses}];
    jacMat[[j, colPointer]] = jacMat[[j, colPointer]] + 1],
   {i, n2}], {j, nClasses}];

jacMatOut = Table[" ", {nGroups + 4}, {nGroups + 4}];
jacMatOut[[1, 1]] = "Groups";
Do[jacMatOut[[i + 1, 1]] = gpNames[[i]];
 jacMatOut[[1, i + 1]] = gpNames[[i]], {i, nGroups}]
Do[jacMatOut[[i + 1, j + 1]] = jacMat[[i, j]], {i, nGroups}, {j, nGroups}]
```

```
jacMatOut[[1, nGroups + 2]] = "No. Correct";
jacMatOut[[1, nGroups + 3]] = "Actual";
jacMatOut[[1, nGroups + 4]] = "Percent Correct";
jacMatOut[[nGroups + 2, 1]] = "No. Correct";
jacMatOut[[nGroups + 3, 1]] = "Predicted";
jacMatOut[[nGroups + 4, 1]] = "Predicted Correct";
Do[jacMatOut[[i + 1, nGroups + 2]] = jacMat[[i, i]], {i, nGroups}]
Do[jacMatOut[[i + 1, nGroups + 3]] = Total[jacMat[[i]]], {i, nGroups}]
Do[
 If[jacMatOut[[i + 1, nGroups + 3]] == 0,
  jacMatOut[[i + 1, nGroups + 4]] = PaddedForm[0.0, {4, 2}],
  jacMatOut[[i + 1, nGroups + 4]] = PaddedForm[
    (jacMatOut[[i + 1, nGroups + 2]] / jacMatOut[[i + 1, nGroups + 3]]) * 100.0,
    {4, 2}]], {i, nGroups}]
Do[jacMatOut[[nGroups + 2, i + 1]] = jacMat[[i, i]], {i, nGroups}]
Do[jacMatOut[[nGroups + 3, i + 1]] = Total[jacMat[[All, i]]], {i, nGroups}]
Do[
 If[jacMatOut[[nGroups + 3, i + 1]] == 0,
  jacMatOut[[nGroups + 4, i + 1]] = PaddedForm[0.00, {4, 2}],
  jacMatOut[[nGroups + 4, i + 1]] = PaddedForm[
    (jacMatOut[[nGroups + 2, i + 1]] / jacMatOut[[nGroups + 3, i + 1]]) * 100.0,
    {4, 2}]], {i, nGroups}]
jacMatOut[[nGroups + 2, nGroups + 2]] = Total[Diagonal[jacMat]];
jacMatOut[[nGroups + 2, nGroups + 3]] =
  Sum[Total[jacMat[[All, i]]], {i, nGroups}];
jacMatOut[[nGroups + 3, nGroups + 2]] = Sum[Total[jacMat[[i, All]]], {i, nGroups}];
If[jacMatOut[[nGroups + 3, nGroups + 2]] == 0,
  jacMatOut[[nGroups + 4, nGroups + 2]] = PaddedForm[0.0, {4, 2}],
  jacMatOut[[nGroups + 4, nGroups + 2]] =
   PaddedForm[(jacMatOut[[nGroups + 2, nGroups + 2]] /
        jacMatOut[[nGroups + 3, nGroups + 2]]) * 100.0, {4, 2}]];
If[jacMatOut[[nGroups + 2, nGroups + 3]] == 0,
  jacMatOut[[nGroups + 2, nGroups + 4]] = PaddedForm[0.0, {4, 2}],
  jacMatOut[[nGroups + 2, nGroups + 4]] =
   PaddedForm[(jacMatOut[[nGroups + 2, nGroups + 2]] /
        jacMatOut[[nGroups + 2, nGroups + 3]]) * 100.0, {4, 2}]];

Labeled[Grid[jacMatOut,
  BaseStyle → (FontFamily → "Arial"), Alignment → {{Right}, {Center}},
  Dividers → {{2 → True, -4 → True}, {2 → True, -4 → True}}, Frame → True,
  BaseStyle → Directive[FontSize → 12, FontFamily → "Arial"]],
 "Jacknifed Confusion Matrix", Top,
 LabelStyle → Directive[FontSize → 14, Bold, FontFamily → "Arial"]]
```

Out[∘]= **Calculation Progress**

Export jackknifed confusion matrix (optional)

In[∘]:=
```
outTab = jacMatOut;
Do[
 If[jacMatOut[[i + 1, nGroups + 3]] == 0, outTab[[i + 1, nGroups + 4]] = 0.0,
  outTab[[i + 1, nGroups + 4]] =
   (jacMatOut[[i + 1, nGroups + 2]] / jacMatOut[[i + 1, nGroups + 3]]) * 100.0];
 If[jacMatOut[[nGroups + 3, i + 1]] == 0, outTab[[nGroups + 4, i + 1]] = 0.0,
  outTab[[nGroups + 4, i + 1]] =
   (jacMatOut[[nGroups + 2, i + 1]] / jacMatOut[[nGroups + 3, i + 1]]) *
    100.0], {i, nGroups}]
If[jacMatOut[[nGroups + 3, nGroups + 2]] == 0,
  outTab[[nGroups + 4, nGroups + 2]] = 0.0,
  outTab[[nGroups + 4, nGroups + 2]] = (jacMatOut[[nGroups + 2, nGroups + 2]] /
      jacMatOut[[nGroups + 3, nGroups + 2]]) * 100.0];
If[jacMatOut[[nGroups + 2, nGroups + 3]] == 0,
  outTab[[nGroups + 2, nGroups + 4]] = 0.0,
  outTab[[nGroups + 2, nGroups + 4]] = (jacMatOut[[nGroups + 2, nGroups + 2]] /
      jacMatOut[[nGroups + 2, nGroups + 3]]) * 100.0];

filenameout = SystemDialogInput["FileSave"];
Export[filenameout, outTab, "CSV", "TextDelimiters" → ""]
```

Out[∘]=
```
/Users/nm/Desktop/Lupus II/Dorsal/EFourier Data
  & Results/Nai¨ve Bayes Results/Confusion Matrix (Jack).csv
```

Read – in test data set.

```
filenamein = SystemDialogInput["FileOpen"];
sourceDirectory = DirectoryName[filenamein];
SetDirectory[sourceDirectory];
dataFileList = Sort[Map[ToString, FileNames["*.tif"]]];
{kgTest} = Dimensions[dataFileList];

x2 = Table[" ", {kgTest}];
Do[xName = StringJoin[sourceDirectory, dataFileList[[k]]];
 x2[[k]] = Import[xName, "TIFF"], {k, kgTest}]
objNamesTest = dataFileList;
Do[objNamesTest[[k]] = StringDrop[objNamesTest[[k]], -4], {k, kgTest}]

sourceDirectory
Print["No. of Test Set Images: ", kgTest]
```

Calculate test set confusion matrix.

```
cmat = ClassifierMeasurements[class, x2 → testGroup, "ConfusionMatrix"];
cmat = Take[cmat, All, nTestGroups];

cmatOut = Table[" ", {nTestGroups + 4}, {nTestGroups + 4}];
cmatOut[[1, 1]] = "Groups";
Do[cmatOut[[i + 1, 1]] = testGpNames[[i]];
 cmatOut[[1, i + 1]] = testGpNames[[i]], {i, nTestGroups}]
Do[cmatOut[[i + 1, j + 1]] = cmat[[i, j]], {i, nTestGroups}, {j, nTestGroups}]
cmatOut[[1, nTestGroups + 2]] = "No. Correct";
cmatOut[[1, nTestGroups + 3]] = "Actual";
cmatOut[[1, nTestGroups + 4]] = "Percent Correct";
cmatOut[[nTestGroups + 2, 1]] = "No. Correct";
cmatOut[[nTestGroups + 3, 1]] = "Predicted";
cmatOut[[nTestGroups + 4, 1]] = "Predicted Correct";
Do[cmatOut[[i + 1, nTestGroups + 2]] = cmat[[i, i]], {i, nTestGroups}]
Do[cmatOut[[i + 1, nTestGroups + 3]] = Total[cmat[[i]]], {i, nTestGroups}]
Do[
 If[cmatOut[[i + 1, nTestGroups + 3]] == 0,
  cmatOut[[i + 1, nTestGroups + 4]] = PaddedForm[0.0, {4, 2}],
  cmatOut[[i + 1, nTestGroups + 4]] = PaddedForm[
    (cmatOut[[i + 1, nTestGroups + 2]] / cmatOut[[i + 1, nTestGroups + 3]]) * 100.0,
    {4, 2}]], {i, nTestGroups}]
Do[cmatOut[[nTestGroups + 2, i + 1]] = cmat[[i, i]], {i, nTestGroups}]
Do[cmatOut[[nTestGroups + 3, i + 1]] = Total[cmat[[All, i]]], {i, nTestGroups}]
Do[
 If[cmatOut[[nTestGroups + 3, i + 1]] == 0,
  cmatOut[[nTestGroups + 4, i + 1]] = PaddedForm[0.0, {4, 2}],
  cmatOut[[nTestGroups + 4, i + 1]] = PaddedForm[
    (cmatOut[[nTestGroups + 2, i + 1]] / cmatOut[[nTestGroups + 3, i + 1]]) * 100.0,
    {4, 2}]], {i, nTestGroups}]
cmatOut[[nTestGroups + 2, nTestGroups + 2]] = Total[Diagonal[cmat]];
cmatOut[[nTestGroups + 2, nTestGroups + 3]] =
  Sum[Total[cmat[[All, i]]], {i, nTestGroups}];
cmatOut[[nTestGroups + 3, nTestGroups + 2]] =
  Sum[Total[cmat[[i, All]]], {i, nTestGroups}];
If[cmatOut[[nTestGroups + 3, nTestGroups + 2]] == 0,
  cmatOut[[nTestGroups + 4, nTestGroups + 2]] = PaddedForm[0.0, {4, 2}],
  cmatOut[[nTestGroups + 4, nTestGroups + 2]] =
   PaddedForm[(cmatOut[[nTestGroups + 2, nTestGroups + 2]] /
       cmatOut[[nTestGroups + 3, nTestGroups + 2]]) * 100.0, {4, 2}]];
If[cmatOut[[nTestGroups + 2, nTestGroups + 3]] == 0,
  cmatOut[[nTestGroups + 2, nTestGroups + 4]] = PaddedForm[0.0, {4, 2}],
  cmatOut[[nTestGroups + 2, nTestGroups + 4]] =
   PaddedForm[(cmatOut[[nTestGroups + 2, nTestGroups + 2]] /
       cmatOut[[nTestGroups + 2, nTestGroups + 3]]) * 100.0, {4, 2}]];
```

```
Labeled[Grid[cmatOut,
   BaseStyle → (FontFamily → "Arial"), Alignment → {{Right}, {Center}},
   Dividers → {{2 → True, -4 → True}, {2 → True, -4 → True}}, Frame → True,
   BaseStyle → Directive[FontSize → 12, FontFamily → "Arial"]],
  "Confusion Matrix: Test Set", Top,
  LabelStyle → Directive[FontSize → 16, Bold, FontFamily → "Arial"]]
```

Export test set confusion matrix (optional).

```
outTab = cmatOut;
Do[
  outTab[[i + 1, nGroups + 4]] =
   (cmatOut[[i + 1, nGroups + 2]] / cmatOut[[i + 1, nGroups + 3]]) * 100.0;
  outTab[[nGroups + 4, i + 1]] =
   (cmatOut[[nGroups + 2, i + 1]] / cmatOut[[nGroups + 3, i + 1]]) * 100.0, {i, nGroups}]
outTab[[nGroups + 4, nGroups + 2]] =
   (cmatOut[[nGroups + 2, nGroups + 2]] / cmatOut[[nGroups + 3, nGroups + 2]]) * 100.0;
outTab[[nGroups + 2, nGroups + 4]] =
   (cmatOut[[nGroups + 2, nGroups + 2]] / cmatOut[[nGroups + 2, nGroups + 3]]) * 100.0;
filenameout = SystemDialogInput["FileSave"];
Export[filenameout, outTab, "CSV"]
```

Read – in unknown data (optional).

```
filenamein = SystemDialogInput["FileOpen"];
sourceDirectory = DirectoryName[filenamein];
SetDirectory[sourceDirectory];
dataFileList = Sort[Map[ToString, FileNames["*.tif"]]];
{kgUnk} = Dimensions[dataFileList];

x3 = Table[" ", {kgUnk}];
Do[xName = StringJoin[sourceDirectory, dataFileList[[k]]];
  x3[[k]] = Import[xName, "TIFF"], {k, kgUnk}]
objNamesUnk = dataFileList;
Do[objNamesUnk[[k]] = StringDrop[objNamesUnk[[k]], -4], {k, kgUnk}]

sourceDirectory
Print["No. of Unknown Images: ", kgUnk]
```

Identify unknown images (optional).

```
IDs = Table[" ", {kgUnk}, {3}];
IDout = Table[" ", {kgUnk + 1}, {3}];
IDoutTab = IDout;

Do[
 IDs[[k, 1]] = objNamesUnk[[k]];
 IDs[[k, 2]] = class[x3[[k]], "Decision"];
 IDs[[k, 3]] = class[x3[[k]], "TopProbabilities" → 1], {k, kgUnk}]
IDs = Partition[Flatten[IDs], 3];
Do[IDs[[k, 3]] = Last[IDs[[k, 3]]], {k, kgUnk}];

IDout[[1, 1]] = Style["Image Name", Bold];
IDout[[1, 2]] = Style["Identification", Bold];
IDout[[1, 3]] = Style["Probability", Bold];
Do[IDout[[k + 1, j]] = PaddedForm[IDs[[k, j]], {4, 3}], {j, 3}, {k, kgUnk}]

Labeled[Grid[IDout, BaseStyle → (FontFamily → "Arial"),
   Alignment → {{Left, Center, Center}}, Frame → True,
   Dividers → {{True, True, True}, {True, True}}], "Identification Table", Top,
 LabelStyle → Directive[Black, Bold, FontSize → 16, FontFamily → "Arial"]]
```

Export identification results (optional).

```
IDoutTab[[1, 1]] = "Image Name";
IDoutTab[[1, 2]] = "Identification";
IDoutTab[[1, 3]] = "Probability";
Do[IDoutTab[[k + 1]] = IDs[[k]], {k, kgUnk}];
filenameout = SystemDialogInput["FileSave"];
Export[filenameout, IDoutTab, "CSV"]
```
