# Supplementary material for: Machine-learning strategies for testing patterns of morphological variation in small samples: sexual dimorphism in gray wolf (Canis lupus) crania
Source: BMC Biol. 2020 Sep 3;18:113. doi: 10.1186/s12915-020-00832-1 (PMC7470621; doi:10.1186/s12915-020-00832-1)
Supplement: Supplementary file 5 — Additional file 5. An archive of all code listings for all data procession/analysis software employed in this investigation. [file 12915_2020_832_MOESM5_ESM.zip › SI File 5/Elliptical Fourier Analysis (vers. 1.3).pdf]

Ellipical Fourier Analysis

This program accepts a set of closed outlines defined by semilandmark

dataset in standard format and performs an elliptical Fourier analysis on each outline in the set.

Author : N. MacLeod

Version : 1.3

Date : 30 April 2016

Initialize libraries.

In[∘]:=
```
<< ComputationalGeometry`
```

Enter input control parameters

In[∘]:=
```
Panel[Labeled[Column[{Panel[PopupMenu[Dynamic[fileType],
      {1 → ".tps-outline datafile", 2 → ".dat datafile"}]],
    Row[{Panel[Grid[{{Style["Enter no. of objects", Bold], SpanFromLeft},
        {InputField[Dynamic[kg], FieldSize → 5], Dynamic[kg];}}]],
      Panel[Grid[{{Style["Enter no. of variables", Bold], SpanFromLeft},
        {InputField[Dynamic[m], FieldSize → 5], Dynamic[m];}}]]}],
    Labeled[
     Row[{Panel[Grid[{{Style["Enter no. of header rows", Bold], SpanFromLeft},
          {InputField[Dynamic[h1], FieldSize → 5], Dynamic[h1];}}]],
       Panel[Labeled[RadioButtonBar[Dynamic[s1], {1 → "Yes", 2 → "No"},
          LabelStyle → (FontFamily → "Arial")], "Is a scale present?",
         Top, LabelStyle → Directive[Bold]]]}], ".tps files only",
     Top, LabelStyle → Directive[FontSize → 12, FontFamily → "Arial"]]},
   Center], "Datafile Input Control Parameters", Top,
  LabelStyle → Directive[FontSize → 14, Bold, FontFamily → "Arial"]]
kg = 12; m = 2; h1 = 3; s1 = 2; fileType = 1;
```

Out[∘]=

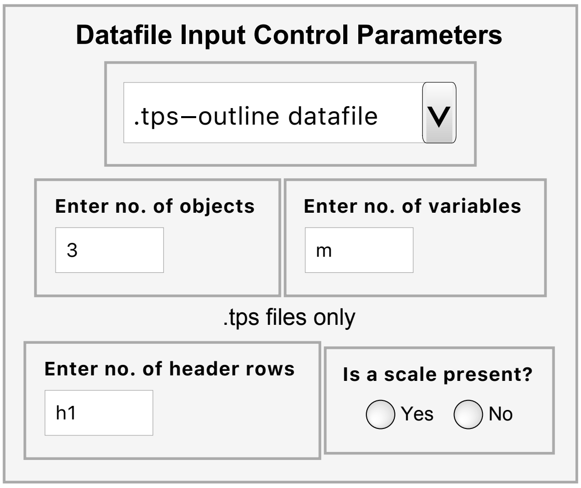

Read in data file & partition into datasets.

```
In[•]:= filenamein = SystemDialogInput["FileOpen"]
x1 = Import[filenamein, "Table"];

If[fileType == 1, {ndf} = Dimensions[x1], {ndf, m} = Dimensions[x1]];
ntot = ndf / kg;
If[fileType == 1, n = ntot - (h1 + 1), n = ntot - 1];

x1Coords = Table[0, {kg}, {n}, {m}];
objNames = Table[0, {kg}];

If[fileType == 1,

 Do[
  knt = k - 1;
  iup = (ntot * knt) + 1;
  ilow = ntot * (knt + 1);
  tmp1 = Take[x1, {iup, ilow}, All];
  tmp2 = Drop[tmp1, h1];
  tmp3 = Take[tmp2, n];
  tmp4 = Take[tmp2, -1];

  name =
   StringDrop[StringDrop[StringJoin[tmp4[[1, 1]], " ", tmp4[[1, 2]]], 6], -5];

  x1Coords[[k]] = tmp3;
  objNames[[k]] = name, {k, kg}]]

If[fileType == 2,
 Do[
  ilow = (n + 1) * k;
  iup = ilow - (n);
  b = Take[x1, iup];
  objNames[[k]] = b[[iup]];
  w1 = ToString[objNames[[k, 1]]];
  w2 = ToString[objNames[[k, 2]]];
  objNames[[k]] = StringJoin[w1, " ", w2];
  x1Coords[[k]] = Take[x1, {iup + 1, ilow}, All], {k, kg}]]
```

Show raw outlines
[Note : you need to run this script the first time through so it can pick up the necessary variable values]

In[∘]:=
```
Manipulate[Show[oPlot = Labeled[ListPlot[x1Coords[[specimenNum]], Joined → True,
      AspectRatio → Automatic, Axes → False], objNames[[specimenNum]], Top,
    LabelStyle → Directive[FontSize → 18, Italic, FontFamily → "Arial"]],
  ImageSize → 400], {{specimenNum, 1, "Specimen number:"},
  1, kg, 1, Appearance → "Open"}]
```

Out[∘]=

Specimen number:

1

M0001

```
ListPlot[x1Coords⟦1⟧, Joined → True,
 AspectRatio → Automatic, Axes → False]
```

Part: Part specification x1Coords⟦1⟧ is longer than depth of object.

ListPlot: x1Coords⟦1⟧ is not a list of numbers or pairs of numbers.

ListPlot: x1Coords⟦1⟧ is not a list of numbers or pairs of numbers.

ListPlot: x1Coords⟦1⟧ is not a list of numbers or pairs of numbers.

General: Further output of ListPlot::lpn will be suppressed during this calculation.

Export plot (optional).

```
filenameout = SystemDialogInput["FileSave"];
Export[filenameout, oPlot, "TIFF"]
```

Set number of harmonics to use in the Fourier series
[Note : default is set to the Nyquist frequency. You'll probably want to step that down.]

In[∘]:=
```
Panel[Grid[{{Style["Enter no. of harmonics to extract",
      Directive[FontSize → 10, Bold, FontFamily → "Arial"]], SpanFromLeft},
    {InputField[Dynamic[fNum], FieldSize → 12], Dynamic[fNum];}}]]
fNum = IntegerPart[((ndf / kg) - h1 - 1) / 2];
```

Out[∘]=

Enter no. of harmonics to extract

fNum

Perform elliptical Fourier decomposition.
[Note : this may take a while. Please be patient.]

In[∘]:=
```
efCoefs = Table[" ", {kg}, {fNum}, {4}];
efCoefsNorm = Table[" ", {kg}, {fNum}, {4}];

Panel[Labeled[ProgressIndicator[Dynamic[sNum], {1, kg}], "Calculation Progress",
  Top, LabelStyle → Directive[FontSize → 12, Bold, FontFamily → "Arial"]]]
```

```
Do[

 currentOL = x1Coords[[sNum]];
 distList = Table[ EuclideanDistance[currentOL[[i]], currentOL[[i - 1]]],
   {i, 2, Length[currentOL]}];
 k = Length[currentOL] - 1;

 Do[tp = Total[Take[distList, p]], {p, k}];
 t0 = 0;
 Do[Δtp = distList[[p]], {p, k}];
 Do[Δxp = currentOL[[p + 1, 1]] - currentOL[[p, 1]], {p, Length[currentOL] - 1}];
 Do[Δyp = currentOL[[p + 1, 2]] - currentOL[[p, 2]], {p, Length[currentOL] - 1}];
 T = tk;

 Do[

  An = T / (2 n^2 π^2) Sum[Δxp / Δtp (Cos[2 π n tp / T] - Cos[2 π n tp-1 / T]), {p=1, k}];

  Bn = T / (2 n^2 π^2) Sum[Δxp / Δtp (Sin[2 π n tp / T] - Sin[2 π n tp-1 / T]), {p=1, k}];

  Cn = T / (2 n^2 π^2) Sum[Δyp / Δtp (Cos[2 π n tp / T] - Cos[2 π n tp-1 / T]), {p=1, k}];

  Dn = T / (2 n^2 π^2) Sum[Δyp / Δtp (Sin[2 π n tp / T] - Sin[2 π n tp-1 / T]), {p=1, k}];

  , {n, fNum}
 ];

 θ = .5 ArcTan[(2 A1 B1 + 2 C1 D1) / (A1^2 + C1^2 - B1^2 - D1^2)];

 {aStar, cStar} = ( A1  B1
                    C1  D1 ).{Cos[θ], Sin[θ]};

 eStar = Sqrt[aStar^2 + cStar^2];
 γ = ArcTan[cStar / aStar];

 Do[
  ( an  bn      = 1 / eStar (  Cos[γ]   Sin[γ]  . ( An  Bn  . ( Cos[n θ]  -Sin[n θ]
    cn  dn )                  -Sin[γ]   Cos[γ] )    Cn  Dn )    Sin[n θ]   Cos[n θ] )
  , {n, fNum}
 ];

 Do[efCoefsNorm[[sNum, i, 1]] = ai, {i, fNum}];
```

```
 Do[efCoefsNorm[[sNum, i, 2]] = b_i, {i, fNum}];
 Do[efCoefsNorm[[sNum, i, 3]] = c_i, {i, fNum}];
 Do[efCoefsNorm[[sNum, i, 4]] = d_i, {i, fNum}], {sNum, kg}]
```

```
Do[
 Do[efCoefs[[sNum, i, 1]] = N[A_i], {i, fNum}];
 Do[efCoefs[[sNum, i, 2]] = N[B_i], {i, fNum}];
 Do[efCoefs[[sNum, i, 3]] = N[C_i], {i, fNum}];
 Do[efCoefs[[sNum, i, 4]] = N[D_i], {i, fNum}], {sNum, kg}]
```

Out[∘]=

Calculation Progress

Show barcharts of normalized EFA coefficients (optional).

Select specimen
[Note : you'll need to run this script to pick up the specimen names.]

In[∘]:=

```
Panel[Labeled[PopupMenu[Dynamic[specName], objNames],
  "Select specimen to plot coefficients for.", Top,
  LabelStyle → Directive[FontSize → 12, Bold, FontFamily → "Arial"]]]
specName = objNames[[1]];
```

Out[∘]=

Select specimen to plot coefficients for.

Plot coefficients.

```
In[•]:= Do[If[specName == objNames[[k]], sNum = k], {k, kg}]

labVals = Table[i, {i, fNum}];
p1 =
  Labeled[BarChart[efCoefsNorm[[sNum, All, 1]], Frame → True, ChartStyle → {Red},
    LabelStyle → Directive[FontSize → 12, FontFamily → "Arial"],
    ImageSize → 300], "A-Coefficients", Top,
   LabelStyle → Directive[FontSize → 12, FontFamily → "Arial"]];
p2 = Labeled[BarChart[efCoefsNorm[[sNum, All, 2]], Frame → True, ChartStyle →
     {Green}, LabelStyle → Directive[FontSize → 12, FontFamily → "Arial"],
    ImageSize → 300], "B-Coefficients", Top,
   LabelStyle → Directive[FontSize → 12, FontFamily → "Arial"]];
p3 = Labeled[BarChart[efCoefsNorm[[sNum, All, 3]], Frame → True, ChartStyle →
     {Blue}, LabelStyle → Directive[FontSize → 12, FontFamily → "Arial"],
    ImageSize → 300], "C-Coefficients", Top,
   LabelStyle → Directive[FontSize → 12, FontFamily → "Arial"]];
p4 = Labeled[BarChart[efCoefsNorm[[sNum, All, 4]], Frame → True, ChartStyle →
     {Yellow}, LabelStyle → Directive[FontSize → 12, FontFamily → "Arial"],
    ImageSize → 300], "D-Coefficients", Top,
   LabelStyle → Directive[FontSize → 12, FontFamily → "Arial"]];
plot1 = Labeled[Labeled[Labeled[Grid[{{p1, p2}, {p3, p4}}, BaselinePosition → Top,
     Alignment → Top], "Normalized Fourier Coefficients", Top,
    LabelStyle → Directive[FontSize → 16, FontFamily → "Arial"]],
   " ", Top], objNames[[sNum]], Top,
  LabelStyle → Directive[FontSize → 20, Italic, FontFamily → "Arial"]]
```

Export plot (optional)

```
filenameout = SystemDialogInput["FileSave"];
Export[filenameout, plot1, "TIFF"]
```

Reconstruct outline using Fourier harmonics (optional).

Set control parameters
[Note : You'll need to run this script to pick up the correct names.]

In[•]:=
```
Panel[Labeled[Column[{Panel[Labeled[PopupMenu[Dynamic[specName], objNames],
      "Select specimen to plot coefficients for.", Top,
      LabelStyle → Directive[FontSize → 12, FontFamily → "Arial"]]],
    Panel[ Labeled[InputField[Dynamic[fMax], FieldSize → 10],
      "Enter no. of harmonics to use in reconstruction.", Top,
      LabelStyle → Directive[FontSize → 12, FontFamily → "Arial"]]]}, Center],
  "Outline Reconstruction Parameters", Top, LabelStyle →
   Directive[FontSize → 14, Bold, FontFamily → "Arial"]]]
specName = objNames[[1]]; fMax = fNum;
```

Out[•]=

Outline Reconstruction Parameters

Select specimen to plot coefficients for.

Enter no. of harmonics to use in reconstruction.

fMax

Reconstruct outline s

In[•]:=
```
Do[If[specName == objNames[[k]], specimenNum = k], {k, kg}]
Do[
 aCoef_i = efCoefsNorm[[specimenNum, i, 1]];
 bCoef_i = efCoefsNorm[[specimenNum, i, 2]];
 cCoef_i = efCoefsNorm[[specimenNum, i, 3]];
 dCoef_i = efCoefsNorm[[specimenNum, i, 4]], {i, fMax}]

Labeled[Manipulate[Show[
   xFunc = Sum_{n=1}^{fNum} (aCoef_n Cos[n t] + bCoef_n Sin[n t]);
   yFunc = Sum_{n=1}^{fNum} (cCoef_n Cos[n t] + dCoef_n Sin[n t]);
   paramPlot = ParametricPlot[{xFunc, yFunc}, {t, 0, 2 π},
     AspectRatio → Automatic, Mesh → None, Axes → False], ImageSize → 400,
   AspectRatio → Automatic], {{fNum, 1, "Number of coefficients"},
   1, fMax, 1, Appearance → "Open"}], objNames[[specimenNum]],
 Top, LabelStyle → Directive[FontSize → 18, Italic, FontFamily → "Arial"]]
```

Export plot (optional)

```
filenameout = SystemDialogInput["FileSave"];
Export[filenameout, paramPlot, "TIFF"]
```

Export EFA coefficients for secondary plotting and/or analysis

In[∘]:=
```
outFile = Table[" ", {kg + 1}, {(fNum * 4) - 2}];
outFile[[1, 1]] = "Specimen";
outFile[[1, 2]] = "Group";
Do[outFile[[i + 1, 1]] = objNames[[i]], {i, kg}];
Do[outFile[[i + 1, 2]] = 1, {i, kg}];
Do[outFile[[1, j + 1]] = StringJoin["a", ToString[j]], {j, 2, fNum}];
Do[outFile[[k + 1, j + 1]] = efCoefsNorm[[k, j, 1]], {k, kg}, {j, 2, fNum}];
Do[outFile[[1, j + (fNum - 1) + 1]] = StringJoin["b", ToString[j]], {j, 2, fNum}];
Do[outFile[[k + 1, j + (fNum - 1) + 1]] = efCoefsNorm[[k, j, 2]], {k, kg}, {j, 2, fNum}]
Do[outFile[[1, j + (((fNum - 1) + 1) * 2) - 1]] =
    StringJoin["c", ToString[j]], {j, 2, fNum}];
Do[outFile[[k + 1, j + (((fNum - 1) + 1) * 2) - 1]] = efCoefsNorm[[k, j, 3]],
   {k, kg}, {j, 2, fNum}];
Do[outFile[[1, j + (((fNum - 1) + 1) * 3) - 2]] = StringJoin["d", ToString[j]],
   {j, 2, fNum}];
Do[outFile[[k + 1, j + (((fNum - 1) + 1) * 3) - 2]] = efCoefsNorm[[k, j, 4]],
  {k, kg}, {j, 2, fNum}]
filenameout = SystemDialogInput["FileSave"];
Export[filenameout, outFile, "CSV"]
```
