# Supplementary material for: Machine-learning strategies for testing patterns of morphological variation in small samples: sexual dimorphism in gray wolf (Canis lupus) crania
Source: BMC Biol. 2020 Sep 3;18:113. doi: 10.1186/s12915-020-00832-1 (PMC7470621; doi:10.1186/s12915-020-00832-1)
Supplement: Supplementary file 5 — Additional file 5. An archive of all code listings for all data procession/analysis software employed in this investigation. [file 12915_2020_832_MOESM5_ESM.zip › SI File 5/Image Classification - Embedded LeNet (vers. 1.0).pdf]

Image Classification - LeNet (Embedded)

xxx

Author : N. MacLeod
Date : 28 January 2020
Version : 1.0
Reference : MacLeod & Kolska (in press)

Initialize libraries.

```
In[]:= << ComputationalGeometry`
```

Read - in training-set images.

```
In[]:= filenamein = SystemDialogInput["FileOpen"];
sourceDirectory = DirectoryName[filenamein]
SetDirectory[sourceDirectory];
dataFileList = Sort[Map[ToString, FileNames["*.tif"]]];
{kgTrain} = Dimensions[dataFileList];

x1 = Table[" ", {kgTrain}];
Do[xName = StringJoin[sourceDirectory, dataFileList[[i]]];
 x1[[i]] = Import[xName, "TIFF"], {i, kgTrain}]
objNamesTrain = dataFileList;
Do[objNamesTrain[[i]] = StringDrop[objNamesTrain[[i]], -4], {i, kgTrain}]
Print["No. of objects: " , kgTrain]
```

Display original images (optional).

Select user - specified plot options.

In[∘]:=
```
Panel[Labeled[Column[{Row[
      {Panel[Labeled[RadioButtonBar[Dynamic[sampOpt], {1 → "All", 2 → "Sample"},
          LabelStyle → (FontFamily → "Arial")], "Select image display option",
         Top, LabelStyle → Directive[FontSize → 12, FontFamily → "Arial"]]], " ",
       Panel[Labeled[InputField[Dynamic[kgImg], FieldSize → 5],
         "Enter no. of images to display.", Top,
         LabelStyle → Directive[FontSize → 12, Plain, FontFamily → "Arial"]]]}],
     Row[{Panel[Labeled[InputField[Dynamic[rowNum], FieldSize → 5],
         "Enter number of plots per grid row.", Top, LabelStyle →
          Directive[FontSize → 12, Plain, FontFamily → "Arial"]]], " ",
       Panel[Labeled[InputField[Dynamic[imgSize], FieldSize → 5],
         "Enter image size control parameter.", Top,
         LabelStyle → Directive[FontSize → 12, Plain, FontFamily → "Arial"]]]}],
     Panel[Labeled[InputField[Dynamic[grdSize], FieldSize → 5],
       "Enter grid size control parameter.", Top,
       LabelStyle → Directive[FontSize → 12, Plain, FontFamily → "Arial"]]]},
    Center], "Processed Image Display Options", Top,
   LabelStyle → Directive[FontSize → 16, Bold, FontFamily → "Arial"]]]
sampOpt = 1; kgImg = kgTrain; rowNum = 4; imgSize = 100; grdSize = 700;
```

Out[∘]=

Processed Image Display Options

Select image display option

◉ All ○ Sample

Enter no. of images to display.

kgImg

Enter number of plots per grid row.

rowNum

Enter image size control parameter.

imgSize

Enter grid size control parameter.

grdSize

Display original images.

In[•]:=
```
If[sampOpt == 1,
  kgDisp = kgTrain;
  gridTitle = "Original Images (All)";
  imgDisp = Table[i, {i, kgTrain}],
  kgDisp = kgImg;
  gridTitle = "Original Images (Random Sample)";
  imgDisp = Sort[RandomSample[Range[kgTrain], kgDisp]]];

t1 = N[kgDisp / rowNum];
t2 = IntegerPart[N[kgDisp / rowNum]];
If[t1 - t2 > 0., kg2 = (t2 + 1) * rowNum, kg2 = t2 * rowNum];
imgTable = Table[" ", {kg2}];
Do[imgTable[[k]] = Labeled[ImageResize[x1[[imgDisp[[k]]]], imgSize],
   objNamesTrain[[imgDisp[[k]]]], Top, LabelStyle →
    Directive[FontSize → 12, Italic, FontFamily → "Arial"]], {k, kgDisp}]
imgPlt1 = Labeled[GraphicsGrid[Partition[imgTable, rowNum],
   Frame → All, ImageSize → grdSize], gridTitle, Top,
  LabelStyle → Directive[FontSize → 18, Bold, FontFamily → "Arial"]]
```

Read in training-set group classification list & assemble training-set data file

In[•]:=
```
filenamein = SystemDialogInput["FileOpen"];
groupTrain = Import[filenamein, "CSV"];
filenamein

groupTrain = Flatten[groupTrain];
{nTrain} = Dimensions[groupTrain];
gpNamesTrain = Union[groupTrain];
nGps = Length[Union[groupTrain]];
mTrain = Length[gpNamesTrain];

trainData = Table[" ", {nTrain}];
Do[
 Do[
  If[groupTrain[[i]] == gpNamesTrain[[j]],
   trainData[[i]] = x1[[i]] → gpNamesTrain[[j]]], {j, nGps}], {i, nTrain}]
Print["No. of labels: " , nTrain]
```

Build a generic version of the LeNet CNN and train using the training-set contrasts

In[ ]:=
```
toRule[{img1_ → l1_, img2_ → l2_}] := {img1, img2} → l1 ≠ l2;
comb = (kgTrain * kgTrain) * 0.8;
trainingData = Table[toRule@RandomSample[trainData, 2], comb];

lenet = NetChain[{
    ConvolutionLayer[10, 4], Ramp, PoolingLayer[2, 2],
    ConvolutionLayer[20, 4], Ramp, PoolingLayer[2, 2],
    FlattenLayer[], 2},
   "Input" → NetEncoder[{"Image", {28, 28}, "Grayscale"}]];
net = NetPairEmbeddingOperator[lenet];
results = NetTrain[net, trainingData,
  All, TrainingProgressReporting → "ProgressIndicator"]
trained = results["TrainedNet"];
embedding = NetExtract[trained, "Net"];

groups = KeySort@GroupBy[trainData, Last → First];
points = Map[embedding /* Normal, groups];
c = Classify[points];
cm = ClassifierMeasurements[c, points];
```

Out[ ]=

NetTrain Results

summary	batches: 728, rounds: 14,<br>time: 14min, examples/s: 55.
data	training examples: 3276,<br>processed examples: 46 592,<br>skipped examples: 0
method	ADAM optimizer, batch size 64, CPU
round	loss: 6.29×10^{-7}

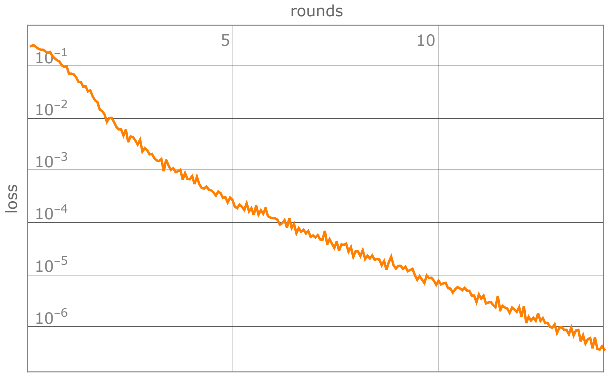

Calculate training-set confusion matrix (optional).

In[•]:=
```
confMatrix = cm["ConfusionMatrix"];
nc = Length[gpNamesTrain];
cMatrix = Table[" ", {nc + 4}, {nc + 4}];
cMatrix[[1, 1]] = Text["Groups"];
cMatrix[[1, nc + 2]] = Text[Rotate["No. Correct", 90 Degree]];
cMatrix[[1, nc + 3]] = Text[Rotate["Group Total", 90 Degree]];
cMatrix[[1, nc + 4]] = Text[Rotate["Percent Correct", 90 Degree]];
Do[cMatrix[[i + 1, 1]] = Text[gpNamesTrain[[i]]], {i, nc}];
Do[cMatrix[[1, i + 1]] = Text[Rotate[gpNamesTrain[[i]], 90 Degree]], {i, nc}]
Do[
  If[confMatrix[[i, j]] == 0, cMatrix[[i + 1, j + 1]] = Text["-"],
   cMatrix[[i + 1, j + 1]] = Text[confMatrix[[i, j]]]], {j, nc}, {i, nc}];
cMatrix[[nc + 2, 1]] = Text["No. Correct"];
cMatrix[[nc + 3, 1]] = Text["Group Total"];
cMatrix[[nc + 4, 1]] = Text["Percent Correct"];
Do[cMatrix[[i + 1, nc + 2]] = Text[confMatrix[[i, i]]], {i, nc}];
cMatrix[[nc + 2, nc + 2]] = Text[Total[Diagonal[confMatrix]]];
cMatrix[[nc + 2, nc + 3]] = Text[Total[Total[confMatrix]]];
cMatrix[[nc + 2, nc + 4]] = Text[PaddedForm[
    N[Total[Diagonal[confMatrix]] / Total[Total[confMatrix]]] * 100.0, {4, 1}]];
Do[cMatrix[[i + 1, nc + 3]] = Text[Total[confMatrix[[i, All]]]], {i, nc}]
Do[
 If[
  confMatrix[[i, i]] == 0,
  cMatrix[[i + 1, nc + 4]] = Text["0.0"], cMatrix[[i + 1, nc + 4]] = Text[
    PaddedForm[N[Take[confMatrix[[i, i]]] / Total[Take[confMatrix[[i, All]]]]] *
      100.0, {4, 1}]]], {i, nc}]
Do[cMatrix[[nc + 2, i + 1]] = Text[confMatrix[[i, i]]], {i, nc}]
Do[cMatrix[[nc + 3, i + 1]] = Text[Total[confMatrix[[All, i]]]], {i, nc}]
cMatrix[[nc + 3, nc + 2]] = Text[Total[Total[confMatrix]]];
cMatrix[[nc + 4, nc + 2]] = Text[PaddedForm[
    N[Total[Diagonal[confMatrix]] / Total[Total[confMatrix]]] * 100.0, {4, 1}]];
Do[
 If[
  confMatrix[[i, i]] == 0, cMatrix[[nc + 4, i + 1]] = Text["0.0"],
  cMatrix[[nc + 4, i + 1]] = Text[
    PaddedForm[N[Take[confMatrix[[i, i]]] / Total[Take[confMatrix[[All, i]]]]] *
      100.0, {4, 1}]]], {i, nc}]

trainConfMatrix =
 Labeled[Grid[cMatrix, Alignment → {{1 → Right, nc + 4 → Right}, {Bottom, Right}},
   Frame → True, Dividers → {{2 → True, -4 → True}, {2 → True, -4 → True}}],
  "Training-Set Confusion Matrix", Top,
  LabelStyle → Directive[FontSize → 16, Bold, FontFamily → "Arial"]]
```

Export training-set confusion matrix

In[∘]:=
```
cMatrix = Table[" ", {nc + 4}, {nc + 4}];
cMatrix[[1, 1]] = "Groups";
cMatrix[[1, nc + 2]] = "No. Correct";
cMatrix[[1, nc + 3]] = "Group Total";
cMatrix[[1, nc + 4]] = "Percent Correct";
Do[cMatrix[[i + 1, 1]] = gpNamesTrain[[i]], {i, nc}];
Do[cMatrix[[1, i + 1]] = gpNamesTrain[[i]], {i, nc}];
Do[
  If[confMatrix[[i, j]] == 0, cMatrix[[i + 1, j + 1]] = "-",
   cMatrix[[i + 1, j + 1]] = confMatrix[[i, j]]], {j, nc}, {i, nc}];
cMatrix[[nc + 2, 1]] = "No. Correct";
cMatrix[[nc + 3, 1]] = "Group Total";
cMatrix[[nc + 4, 1]] = "Percent Correct";
Do[cMatrix[[i + 1, nc + 2]] = confMatrix[[i, i]], {i, nc}];
cMatrix[[nc + 2, nc + 2]] = Total[Diagonal[confMatrix]];
cMatrix[[nc + 2, nc + 3]] = Total[Total[confMatrix]];
cMatrix[[nc + 2, nc + 4]] =
  N[Total[Diagonal[confMatrix]] / Total[Total[confMatrix]]] * 100.0;
Do[cMatrix[[i + 1, nc + 3]] = Total[confMatrix[[i, All]]], {i, nc}];
Do[
  If[
   confMatrix[[i, i]] == 0, cMatrix[[i + 1, nc + 4]] = 0.0,
   cMatrix[[i + 1, nc + 4]] = N[Take[confMatrix[[i, i]]] /
       Total[Take[confMatrix[[i, All]]]]] * 100.0], {i, nc}];
Do[cMatrix[[nc + 2, i + 1]] = confMatrix[[i, i]], {i, nc}];
Do[cMatrix[[nc + 3, i + 1]] = Total[confMatrix[[All, i]]], {i, nc}];
cMatrix[[nc + 3, nc + 2]] = Total[Total[confMatrix]];
cMatrix[[nc + 4, nc + 2]] =
  N[Total[Diagonal[confMatrix]] / Total[Total[confMatrix]]] * 100.0;
Do[
  If[
   confMatrix[[i, i]] == 0, cMatrix[[nc + 4, i + 1]] = 0.0,
   cMatrix[[nc + 4, i + 1]] =
    N[Take[confMatrix[[i, i]]] / Total[Take[confMatrix[[All, i]]]]] *
     100.0], {i, nc}];

filenameout = SystemDialogInput["FileSave"];
Export[filenameout, cMatrix, "CSV", "TextDelimiters" → ""]
```

Create 2 D feature space scatterplot .

Specify 2 D plot options.

You must run this code after you read in the data so it can pick up the proper variable names.

```
In[•]:= axisNames = Table[StringJoin["FS Axis", ToString[i]], {i, 2}];
Panel[
 Labeled[Column[{Row[{Panel[Labeled[PopupMenu[Dynamic[xAxisName], axisNames],
        "Select variable to be plotted on x-Axis.", Top, LabelStyle →
         Directive[FontSize → 12, Bold, FontFamily → "Arial"]]], "  ",
      Panel[Labeled[PopupMenu[Dynamic[yAxisName], axisNames],
        "Select variable to be plotted on y-Axis.", Top,
        LabelStyle → Directive[FontSize → 12, Bold, FontFamily → "Arial"]]]}],
    Row[{
      Panel[Labeled[PopupMenu[Dynamic[pltAspect],
         {1 → "Golden Ratio Plot", 2 → "Square Plot (equi-length axes)",
          3 → "True-Scale Plot (actual axis scales)"}],
        "Enter plot aspect ratio type.", Top, LabelStyle →
         Directive[FontSize → 12, Bold, FontFamily → "Arial"]]], "  ",
      Panel[Labeled[PopupMenu[Dynamic[lch], {1 → "Simple scatterplot",
          2 → "Scatterplot w/ convex hulls"}],
        "Show group domians?", Top, LabelStyle →
         Directive[FontSize → 12, Bold, FontFamily → "Arial"]]], "  ",
      Panel[Labeled[PopupMenu[Dynamic[ptsJoin], {1 → "No", 2 → "Yes"}],
        "Join datapoints?", Top,
        LabelStyle → Directive[FontSize → 12, Bold, FontFamily → "Arial"]]]}],
    Row[{Panel[Labeled[InputField[Dynamic[pltSize], FieldSize → 10],
        "Enter plot size value.", Top,
        LabelStyle → Directive[FontSize → 12, Bold, FontFamily → "Arial"]]],
      "  ", Panel[Labeled[InputField[Dynamic[pltPad], FieldSize → 10],
        "Enter plot margin padding value.", Top, LabelStyle →
         Directive[FontSize → 12, Bold, FontFamily → "Arial"]]], "  ",
      Panel[Labeled[InputField[Dynamic[iconSize], FieldSize → 10],
        "Enter plot icon size value.", Top, LabelStyle →
         Directive[FontSize → 12, Bold, FontFamily → "Arial"]]]}]}, Center],
  "2D Plot Options", Top, LabelStyle → Directive[FontSize → 18,
    Bold, FontFamily → "Arial"]]]
pltSize = 500; iconSize = 0.03; pltPad = 0.1; xAxisName = axisNames[[1]];
yAxisName = axisNames[[2]];
 ptsJoin = 1; dataTrans = 1;  pltAspect = 1 ; lch = 2;
```

Out[•]=

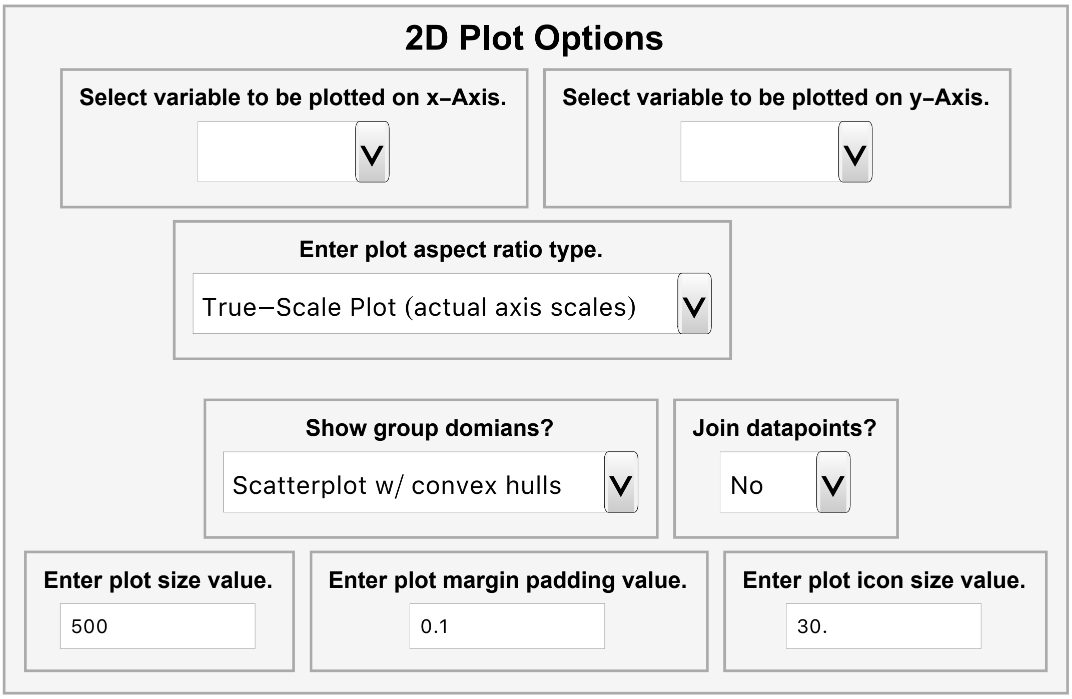

Plot trained feature space

In[•]:=
```
points = Table[0.0, {kgTrain}, {2}];
Do[points[[i]] = embedding[trainData[[i, 1]]], {i, kgTrain}];

pltTable = Table[" ", {nGps}, {3}];
Do[If[xAxisName == axisNames[[j]], axis1 = j], {j, nGps}];
Do[If[yAxisName == axisNames[[j]], axis2 = j], {j, nGps}];

groupNames = gpNamesTrain;
numGroups = Length[groupNames];
groupPosns =
  Table[Flatten[Position[groupTrain, groupNames[[i]], 1]], {i, numGroups}];
pointsT = Transpose[points];

xAxis = pointsT[[axis1]]; yAxis = pointsT[[axis2]];
lab1 = StringJoin[{"Feature Space Axis ", ToString[axis1]}];
lab2 = StringJoin[{"Feature Space Axis ", ToString[axis2]}];
maxx = Max[xAxis];
minx = Min[xAxis];
maxy = Max[yAxis];
miny = Min[yAxis];

If[pltAspect == 1 || pltAspect == 3,
  xPlotLow = minx; xPlotHi = maxx; yPlotLow = miny; yPlotHi = maxy];
If[pltAspect == 2,
  If[minx > miny,
   xPlotLow = miny; yPlotLow = miny,
```

```
    xPlotLow = minx; yPlotLow = minx ]];
If[pltAspect == 2,
  If[maxx < maxy,
   xPlotHi = maxy; yPlotHi = maxy,
   xPlotHi = maxx; yPlotHi = maxx]];
If[pltAspect == 1, aRatio = 1 / N[GoldenRatio]];
If[pltAspect == 2, aRatio = 1];
If[pltAspect == 3, aRatio = Automatic];

tmpPoints = Transpose[List[xAxis, yAxis]];
pltPoints = Table[tmpPoints[[groupPosns[[j]]]], {j, numGroups}];
iconList = Flatten[Table[
    {Graphics[{EdgeForm[{Thickness[0.005], Black}],
       Hue[N[((numGroups + 1) - j) / numGroups]],
       Disk[{0, 0}, Scaled[iconSize]]}]}, {j, numGroups}]];

If[lch == 1 || ptsJoin == 1,
 Do[
  pltTable[[k, 1]] =
   ListPlot[pltPoints[[k]], AspectRatio → aRatio, Frame → True, Joined → False,
    Axes → False, PlotRange → {{xPlotLow, xPlotHi}, {yPlotLow, yPlotHi}},
    PlotRangePadding → Scaled[pltPad], Ticks → Automatic, FrameLabel →
     {lab1, lab2}, PlotMarkers → iconList[[k]], ImageSize → pltSize, LabelStyle →
     Directive[FontSize → 14, Black, FontFamily → "Arial"]], {k, numGroups}],
 Do[
  pltTable[[k, 1]] =
   ListPlot[pltPoints[[k]], Frame → True, Axes → False, AspectRatio → aRatio,
    PlotRange → {{xPlotLow, xPlotHi}, {yPlotLow, yPlotHi}}, PlotRangePadding →
     Scaled[pltPad], Ticks → Automatic, FrameLabel → {lab1, lab2},
    LabelStyle → Directive[Black, FontSize → 14, FontFamily → "Arial"],
    ImageSize → pltSize, PlotStyle → Directive[Disk[],
      Hue[N[((numGroups + 1) - k) / numGroups]], EdgeForm[{Thickness[1.0], Black}],
      PointSize[Scaled[iconSize - 0.009]]]], {k, numGroups}]]

If[ptsJoin == 2,
  Do[
   pltTable[[k, 2]] = ListLinePlot[pltPoints[[k]],
     AspectRatio → aRatio, Frame → True, Joined → True, Axes → False,
     PlotStyle → Directive[Hue[N[((numGroups + 1) - k) / numGroups]], Thin],
     PlotRange → {{xPlotLow, xPlotHi}, {yPlotLow, yPlotHi}},
     PlotRangePadding → Scaled[pltPad], Ticks → Automatic,
     FrameLabel → {lab1, lab2}, ImageSize → pltSize, LabelStyle →
      Directive[FontSize → 14, Black, FontFamily → "Arial"]], {k, numGroups}]];

If[lch == 2,
  Do[
   hull = ConvexHullMesh[pltPoints[[k]]];
```

```
    pltTable[[k, 3]] = HighlightMesh[hull,
      Style[2, Opacity[0.2], Hue[N[((numGroups + 1) - k) / numGroups]]],
      Frame → True, Axes → False, AspectRatio → aRatio,
      PlotRange → {{xPlotLow, xPlotHi}, {yPlotLow, yPlotHi}}, PlotRangePadding →
       Scaled[pltPad], Ticks → Automatic, FrameLabel → {lab1, lab2},
      LabelStyle → Directive[Black, FontSize → 14, FontFamily → "Arial"],
      ImageSize → pltSize], {k, numGroups}]];

If[ptsJoin == 1 && lch == 1, p0 = Show[pltTable[[All, 1]]]];
If[ptsJoin == 2 && lch == 1, p0 = Show[pltTable[[All, 2]], pltTable[[All, 1]]]];
If[ptsJoin == 1 && lch == 2, p0 = Show[pltTable[[All, 3]], pltTable[[All, 1]]]];
If[ptsJoin == 2 && lch == 2,
  p0 = Show[pltTable[[All, 3]], pltTable[[All, 2]], pltTable[[All, 1]]]];

p1 = Labeled[p0, "            Trained Feature-Space Plot", Top,
    LabelStyle → Directive[FontSize → 18, Bold, FontFamily → "Arial"]];
g1 = Grid[Table[
     {Graphics[{EdgeForm[{Thin, Black}], Hue[N[((numGroups + 1) - j) / numGroups]],
         Disk[]}]}, {j, numGroups}], Frame → False, ItemSize → 0.9];
g2 = Grid[Partition[groupNames, 1], Alignment → Left,
    BaseStyle → {FontFamily → "Arial", FontSize → 13, Italic}];
p2 = Labeled[Text[Grid[{{g1, g2}}, Alignment → Bottom, Frame → True]], "Legend",
    Top, LabelStyle → Directive[Black, FontSize → 18, Bold, FontFamily → "Arial"]];

plt2D = Grid[{{p1, p2}}, BaselinePosition → Top, Alignment → Top]
```

Export trained feature space plot

In[•]:=
```
filenameout = SystemDialogInput["FileSave"];
Export[filenameout, plt2D, "TIFF", ImageResolution → 150]
```

Jackknife estimate of classification performance

In[•]:=
```
jackID = Table[" ", {kgTrain}, {2}];
Do[jackID[[i, 1]] = groupTrain[[i]], {i, kgTrain}];
```

In[•]:=
```
i = 20;

jackTrainData = Drop[trainData, {i}];
jackGroupTrain = Drop[groupTrain, {i}];
jackTrainingData = Table[toRule@RandomSample[jackTrainData, 2], comb];
jackResults = NetTrain[net, jackTrainingData, All];
jackTrained = jackResults["TrainedNet"];
jackEmbedding = NetExtract[jackTrained, "Net"];

jackTrained = jackResults["TrainedNet"];
jackEmbedding = NetExtract[jackTrained, "Net"];
jackGroups = KeySort@GroupBy[jackTrainData, Last → First];
jackPoints = Map[embedding /* Normal, jackGroups];
jackC = Classify[jackPoints];

testImage = embedding[trainData[[i, 1]]];
jackID[[i, 2]] = jackC[testImage];
```

In[•]:=
```
testImage
```

Out[•]= {2.48759, -1.54285}

In[•]:=
```
MatrixForm[jackID]
```

In[•]:=
```
{nc} = Dimensions[gpNamesTrain];
jackConfMat = Table[0, {nc}, {nc}];

Do[
 Do[
  If[ jackID[[i, 1]] == gpNamesTrain[[j]] && jackID[[i, 2]] == jackID[[i, 1]],
   jackConfMat[[j, j]] = jackConfMat[[j, j]] + 1];
  If[ jackID[[i, 1]] == gpNamesTrain[[j]] && jackID[[i, 2]] ≠ jackID[[i, 1]],
   Do[
    If[jackID[[i, 2]] == gpNamesTrain[[jj]], jackConfMat[[j, jj]] =
      jackConfMat[[j, jj]] + 1], {jj, nGps}]], {j, nGps}], {i, kgTrain}]

jcMatrix = Table[" ", {nGps + 4}, {nGps + 4}];
jcMatrix[[1, 1]] = Text["Groups"];
jcMatrix[[1, nGps + 2]] = Text[Rotate["No. Correct", 90 Degree]];
jcMatrix[[1, nGps + 3]] = Text[Rotate["Group Total", 90 Degree]];
jcMatrix[[1, nGps + 4]] = Text[Rotate["Percent Correct", 90 Degree]];
Do[jcMatrix[[i + 1, 1]] = Text[gpNamesTrain[[i]]], {i, nGps}];
Do[jcMatrix[[1, i + 1]] = Text[Rotate[gpNamesTrain[[i]], 90 Degree]], {i, nGps}]
Do[
  If[jackConfMat[[i, j]] == 0, jcMatrix[[i + 1, j + 1]] = Text["-"],
   jcMatrix[[i + 1, j + 1]] = Text[jackConfMat[[i, j]]]], {j, nGps}, {i, nGps}];
jcMatrix[[nGps + 2, 1]] = Text["No. Correct"];
jcMatrix[[nGps + 3, 1]] = Text["Group Total"];
jcMatrix[[nGps + 4, 1]] = Text["Percent Correct"];
```

```
Do[jcMatrix[[i + 1, nc + 2]] = Text[jackConfMat[[i, i]]], {i, nGps}];
jcMatrix[[nGps + 2, nGps + 2]] = Text[Total[Diagonal[jackConfMat]]];
jcMatrix[[nGps + 2, nGps + 3]] = Text[Total[Total[jackConfMat]]];
jcMatrix[[nGps + 2, nGps + 4]] =
  Text[PaddedForm[N[Total[Diagonal[jackConfMat]] / Total[Total[jackConfMat]]] *
     100.0, {4, 1}]];
Do[jcMatrix[[i + 1, nGps + 3]] = Text[Total[jackConfMat[[i, All]]]], {i, nGps}]
Do[
 If[
  jackConfMat[[i, i]] == 0, cMatrix[[i + 1, nGps + 4]] = Text["0.0"],
  jcMatrix[[i + 1, nGps + 4]] = Text[PaddedForm[
     N[Take[jackConfMat[[i, i]]] / Total[Take[jackConfMat[[i, All]]]]] * 100.0,
     {4, 1}]]]], {i, nGps}]
Do[jcMatrix[[nGps + 2, i + 1]] = Text[jackConfMat[[i, i]]], {i, nGps}]
Do[jcMatrix[[nGps + 3, i + 1]] = Text[Total[jackConfMat[[All, i]]]], {i, nGps}]
jcMatrix[[nGps + 3, nc + 2]] = Text[Total[Total[jackConfMat]]];
jcMatrix[[nGps + 4, nc + 2]] =
  Text[PaddedForm[N[Total[Diagonal[jackConfMat]] / Total[Total[jackConfMat]]] *
     100.0, {4, 1}]];
Do[
 If[
  jackConfMat[[i, i]] == 0, cMatrix[[nGps + 4, i + 1]] = Text["0.0"],
  jcMatrix[[nGps + 4, i + 1]] = Text[PaddedForm[
     N[Take[jackConfMat[[i, i]]] / Total[Take[jackConfMat[[All, i]]]]] * 100.0,
     {4, 1}]]]], {i, nGps}]

Labeled[Grid[jcMatrix, Alignment → {{1 → Right, nGps + 4 → Right}, {Bottom, Right}},
  Frame → True, Dividers → {{2 → True, -4 → True}, {2 → True, -4 → True}}],
 "Jackknife ID Confusion Matrix", Top,
 LabelStyle → Directive[FontSize → 16, Bold, FontFamily → "Arial"]]
```

Export jackknife ID confusion matrix

```
In[•]:= jcMatrix = Table[" ", {nGps + 4}, {nGps + 4}];
jcMatrix[[1, 1]] = "Groups";
jcMatrix[[1, nGps + 2]] = "No. Correct";
jcMatrix[[1, nGps + 3]] = "Group Total";
jcMatrix[[1, nGps + 4]] = "Percent Correct";
Do[jcMatrix[[i + 1, 1]] = gpNamesTrain[[i]], {i, nGps}];
Do[jcMatrix[[1, i + 1]] = gpNamesTrain[[i]], {i, nGps}];
Do[
  If[jackConfMat[[i, j]] == 0, cMatrix[[i + 1, j + 1]] = "-",
   jcMatrix[[i + 1, j + 1]] = jackConfMat[[i, j]]], {j, nGps}, {i, nGps}];
jcMatrix[[nGps + 2, 1]] = "No. Correct";
jcMatrix[[nGps + 3, 1]] = "Group Total";
jcMatrix[[nGps + 4, 1]] = "Percent Correct";
Do[jcMatrix[[i + 1, nGps + 2]] = jackConfMat[[i, i]], {i, nGps}];
jcMatrix[[nGps + 2, nGps + 2]] = Total[Diagonal[jackConfMat]];
jcMatrix[[nGps + 2, nGps + 3]] = Total[Total[jackConfMat]];
jcMatrix[[nGps + 2, nGps + 4]] =
  N[Total[Diagonal[jackConfMat]] / Total[Total[jackConfMat]]] * 100.0;
Do[jcMatrix[[i + 1, nc + 3]] = Total[jackConfMat[[i, All]]], {i, nGps}];
Do[
  If[
   jackConfMat[[i, i]] == 0, jcMatrix[[i + 1, nGps + 4]] = 0.0,
   jcMatrix[[i + 1, nGps + 4]] = N[Take[jackConfMat[[i, i]]] /
       Total[Take[jackConfMat[[i, All]]]]] * 100.0], {i, nGps}];
Do[jcMatrix[[nGps + 2, i + 1]] = jackConfMat[[i, i]], {i, nGps}];
Do[jcMatrix[[nGps + 3, i + 1]] = Total[jackConfMat[[All, i]]], {i, nGps}];
jcMatrix[[nGps + 3, nGps + 2]] = Total[Total[jackConfMat]];
jcMatrix[[nGps + 4, nGps + 2]] =
  N[Total[Diagonal[jackConfMat]] / Total[Total[jackConfMat]]] * 100.0;
Do[
  If[
   jackConfMat[[i, i]] == 0, cMatrix[[nGps + 4, i + 1]] = 0.0,
   jcMatrix[[nGps + 4, i + 1]] =
    N[Take[jackConfMat[[i, i]]] / Total[Take[jackConfMat[[All, i]]]]] *
     100.0], {i, nGps}];

filenameout = SystemDialogInput["FileSave"];
Export[filenameout, jcMatrix, "CSV", "TextDelimiters" → ""]
```
