# Supplementary material for: Machine-learning strategies for testing patterns of morphological variation in small samples: sexual dimorphism in gray wolf (Canis lupus) crania
Source: BMC Biol. 2020 Sep 3;18:113. doi: 10.1186/s12915-020-00832-1 (PMC7470621; doi:10.1186/s12915-020-00832-1)
Supplement: Supplementary file 5 — Additional file 5. An archive of all code listings for all data procession/analysis software employed in this investigation. [file 12915_2020_832_MOESM5_ESM.zip › SI File 5/PCA (vers. 5.8).pdf]

Principal Component Analysis (PCA)

This program accepts a data matrix in standard format, performs a principal component analsyis on the data columns, and allows the result to be viewed as 2 D or (interactive) 3 D scatterplots. Full support is provided for tracking groups that have been identified in the datafile.

Author : N. MacLeod

Version : 5.8

Date : 28 May 2019

Reference : MacLeod (2007)

Initialize libraries.

In[40]:=
```
<< ComputationalGeometry`
```

Read in data file & partition into datasets.

In[41]:=
```
filenamein = SystemDialogInput["FileOpen"];

x1 = Import[filenamein, "CSV"];
filenamein

{n1, m1} = Dimensions[x1];

varNames = Flatten[Take[x1, 1]];
x2 = Drop[x1, 1];
varNames = Drop[varNames, 1];
varNames = Drop[varNames, 1];

objNames = Flatten[Take[x2, n1 - 1, 1]];
x2 = Drop[x2, 0, 1];

Group = Flatten[Take[x2, n1 - 1, 1]];
numGroups = Length[Union[Group]];
x2 = Drop[x2, 0, 1];
kg = Length[Union[Group]];

{n2, m2} = Dimensions[x2];
Print["No. of Objects: ", n2]
Print["No. of Variables: ", m2]
Print["No. of Groups: ", kg]

varNames;
objNames;
Group;
x2;
```

Data transformations (optional)

Specify global data transformations (if any).
Note : Remember you cannot subsequently take the logarithm of mean – centered or standardized data. If you wish to perform such an analysis you must shift the mean – centered or standardized data by a constant (e.g., 1, 10)

In[1]:=
```
Panel[Labeled[Column[
   {Row[{Panel[Labeled[PopupMenu[Dynamic[meanTrans], {1 → "No", 2 → "Yes"}],
        "Mean center data?", Top, LabelStyle →
         Directive[FontSize → 12, Bold, FontFamily → "Arial"]]], "  ",
      Panel[Labeled[PopupMenu[Dynamic[logTrans], {1 → "No", 2 → "Yes"}],
        "Log10-trasform data?", Top, LabelStyle →
         Directive[FontSize → 12, Bold, FontFamily → "Arial"]]], "  ",
      Panel[Labeled[PopupMenu[Dynamic[stdTrans], {1 → "No", 2 → "Yes"}],
        "Standardize data?", Top,
        LabelStyle → Directive[FontSize → 12, Bold, FontFamily → "Arial"]]]}],
    Row[{Panel[Labeled[PopupMenu[Dynamic[shiftTrans], {1 → "No", 2 → "Yes"}],
        "Shift data by a constant?", Top, LabelStyle →
         Directive[FontSize → 12, Bold, FontFamily → "Arial"]]], "  ",
      Panel[Labeled[InputField[Dynamic[knsnt], FieldSize → 5],
        "Enter shift constant value.", Top,
        LabelStyle → Directive[FontSize → 12, Bold, FontFamily → "Arial"]]]}]},
   Center], "Global Data Transform Options", Top, LabelStyle →
   Directive[FontSize → 18, Bold, FontFamily → "Arial"]]]
meanTrans = 1; logTrans = 1; stdTrans = 1; shiftTrans = 1; knsnt = 10;
```

Out[1]=

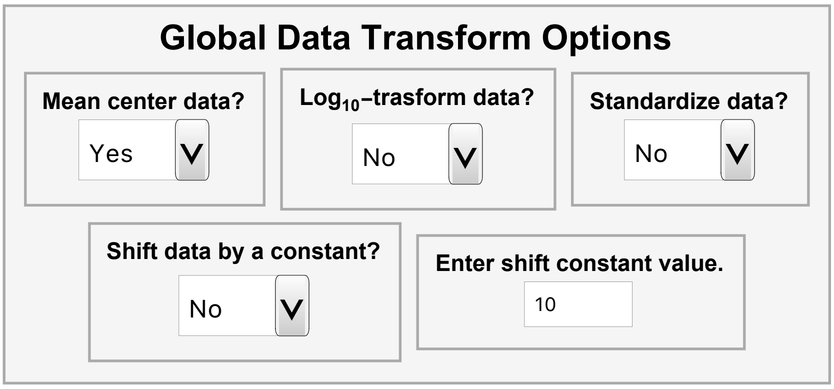

Perform global data transformation (optional)

In[63]:=
```
If[meanTrans == 2,
  mVec = N[Mean[x2]];
  Do[x2[[i]] = x2[[i]] - mVec, {i, n2}]];
If[logTrans == 2, x2 = N[Log10[x2]]];
If[stdTrans == 2, x2 = Standardize[x2]];
If[shiftTrans == 2, x2 = x2 + knsnt];
```

Export mean vector (optional; may be useful in modelling).

In[∘]:=
```
filenameout = SystemDialogInput["FileSave"];
Export[filenameout, mVec, "csv"]
```

Out[∘]=
```
/Users/nm/Desktop/Lupus II/Lateral/EFourier Data
  & Results/PCA Results/Lateral Outlines (Mean Vector).csv
```

Export processed dataset (optional; may be useful in other analyses).

```
x2Trans = Table[" ", {n2 + 1}, {m2 + 2}];
x2Trans[[1, 1]] = "Object";
x2Trans[[1, 2]] = "Group";
Do[x2Trans[[1, j + 2]] = varNames[[j]], {j, m2}]
Do[x2Trans[[i + 1, 1]] = objNames[[i]], {i, n2}]
Do[x2Trans[[i + 1, 2]] = Group[[i]], {i, n2}]
Do[x2Trans[[i + 1, j + 2]] = x2[[i, j]], {i, n2}, {j, m2}]

filenameout = SystemDialogInput["FileSave"];
Export[filenameout, x2Trans, "CSV", "TextDelimiters" → ""]
```

Choose basis matrix and eigenanalysis method

In[3]:=
```
Panel[Labeled[Row[{Panel[
      Labeled[PopupMenu[Dynamic[basisMat], {1 → "Covariance Matrix (Calculated)",
         2 → "Correlation Matrix (Calculated)", 3 → "Covariance Matrix (Input)",
         4 → "Correlation Matrix (Input)"}], "Choose similarity matrix.", Top,
       LabelStyle → Directive[FontSize → 12, FontFamily → "Arial"]]], "  ",
     Panel[Labeled[PopupMenu[Dynamic[eMethod], {1 → "Std. Eigenanalysis",
         2 → "Singular Value Decomposition"}], "Choose eigenanalysis method.",
       Top, LabelStyle → Directive[FontSize → 12, FontFamily → "Arial"]]]}],
  "Choose Analysis Methods", Top, LabelStyle →
   Directive[FontSize → 14, Bold, FontFamily → "Arial"]]]
basisMat = 1; eMethod = 1;
```

Out[3]=

Choose Analysis Methods

Choose similarity matrix.	Choose eigenanalysis method.
Covariance Matrix (Calculated) ∨	Std. Eigenanalysis ∨

Perform eigenanalysis.

In[67]:=
```
If[basisMat == 1, coVar = Covariance[x2]];
If[basisMat == 2, coVar = Correlation[x2]];
If[basisMat > 2, filenamein = SystemDialogInput["FileOpen"];
  coVar = Import[filenamein, "CSV"]];
If[n2 > m2, mvecs = m2, mvecs = n2];
If[n2 > m2, mvecs = m2, mvecs = n2];
If[eMethod == 1,
  eVals = Eigenvalues[coVar];
  w = Eigenvectors[coVar];
  eVecs = Transpose[w]];
If[eMethod == 2,
  {w, u, v} = SingularValueDecomposition[coVar];
  eVals = N[Diagonal[u], 3];
  eVecs = w];
If[basisMat == 1 || basisMat == 3,
  eScores = x2.eVecs, eScores = Standardize[x2].eVecs];

s1 = Total[eVals];
evalTable = Table[0.0, {i, mvecs}, {j, 4}];
Do[evalTable[[i, 1]] = i, {i, 1, mvecs}];
Do[evalTable[[i, 2]] = eVals[[i]], {i, 1, mvecs}];
Do[evalTable[[i, 3]] = eVals[[i]] 100.` / s1, {i, 1, mvecs}];
Do[evalTable[[i, 4]] = evalTable[[i, 3]], {i, 1, mvecs}];
Do[evalTable[[i, 4]] = evalTable[[i - 1, 4]] + evalTable[[i, 3]], {i, 2, mvecs}];

tabHeads =
  {"Component", " Eigenvalue", "  Variance (%)", "  Cum. Variance (%)"};
t2 = Partition[Flatten[Join[tabHeads, evalTable]], 4];
t3 = Partition[Flatten[t2], 4];

Do[
 Do[
  t3[[i, j]] = PaddedForm[t3[[i, j]], {5, 3}], {i, 2, mvecs + 1}], {j, 2, 4}]

Labeled[Grid[t3, BaseStyle → (FontFamily → "Arial"),
  Alignment → {{Center, Right, Right, Right}}, Frame → True,
  Dividers → {{True, True}, {True, True}}], "Eigenvalue Table", Top,
 LabelStyle → Directive[FontSize → 14, Bold, FontFamily → "Arial"]]
```

Specify no. of axes to display

In[5]:=
```
Panel[Labeled[InputField[Dynamic[noAxes], FieldSize → 10],
   "Enter number of PC axes to retain.", Top,
   LabelStyle → Directive[FontSize → 12, FontFamily → "Arial"]]]
noAxes = mvecs;
```

Out[5]=

Enter number of PC axes to retain.

18

Display PCA Results (optional).

Display PC loadings (optional).

```
evecTable = Table[0.0, {i, m2 + 1}, {j, noAxes + 1}];
Do[evecTable[[i + 1, 1]] = varNames[[i]], {i, m2}]
evecTable[[1, 1]] = "Variable";
Do[evecTable[[1, i + 1]] = StringJoin["PC-", ToString[i]], {i, noAxes}]
Do[Do[evecTable[[i + 1, j + 1]] = PaddedForm[eVecs[[i, j]], {5, 3}], {i, m2}],
  {j, noAxes}]
Labeled[Grid[evecTable, BaseStyle → (FontFamily → "Arial"),
   Alignment → {{Center, Right, Right, Right, Right, Right}}, Frame → True,
   Dividers → {{True, True}, {True, True}}], "Eigenvector Table", Top,
  LabelStyle → Directive[Black, Bold, FontSize → 14, FontFamily → "Arial"]]
```

Reverse direction of eigenvectors (optional).

```
eVecs = eVecs * -1;
If[basisMat == 1 || basisMat == 3,
   eScores = x2.eVecs, eScores = Standardize[x2].eVecs];
```

Display PC scores (optional).

In[∘]:=
```
scrTable = Table[0.0, {i, n2 + 1}, {j, noAxes + 1}];
Do[scrTable[[i + 1, 1]] = objNames[[i]], {i, n2}]
scrTable[[1, 1]] = "Objects";
Do[scrTable[[1, i + 1]] = StringJoin["PC-", ToString[i]], {i, noAxes}]
Do[Do[scrTable[[i + 1, j + 1]] = PaddedForm[eScores[[i, j]], {5, 3}], {i, n2}],
  {j, noAxes}]
Labeled[Grid[scrTable, BaseStyle → (FontFamily → "Arial"),
   Alignment → {{Left, Right, Right, Right, Right, Right}}, Frame → True,
   Dividers → {{True, True}, {True, True}}], "Eigenscore Table", Top,
  LabelStyle → Directive[Black, Bold, FontSize → 14, FontFamily → "Arial"]]
```

Calculate original, reproduced and residual covariance/correlation matrices

```
In[•]:= Λ = Table[0.0, {noAxes}, {noAxes}];
Do[Λ[[i, i]] = √eVals[[i]] , {i, noAxes}];
fVecs = Take[eVecs, All, noAxes].Λ;
If[basisMat == 1 || basisMat == 3,
  fScores = x2.fVecs, fScores = Standardize[x2].fVecs];

covarTable = Table[" ", {m2 + 1}, {m2 + 1}];
reproTable = Table[" ", {m2 + 1}, {m2 + 1}];
residTable = Table[" ", {m2 + 1}, {m2 + 1}];
repro = fVecs.Transpose[fVecs];
resid = coVar - repro;
```

Display original, reproduced and residual covariance/correlation matrices.
[Note: only invoke this option if your covariance matrix is small (c. 10 variables)]

```
If[basisMat == 1 || basisMat == 3,
  title1 = StringJoin["Original ", ToString["Covariances"]],
  title1 = StringJoin["Original ", ToString["Correlations"]]];
If[basisMat == 1 || basisMat == 3,
  title2 = StringJoin["Reproduced ", ToString["Covariances"]],
  title2 = StringJoin["Reproduced ", ToString["Correlations"]]];
If[basisMat == 1 || basisMat == 3,
  title3 = StringJoin["Residual ", ToString["Covariances"]],
  title3 = StringJoin["Residual ", ToString["Correlations"]]];

Do[covarTable[[1, j + 1]] = varNames[[j]], {j, m2}]
Do[covarTable[[i + 1, 1]] = varNames[[i]], {i, m2}]
Do[covarTable[[i + 1, j + 1]] = coVar[[i, j]], {i, m2}, {j, m2}]
t5 = covarTable;
Do[
 Do[
  t5[[i, j]] = PaddedForm[t5[[i, j]], {5, 4}], {i, 2, m2 + 1}], {j, 2, m2 + 1}]
Labeled[Grid[t5, BaseStyle → (FontFamily → "Arial"), Alignment → {{Center}},
  Frame → True, Dividers → {{True, True}, {True, True}}], title1, Top,
 LabelStyle → Directive[Bold, FontSize → 14, FontFamily → "Arial"]]

Do[reproTable[[1, j + 1]] = varNames[[j]], {j, m2}]
Do[reproTable[[i + 1, 1]] = varNames[[i]], {i, m2}]
Do[reproTable[[i + 1, j + 1]] = repro[[i, j]], {i, m2}, {j, m2}]
t6 = reproTable;
Do[
 Do[
  t6[[i, j]] = PaddedForm[t6[[i, j]], {5, 4}], {i, 2, m2 + 1}], {j, 2, m2 + 1}]
Labeled[Grid[t6, BaseStyle → (FontFamily → "Arial"), Alignment → {{Center}},
  Frame → True, Dividers → {{True, True}, {True, True}}], title2, Top,
 LabelStyle → Directive[Bold, FontSize → 14, FontFamily → "Arial"]]

Do[residTable[[1, j + 1]] = varNames[[j]], {j, m2}]
Do[residTable[[i + 1, 1]] = varNames[[i]], {i, m2}]
Do[residTable[[i + 1, j + 1]] = resid[[i, j]], {i, m2}, {j, m2}]
t7 = residTable;
Do[
 Do[
  t7[[i, j]] = PaddedForm[t7[[i, j]], {5, 4}], {i, 2, m2 + 1}], {j, 2, m2 + 1}]
Labeled[Grid[t7, BaseStyle → (FontFamily → "Arial"), Alignment → {{Center}},
  Frame → True, Dividers → {{True, True}, {True, True}}], title3, Top,
 LabelStyle → Directive[Bold, FontSize → 14, FontFamily → "Arial"]]
```

Export original reproduced and residual covariances/correlations tables (optional).

```
filenameout = SystemDialogInput["FileSave"];
Export[filenameout, covarTableTable, "CSV", "TextDelimiters" → ""]
filenameout = SystemDialogInput["FileSave"];
Export[filenameout, reproTable, "CSV", "TextDelimiters" → ""]
filenameout = SystemDialogInput["FileSave"];
Export[filenameout, residTable, "CSV", "TextDelimiters" → ""]
```

Display scatterplot of original vs reproduced covariance/correlation values w/ cophenetic correlation (optional)

```
In[•]:= pltPad = 0.10;
iconList = Flatten[Table[
    {Graphics[{EdgeForm[{Thin, Black}], Hue[1 / 3], Disk[{0, 0}, Scaled[0.035]]}]},
    {j, 1}]];
If[basisMat == 1 || basisMat == 3, tit1 = "Covariance Values",
  tit1 = "Correlation Values"];
title = StringJoin["       Original vs Reproduced ", ToString[tit1]];
orig = Flatten[coVar];
rep = Flatten[repro];
ccText = StringJoin["Cophenetic Correlation:",
   ToString[PaddedForm[Correlation[orig, rep], {5, 4}]]];
ccText = Text[ccText, BaseStyle → {FontSize → 14, FontFamily → "Arial"}];
lData = Riffle[Flatten[coVar], Flatten[repro]];
pos = {Min[lData], Max[lData]};
int = (pos[[2]] - pos[[1]]) * 0.05;
lowpos = pos[[1]] - int;
highpos = pos[[2]] + int;
txtData = {Text[ccText, pos, {-1, 0}]};
pData = Partition[lData, 2];

lnPlot = Graphics[{Black, Thin, Line[{{lowpos, lowpos}, {highpos, highpos}}]},
   AspectRatio → 1 / GoldenRatio, Frame → True, Axes → False,
   PlotRange → {{pos[[1]], pos[[2]]}, {pos[[1]], pos[[2]]}},
   PlotRangePadding → Scaled[pltPad], Ticks → Automatic,
   FrameLabel → {"Original Values", "Reproduced Values"}, ImageSize → 500,
   LabelStyle → Directive[Black, FontSize → 14, FontFamily → "Arial"]];
ptPlot = ListPlot[pData, AspectRatio → 1 / GoldenRatio, Frame → True,
   Axes → False, PlotRange → Automatic, PlotMarkers → iconList,
   PlotRangePadding → Scaled[pltPad], Ticks → Automatic,
   FrameLabel → {"Original Values", "Reproduced Values"}, ImageSize → 500,
   LabelStyle → Directive[Black, FontSize → 14, FontFamily → "Arial"]];
txtPlot = Graphics[txtData, AspectRatio → 1 / GoldenRatio, Frame → True,
   Axes → False, PlotRange → {{pos[[1]], pos[[2]]}, {pos[[1]], pos[[2]]}},
   PlotRangePadding → Scaled[pltPad], Ticks → Automatic,
   FrameLabel → {"Original Values", "Reproduced Values"}, ImageSize → 500,
   LabelStyle → Directive[Black, FontSize → 14, FontFamily → "Arial"]];
ccPlot = Labeled[Show[{lnPlot, ptPlot, txtPlot}], title, Top,
  LabelStyle → Directive[FontSize → 18, FontFamily → "Arial"]]
```

Export the cophenetic correlation plot.

In[•]:=
```
filenameout = SystemDialogInput["FileSave"];
Export[filenameout, ccPlot, "TIFF", ImageResolution → 150]
```

Out[•]=
```
/Users/nm/Projects/Storage/MacLeod/Meetings/2018/06
  China Trip/Presentations/Short Course/GBDB
  Morphometrics Workshop/Data Analysis Results/Procrustes
  Alignment/Outlines/PCA Results/Cophenetic Correlation Plot.tif
```

Display PC score distances from origin (= mean).

```
distTable = Table[" ", {n2 + 1}, {2}];
origin = Table[0.0, {noAxes}];
distTable[[1, 1]] = "Objects";
distTable[[1, 2]] = "Distance From Origin";
rScores = Take[eScores, All, noAxes];
Do[distTable[[i + 1, 1]] = objNames[[i]];
 distTable[[i + 1, 2]] = EuclideanDistance[origin, rScores[[i]]], {i, n2}]
Labeled[Grid[distTable, BaseStyle → (FontFamily → "Arial"),
  Alignment → {{Left, Center}}, Frame → True,
  Dividers → {{True, True}, {True, True}}], "Distance Table", Top,
 LabelStyle → Directive[Black, Bold, FontSize → 14, FontFamily → "Arial"]]
```

Export distances from origin (= mean) .

```
filenameout = SystemDialogInput["FileSave"];
Export[filenameout, distTable, "CSV", "TextDelimiters" → ""]
```

Detect groups based on local optimization (optional).

Specify group inference method (if applicable)

In[•]:=
```
Panel[Labeled[InputField[Dynamic[noVar], FieldSize → 5],
  "Enter number of varables to include.", Top,
  LabelStyle → Directive[FontSize → 12, Bold, FontFamily → "Arial"]]]
```

Out[•]=

Enter number of varables to include.

25

In[7]:=
```
Panel[
 Labeled[Column[{Panel[Labeled[PopupMenu[Dynamic[klustMeth], {1 → "Automatic",
        2 → "Single-linkage clustering", 3 → "Density-based spatial clustering",
        4 → "High-density region displacement",
        5 → "Jarvis-Patrick clustering", 6 → "k-means clustering",
        7 → "Mean-shift clustering", 8 → "Medoid partitioning",
        9 → "Minimum spanning tree clustering", 10 → "Spectral clustering",
        11 → "Variational Gaussian mixture clustering"}],
      "Select data clustering method.", Top, LabelStyle →
       Directive[FontSize → 12, Bold, FontFamily → "Arial"]]],
    Row[{Panel[Labeled[InputField[Dynamic[noVar], FieldSize → 5],
        "Enter number of varables to include.", Top,
        LabelStyle → Directive[FontSize → 12, Bold, FontFamily → "Arial"]]],
      "  ", Panel[Labeled[InputField[Dynamic[ng], FieldSize → 5],
        "Enter number of groups (if applicable).", Top,
        LabelStyle → Directive[FontSize → 12, Bold, FontFamily → "Arial"]]]}]},
   Center], "Data Clustering Options", Top, LabelStyle →
   Directive[FontSize → 18, Bold, FontFamily → "Arial"]]]
klustMeth = 1; noVar = noAxes; ng = 3;
```

Out[7]=

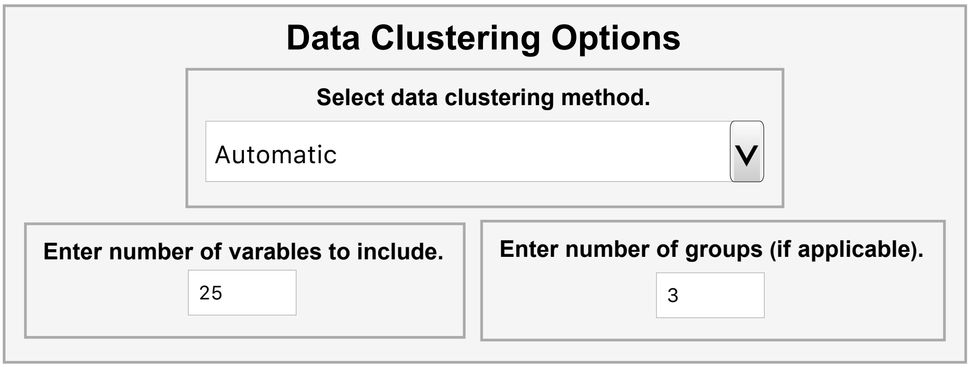

Perform data clustering (optional).

In[•]:=
```
If[klustMeth == 1, meth = Automatic];
If[klustMeth == 2, meth = "Agglomerate"];
If[klustMeth == 3, meth = "DBSCAN"];
If[klustMeth == 4, meth = "NeighborhoodContraction"];
If[klustMeth == 5, meth = "JarvisPatrick"];
If[klustMeth == 6, meth = "KMeans"];
If[klustMeth == 7, meth = "MeanShift"];
If[klustMeth == 8, meth = "KMedoids"];
If[klustMeth == 9, meth = "SpanningTree"];
If[klustMeth == 10, meth = "Spectral"];
If[klustMeth == 11, meth = "GaussianMixture"];

p1 = Take[eScores, All, noVar];
optGroups = FindClusters[p1, Method → meth, PerformanceGoal → "Quality"];
{kg3} = Dimensions[optGroups];
optGpNames = Table[" ", {n2}];

Do[
 l1 = StringJoin["Group ", ToString[k]];
 {n3, m3} = Dimensions[optGroups[[k]]];
 Do[
  Do[If[optGroups[[k, j]] == p1[[i]], optGpNames[[i]] = l1], {i, n2}], {j, n3}],
 {k, kg3}]
kg = kg3;
numGroups = kg;
Group = optGpNames;
```

Out[•]=
```
$Aborted
```

Set: Lists {kg3} and {} are not the same shape.

Do: Iterator {k, kg3} does not have appropriate bounds.

Out[•]=
```
Do[l1 = Group <> ToString[k];
 {n3, m3} = Dimensions[optGroups⟦k⟧];
 Do[Do[If[optGroups⟦k, j⟧ == p1⟦i⟧, optGpNames⟦i⟧ = l1], {i, n2}], {j, n3}],
 {k, kg3}]
```

Plot single – axis histogram.

Specify single – variable plot (histogram) options.

In[87]:=
```
pcNames = Table[StringJoin["PC-", ToString[i]], {i, noAxes}];
Panel[Labeled[Column[{
    Row[{Panel[Labeled[PopupMenu[Dynamic[axisName], pcNames],
        "Select component to be plotted.", Top,
        LabelStyle → Directive[FontSize → 12, Bold, FontFamily → "Arial"]]],
      "  ", Panel[Labeled[PopupMenu[Dynamic[histType],
         {1 → "Stacked", 2 → "Overlapped"}], "Select histogram type.", Top,
        LabelStyle → Directive[FontSize → 12, Bold, FontFamily → "Arial"]]]}],
    Row[{Panel[Labeled[InputField[Dynamic[noBins], FieldSize → 5],
        "Enter no. of histogram bins.", Top, LabelStyle →
         Directive[FontSize → 12, Bold, FontFamily → "Arial"]]], "  ",
      Panel[Labeled[InputField[Dynamic[hSize], FieldSize → 5],
        "Enter histogram plot size.", Top,
        LabelStyle → Directive[FontSize → 12, Bold, FontFamily → "Arial"]]]}]},
   Center], "Single Axis (Histogram) Plot Options", Top,
  LabelStyle → Directive[FontSize → 16, Bold, FontFamily → "Arial"]]]
axisName = pcNames[[1]]; noBins = 15; hSize = 500; histType = 1;
```

Out[88]=

Single Axis (Histogram) Plot Options

Select component to be plotted.	Select histogram type.
PC−1	Stacked
**Enter no. of histogram bins.**	**Enter histogram plot size.**
15	500

Construct and display histogram plot .

In[•]:=
```
Do[If[axisName == pcNames[[j]], axis = j], {j, noAxes}];
pltScores = Flatten[Take[eScores, All, {axis}]];
If[histType == 1, htype = "Stacked", htype = "Overlapped"];
gpNames = Union[Group];
groupPosns = Table[Flatten[Position[Group, gpNames[[i]], 1]], {i, numGroups}];
pltPoints = Table[pltScores[[groupPosns[[j]]]], {j, numGroups}];
hueList = Table[Hue[N[((numGroups + 1) - j) / numGroups]], {j, numGroups}];
h1 =
 Labeled[Histogram[pltPoints, noBins, ChartStyle → {hueList}, ChartLayout → htype,
   LabelStyle → Directive[FontSize → 12, Black, FontFamily → "Arial"],
   AxesLabel → {"PC Score", "Frequency"}, ImageSize → hSize,
   ChartLegends → gpNames], StringJoin["Principal Component ", ToString[axis]],
  Top, LabelStyle → Directive[FontSize → 16, Bold, FontFamily → "Arial"]]
```

Export current histogram.

```
filenameout = SystemDialogInput["FileSave"];
Export[filenameout, h1, "TIFF", ImageResolution → 150]
```

```
/Users/nm/Desktop/Varotis Results/CVA Results/CV-1.tif
```

Create 2 D scatterplot .

Specify 2 D plot options.
You must run this code after you read in the data so it can pick up the proper variable names.

In[90]:=
```
pcNames = Table[StringJoin["PC-", ToString[i]], {i, noAxes}];
Panel[
 Labeled[Column[{Row[{Panel[Labeled[PopupMenu[Dynamic[xAxisName], pcNames],
       "Select variable to be plotted on x-Axis.", Top, LabelStyle →
        Directive[FontSize → 12, Bold, FontFamily → "Arial"]]], "  ",
     Panel[Labeled[PopupMenu[Dynamic[yAxisName], pcNames],
       "Select variable to be plotted on y-Axis.", Top,
       LabelStyle → Directive[FontSize → 12, Bold, FontFamily → "Arial"]]]}],
   Row[{
     Panel[Labeled[PopupMenu[Dynamic[pltAspect],
        {1 → "Golden Ratio Plot", 2 → "Square Plot (equi-length axes)",
         3 → "True-Scale Plot (actual axis scales)"}],
       "Enter plot aspect ratio type.", Top, LabelStyle →
        Directive[FontSize → 12, Bold, FontFamily → "Arial"]]], "  ",
     Panel[Labeled[PopupMenu[Dynamic[lch], {1 → "Simple scatterplot",
         2 → "Scatterplot w/ convex hulls"}],
       "Show group domians?", Top, LabelStyle →
        Directive[FontSize → 12, Bold, FontFamily → "Arial"]]], "  ",
     Panel[Labeled[PopupMenu[Dynamic[ptsJoin], {1 → "No", 2 → "Yes"}],
       "Join datapoints?", Top,
       LabelStyle → Directive[FontSize → 12, Bold, FontFamily → "Arial"]]]}],
   Row[{Panel[Labeled[InputField[Dynamic[pltSize], FieldSize → 10],
       "Enter plot size value.", Top,
       LabelStyle → Directive[FontSize → 12, Bold, FontFamily → "Arial"]]],
     "  ", Panel[Labeled[InputField[Dynamic[pltPad], FieldSize → 10],
       "Enter plot margin padding value.", Top, LabelStyle →
        Directive[FontSize → 12, Bold, FontFamily → "Arial"]]], "  ",
     Panel[Labeled[InputField[Dynamic[iconSize], FieldSize → 10],
       "Enter plot icon size value.", Top, LabelStyle →
        Directive[FontSize → 12, Bold, FontFamily → "Arial"]]]}]}, Center],
  "2D Plot Options", Top, LabelStyle → Directive[FontSize → 18,
    Bold, FontFamily → "Arial"]]]
pltSize = 500; iconSize = 0.03; pltPad = 0.1; xAxisName = pcNames[[1]];
yAxisName = pcNames[[2]];
 ptsJoin = 1; dataTrans = 1;  pltAspect = 1 ; lch = 2;
```

Out[91]=

2D Plot Options

Select variable to be plotted on x-Axis.
PC–1

Select variable to be plotted on y-Axis.
PC–2

Enter plot aspect ratio type.
True–Scale Plot (actual axis scales)

Show group domians?
Scatterplot w/ convex hulls

Join datapoints?
No

Enter plot size value.
500

Enter plot margin padding value.
0.1

Enter plot icon size value.
30.

Plot script

In[128]:=

```
pltTable = Table[" ", {numGroups}, {3}];
Do[If[xAxisName == pcNames[[j]], axis1 = j], {j, noAxes}];
Do[If[yAxisName == pcNames[[j]], axis2 = j], {j, noAxes}];

groupNames = Union[Group];
numGroups = Length[groupNames];
groupPosns =
  Table[Flatten[Position[Group, groupNames[[i]], 1]], {i, numGroups}];
eScoresT = Transpose[eScores];

xAxis = eScoresT[[axis1]]; yAxis = eScoresT[[axis2]];
lab1 = StringJoin[{"Principal Component ", ToString[axis1]},
   {" (Var. =", ToString[t3[[axis1 + 1, 3]]]}, {"%)"}];
lab2 = StringJoin[{"Principal Component ", ToString[axis2]},
   {" (Var. =", ToString[t3[[axis2 + 1, 3]]]}, {"%)"}];
maxx =
 Max[
  xAxis];
minx = Min[xAxis];
maxy = Max[yAxis];
miny = Min[yAxis];

If[pltAspect == 1 || pltAspect == 3,
  xPlotLow = minx; xPlotHi = maxx; yPlotLow = miny; yPlotHi = maxy];
If[pltAspect == 2,
  If[minx > miny,
```

```
    xPlotLow = miny; yPlotLow = miny,
    xPlotLow = minx; yPlotLow = minx ]];
If[pltAspect == 2,
  If[maxx < maxy,
    xPlotHi = maxy; yPlotHi = maxy,
    xPlotHi = maxx; yPlotHi = maxx]];
If[pltAspect == 1, aRatio = 1 / N[GoldenRatio]];
If[pltAspect == 2, aRatio = 1];
If[pltAspect == 3, aRatio = Automatic];

tmpPoints = Transpose[List[xAxis, yAxis]];
pltPoints = Table[tmpPoints[[groupPosns[[j]]]], {j, numGroups}];
iconList = Flatten[Table[
     {Graphics[{EdgeForm[{Thickness[0.005], Black}],
        Hue[N[((numGroups + 1) - j) / numGroups]],
        Disk[{0, 0}, Scaled[iconSize]]}]}, {j, numGroups}]];

If[lch == 1 || ptsJoin == 1,
 Do[
  pltTable[[k, 1]] =
   ListPlot[pltPoints[[k]], AspectRatio → aRatio, Frame → True, Joined → False,
    Axes → False, PlotRange → {{xPlotLow, xPlotHi}, {yPlotLow, yPlotHi}},
    PlotRangePadding → Scaled[pltPad], Ticks → Automatic, FrameLabel →
     {lab1, lab2}, PlotMarkers → iconList[[k]], ImageSize → pltSize, LabelStyle →
     Directive[FontSize → 14, Black, FontFamily → "Arial"]], {k, numGroups}],
 Do[
  pltTable[[k, 1]] =
   ListPlot[pltPoints[[k]], Frame → True, Axes → False, AspectRatio → aRatio,
    PlotRange → {{xPlotLow, xPlotHi}, {yPlotLow, yPlotHi}}, PlotRangePadding →
     Scaled[pltPad], Ticks → Automatic, FrameLabel → {lab1, lab2},
    LabelStyle → Directive[Black, FontSize → 14, FontFamily → "Arial"],
    ImageSize → pltSize, PlotStyle → Directive[Disk[],
      Hue[N[((numGroups + 1) - k) / numGroups]], EdgeForm[{Thickness[1.0], Black}],
      PointSize[Scaled[iconSize - 0.009]]]], {k, numGroups}]]

If[ptsJoin == 2,
  Do[
   pltTable[[k, 2]] = ListLinePlot[pltPoints[[k]],
     AspectRatio → aRatio, Frame → True, Joined → True, Axes → False,
     PlotStyle → Directive[Hue[N[((numGroups + 1) - k) / numGroups]], Thin],
     PlotRange → {{xPlotLow, xPlotHi}, {yPlotLow, yPlotHi}},
     PlotRangePadding → Scaled[pltPad], Ticks → Automatic,
     FrameLabel → {lab1, lab2}, ImageSize → pltSize, LabelStyle →
      Directive[FontSize → 14, Black, FontFamily → "Arial"]], {k, numGroups}]];

If[lch == 2,
  Do[
```

```
    hull = ConvexHullMesh[pltPoints[[k]]];
    pltTable[[k, 3]] = HighlightMesh[hull,
      Style[2, Opacity[0.2], Hue[N[((numGroups + 1) - k) / numGroups]]],
      Frame → True, Axes → False, AspectRatio → aRatio,
      PlotRange → {{xPlotLow, xPlotHi}, {yPlotLow, yPlotHi}}, PlotRangePadding →
       Scaled[pltPad], Ticks → Automatic, FrameLabel → {lab1, lab2},
      LabelStyle → Directive[Black, FontSize → 14, FontFamily → "Arial"],
      ImageSize → pltSize], {k, numGroups}]];

If[ptsJoin == 1 && lch == 1, p0 = Show[pltTable[[All, 1]]]];
If[ptsJoin == 2 && lch == 1, p0 = Show[pltTable[[All, 2]], pltTable[[All, 1]]]];
If[ptsJoin == 1 && lch == 2, p0 = Show[pltTable[[All, 3]], pltTable[[All, 1]]]];
If[ptsJoin == 2 && lch == 2,
  p0 = Show[pltTable[[All, 3]], pltTable[[All, 2]], pltTable[[All, 1]]]];

p1 = Labeled[p0, "            PC Score Plot", Top,
    LabelStyle → Directive[FontSize → 18, Bold, FontFamily → "Arial"]];
g1 = Grid[Table[
     {Graphics[{EdgeForm[{Thin, Black}], Hue[N[((numGroups + 1) - j) / numGroups]],
        Disk[]}]}, {j, numGroups}], Frame → False, ItemSize → 0.9];
g2 = Grid[Partition[groupNames, 1], Alignment → Left,
    BaseStyle → {FontFamily → "Arial", FontSize → 13, Italic}];
p2 = Labeled[Text[Grid[{{g1, g2}}, Alignment → Bottom, Frame → True]], "Legend",
    Top, LabelStyle → Directive[Black, FontSize → 18, Bold, FontFamily → "Arial"]];

plt2D = Grid[{{p1, p2}}, BaselinePosition → Top, Alignment → Top]
```

Export current 2 D plot.

In[160]:=
```
filenameout = SystemDialogInput["FileSave"];
Export[filenameout, plt2D, "TIFF", ImageResolution → 150]
```

Label plotted points.

In[•]:=
```
pn1 = p0;
namePoints = tmpPoints;
tempPointsT = Transpose[tmpPoints];
mxY = Max[tempPointsT[[2]]];
mnY = Min[tempPointsT[[2]]];
incY = N[(mxY - mnY) / 15];
Do[namePoints[[i, 2]] = tmpPoints[[i, 2]] - incY, {i, n2}]
nPointsTable = Table[{Text[objNames[[i]], namePoints[[i]], {-1, 0}]}, {i, n2}];
pn2 = Graphics[nPointsTable, Frame → True, AspectRatio → aRatio , Axes → False,
    FrameLabel  → {lab1, lab2}, PlotRangePadding → Scaled[pltPad], BaseStyle →
     Directive[FontSize → 12, FontFamily → "Arial"], ImageSize → pltSize];

p1 = Labeled[
    Show[pn1, pn2, BaseStyle → {FontFamily → "Arial"}], "        2D Data Plot",
    Top, LabelStyle → Directive[FontSize → 18, Bold, FontFamily → "Arial"]];

plt2D = Grid[{{p1, p2}}, BaselinePosition → Top, Alignment → Top]
```

Export current 2 D plot.

In[•]:=
```
filenameout = SystemDialogInput["FileSave"];
Export[filenameout, plt2D, "TIFF", ImageResolution → 150]
```

Create 3 D scatterplot .

Specify 3 D plot options.
You must run this code after you read in the data so it can pick up the proper variable names.

In[162]:=
```
pcNames = Table[StringJoin["PC-", ToString[i]], {i, noAxes}];
Panel[
 Labeled[Column[{Row[{Panel[Labeled[PopupMenu[Dynamic[xAxisName], pcNames],
        "Select variable to be plotted on x-Axis.", Top, LabelStyle →
         Directive[FontSize → 12, Bold, FontFamily → "Arial"]]], "  ",
      Panel[Labeled[PopupMenu[Dynamic[yAxisName], pcNames],
        "Select variable to be plotted on y-Axis.", Top, LabelStyle →
         Directive[FontSize → 12, Bold, FontFamily → "Arial"]]], "  ",
      Panel[Labeled[PopupMenu[Dynamic[zAxisName], pcNames],
        "Select variable to be plotted on z-Axis.", Top,
        LabelStyle → Directive[FontSize → 12, Bold, FontFamily → "Arial"]]]}],
    Row[{
      Panel[Labeled[PopupMenu[Dynamic[pltAspect],
          {1 → "Golden Ratio Plot", 2 → "Square Plot (equi-length axes)",
           3 → "True-Scale Plot (actual axis scales)"}],
        "Enter plot aspect ratio type.", Top, LabelStyle →
         Directive[FontSize → 12, Bold, FontFamily → "Arial"]]], "  ",
      Panel[Labeled[PopupMenu[Dynamic[lch], {1 → "Simple scatterplot",
           2 → "Scatterplot w/ convex hulls"}],
        "Show group domians?", Top, LabelStyle →
         Directive[FontSize → 12, Bold, FontFamily → "Arial"]]], " ",
      Panel[Labeled[PopupMenu[Dynamic[ptsJoin], {1 → "No", 2 → "Yes"}],
        "Join datapoints?", Top,
        LabelStyle → Directive[FontSize → 12, Bold, FontFamily → "Arial"]]]}],
    Row[{Panel[Labeled[InputField[Dynamic[pltSize], FieldSize → 5],
        "Enter plot size value.", Top,
        LabelStyle → Directive[FontSize → 12, Bold, FontFamily → "Arial"]]],
      " ", Panel[Labeled[InputField[Dynamic[pltPad], FieldSize → 5],
        "Enter plot margin padding value.", Top, LabelStyle →
         Directive[FontSize → 12, Bold, FontFamily → "Arial"]]], " ",
      Panel[Labeled[InputField[Dynamic[iconSize3D], FieldSize → 5],
        "Enter plot icon size value.", Top, LabelStyle →
         Directive[FontSize → 12, Bold, FontFamily → "Arial"]]]}]}, Center],
  "3D Plot Options", Top, LabelStyle → Directive[FontSize → 18,
    Bold, FontFamily → "Arial"]]]
pltSize = 500; iconSize3D = 10; pltPad = 0.1; xAxisName = pcNames[[1]];
yAxisName = pcNames[[2]];
zAxisName = pcNames[[3]]; ptsJoin = 1;
dataTrans = 1; pltAspect = 3 ;
lch = 2;
```

Out[163]=

3D Plot Options

Select variable to be plotted on x–Axis.
PC–1

Select variable to be plotted on y–Axis.
PC–2

Select variable to be plotted on z–Axis.
PC–3

Enter plot aspect ratio type.
True–Scale Plot (actual axis scales)

Show group domians?
Scatterplot w/ convex hulls

Join datapoints?
No

Enter plot size value.
500

Enter plot margin padding value.
0.1

Enter plot icon size value.
10

Plot script

In[166]:=
```
Do[If[xAxisName == pcNames[[j]], axis1 = j], {j, noAxes}]
Do[If[yAxisName == pcNames[[j]], axis2 = j], {j, noAxes}]
Do[If[zAxisName == pcNames[[j]], axis3 = j], {j, noAxes}]
iconSize = iconSize3D * 3.0;
If [lch == 1, iconSz3D = 50, iconSz3D = iconSize3D];

groupNames = Union[Group];
numGroups = Length[groupNames];
groupPosns =
  Table[Flatten[Position[Group, groupNames[[i]], 1]], {i, numGroups}];
eScoresT = Transpose[eScores];

xAxis = eScoresT[[axis1]];
yAxis = eScoresT[[axis2]];
zAxis = eScoresT[[axis3]];
lab1 = StringJoin["PC-", ToString[axis1]];
lab2 = StringJoin["PC-", ToString[axis2]];
lab3 = StringJoin["PC-", ToString[axis3]];
maxx = Max[xAxis];
minx = Min[xAxis];
maxy = Max[yAxis];
miny = Min[yAxis];
```

```
maxz = Max[zAxis]; minz = Min[zAxis];

If[pltAspect == 1 || pltAspect == 3,
  xPlotLow = minx; xPlotHi = maxx; yPlotLow = miny; yPlotHi = maxy];
If[pltAspect == 2,
  If[minx > miny,
   xPlotLow = miny; yPlotLow = miny,
   xPlotLow = minx; yPlotLow = minx ]];
If[pltAspect == 2,
  If[maxx < maxy,
   xPlotHi = maxy; yPlotHi = maxy,
   xPlotHi = maxx; yPlotHi = maxx]];

If[pltAspect == 1, bRatio = {1.61803, 1, 1}];
If[pltAspect == 2, bRatio = {1, 1, 1}];
If[pltAspect == 3, bRatio = Automatic];

points3 = Transpose[List[xAxis, yAxis, zAxis]];
gp1 = groupPosns;
h = Table[0, {n2}];
Do[
 tmp = gp1[[i]];
 itr = Length[tmp];
 Do[h[[tmp[[j]]]] = Hue[N[((numGroups + 1) - i) / numGroups]], {j, itr}],
 {i, numGroups}]

If[lch == 1 && pltAspect == 3,
  pltPoints = Table[{h[[i]], Sphere[points3[[i]], iconSz3D / 55]}, {i, n2}],
  pltPoints =
   Table[{h[[i]], AbsolutePointSize[iconSz3D], Point[points3[[i]]]}, {i, n2}]];
p0 = Graphics3D[pltPoints, Axes → True, Boxed → True,
   PlotRangePadding → Scaled[pltPad],
   LabelStyle → Directive[FontSize → 12, Black, FontFamily → "Arial"],
   AxesLabel → {lab1, lab2, lab3}, ImageSize → pltSize, BoxRatios → bRatio];

If[ptsJoin == 2,
  pltLineTable = Table[" ", {numGroups}];
  gpPoints = Table[points3[[groupPosns[[j]]]], {j, numGroups}];
  Do[
   pltLineTable[[k]] =
    Graphics3D[{Hue[N[((numGroups + 1) - k) / numGroups]], Line[gpPoints[[k]]]},
     Axes → True, Boxed → True, PlotRangePadding → Scaled[pltPad],
     LabelStyle → Directive[FontSize → 12, Black, FontFamily → "Arial"],
     AxesLabel → {lab1, lab2, lab3}, ImageSize → pltSize,
     BoxRatios → bRatio, ViewPoint -> {xax, yax, zax}], {k, numGroups}];
  pTmp = Graphics3D[pltPoints, Axes → True, Boxed → True,
    PlotRangePadding → Scaled[pltPad],
```

```
    LabelStyle → Directive[FontSize → 12, Black, FontFamily → "Arial"],
    AxesLabel → {lab1, lab2, lab3}, ImageSize → pltSize,
    BoxRatios → bRatio, ViewPoint -> {xax, yax, zax}];
  p0 = Show[{pltLineTable, pTmp}]];

If[lch == 2,
  pltMeshTable = Table[" ", {numGroups}];
  gpPoints = Table[points3[[groupPosns[[j]]]], {j, numGroups}];
  Do[
   {n3, m3} = Dimensions[gpPoints[[k]]];
   pltPoints = Table[{Hue[N[((numGroups + 1) - k) / numGroups]],
      AbsolutePointSize[iconSize3D], Point[gpPoints[[k, i]]]}, {i, n3}];
   pTmp = Graphics3D[pltPoints, Axes → True, Boxed → True,
     PlotRangePadding → Scaled[pltPad],
     LabelStyle → Directive[FontSize → 12, Black, FontFamily → "Arial"],
     AxesLabel → {lab1, lab2, lab3}, ImageSize → pltSize,
     BoxRatios → bRatio, ViewPoint -> {xax, yax, zax}];
   cHull3D = ConvexHullMesh[gpPoints[[k]], BaseStyle → {EdgeForm[]},
     Boxed → True, Axes → True, PlotRangePadding → Scaled[pltPad],
     LabelStyle → Directive[FontSize → 12, Black, FontFamily → "Arial"],
     AxesLabel → {lab1, lab2, lab3}, ImageSize → pltSize,
     BoxRatios → bRatio, ViewPoint -> {xax, yax, zax}];
   pltMeshTable[[k]] = Show[{HighlightMesh[cHull3D, Style[2, Opacity[0.2],
        Hue[N[((numGroups + 1) - k) / numGroups]]]], p0}], {k, numGroups}];
  p0 = Show[pltMeshTable]];

p1 = Labeled[p0, "          PC Score Plot", Top,
   LabelStyle → Directive[FontSize → 18, Bold, FontFamily → "Arial"]];
If[lch == 1 && pltAspect == 3, g1 = Table[Graphics[
     {Inset[Graphics3D[{Hue[N[((numGroups + 1) - i) / numGroups]], Sphere[]},
        Boxed → False]]}], {i, numGroups}],
  g1 = Table[Graphics[{Inset[Graphics[{Hue[N[((numGroups + 1) - i) / numGroups]],
         Disk[{0, 0}, Scaled[0.3]]}]]}], {i, numGroups}]];
g2 = Labeled[Text[Grid[Transpose[Partition[Join[g1, groupNames], numGroups]],
     ItemSize → {{Scaled[0.02], Automatic}}, Alignment → Left, BaseStyle →
      {FontSize → 12, FontFamily → "Arial", Italic}, Frame → True]], "Legend",
   Top, LabelStyle → Directive[FontSize → 14, Bold, FontFamily → "Arial"]];

plt3D = Grid[{{p1, g2}}, BaselinePosition → Top, Alignment → Top]
```

Adjust orientation of 3 D plot (if necessary).
You must replot the data to activate the changes. These changes will be able to be exported using the script below.

In[20]:=
```
Panel[
 Labeled[Row[{Labeled[Slider[Dynamic[xax], {-10, 10}, Appearance → "Labeled"],
      "x-Axis Viewpoint", Top,
      LabelStyle → Directive[FontSize → 10, Bold, FontFamily → "Arial"]] ×
     Labeled[Slider[Dynamic[yax], {-10, 10}, Appearance → "Labeled"],
      "y-Axis Viewpoint", Top,
      LabelStyle → Directive[FontSize → 10, Bold, FontFamily → "Arial"]] ×
     Labeled[Slider[Dynamic[zax], {-10, 10}, Appearance → "Labeled"],
      "z-Axis Viewpoint", Top,
      LabelStyle → Directive[FontSize → 10, Bold, FontFamily → "Arial"]]}],
   "3D Plot Orientation Controls", Top, LabelStyle →
    Directive[FontSize → 14, Bold, FontFamily → "Ariel"]]]
xax = 2.5; yax = -2.5; zax = 2.5;
```

Out[20]=

3D Plot Orientation Controls

x–Axis Viewpoint 2.5

y–Axis Viewpoint −2.5

z–Axis Viewpoint 2.5

Export current 3 D plot.

In[199]:=
```
filenameout = SystemDialogInput["FileSave"];
Export[filenameout, plt3D, "TIFF", ImageResolution → 150]
```

Export PCA results (optional).

Export eigenvalue table (optional).

In[201]:=
```
filenameout = SystemDialogInput["FileSave"];
Export[filenameout, t2, "CSV", "TextDelimiters" → ""]
```

Export eigenvector table (optional).

In[ ]:=
```
filenameout = SystemDialogInput["FileSave"];
Export[filenameout, eVecs, "CSV", "TextDelimiters" → ""]
```

Export PCA scores (optional).

In[203]:=
```
adata = Table[" ", {n2 + 1}, {noAxes + 2}];
adata[[1, 1]] = "Object";
adata[[1, 2]] = "Group";
Do[adata[[i + 1, 1]] = objNames[[i]], {i, n2}]
Do[adata[[i + 1, 2]] = Group[[i]], {i, n2}]
Do[
 adata[[1, j + 2]] = StringJoin["PC-", ToString[j]], {j, 1, noAxes}]
Do[adata[[i + 1, j + 2]] = eScores[[i, j]], {i, n2}, {j, noAxes}]

filenameout = SystemDialogInput["FileSave"];
Export[filenameout, adata, "CSV", "TextDelimiters" → ""]
```

Modelling Section (optional).

Calculate evenly spaced models along a set of axes (optional).

Specify no. of model axes and no. of models to be calcuated per axis

In[22]:=
```
Panel[Labeled[Row[{Panel[Labeled[InputField[Dynamic[modelAxes], FieldSize → 5],
      "Enter no. of axes to be modeled.", Top,
      LabelStyle → Directive[FontSize → 12, FontFamily → "Arial"]]], , ,
    Panel[Labeled[InputField[Dynamic[modelsPerAxis], FieldSize → 5],
      "Enter no. models to be plotted per axis.", Top,
      LabelStyle → Directive[FontSize → 12, FontFamily → "Arial"]]]}],
  "Along-Axis Modeling Parameters", Top, LabelStyle →
   Directive[FontSize → 16, Bold, FontFamily → "Arial"]]]
modelsPerAxis = 5; modelAxes = 3;
```

Out[22]=

Along-Axis Modeling Parameters

Enter no. of axes to be modeled.	Enter no. models to be plotted per axis.
3	5

Calculate along – axis PCA model coordinates.

```
In[•]:= modTable = Table[0.0, {modelAxes}, {modelsPerAxis}, {modelAxes}];
Do[
 Do[
  Do[modTable[[k, j, i]] = Mean[Take[eScores[[All, i]]]], {j, modelsPerAxis}],
  {i, modelAxes}], {k, modelAxes}]
eScoresT = Transpose[eScores];
Do[
 xMax = Max[eScoresT[[k]]];
 xMin = Min[eScoresT[[k]]];
 xRng = xMax - xMin;
 xInt = xRng / (modelsPerAxis - 1);
 xBase = xMin - xInt;
 Do[modTable[[k, i, k]] = xBase + (xInt * i), {i, modelsPerAxis}], {k, modelAxes}]
modSet = 1;
```

Calculate evenly spaced models across a planar subspace (optional).

Specify subspace modelling options.

In[24]:=
```
Panel[
 Labeled[Column[{Row[{Panel[Labeled[PopupMenu[Dynamic[xAxisName], pcNames],
        "Select principal component to be modeled on x-axis.", Top,
        LabelStyle → Directive[FontSize → 12, FontFamily → "Arial"]]], , ,
      Panel[Labeled[InputField[Dynamic[xModelNum], FieldSize → 5],
        "Enter no. of models along the x-axis.", Top,
        LabelStyle → Directive[FontSize → 12, FontFamily → "Arial"]]]}],
    Row[{Panel[Labeled[PopupMenu[Dynamic[yAxisName], pcNames],
        "Select principal component to be modeled on y-axis.", Top,
        LabelStyle → Directive[FontSize → 12, FontFamily → "Arial"]]], , ,
      Panel[Labeled[InputField[Dynamic[yModelNum], FieldSize → 5],
        "Enter no. of models along the y-axis.", Top,
        LabelStyle → Directive[FontSize → 12, FontFamily → "Arial"]]]}]}],
  "Subspace Modeling Parameters", Top, LabelStyle →
   Directive[FontSize → 18, Bold, FontFamily → "Arial"]]]
xAxisName = pcNames[[1]];
yAxisName = pcNames[[2]];
xModelNum = 5;
yModelNum = 4;
```

Out[24]=

Subspace Modeling Parameters

Select principal component to be modeled on x–axis.<br>PopupMenu[PC–1, Table[PC– <> ToString[i], {i, mvecs}]]	Enter no. of models along the x–axis.<br>5
Select principal component to be modeled on y–axis.<br>PopupMenu[PC–2, Table[PC– <> ToString[i], {i, mvecs}]]	Enter no. of models along the y–axis.<br>4

Perform subspace model location calculations

In[•]:=
```
modTable = Table[0.0, {yModelNum}, {xModelNum}, {2}];
eScoresT = Transpose[eScores];
Do[If[xAxisName == pcNames[[j]], axis1 = j], {j, noAxes}]
Do[If[yAxisName == pcNames[[j]], axis2 = j], {j, noAxes}]
xMax = Max[eScoresT[[axis1]]];
xMin = Min[eScoresT[[axis1]]];
xRng = xMax - xMin;
xInt = xRng / (xModelNum - 1);
yMax = Max[eScoresT[[axis2]]];
yMin = Min[eScoresT[[axis2]]];
yRng = yMax - yMin;
yInt = yRng / (yModelNum - 1);

xBase = xMin - xInt;
yBase = yMin - yInt;
Do[
 yVal = yBase + (yInt * j);
 Do[modTable[[j, i, 1]] = xBase + (xInt * i);
  modTable[[j, i, 2]] = yVal, {i, xModelNum}], {j, yModelNum}]
modSet = 2;
```

Import external set of model coordinates
(total must match along – axis model specifications [above] and contain the complete variable set).

```
filenamein = SystemDialogInput["FileOpen"];
modTable = Import[filenamein, "CSV"];
```

Plot model coordinates in the PC space.

Specify 2 D plot options.
You must run this code after you read in the data so it can pick up the proper variable names.

In[•]:=
```
pcNames = Table[StringJoin["PC-", ToString[i]], {i, noAxes}];
Panel[Labeled[Column[
   {Panel[Labeled[RadioButtonBar[Dynamic[modSet], {1 → "Along-axis Models",
        2 → "Subspace models"}], "Enter type of model set.", Top,
      LabelStyle → Directive[FontSize → 12, FontFamily → "Arial"]]],
    Row[{Panel[Labeled[PopupMenu[Dynamic[xAxisName], pcNames],
        "Select variable to be plotted on x-Axis.", Top,
        LabelStyle → Directive[FontSize → 12, FontFamily → "Arial"]]], "  ",
      Panel[Labeled[PopupMenu[Dynamic[yAxisName], pcNames],
        "Select variable to be plotted on y-Axis.", Top,
        LabelStyle → Directive[FontSize → 12, FontFamily → "Arial"]]]}],
    Row[{
      Panel[Labeled[PopupMenu[Dynamic[pltAspect],
         {1 → "Golden Ratio Plot", 2 → "Square Plot (equi-length axes)",
          3 → "True-Scale Plot (actual axis scales)"}],
        "Enter plot aspect ratio type.", Top, LabelStyle →
         Directive[FontSize → 12, Bold, FontFamily → "Arial"]]], "  ",
      Panel[Labeled[PopupMenu[Dynamic[lch], {1 → "Simple scatterplot",
          2 → "Scatterplot w/ convex hulls"}],
        "Show group domians?", Top, LabelStyle →
         Directive[FontSize → 12, Bold, FontFamily → "Arial"]]], "  ",
      Panel[Labeled[PopupMenu[Dynamic[ptsJoin], {1 → "No", 2 → "Yes"}],
        "Join datapoints?", Top,
        LabelStyle → Directive[FontSize → 12, Bold, FontFamily → "Arial"]]]}],
    Row[{Panel[Labeled[InputField[Dynamic[pltSz], FieldSize → 5],
        "Enter plot size value.", Top,
        LabelStyle → Directive[FontSize → 12, FontFamily → "Arial"]]], "  ",
      Panel[Labeled[InputField[Dynamic[iconSizeMods], FieldSize → 5],
        "Enter plot icon size value.", Top,
        LabelStyle → Directive[FontSize → 12, FontFamily → "Arial"]]], "  ",
      Panel[Labeled[InputField[Dynamic[pltPad], FieldSize → 5],
        "Enter plot margin padding value.", Top,
        LabelStyle → Directive[FontSize → 12, FontFamily → "Arial"]]]}]},
   Center], "Model Coordinate Options", Top, LabelStyle →
   Directive[FontSize → 18, Bold, FontFamily → "Arial"]]]
pltSize = 500; iconSizeMods = 0.03; pltPad = 0.1;
xAxisName = pcNames[[1]];
pltSz = 500; yAxisName = pcNames[[2]]; ptsJoin = 1;
dataTrans = 1; pltAspect = 1 ;
lch = 2;
```

Out[•]=

Model Coordinate Options

Enter type of model set.
◉ Along–axis Models ◯ Subspace models

Select variable to be plotted on x–Axis. PC–1

Select variable to be plotted on y–Axis. PC–2

Enter plot aspect ratio type.
True–Scale Plot (actual axis scales)

Show group domians?
Scatterplot w/ convex hulls

Join datapoints?
No

Enter plot size value. pltSz

Enter plot icon size value. iconSiz⋱ eMod⋱ s

Enter plot margin padding value. 0.1

Plot model coordinates in space of a PCA plane (optional).

In[•]:=
```
modpts1 = Table[0.0, {modelsPerAxis}, {2}];
modpts2 = Table[0.0, {modelsPerAxis}, {2}];
Do[If[xAxisName == pcNames[[j]], axis1 = j], {j, noAxes}]
Do[If[yAxisName == pcNames[[j]], axis2 = j], {j, noAxes}]

If[modSet == 1,
  If[meanTrans == 1,
   modpts1 = Take[modTable[[axis1, All, {axis1, axis2}]]];
   modpts2 = Take[modTable[[axis2, All, {axis1, axis2}]]];
   modpts = Partition[Flatten[modpts], 2]];
  If[meanTrans == 2,
   modpts1[[All, 1]] = Take[modTable[[axis1, All, axis1]]];
   modpts2[[All, 2]] = Take[modTable[[axis2, All, axis2]]]];
  modpts = Partition[Flatten[Append[modpts1, modpts2]], 2]];
modpts = Partition[modpts, modelsPerAxis];

If[modSet == 2, modpts = Partition[Flatten[modTable], 2]];
axisPlots = Table[" ", {2}];

groupNames = Union[Group];
numGroups = Length[groupNames];
groupPosns =
```

```
  Table[Flatten[Position[Group, groupNames[[i]], 1]], {i, numGroups}];
eScoresT = Transpose[eScores];

xAxis = eScoresT[[axis1]]; yAxis = eScoresT[[axis2]];
lab1 = StringJoin[{"Principal Component ", ToString[axis1]},
   {" (Var. =", ToString[t3[[axis1 + 1, 3]]]}, {"%)"}];
lab2 = StringJoin[{"Principal Component ", ToString[axis2]},
   {" (Var. =", ToString[t3[[axis2 + 1, 3]]]}, {"%)"}];
maxx =
 Max[
  xAxis];
minx = Min[xAxis];
maxy = Max[yAxis];
miny = Min[yAxis];

If[pltAspect == 1 || pltAspect == 3,
  xPlotLow = minx; xPlotHi = maxx; yPlotLow = miny; yPlotHi = maxy];
If[pltAspect == 2,
  If[minx > miny,
   xPlotLow = miny; yPlotLow = miny,
   xPlotLow = minx; yPlotLow = minx ]];
If[pltAspect == 2,
  If[maxx < maxy,
   xPlotHi = maxy; yPlotHi = maxy,
   xPlotHi = maxx; yPlotHi = maxx]];
If[pltAspect == 1, aRatio = 1 / N[GoldenRatio]];
If[pltAspect == 2, aRatio = 1];
If[pltAspect == 3, aRatio = Automatic];

tmpPoints = Transpose[List[xAxis, yAxis]];
pltPoints = Table[tmpPoints[[groupPosns[[j]]]], {j, numGroups}];
iconList = Flatten[Table[
    {Graphics[{EdgeForm[{Thickness[0.005], Black}],
       Hue[N[((numGroups + 1) - j) / numGroups]],
       Disk[{0, 0}, Scaled[iconSizeMods]]}]}, {j, numGroups}]];

If[lch == 1 || ptsJoin == 1,
 Do[
  pltTable[[k, 1]] = pTmp =
    ListPlot[pltPoints[[k]], AspectRatio → aRatio, Frame → True, Joined → False,
     Axes → False, PlotRange → {{xPlotLow, xPlotHi}, {yPlotLow, yPlotHi}},
     PlotRangePadding → Scaled[pltPad], Ticks → Automatic, FrameLabel →
      {lab1, lab2}, PlotMarkers → iconList[[k]], ImageSize → pltSize, LabelStyle →
      Directive[FontSize → 14, Black, FontFamily → "Arial"]], {k, kg}],
 Do[
  pltTable[[k, 1]] =
   ListPlot[pltPoints[[k]], Frame → True, Axes → False, AspectRatio → aRatio,
```

```
    PlotRange → {{xPlotLow, xPlotHi}, {yPlotLow, yPlotHi}}, PlotRangePadding →
     Scaled[pltPad], Ticks → Automatic, FrameLabel → {lab1, lab2},
    LabelStyle → Directive[Black, FontSize → 14, FontFamily → "Arial"],
    ImageSize → pltSize, PlotStyle →
     Directive[Disk[], Hue[N[((+1) - k) / kg]], EdgeForm[{Thickness[1.0], Black}],
      PointSize[Scaled[iconSize - 0.009]]]], {k, kg}]]

If[ptsJoin == 2,
  Do[
   pltTable[[k, 2]] = ListLinePlot[pltPoints[[k]],
     AspectRatio → aRatio, Frame → True, Joined → True, Axes → False,
     PlotStyle → Directive[Hue[N[((+1) - k) / kg]], Thin],
     PlotRange → {{xPlotLow, xPlotHi}, {yPlotLow, yPlotHi}},
     PlotRangePadding → Scaled[pltPad], Ticks → Automatic,
     FrameLabel → {lab1, lab2}, ImageSize → pltSize, LabelStyle →
      Directive[FontSize → 14, Black, FontFamily → "Arial"]], {k, kg}]];

If[lch == 2,
  Do[
   hull = ConvexHullMesh[pltPoints[[k]]];
   pltTable[[k, 3]] =
    HighlightMesh[hull, Style[2, Opacity[0.2], Hue[N[((+1) - k) / kg]]],
     Frame → True, Axes → False, AspectRatio → aRatio,
     PlotRange → {{xPlotLow, xPlotHi}, {yPlotLow, yPlotHi}}, PlotRangePadding →
      Scaled[pltPad], Ticks → Automatic, FrameLabel → {lab1, lab2},
     LabelStyle → Directive[Black, FontSize → 14, FontFamily → "Arial"],
     ImageSize → pltSize], {k, kg}]];

If[ptsJoin == 1 && lch == 1, p0 = Show[pltTable[[All, 1]]]];
If[ptsJoin == 2 && lch == 1, p0 = Show[pltTable[[All, 2]], pltTable[[All, 1]]]];
If[ptsJoin == 1 && lch == 2, p0 = Show[pltTable[[All, 3]], pltTable[[All, 1]]]];
If[ptsJoin == 2 && lch == 2,
  p0 = Show[pltTable[[All, 3]], pltTable[[All, 2]], pltTable[[All, 1]]]];

iconList = Flatten[Table[
    {Graphics[{EdgeForm[{Thin, Black}],
       Black, Disk[{0, 0}, Scaled[iconSizeMods / 2]]}]}, {j, 1}]];
If[modSet == 1,
  Do[
   axisPlots[[i]] = ListPlot[modpts[[i]], AspectRatio → aRatio,
     Frame → True, Joined → True, Axes → False, PlotRange → All,
     PlotStyle → Directive[Black, AbsoluteThickness[0.8]],
     PlotRangePadding → Scaled[pltPad], Ticks → Automatic,
     FrameLabel → {lab1, lab2}, PlotMarkers → iconList], {i, 2}];
  p4 = Show[axisPlots]];
If[modSet == 2,
  subSpacePlot = ListPlot[modpts, AspectRatio → aRatio,
```

```
      Frame → True, Joined → False, Axes → False, PlotRange → All,
      PlotStyle → Directive[Black, AbsoluteThickness[0.8]],
      PlotRangePadding → Scaled[pltPad], Ticks → Automatic,
      FrameLabel → {lab1, lab2}, PlotMarkers → iconList];
   p4 = subSpacePlot];
p1 = Labeled[Show[p0, p4], "           PC Score Plot", Top,
    LabelStyle → Directive[FontSize → 18, Bold, FontFamily → "Arial"]];
g1 = Grid[Table[
      {Graphics[{EdgeForm[{Thin, Black}], Hue[N[((numGroups + 1) - j) / numGroups]],
         Disk[]}]}, {j, numGroups}], Frame → False, ItemSize → 0.9];
g2 = Grid[Partition[groupNames, 1], Alignment → Left,
    BaseStyle → {FontFamily → "Arial", FontSize → 13, Italic}];
p2 = Labeled[Text[Grid[{{g1, g2}}, Alignment → Bottom, Frame → True]], "Legend",
    Top, LabelStyle → Directive[Black, FontSize → 18, Bold, FontFamily → "Arial"]];

plt2D = Grid[{{p1, p2}}, BaselinePosition → Top, Alignment → Top]
```

Export plot (optional).

```
In[•]:= filenameout = SystemDialogInput["FileSave"];
Export[filenameout, plt2D, "TIFF", ImageResolution → 150]
```

Export PCA model coordinates (optional).

```
In[•]:= If[modSet == 1, partNum = modelAxes, partNum = 2];
If[modSet == 2, modTable = Reverse[modTable]];
modelsOut = Partition[Flatten[modTable], partNum];
filenameout = SystemDialogInput["FileSave"];
Export[filenameout, modelsOut, "CSV", "TextDelimiters" → ""]
```

Data – Projection Module (optional)

This routine allows you to import data for objects that were not used to contruct the PCA space and project them into the PCA space with full support for data – visualization graphics

Read in projection datafile & partition into datasets.

In[•]:=
```
filenamein = SystemDialogInput["FileOpen"];
x3 = Import[filenamein, "CSV"];
filenamein

{nProj, mProj} = Dimensions[x3];
varNames = Flatten[Take[x3, 1]];
x4 = Drop[x3, 1];
varNames = Drop[varNames, 1];
varNames = Drop[varNames, 1];

projObjNames = Flatten[Take[x4, nProj - 1, 1]];
x4 = Drop[x4, 0, 1];

projGroup = Flatten[Take[x4, nProj - 1, 1]];
x4 = Drop[x4, 0, 1];
numProjGroups = Length[Union[projGroup]];

{n4, m4} = Dimensions[x4];
Print["No. of objects: ", n4]
Print["No. of variables: ", m4]
```

Out[•]=
```
/Users/n.macleod/Desktop/Archaeological Types/Ford's Houses/Figure
  1/Images (Houses + Piers)/Ford's Houses (Figs 2 & 3 Means).csv
```

```
No. of objects: 2

No. of variables: 8300
```

Project into PCA space and combine with original data.

In[•]:=
```
If[meanTrans == 2, Do[x4[[i]] = x4[[i]] - mVec, {i, n4}]];
If[logTrans == 2, x2 = N[Log10[x4]]];
If[stdTrans == 2, x2 = Standardize[x4]];
If[shiftTrans == 2, x4 = x4 + knsnt];

projEScores = x4.eVecs;
projEScoresT = Transpose[projEScores];
combGroups = Join[Group, projGroup];
combGpNames = Union[combGroups];
combEScores = Join[eScores, projEScores];
combNumGroups = numGroups + numProjGroups;
combObjNames = Join[objNames, projObjNames];
combN = n2 + n4;
combGpNames = Union[combGroups];
numProjGroups = Length[combGpNames];
combProjPosns =
  Table[Flatten[Position[combGroups, combGpNames[[i]], 1]], {i, combNumGroups}];
```

Projection Plot section

Plot single – axis histogram.

Specify single – variable plot (histogram) options.

In[26]:=
```
pcNames = Table[StringJoin["PC-", ToString[i]], {i, noAxes}];
Panel[Labeled[Column[{
    Row[{Panel[Labeled[PopupMenu[Dynamic[axisName], pcNames],
        "Select component to be plotted.", Top,
        LabelStyle → Directive[FontSize → 12, Bold, FontFamily → "Arial"]]],
      "  ", Panel[Labeled[PopupMenu[Dynamic[histType],
         {1 → "Stacked", 2 → "Overlapped"}], "Select histogram type.", Top,
        LabelStyle → Directive[FontSize → 12, Bold, FontFamily → "Arial"]]]}],
    Row[{Panel[Labeled[InputField[Dynamic[noBins], FieldSize → 5],
        "Enter no. of histogram bins.", Top, LabelStyle →
         Directive[FontSize → 12, Bold, FontFamily → "Arial"]]], "  ",
      Panel[Labeled[InputField[Dynamic[hSize], FieldSize → 5],
        "Enter histogram plot size.", Top,
        LabelStyle → Directive[FontSize → 12, Bold, FontFamily → "Arial"]]]}]},
   Center], "Single Axis (Histogram) Plot Options", Top,
  LabelStyle → Directive[FontSize → 16, Bold, FontFamily → "Arial"]]]
axisName = pcNames[[1]]; noBins = 15; hSize = 500; histType = 1;
```

```
··· Table: Iterator {i, mvecs} does not have appropriate bounds.
··· Table: Iterator {i, mvecs} does not have appropriate bounds.
```

Out[27]=

Single Axis (Histogram) Plot Options

Select component to be plotted.	Select histogram type.
PopupMenu[PC−1, Table[PC−<>ToString[i], {i, mvecs}]]	Stacked

Enter no. of histogram bins.	Enter histogram plot size.
15	500

Construct and display histogram plot .

In[•]:=
```
Do[If[axisName == pcNames[[j]], axis = j], {j, noAxes}];
hScores = Flatten[Take[combEScores, All, {axis}]];
If[histType == 1, htype = "Stacked", htype = "Overlapped"];
projGpNames = Union[combGroups];
combProjPosns =
  Table[Flatten[Position[combGroups, projGpNames[[i]], 1]], {i, combNumGroups}];
hPltPoints = Table[hScores[[combProjPosns[[j]]]], {j, combNumGroups}];
hueList =
  Table[Hue[N[((combNumGroups + 1) - j) / combNumGroups]], {j, combNumGroups}];
h2 = Labeled[Histogram[hPltPoints, noBins,
   ChartStyle → {hueList}, ChartLayout → htype,
   LabelStyle → Directive[FontSize → 12, Black, FontFamily → "Arial"], AxesLabel →
    {"PC Score", "Frequency"}, ImageSize → hSize, ChartLegends → projGpNames],
  StringJoin["Principal Component ", ToString[axis]], Top,
  LabelStyle → Directive[FontSize → 16, Bold, FontFamily → "Arial"]]
```

Export data projection histogram.

```
filenameout = SystemDialogInput["FileSave"];
Export[filenameout, projh1, "TIFF"]
```

```
/Users/nm/Projects/Storage/MacLeod/Manuscripts/01
  Developing/Wolf Dimorphism/Wolf Data/Mandibles/Data &
  Results 2/CVA Results/CV-1 Histogram (w: Unknowns).tif
```

Create 2 D scatterplot .

Specify 2 D plot options.
You must run this code after you read in the data so it can pick up the proper variable names.

In[29]:=
```
pcNames = Table[StringJoin["PC-", ToString[i]], {i, noAxes}];
Panel[
 Labeled[Column[{Row[{Panel[Labeled[PopupMenu[Dynamic[xAxisName], pcNames],
        "Select variable to be plotted on x-Axis.", Top, LabelStyle →
         Directive[FontSize → 12, Bold, FontFamily → "Arial"]]], "  ",
      Panel[Labeled[PopupMenu[Dynamic[yAxisName], pcNames],
        "Select variable to be plotted on y-Axis.", Top,
        LabelStyle → Directive[FontSize → 12, Bold, FontFamily → "Arial"]]]}],
    Row[{
      Panel[Labeled[PopupMenu[Dynamic[pltAspect],
          {1 → "Golden Ratio Plot", 2 → "Square Plot (equi-length axes)",
           3 → "True-Scale Plot (actual axis scales)"}],
        "Enter plot aspect ratio type.", Top, LabelStyle →
         Directive[FontSize → 12, Bold, FontFamily → "Arial"]]], "  ",
      Panel[Labeled[PopupMenu[Dynamic[lch], {1 → "Simple scatterplot",
           2 → "Scatterplot w/ convex hulls"}],
        "Show group domians?", Top, LabelStyle →
         Directive[FontSize → 12, Bold, FontFamily → "Arial"]]], "  ",
      Panel[Labeled[PopupMenu[Dynamic[ptsJoin], {1 → "No", 2 → "Yes"}],
        "Join datapoints?", Top,
        LabelStyle → Directive[FontSize → 12, Bold, FontFamily → "Arial"]]]}],
    Row[{Panel[Labeled[InputField[Dynamic[pltSize], FieldSize → 10],
        "Enter plot size value.", Top,
        LabelStyle → Directive[FontSize → 12, Bold, FontFamily → "Arial"]]],
      "  ", Panel[Labeled[InputField[Dynamic[pltPad], FieldSize → 10],
        "Enter plot margin padding value.", Top, LabelStyle →
         Directive[FontSize → 12, Bold, FontFamily → "Arial"]]], "  ",
      Panel[Labeled[InputField[Dynamic[iconSize], FieldSize → 10],
        "Enter plot icon size value.", Top, LabelStyle →
         Directive[FontSize → 12, Bold, FontFamily → "Arial"]]]}]}, Center],
  "2D Plot Options", Top, LabelStyle → Directive[FontSize → 18,
    Bold, FontFamily → "Arial"]]]
pltSize = 500; iconSize = 0.03; pltPad = 0.1; xAxisName = pcNames[[1]];
yAxisName = pcNames[[2]];
 ptsJoin = 1; dataTrans = 1;  pltAspect = 1 ; lch = 2;
```

Table: Iterator {i, mvecs} does not have appropriate bounds.

Table: Iterator {i, mvecs} does not have appropriate bounds.

Out[30]=

2D Plot Options

Select variable to be plotted on x–Axis.
PopupMenu[PC–1, Table[PC– <> ToString[i], {i, mvecs}]]

Select variable to be plotted on y–Axis.
PopupMenu[PC–2, Table[PC– <> ToString[i], {i, mvecs}]]

Enter plot aspect ratio type.
True–Scale Plot (actual axis scales)

Show group domians?
Scatterplot w/ convex hulls

Join datapoints?
No

Enter plot size value.
500

Enter plot margin padding value.
0.1

Enter plot icon size value.
30.

Plot script

In[∘]:=

```
projPltTable = Table[" ", {combNumGroups}, {3}];
Do[If[xAxisName == pcNames[[j]], axis1 = j], {j, noAxes}];
Do[If[yAxisName == pcNames[[j]], axis2 = j], {j, noAxes}];

combGpNames = Union[combGroups];
numProjGroups = Length[combGpNames];
combProjPosns =
  Table[Flatten[Position[combGroups, combGpNames[[i]], 1]], {i, combNumGroups}];

combEScoresT = Transpose[combEScores];
xAxis = combEScoresT[[axis1]]; yAxis = combEScoresT[[axis2]];
lab1 = StringJoin[{"Principal Component ", ToString[axis1]},
   {" (Var. =", ToString[t3[[axis1 + 1, 3]]]}, {"%)"}];
lab2 = StringJoin[{"Principal Component ", ToString[axis2]},
   {" (Var. =", ToString[t3[[axis2 + 1, 3]]]}, {"%)"}];
maxx =
 Max[
  xAxis];
minx = Min[xAxis];
maxy = Max[yAxis];
miny = Min[yAxis];

If[pltAspect == 1 || pltAspect == 3,
  xPlotLow = minx; xPlotHi = maxx; yPlotLow = miny; yPlotHi = maxy];
```

```
If[pltAspect == 2,
  If[minx > miny,
   xPlotLow = miny; yPlotLow = miny,
   xPlotLow = minx; yPlotLow = minx ]];
If[pltAspect == 2,
  If[maxx < maxy,
   xPlotHi = maxy; yPlotHi = maxy,
   xPlotHi = maxx; yPlotHi = maxx]];
If[pltAspect == 1, aRatio = 1 / N[GoldenRatio]];
If[pltAspect == 2, aRatio = 1];
If[pltAspect == 3, aRatio = Automatic];

projTmpPoints = Transpose[List[xAxis, yAxis]];
projPltPoints =
  Table[projTmpPoints[[combProjPosns[[j]]]], {j, combNumGroups}];
iconList = Flatten[Table[
    {Graphics[
      {EdgeForm[{Thin, Black}], Hue[N[((combNumGroups + 1) - j) / combNumGroups]],
       Disk[{0, 0}, Scaled[iconSize]]}]}, {j, combNumGroups}]];

If[lch == 1 || ptsJoin == 1,
  Do[
   projPltTable[[k, 1]] = ListPlot[projPltPoints[[k]],
     AspectRatio → aRatio, Frame → True, Joined → False, Axes → False,
     PlotRange → {{xPlotLow, xPlotHi}, {yPlotLow, yPlotHi}},
     PlotRangePadding → Scaled[pltPad], Ticks → Automatic,
     FrameLabel → {lab1, lab2}, PlotMarkers → iconList[[k]],
     ImageSize → pltSize, LabelStyle → Directive[FontSize → 14,
       Black, FontFamily → "Arial"]], {k, combNumGroups}],
  Do[
   projPltTable[[k, 1]] =
    ListPlot[projPltPoints[[k]], Frame → True, Axes → False, AspectRatio → aRatio,
     PlotRange → {{xPlotLow, xPlotHi}, {yPlotLow, yPlotHi}}, PlotRangePadding →
      Scaled[pltPad], Ticks → Automatic, FrameLabel → {lab1, lab2},
     LabelStyle → Directive[Black, FontSize → 14, FontFamily → "Arial"],
     ImageSize → pltSize, PlotStyle → Directive[Disk[], Hue[
        N[((numGroups + 1) - k) / numGroups]], EdgeForm[{Thickness[1.0], Black}],
       PointSize[Scaled[iconSize - 0.009]]]], {k, combNumGroups}]];

If[ptsJoin == 2,
  Do[
   projPltTable[[k, 2]] = ListLinePlot[projPltPoints[[k]],
     AspectRatio → aRatio, Frame → True, Joined → True, Axes → False, PlotStyle →
      Directive[Hue[N[((numProjGroups + 1) - k) / numProjGroups]], Thin],
     PlotRange → {{xPlotLow, xPlotHi}, {yPlotLow, yPlotHi}},
     PlotRangePadding → Scaled[pltPad], Ticks → Automatic,
     FrameLabel → {lab1, lab2}, ImageSize → pltSize, LabelStyle → Directive[
```

```
        FontSize → 14, Black, FontFamily → "Arial"]], {k, combNumGroups}]];

If[lch == 2,
  Do[
   hull = ConvexHullMesh[projPltPoints[[k]]];
   projPltTable[[k, 3]] = HighlightMesh[hull,
     Style[2, Opacity[0.2], Hue[N[((combNumGroups + 1) - k) / combNumGroups]]],
     Frame → True, Axes → False, AspectRatio → aRatio,
     PlotRange → {{xPlotLow, xPlotHi}, {yPlotLow, yPlotHi}}, PlotRangePadding →
      Scaled[pltPad], Ticks → Automatic, FrameLabel → {lab1, lab2},
     LabelStyle → Directive[Black, FontSize → 14, FontFamily → "Arial"],
     ImageSize → pltSize], {k, combNumGroups}]];

If[ptsJoin == 1 && lch == 1, p0 = Show[projPltTable[[All, 1]]]];
If[ptsJoin == 2 && lch == 1,
  p0 = Show[projPltTable[[All, 2]], projPltTable[[All, 1]]]];
If[ptsJoin == 1 && lch == 2,
  p0 = Show[projPltTable[[All, 3]], projPltTable[[All, 1]]]];
If[ptsJoin == 2 && lch == 2, p0 = Show[projPltTable[[All, 3]],
    projPltTable[[All, 2]], projPltTable[[All, 1]]]];

p1 = Labeled[p0, "           PC Score Plot", Top,
   LabelStyle → Directive[FontSize → 18, Bold, FontFamily → "Arial"]];
g1 = Grid[Table[
    {Graphics[{EdgeForm[{Thin, Black}],
       Hue[N[((combNumGroups + 1) - j) / combNumGroups]], Disk[]}]},
    {j, combNumGroups}], Frame → False, ItemSize → 0.9];
g2 = Grid[Partition[combGpNames, 1], Alignment → Left,
   BaseStyle → {FontFamily → "Arial", FontSize → 13, Italic}];
p2 = Labeled[Text[Grid[{{g1, g2}}, Alignment → Bottom, Frame → True]], "Legend",
   Top, LabelStyle → Directive[Black, FontSize → 18, Bold, FontFamily → "Arial"]];

projPlt2D = Grid[{{p1, p2}}, BaselinePosition → Top, Alignment → Top]
```

Export current 2 D plot.

```
In[]:= filenameout = SystemDialogInput["FileSave"];
Export[filenameout, projPlt2D, "TIFF", ImageResolution → 150]

Out[]= /Users/n.macleod/Desktop/Archaeological
  Types/Ford's Houses/Figure 1/Images (Houses + Piers)/Circle
  Groups/PCA Results/PC-3 vs PC-2 (w: figs 2 & 3 Means).tif
```

Label plotted points.

```
In[•]:= pn1 = p0;
projNamePoints = projTmpPoints;
projTempPointsT = Transpose[projTmpPoints];
mxY = Max[projTempPointsT[[2]]];
mnY = Min[projTempPointsT[[2]]];
incY = N[(mxY - mnY) / 16];
Do[projNamePoints[[i, 2]] = projTmpPoints[[i, 2]] - incY, {i, combN}];
nPointsTable =
  Table[{Text[combObjNames[[i]], projNamePoints[[i]], {-1, 0}]}, {i, combN}];
pn2 = Graphics[nPointsTable, Frame → True, AspectRatio → aRatio , Axes → False,
   FrameLabel  → {lab1, lab2}, PlotRangePadding → Scaled[pltPad], BaseStyle →
    Directive[FontSize → 12, FontFamily → "Arial"], ImageSize → pltSize];
p1 = Labeled[Show[pn1, pn2, BaseStyle → {FontFamily → "Arial"}],
   "          PC Score Plot", Top,
   LabelStyle → Directive[FontSize → 18, Bold, FontFamily → "Arial"]];

Plt2D = Grid[{{p1, p2}}, BaselinePosition → Top, Alignment → Top]
```

Export current 2 D plot.

```
filenameout = SystemDialogInput["FileSave"];
Export[filenameout, Plt2D, "TIFF", ImageResolution → 150]
```

Create 3 D scatterplot .

Specify 3 D plot options.
You must run this code after you read in the data so it can pick up the proper variable names.

In[33]:=
```
pcNames = Table[StringJoin["PC-", ToString[i]], {i, noAxes}];
Panel[
 Labeled[Column[{Row[{Panel[Labeled[PopupMenu[Dynamic[xAxisName], pcNames],
        "Select variable to be plotted on x-Axis.", Top, LabelStyle →
         Directive[FontSize → 12, Bold, FontFamily → "Arial"]]], "  ",
      Panel[Labeled[PopupMenu[Dynamic[yAxisName], pcNames],
        "Select variable to be plotted on y-Axis.", Top, LabelStyle →
         Directive[FontSize → 12, Bold, FontFamily → "Arial"]]], "  ",
      Panel[Labeled[PopupMenu[Dynamic[zAxisName], pcNames],
        "Select variable to be plotted on z-Axis.", Top,
        LabelStyle → Directive[FontSize → 12, Bold, FontFamily → "Arial"]]]}],
    Row[{
      Panel[Labeled[PopupMenu[Dynamic[pltAspect],
          {1 → "Golden Ratio Plot", 2 → "Square Plot (equi-length axes)",
           3 → "True-Scale Plot (actual axis scales)"}],
        "Enter plot aspect ratio type.", Top, LabelStyle →
         Directive[FontSize → 12, Bold, FontFamily → "Arial"]]], "  ",
      Panel[Labeled[PopupMenu[Dynamic[lch], {1 → "Simple scatterplot",
           2 → "Scatterplot w/ convex hulls"}],
        "Show group domians?", Top, LabelStyle →
         Directive[FontSize → 12, Bold, FontFamily → "Arial"]]], " ",
      Panel[Labeled[PopupMenu[Dynamic[ptsJoin], {1 → "No", 2 → "Yes"}],
        "Join datapoints?", Top,
        LabelStyle → Directive[FontSize → 12, Bold, FontFamily → "Arial"]]]}],
    Row[{Panel[Labeled[InputField[Dynamic[pltSize], FieldSize → 5],
        "Enter plot size value.", Top,
        LabelStyle → Directive[FontSize → 12, Bold, FontFamily → "Arial"]]],
      " ", Panel[Labeled[InputField[Dynamic[pltPad], FieldSize → 5],
        "Enter plot margin padding value.", Top, LabelStyle →
         Directive[FontSize → 12, Bold, FontFamily → "Arial"]]], " ",
      Panel[Labeled[InputField[Dynamic[iconSize3D], FieldSize → 5],
        "Enter plot icon size value.", Top, LabelStyle →
         Directive[FontSize → 12, Bold, FontFamily → "Arial"]]]}]}, Center],
  "3D Plot Options", Top, LabelStyle → Directive[FontSize → 18,
    Bold, FontFamily → "Arial"]]]
pltSize = 500; iconSize3D = 10; pltPad = 0.1; xAxisName = pcNames[[1]];
 yAxisName = pcNames[[2]];
zAxisName = pcNames[[3]]; ptsJoin = 1;
dataTrans = 1;
 pltAspect = 3 ; lch = 2;
```

··· Table: Iterator {i, mvecs} does not have appropriate bounds.

··· Table: Iterator {i, mvecs} does not have appropriate bounds.

Out[34]=

3D Plot Options

Select variable to be plotted on x–Axis.
PopupMenu[PC–1, Table[PC– <> ToString[i], {i, mvecs}]]

Select variable to be plotted on y–Axis.
PopupMenu[PC–2, Table[PC– <> ToString[i], {i, mvecs}]]

Select variable to be plotted on z–Axis.
PopupMenu[PC–3, Table[PC– <> ToString[i], {i, mvecs}]]

Enter plot aspect ratio type.
True–Scale Plot (actual axis scales)

Show group domians?
Scatterplot w/ convex hulls

Join datapoints?
No

Enter plot size value.
500

Enter plot margin padding value.
0.1

Enter plot icon size value.
10

Part: Part 3 of Table[PC– <> ToString[i], {i, mvecs}] does not exist.

Plot script

In[∘]:=

```
Do[If[xAxisName == pcNames[[j]], axis1 = j], {j, noAxes}]
Do[If[yAxisName == pcNames[[j]], axis2 = j], {j, noAxes}]
Do[If[zAxisName == pcNames[[j]], axis3 = j], {j, noAxes}]
iconSize = iconSize3D - 50.0;

xAxis = combEScoresT[[axis1]];
yAxis = combEScoresT[[axis2]];
zAxis = combEScoresT[[axis3]];
lab1 = StringJoin["CV-", ToString[axis1]];
lab2 = StringJoin["CV-", ToString[axis2]];
lab3 = StringJoin["CV-", ToString[axis3]];
maxx = Max[xAxis];
minx = Min[xAxis];
maxy = Max[yAxis];
miny = Min[yAxis];
maxz = Max[zAxis]; minz = Min[zAxis];

If[pltAspect == 1 || pltAspect == 3,
  xPlotLow = minx; xPlotHi = maxx; yPlotLow = miny; yPlotHi = maxy];
If[pltAspect == 2,
```

```
    If[minx > miny,
     xPlotLow = miny; yPlotLow = miny,
     xPlotLow = minx; yPlotLow = minx ]];
  If[pltAspect == 2,
    If[maxx < maxy,
     xPlotHi = maxy; yPlotHi = maxy,
     xPlotHi = maxx; yPlotHi = maxx]];

  If[pltAspect == 1, bRatio = {1.61803, 1, 1}];
  If[pltAspect == 2, bRatio = {1, 1, 1}];
  If[pltAspect == 3, bRatio = Automatic];

  projPoints3 = Transpose[List[xAxis, yAxis, zAxis]];
  comGp1 = combProjPosns;
  combProjH = Table[0, {combN}];
  Do[
    tmp = comGp1[[i]];
    itr = Length[tmp];
    Do[combProjH[[tmp[[j]]]] = Hue[N[((combNumGroups + 1) - i) / combNumGroups]],
     {j, itr}], {i, combNumGroups}];

  If[lch == 1 && pltAspect == 3,
    projPltPoints = Table[
      {combProjH[[i]], Sphere[projPoints3[[i]], iconSize3D / 55]}, {i, combN}],
    projPltPoints = Table[{combProjH[[i]], AbsolutePointSize[iconSize3D],
       Point[projPoints3[[i]]]}, {i, combN}]];
  p0 = Graphics3D[projPltPoints, Axes → True, Boxed → True,
     PlotRangePadding → Scaled[pltPad],
     LabelStyle → Directive[FontSize → 12, Black, FontFamily → "Arial"],
     AxesLabel → {lab1, lab2, lab3}, ImageSize → pltSize, BoxRatios → bRatio];

  If[ptsJoin == 2,
    projPltLineTable = Table[" ", {combNumGroups}];
    projGpPoints = Table[projPoints3[[combProjPosns[[j]]]], {j, combNumGroups}];
    Do[
     projPltLineTable[[k]] = Graphics3D[
       {Hue[N[((combNumGroups + 1) - k) / combNumGroups]], Line[projGpPoints[[k]]]},
       Axes → True, Boxed → True, PlotRangePadding → Scaled[pltPad],
       LabelStyle → Directive[FontSize → 12, Black, FontFamily → "Arial"],
       AxesLabel → {lab1, lab2, lab3}, ImageSize → pltSize, BoxRatios → bRatio,
       ViewPoint -> {xax, yax, zax}], {k, combNumGroups}];
    pTmp = Graphics3D[projPltPoints, Axes → True, Boxed → True,
      PlotRangePadding → Scaled[pltPad],
      LabelStyle → Directive[FontSize → 12, Black, FontFamily → "Arial"],
      AxesLabel → {lab1, lab2, lab3}, ImageSize → pltSize,
      BoxRatios → bRatio, ViewPoint -> {xax, yax, zax}];
    p0 = Show[{projPltLineTable, pTmp}]];
```

```
If[lch == 2,
  projPltMeshTable = Table[" ", {combNumGroups}];
  projGpPoints = Table[projPoints3[[combProjPosns[[j]]]], {j, combNumGroups}];
  Do[
   projPltPoints =
    Table[{Hue[N[((combNumGroups + 1) - k) / combNumGroups]], AbsolutePointSize[
        iconSize3D], Point[projGpPoints[[k, i]]]}, {i, combNumGroups}];
   pTmp = Graphics3D[projPltPoints, Axes → True, Boxed → True,
     PlotRangePadding → Scaled[pltPad],
     LabelStyle → Directive[FontSize → 12, Black, FontFamily → "Arial"],
     AxesLabel → {lab1, lab2, lab3}, ImageSize → pltSize,
     BoxRatios → bRatio, ViewPoint -> {xax, yax, zax}];
   cHull3D = ConvexHullMesh[projGpPoints[[k]], BaseStyle → {EdgeForm[]},
     Boxed → True, Axes → True, PlotRangePadding → Scaled[pltPad],
     LabelStyle → Directive[FontSize → 12, Black, FontFamily → "Arial"],
     AxesLabel → {lab1, lab2, lab3}, ImageSize → pltSize,
     BoxRatios → bRatio, ViewPoint -> {xax, yax, zax}];
   projPltMeshTable[[k]] = Show[{HighlightMesh[cHull3D,
        Style[2, Opacity[0.2], Hue[N[((combNumGroups + 1) - k) / combNumGroups]]]],
      p0}], {k, combNumGroups}];
  p0 = Show[projPltMeshTable]];

p1 = Labeled[p0, "           PC Score Plot", Top,
   LabelStyle → Directive[FontSize → 18, Bold, FontFamily → "Arial"]];
If[lch == 1 && pltAspect == 3, g1 = Table[
    Graphics[{Inset[Graphics3D[{Hue[N[((combNumGroups + 1) - i) / combNumGroups]],
          Sphere[]}, Boxed → False]]}], {i, combNumGroups}],
  g1 = Table[Graphics[{Inset[Graphics[
         {Hue[N[((combNumGroups + 1) - i) / combNumGroups]],
          Disk[{0, 0}, Scaled[0.3]]}]]}], {i, combNumGroups}]];
g2 = Labeled[Text[Grid[Transpose[Partition[Join[g1, combGpNames],
        combNumGroups]], ItemSize → {{Scaled[0.02], Automatic}}, Alignment → Left,
      BaseStyle → {FontSize → 12, FontFamily → "Arial", Italic},
      Frame → True]], "Legend", Top,
   LabelStyle → Directive[FontSize → 14, Bold, FontFamily → "Arial"]];

projPlt3D = Grid[{{p1, g2}}, Alignment → Top]
```

Part: Part 3 of {{−0.932953, 0.504831, −0.493432}, {−1.41632, −0.210324, −1.64132}} does not exist.

Adjust orientation of 3 D plot (if necessary).
You must replot the data to activate the changes. These changes will be able to be exported using the script below.

In[38]:=
```
Panel[
 Labeled[Row[{Labeled[Slider[Dynamic[xax], {-10, 10}, Appearance → "Labeled"],
      "x-Axis Viewpoint", Top,
      LabelStyle → Directive[FontSize → 10, Bold, FontFamily → "Arial"]] ×
     Labeled[Slider[Dynamic[yax], {-10, 10}, Appearance → "Labeled"],
      "y-Axis Viewpoint", Top,
      LabelStyle → Directive[FontSize → 10, Bold, FontFamily → "Arial"]] ×
     Labeled[Slider[Dynamic[zax], {-10, 10}, Appearance → "Labeled"],
      "z-Axis Viewpoint", Top,
      LabelStyle → Directive[FontSize → 10, Bold, FontFamily → "Arial"]]}],
  "3D Plot Orientation Controls", Top, LabelStyle →
   Directive[FontSize → 14, Bold, FontFamily → "Ariel"]]]
xax = 2.5; yax = -2.5; zax = 2.5;
```

Out[38]=
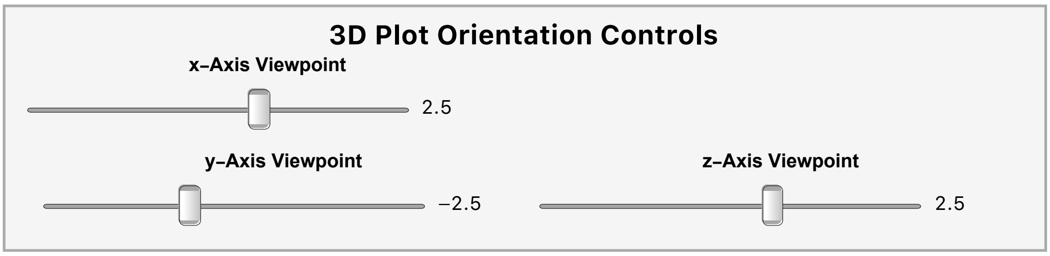

Export current 3 D plot.

In[∘]:=
```
filenameout = SystemDialogInput["FileSave"];
Export[filenameout, plt3D, "TIFF", ImageResolution → 150]
```

Out[∘]=
```
/Users/n.macleod/Desktop/Archaeological Types/Ford's
  Houses/Figure 1/Images (Houses + Piers)/Circle Groups/PCA
  Results/PC-1 vs PC-2 vs PC-3 (w: figs 2 & 3 Means).tif
```

Export projected PCA scores.

```
Proj = Length[Union[projGroup]];
adata = Table[" ", {n4 + 1}, {noAxes + 2}];
adata[[1, 1]] = "Object";
adata[[1, 2]] = "Group";
Do[adata[[i + 1, 1]] = projObjectNames[[i]], {i, n4}]
Do[adata[[i + 1, 2]] = projGroup[[i]], {i, n4}]
Do[
 adata[[1, j + 2]] = StringJoin["PC-", ToString[j]], {j, 1, noAxes}]
Do[adata[[i + 1, j + 2]] = projEScores[[i, j]], {i, n4}, {j, noAxes}]

filenameout = SystemDialogInput["FileSave"];
Export[filenameout, adata, "CSV", "TextDelimiters" → ""]
```
